# Dynamics and regulatory role of circRNAs in Asian honey bee larvae following fungal infection

**DOI:** 10.1007/s00253-024-13102-9

**Published:** 2024-03-13

**Authors:** Rui Guo, Kaiyao Zhang, He Zang, Sijia Guo, Xiaoyu Liu, Xin Jing, Yuxuan Song, Kunze Li, Ying Wu, Haibing Jiang, Zhongmin Fu, Dafu Chen

**Affiliations:** 1 College of Bee Science and Biomedicine, Fujian Agriculture and Forestry University, Fuzhou, 350002 China; 2 National & Local United Engineering Laboratory of Natural Biotoxin, Fuzhou, 350002 China; 3 Apitherapy Research Institute of Fujian Province, Fuzhou, 350002 China; 4 Apiculture Science Institute of Jilin Province, Jilin, Jilin, 132000 China

**Keywords:** *Apis cerana cerana*, *Ascosphaera apis*, circRNA, Antioxidant enzyme, Immune response, Regulatory network

## Abstract

**Abstract:**

Non-coding RNA (ncRNA) plays a vital part in the regulation of immune responses, growth, and development in plants and animals. Here, the identification, characteristic analysis, and molecular verification of circRNAs in *Apis cerana cerana* worker larval guts were conducted, followed by in-depth investigation of the expression pattern of larval circRNAs during *Ascosphaera apis* infection and exploration of the potential regulatory part of differentially expressed circRNAs (DEcircRNAs) in host immune responses. A total of 3178 circRNAs in the larval guts of *A. c. cerana* were identified, with a length distribution ranging from 15 to 96,007 nt. Additionally, 155, 95, and 86 DEcircRNAs were identified in the in the 4-, 5-, and 6-day-old larval guts following *A. apis* infection. These DEcircRNAs were predicted to target 29, 25, and 18 parental genes relevant to 12, 20, and 17 GO terms as well as 144, 114, and 61 KEGG pathways, including 5 cellular and 4 humoral immune pathways. Complex competing endogenous RNA (ceRNA) regulatory networks were detected as being formed among DEcircRNAs, DEmiRNAs, and DEmRNAs. The target DEmRNAs were engaged in 36, 47, and 47 GO terms as well as 331, 332, and 331 pathways, including 6 cellular and 6 humoral immune pathways. Further, 19 DEcircRNAs, 5 DEmiRNAs, and 3 mRNAs were included in the sub-networks relative to 3 antioxidant enzymes. Finally, back-splicing sites within 15 circRNAs and the difference in the 15 DEcircRNAs’ expression between uninoculated and *A. apis*–inoculated larval guts were confirmed based on molecular methods. These findings not only enrich our understanding of bee host–fungal pathogen interactions but also lay a foundation for illuminating the mechanism underlying the DEcircRNA-mediated immune defense of *A. c. cerana* larvae against *A. apis* invasion.

**Key points:**

•* The expression pattern of circRNAs was altered in the A. cerana worker larval guts following A. apis infection.*

•* Back-splicing sites within 15 A. cerana circRNAs were verified using molecular approaches. DEcircRNAs potentially modulated immune responses and antioxidant enzymes in A. apis–challenged host guts.*

**Supplementary Information:**

The online version contains supplementary material available at 10.1007/s00253-024-13102-9.

## Introduction

As the most important pollinating insect in nature, honey bees play an important role in pollinating wild plants and crops and play a pivotal role in the maintenance of biodiversity and ecological balance (Tan et al. 2022). *Apis cerana cerana* is an endemic species in China. It is a subspecies of *A. cerana* and one of the major bee species used in beekeeping production. *Ascosphaera apis*, a specialized fungal pathogen of honey bee larvae, causes chalkbrood disease, which causes a dramatic decrease in colony strength and productivity (Aronstein and Murray 2010).

Circular RNAs (circRNAs), a new class of non-coding RNAs, a large class of non-coding RNAs that are produced by a downstream splice-donor site that is covalently linked to an upstream splice-acceptor site, lack the 5′ cap and the poly(A) tail (Kristensen et al. 2019). Arnberg et al. (1980) first observed yeast mitochondrial circRNA by electron microscopy in 1980. However, due to the limitations of technical means, the research on circRNAs was nearly stagnant for a long time afterward. With the development of high-throughput sequencing technology and the continuous improvement of RNA cyclization prediction algorithms, the systematic identification of circRNAs at the whole transcriptome level has been achieved. In recent years, circRNAs have been shown to play a wide range of roles in gene regulation at the transcriptional level, post-transcriptional level, and translation level. This includes interacting with RNA polymerase II to promote host gene transcription, acting as molecular sponges to regulate miRNA activity and function, influencing variable gene shearing, and encoding small peptides to translate proteins. Abundant circRNAs have been identified and reported in increasing numbers of animals, plants, and microorganisms such as humans (Li et al. 2015; Yang et al. 2018), mice (Maamar et al. 2013), rice (Huang et al. 2021), *Arabidopsis thaliana* (Zhang et al. 2020a, b, c), *Nosema ceranae* (Guo et al. 2018), and *Magnaporthe oryzae* (Yuan et al. 2018). However, research on insect circRNAs is currently still in the preliminary stage. There has been limited documentation for a small number of insects including *Culex pipiens pallens* (Lv et al. 2022), *Apis mellifera* (Ye et al. 2022), *Bombyx mori* (Gan et al. 2017), *Drosophila* (Weigelt et al. 2020), and *Laodelphax striatellus* (Zhang et al. 2020b). Accumulating evidence has demonstrated that circRNAs are engaged in the regulation of interactions between insects and pathogens/parasites (Lv et al. 2022; Zhang et al. 2020b; Hu et al. 2018; Zhu et al. 2022). For example, based on high-throughput sequencing of the midgut tissues of both uninfected and BmCPV-infected *B. mori*, Hu et al. (2018) detected that 400 host circRNAs were significantly differentially expressed following BmCPV infection. In addition, they found that circRNA_9444, circRNA_8115, circRNA_4553, and circRNA_6649 could act as “molecular sponges” to absorb bmo-miR-278-3p, which negatively regulated the insulin-related peptide binding protein 2 gene and thus participated in the BmCPV-*B. mori* interactions. Zhang et al. (2020b) identified 2523 circRNAs in the RBSDV-infected midgut tissues of *Laodelphax striatellus* by transcriptome sequencing and bioinformatics. They further screened eight upregulated and five downregulated circRNAs, indicative of the involvement of these circRNAs in host response to RBSDV infection. Compared with *Drosophila* and *B. mori*, understanding of circRNA in honey bees is fairly limited. Huang et al. (2022a, 2022) identified 33, 144, and 211 DEcircRNAs by resolving the differential expression profiles of *Apis mellifera ligustica* workers at 1, 5, and 10 days after dinotefuran exposure. Further DEcircRNA-miRNA-mRNA regulatory network analysis showed that circ_0008898 and circ_0001829 were potentially involved in the host immune response as “molecular sponges” of miRNAs. Previously, we discovered 10,833 and 9589 circRNAs in *A. m. ligustica* and *A. cerana* workers’ midguts and investigated the potential roles of circRNAs in modulating the development of the midguts (Xiong et al. 2018; Chen et al. 2020). Our previous studies suggested that circRNAs were crucial regulators in responses of bee host to infections by fungal pathogens such as *N. ceranae* and *A. apis*. For example, Chen et al. (2022) predicted 8199 and 8711 circRNAs in the midgut tissues of *A. m. ligustica* workers at 7 days and 10 days post inoculation (dpi) with *N. ceranae*. They found that 16 circRNAs were highly conserved among *Homo sapiens*, *A. m. ligustica*, and *A. c. cerana* and deciphered the expression pattern and regulatory role of DEcircRNAs in the host response. Ye et al. (2022) identified 2083 circRNAs in the *A. m. ligustica* larval guts and analyzed the structural property of circRNAs, followed by investigation of the roles of DEcircRNAs in modulating the host immune response. However, so far, studies on whether and how circRNAs regulate interactions between *A. cerana* larvae and *A. apis* are completely unknown.

In this current study, on the basis of previously obtained high-quality RNA-seq datasets, transcriptome-wide identification of circRNAs in *A. c. cerana* larval guts was conducted, and the expression profile of circRNAs was analyzed. This was followed by investigation of the potential roles of DEcircRNAs in regulating the host response to *A. apis* infection. DEcircRNAs and corresponding target genes relevant to antioxidant enzymes and immune responses in the host guts were further analyzed, and ultimately, validation of back-splicing sites and expression trends of DEcircRNAs were performed. Our data could not only lay a foundation for clarifying the mechanisms underlying circRNA-mediated responses of *A. c. cerana* larvae to *A. apis* infection but also offer new insights into interactions between *A. c. cerana* larvae and *A. apis*.

## Materials and methods

### Fungi and bee larvae

*A. apis* was previously isolated from chalkbrood mummies and by our group (Guo et al. 2018, 2018; Chen et al. 2017b) conserved at China General Microbiological Culture Collection Center (CGMCC NO. 40,895). *A. c. cerana* worker larvae were derived from three colonies reared in the teaching apiary of the College of Animal Sciences (College of Bee Science) at Fujian Agriculture and Forestry University, Fuzhou, China.

### RNA-seq data source

In our previous study, the 4-, 5- and 6-day-old larval guts inoculated with *A. apis* spores (AcT1, AcT2, and AcT3 groups) and the uninoculated 4-, 5- and 6-day-old larval guts (AcCK1, AcCK2, and AcCK3 groups) were prepared and subjected to RNA isolation and strand-specific cDNA-library-based RNA-seq (Chen et al. 2017, 2017b; Xiong et al. 2019). Briefly, the total RNA of three samples in each group was extracted with the TaKaRa MiniBEST Universal RNA Extraction Kit (Takara, Shiga, Japan), and linear RNA was then removed with Rnase R (GEENESEED, Guangzhou, China) after removal of rRNA. The obtained circRNA fragments were fragmented into small fragments using a fragmentation buffer, and the first-strand cDNA was synthesized using random hexamer primers and reverse transcription. Second-strand cDNA synthesis was carried out with DNA polymerase I and RNase H, and the double-stranded cDNAs were then purified using the QiaQuick PCR extraction kit (QIAGEN, Hilden, Germany); the required fragments were then purified by agarose gel electrophoresis followed by enrichment through PCR amplification. The constructed cDNA libraries were sequenced on the Illumina HiSeq™ 2500 platform (GeneDenovo Co., Guangzhou, China). Following our previously described method (Chen et al. 2020, 2020b), the produced raw data were subjected to quality control to gain high-quality clean reads, which were used for downstream bioinformatic analyses. Clean reads were mapped to the *A. cerana* reference genome (assembly ACSNU-2.0), followed by identification of circRNAs according to the described method by Guo et al. (2018d). Different types of circRNAs were then calculated, and raw data were deposited in the NCBI SRA database under the BioProject number: PRJNA560730.

### Screening of DEcircRNAs

The expression level of each circRNA was calculated by RPM (reverse splicing node reads per million mapping) method. Following the standard of *P* < 0.05 and fold change (FC) ≥ 2, DEcircRNAs in every comparison groups were screened. Venn analysis of DEcircRNAs was then performed using the OmicShare platform (https://www.omicshare.com/).

### Prediction and annotation of parental genes of DEcircRNAs

Following the method reported by Chen et al. (2020), the parental genes of DEcircRNAs were predicted by mapping the anchor reads at both ends of DEcircRNAs to the *A. cerana* reference genome (assembly ACSNU-2.0) using Bowtie 2 software (Langmead and Salzberg 2012) with default parameters. If both ends of one circRNA were aligned to the same gene, this gene was regarded as the source gene of the circRNA. Next, the parental genes were annotated to GO (http://www.geneontology.org/) and KEGG (https://www.kegg.jp/) databases by the BLAST tool with default parameters.

### Source of small RNA-seq datasets

In another previous study, *A. apis*–inoculated and uninoculated 4-, 5-, and 6-day-old larval guts of *A. c. cerana* were prepared, followed by RNA isolation, cDNA library construction, and sRNA-seq. Quality control of the raw data was then carried out to gain high-quality clean tags (Wang 2021), which were used for target prediction in this study.

### Analysis of the ceRNA regulatory network

The potential targeting relationships between the DEcircRNAs and DEmiRNAs, as well as those between the DEmiRNAs and DEmRNAs, were predicted using a combination of the TargetFind software (Kiełbasa et al. 2010) and mirTarBase software (Huang et al. 2022a, b). On the basis of the predicted targeting relationships, DEcircRNA-DEmiRNA-DEmRNA regulatory networks were constructed and then visualized using the Cytoscape v.3.2.1 software (Smoot et al. 2011) with default parameters. Further, the targets were mapped to GO and KEGG databases.

### Investigation of antioxidant enzyme–associated DEcircRNAs and the corresponding regulatory network

Antioxidant enzymes like superoxide dismutase (SOD), catalase (CAT), and glutathione *S*-transferase (GST) were used as weapons by insects to combat pathogens or parasites (Ding et al. 2001; Zhang et al. 2023b). Based on the Nr annotations, similarly, the potential targeting relationships between antioxidant enzyme–associated mRNAs and DEmiRNAs, as well as between DEmiRNAs and DEcircRNAs, were predicted with Targetfinder (Kiełbasa et al. 2010) and mirTarBase software (Huang et al. 2022a, 2022). Further, DEcircRNA-DEmiRNA-mRNA regulatory networks were constructed and then visualized by Cytoscape v.3.2.1 software (Smoot et al. 2011).

### Investigation of immune response–related DEcircRNAs and the corresponding regulatory network

The immune system of insects is composed of cellular and humoral immune, which play a pivotal role in host response to infections by pathogens or parasites (Wu and Ling 2009; Bai et al. 2020). The potential targeting relationships between immune-defense-related DEmRNAs and DEmiRNAs, as well as DEmiRNAs and DEcircRNAs, were predicted with Targetfinder (Kiełbasa et al. 2010) and mirTarBase software (Huang et al. 2022a, b). On the basis of the predicted targeting relationships, DEcircRNA-DEmiRNA-DEmRNA regulatory networks were constructed and then visualized by Cytoscape v.3.2.1 software (Smoot et al. 2011).

### Prediction and analysis of DEcircRNAs with coding potential

The internal ribosome entry sites (IRES) contained in DEcircRNA were predicted with IRESfinder software (Zhao et al. 2018). The open-reading frames (ORFs) were predicted using ORFfinder (Pamudurti et al. 2017). The ORFs were annotated to the GO and KEGG databases.

### PCR amplification and Sanger sequencing of circRNAs

To confirm the authenticity of circRNAs, 15 circRNAs were randomly selected for PCR amplification and Sanger sequencing, including novel_circ_000983, novel_circ_001484, novel_circ_002377, novel_circ_002038, novel_circ_002313, novel_circ_002486, novel_circ_000504, novel_circ_001175, novel_circ_002439, novel_circ_000526, novel_circ_000446, novel_circ_001799, novel_circ_001391, novel_circ_002045, and novel_circ_002378. Across the back-splicing sites, a divergent primer was designed using DNAMAN software (shown in Supplemental Table S1) and synthesized by Sangon Biotech (Shanghai) Co., Ltd (China). The TaKaRa MiniBEST Universal RNA Extraction Kit (Takara, Shiga, Japan) was used to extract the total RNA from the total RNA of larval gut samples in the AcCK1, AcCK2, AcCK3, AcT1, AcT2, and AcT3 groups. This was followed by digestion of linear RNA with 3 U/mg RNase R to enrich circRNAs. The template was treated at 37 °C for 15 min, and the cDNA of circRNA was obtained by reverse transcription with random primers. These were then used as templates for PCR amplification, which was conducted on a T100 thermal cycler (BioRad, Hercules, CA, USA). The PCR reaction system consisted of 10 µL of PCR Mix, 2 µL of the DNA template, 1 µL each of upstream and downstream primers (2.5 pmol/µL), and 6 µL of sterile water. The reaction procedure was set as follows: 95 °C for 5 min, 95°C for 30 s, and 60°C for 30 s, for 34 cycles; then, 72 °C for 2 min. The amplified products were detected by 1.5% agarose gel electrophoresis with Ultra GelRed staining (Vazyme, Nanjing, China). This was followed by purification of the target fragments with the FastPure Gel DNA Extraction Mini Kit (Vazyme, Nanjing, China) and then Sanger sequencing by Sangon Biotech (Shanghai) Co., Ltd (China).

### RT-qPCR detection of DEcircRNAs

To further verify the reliability of circRNA sequencing data, five circRNAs were randomly selected from each of the three comparison groups for RT-qPCR validation. The AcCK1 vs. AcT1 group included novel_circ_000983, novel_circ_001484, novel_circ_000882, novel_circ_002486, and novel_circ_002377; the AcCK2 vs. AcT2 group included novel_circ_000504, novel_circ_000405, novel_circ_000526, novel_circ_001175, and novel_circ_002439; the AcCK3 vs. AcT3 group included novel_circ_002378, novel_circ_000102, novel_circ_001799, novel_circ_002486, and novel_circ_001391. The total RNA obtained was divided into two portions: one portion was digested with RNase R to enrich circRNA, and the resulting cDNA obtained by reverse transcription with random primers was used as the templates for RT-qPCR detection of DEcircRNAs; the other portion was subjected to reverse transcription with Oligo dT primers, and the resulting cDNAs were used as the templates for RT-qPCR detection of the internal reference gene actin (gene ID: XM_017059068.2). There were three parallel samples and each experiment was repeated three times. The reaction system followed the method of Ye et al. (2022). The reaction was conducted on Applied Biosystems® QuantStudio 3 (ABI, Waltham, MA, USA) following the conditions: 95 °C pre-denaturation for 5 min, 95 °C denaturation for 15 s, and 60 °C annealing and extension for 30 s, with a total of 40 cycles of qPCR reaction. The relative expression level of each DEcircRNA was calculated using the 2^−∆∆Ct^ method (Livak and Schmittgen 2001). Data were shown as the mean ± standard deviation (SD) and subjected to Student’s *t*-test by Graph Prism 8 software (GraphPad Inc., San Diego, CA, USA). “ns” represents *P* > 0.05, “*” represents *P* < 0.05; “**” represents *P* < 0.01; “***” represents *P* < 0.001. Details of RT-qPCR primers are presented in Supplemental Table S1.

## Results

### Quality control of deep sequencing data

In total, 73,830,148; 96,586,212; 94,552,744; 76,672,564; 90,954,858; and 83,418,832 raw reads were generated in the AcCK1, AcCK2, AcCK3, AcT1, AcT2, and AcT3 groups, respectively (Supplemental Table S2). After undergoing strict quality control, 73,775,592; 96,513,798; 94,495,000; 76,593,924; 90,870,608; and 83,339,288 clean reads were identified, with Q30 above 93.42% (Supplemental Table S2). The results indicated that the sequencing data were of high quality.

### Identification and characterization of the A. c. cerana circRNAs

Based on the total clean reads, 3178 *A. c. cerana* circRNAs were discovered (Supplemental Table S3), with a length distribution ranging from 15 to 96,007 nt; those circRNAs distributed among 301 ~ 400 nt represented the largest group (757, 23.81%) (Supplemental Fig. S1A). The identified circRNAs included five types, among which exonic circRNA (66.76%) was the most abundant type, followed by exon–intron circRNA (15.73%), antisense circRNA (12.30%), intronic circRNA (2.64%), and intergenic region circRNA (2.55%). Additionally, it was found that NW_016019774.1 was the most widely distributed chromosome by circRNAs in the AcCK1 group, while NW_016017967.1 was the most enriched by circRNAs in the AcCK2 and AcCK3 groups; NW_016017455.1 was the most commonly distributed chromosome by circRNAs in the AcT1 group, whereas NW_016017967.1 was the most enriched by circRNAs in the AcT2 and AcT3 groups (Supplemental Fig. S1C).

### Expression profile of circRNAs engaged in larval response to A. apis infection

In the 4-day-old comparison group, 155 DEcircRNAs were screened, including 45 upregulated and 110 downregulated circRNAs (Supplemental Table S4A); in the 5- and 6-day-old comparison groups, 33 and 48 upregulated circRNAs as well as 62 and 38 downregulated circRNAs were detected, respectively (Supplemental Fig. S2A, Supplemental Table S4B,C). Venn analysis showed that 4 upregulated and 1 downregulated circRNAs were shared by the above-mentioned three comparison groups (Table 1), whereas the numbers of unique circRNAs were 125, 70, and 66, respectively (Supplemental Fig. S2B).

**Table 1:** Detailed information about circRNAs shared by 4-, 5-, and 6-day-old comparison groups

ID	Source gene	Chromosome	Strand	Genomic start	Genomic end	Spliced length	Annotated type
novel_circ_001502	ncbi_107996165	NW_016019064.1	-	1,633,180	1,635,297	2118	antisense
novel_circ_002548	ncbi_108001011	NW_016019508.1	+	95,288	95,775	488	one_exon
novel_circ_002606	ncbi_108001152	NW_016019530.1	-	119,107	119,379	273	one_exon
novel_circ_002608	ncbi_108001152	NW_016019530.1	-	119,108	119,311	204	one_exon
novel_circ_002609	ncbi_108001152	NW_016019530.1	-	119,108	119,380	273	one_exon

### Annotation of the DEcircRNAs’ parental genes

**Fig. 1:**
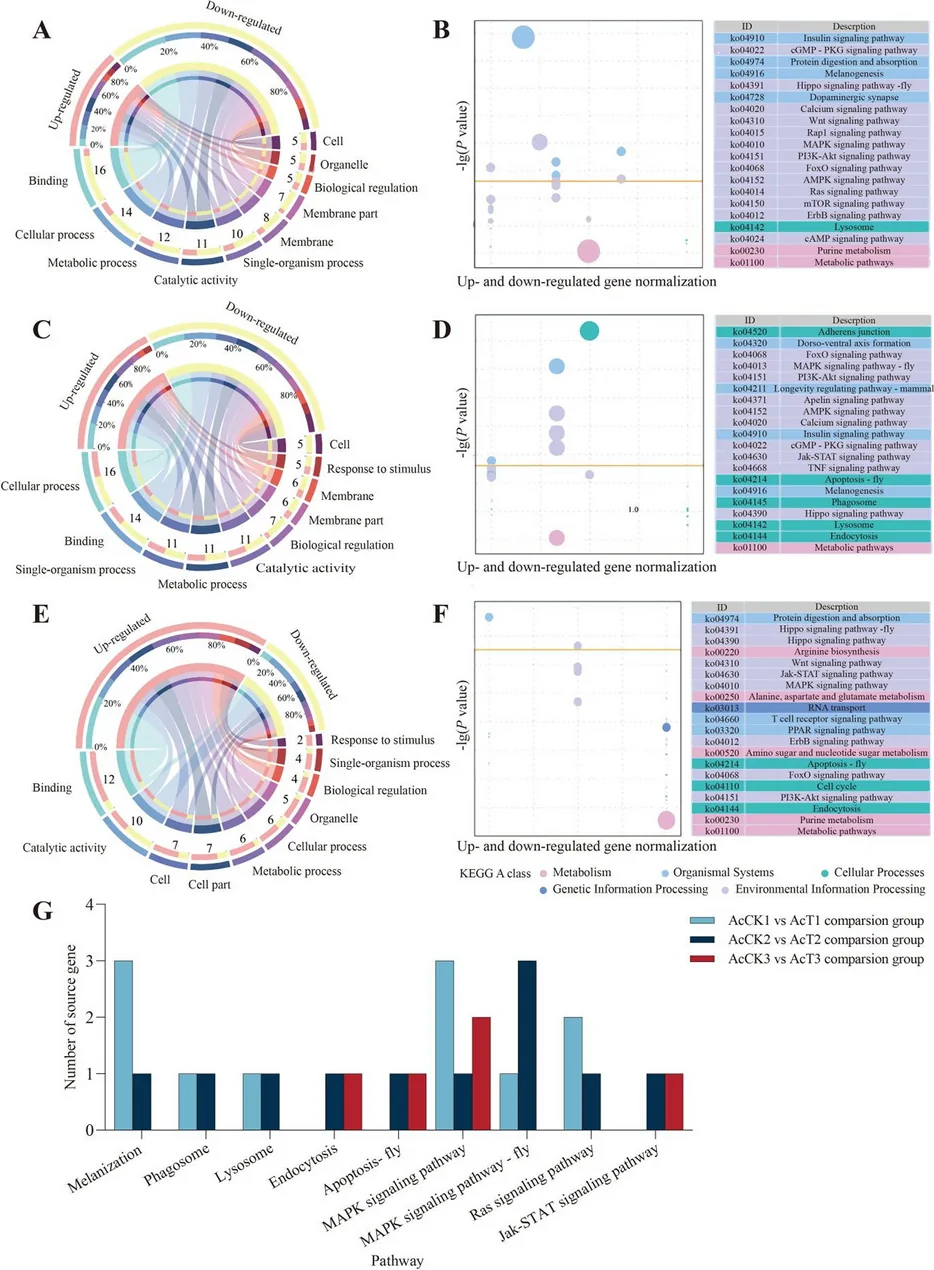
Annotation of parental genes of DEcircRNAs in three comparison groups. **A**, **C**, **E** Loop graphs of GO terms annotated by parental genes; **B**, **D**, **F** KEGG pathways annotated by parental genes; **G** number statistics of parental genes relative to cellular and humoral immune pathways

It is predicted that 29 parental genes of DEcircRNAs in the 4-day-old comparison group were annotated to 12 biological process-associated GO terms such as cellular process and metabolic process, 8 cellular component-associated terms such as membrane part and cell, and 6 molecular function-associated terms such as catalytic activity and transporter activity (Fig. 1A); the parental genes were also enriched in 144 KEGG pathways, such as lysosome, melanogenesis, and PI3K-Akt signaling pathway (Fig. 1B). Comparatively, 25 parental genes of DEcircRNAs in the 5-day-old comparison group were engaged in 20 terms (cellular process, binding, single-organism process, etc.) (Fig. 1C) and 114 pathways (phagosome, apoptosis (fly), MAPK signaling pathway (fly), etc.) (Fig. 1D). In the 6-day-old comparison group, 18 parental genes were involved in 17 terms (binding, catalytic activity, cell, etc.) (Fig. 1E) and 61 pathways (apoptosis, endocytosis, Jak-STAT signaling pathway, etc.) (Fig. 1F). The numbers of parental genes relevant to cellular and humoral immune pathways are summarized in Fig. 1G. Intriguingly, MAPK signaling pathway was observed to be enriched by parental genes of DEcircRNAs in the aforementioned three comparison groups.

### Analysis of ceRNA regulatory networks

**Fig. 2:**
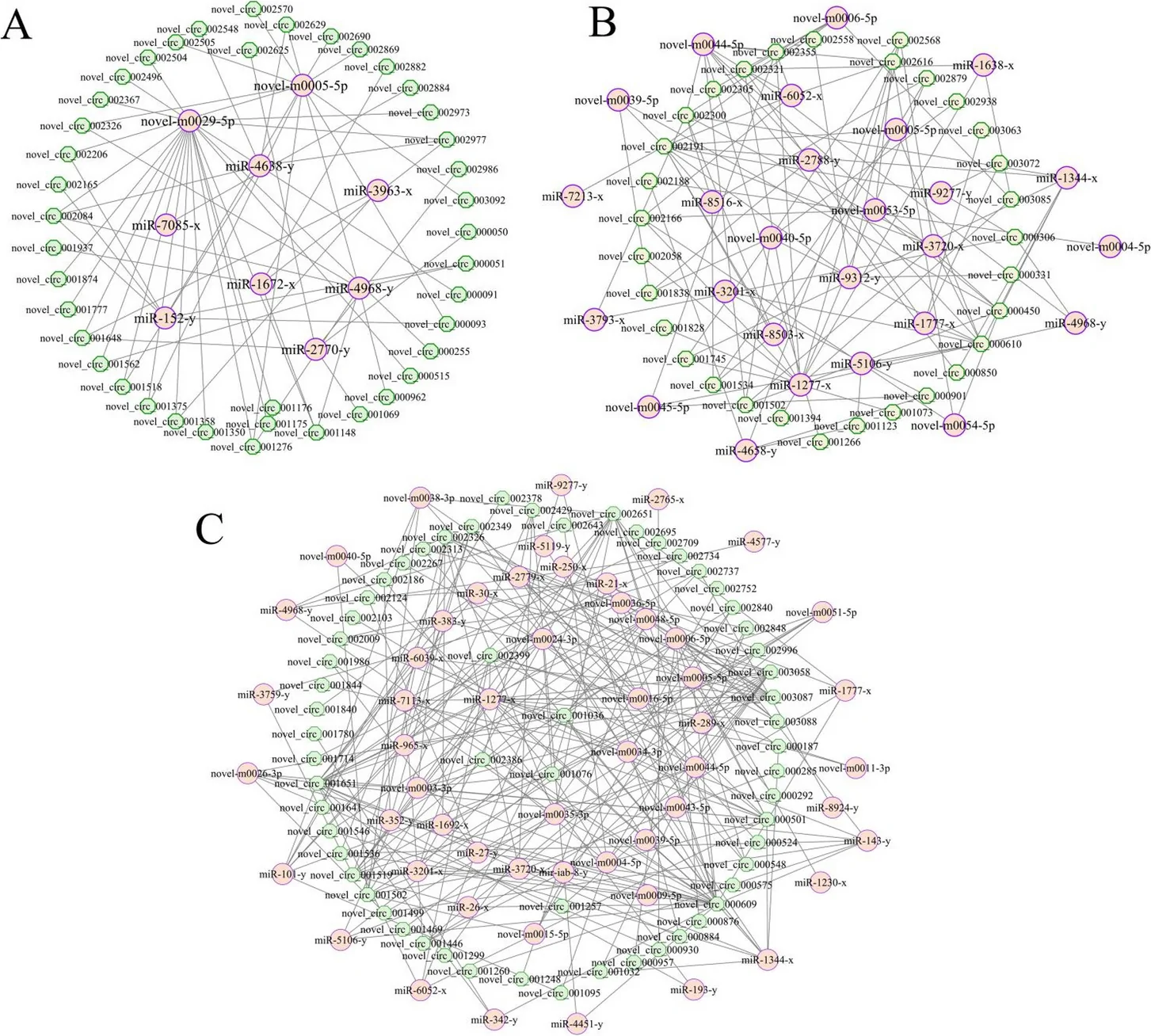
Regulatory networks between DEcircRNAs and DEmiRNAs. **A**–**C** DEcircRNA–DEmiRNA networks in AcCK1 vs. AcT1, AcCK2 vs. AcT2, and AcCK3 vs. AcT3 comparison groups. Hexagons represent DEcircRNAs, while circles represent DEmiRNAs

CeRNA regulatory network analysis demonstrated that 41, 31, and 59 DEcircRNAs in the above-mentioned three comparison groups could target 9, 26, and 54 DEmiRNAs (Fig. 2), further targeting 760, 4464, and 5015 DEmRNAs, respectively. Additionally, a subseries of DEcircRNAs can simultaneously target multiple DEmiRNAs, e.g., novel_circ_002084, novel_circ_002977, and novel_circ_001648 in the 4-day-old comparison group could target 4, 3, and 3 DEmiRNAs. Meanwhile, some DEmiRNAs could also be targeted by several DEcircRNAs at the same time, e.g., miR-1277-x, novel-m0006-5p, and miR-1344-x could be targeted by 15, 10, and 9 DEcircRNAs in the 6-day-old comparison groups.

**Fig. 3:**
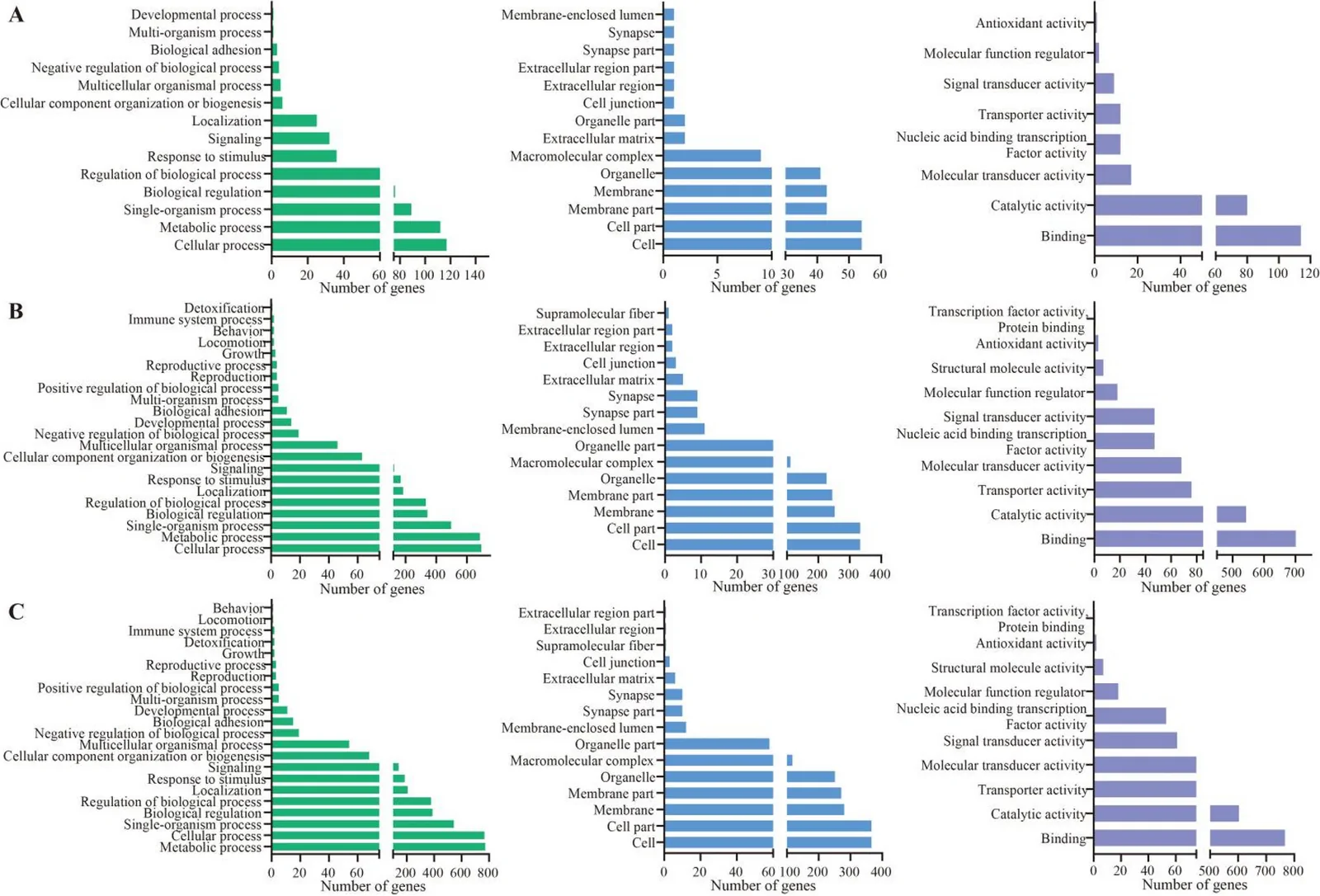
GO terms annotated by targets in DEcircRNA-involved ceRNA regulatory networks. **A**–**C** Biological process, cell component, and molecular function-related terms annotated by targets in the AcCK1 vs. AcT1, AcCK2 vs. AcT2, and AcCK3 vs. AcT3 comparison groups. Green columns represent biological process-related terms; blue columns represent cell component-related terms; purple columns represent molecular function-related terms

**Fig. 4:**
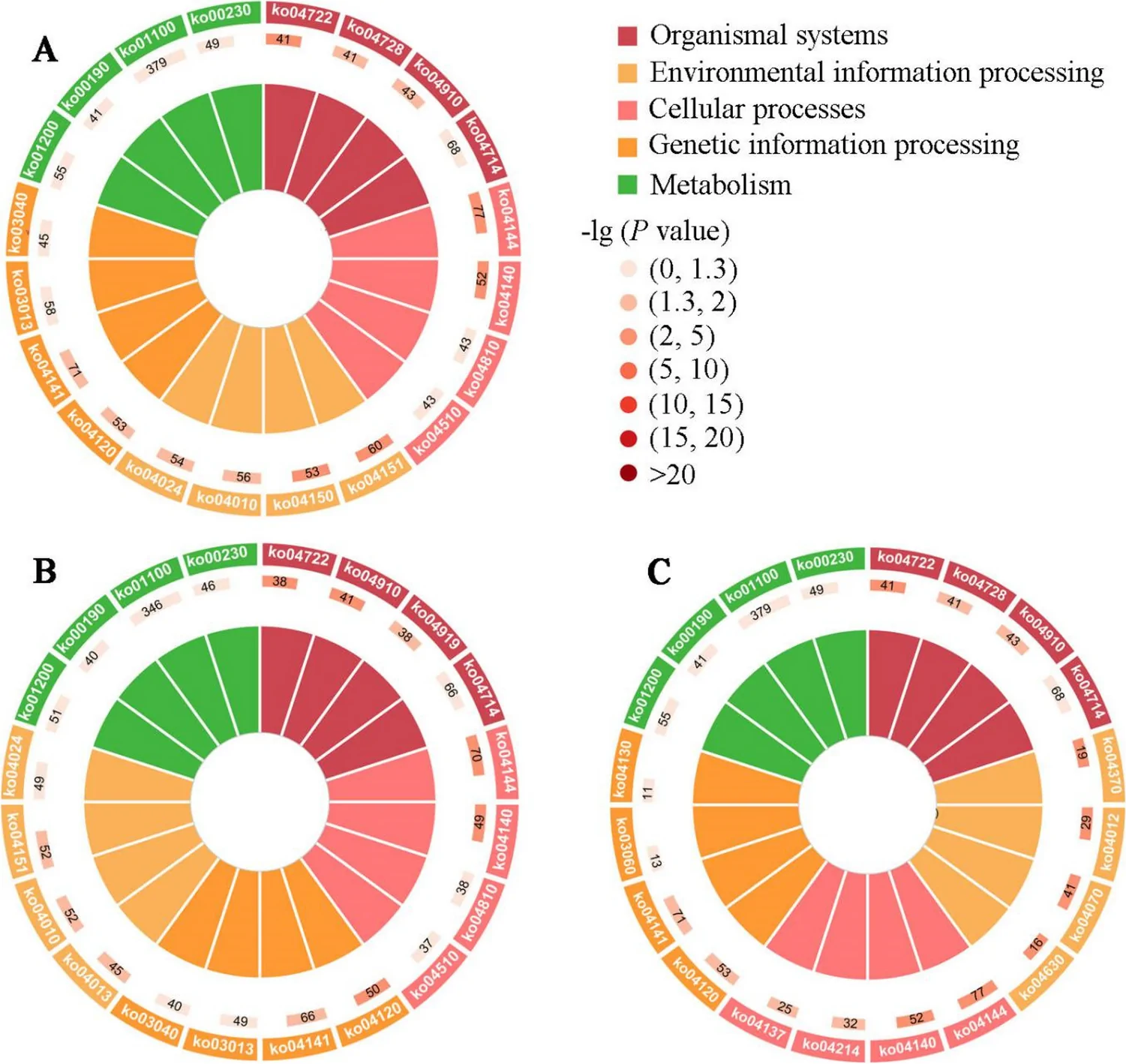
KEGG pathways enriched by targets in DEcircRNA-involved ceRNA regulatory networks. **A**–**C** Pathways enriched by targets in the 4-, 5-, and 6-day-old comparison groups

Targets in the 4-day-old comparison group were involved in 36 GO terms (cellular process, cell, binding, etc.) (Fig. 3A) and 331 KEGG pathways (metabolic pathways, endocytosis, RNA transport, etc.) (Fig. 4A). In contrast, targets in the 5-day-old comparison group were engaged in 47 terms (cellular process, cell, binding, etc.) (Fig. 3B) and 332 pathways (metabolic pathways, endocytosis, MAPK signaling pathway, etc.) (Fig. 4B). In the 6-day-old comparison group, these targets were relevant to 47 terms (metabolic process, cell, binding, etc.) (Fig. 3C), as well as 331 pathways (metabolic pathways, RNA transport, Wnt signaling pathway, etc.) (Fig. 4C).

### Investigation of antioxidant enzyme–associated DEcircRNAs and the corresponding regulatory network

**Fig. 5:**
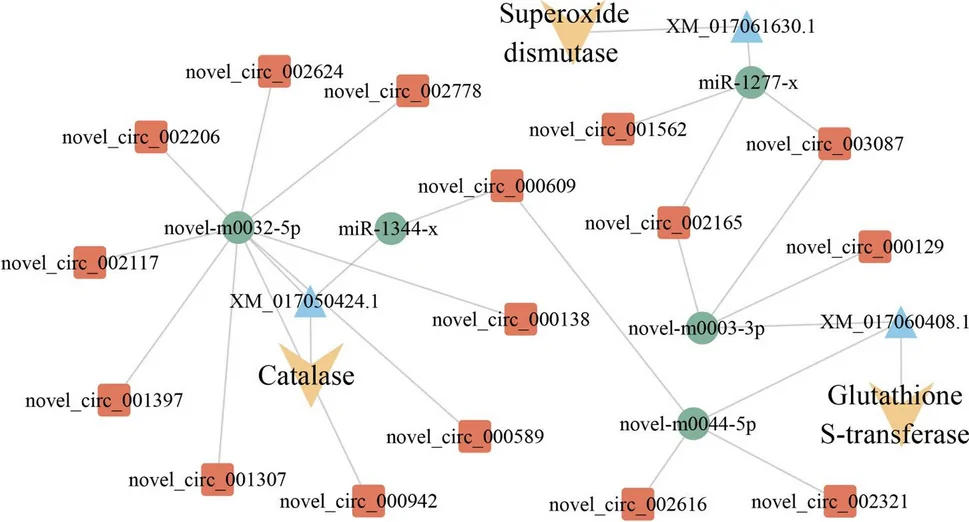
CeRNA regulatory networks of antioxidant enzyme–assisted DEcircRNAs

Further analysis demonstrated that 19 DEcircRNAs, 5 DEmiRNAs, and 3 mRNAs shared by the 4-, 5-, and 6-day-old comparison groups were included in the sub-networks relative to three antioxidant enzymes including superoxide dismutase (SOD), catalase (CAT), and glutathione *S*-transferase (GST) (Fig. 5, see also Supplemental Table S5). In detail, 3 DEcircRNAs potentially targeted 1 DEmiRNA, further targeting 1 mRNA associated with superoxide dismutase (Fig. 5, see also Supplemental Table S5); 10 DEcircRNAs putatively targeted 2 DEmiRNAs, further targeting 1 mRNA related to catalase (Fig. 5, see also Supplemental Table S5); 6 DEcircRNAs potentially targeted 2 DEmiRNAs, further targeting 1 mRNA relevant to glutathione *S*-transferase (Fig. 5, see also Supplemental Table S5).

### Analysis of immune-defense-related DEcircRNAs and the corresponding regulatory network

**Fig. 6:**
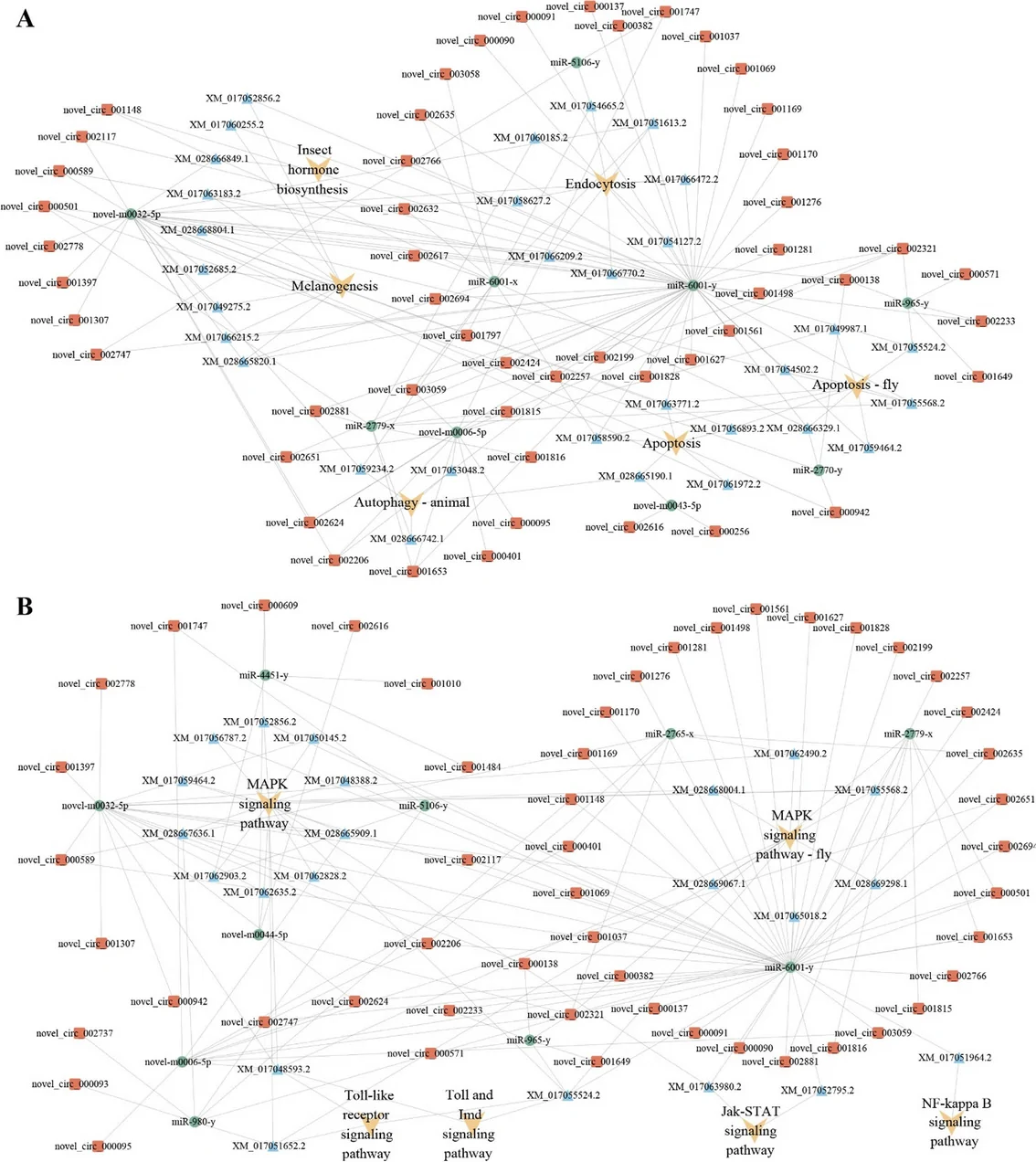
CeRNA regulatory networks of cellular and humoral immune-related DEcircRNAs

It was observed that immune-defense-related sub-networks included 56 DEcircRNAs, 13 DEmiRNAs, and 49 DEmRNAs for the 4-, 5-, and 6-day-old comparison groups, respectively (Fig. 6, see also Supplemental Table S6). In detail, 51 DEcircRNAs putatively targeted 9 DEmiRNAs, further targeting 31 DEmRNAs involved in 6 cellular immune-related pathways, including apoptosis, melanogenesis, endocytosis, autophagy (animal), apoptosis (fly), and insect hormone biosynthesis (Fig. 6, see also Supplemental Table S6); 51 DEcircRNAs potentially targeted 10 DEmiRNAs, further targeting 22 DEmRNAs engaged in 6 humoral immune-related pathways, such as the Toll and Imd, Toll-like receptors, NF-kappa B, Jak-STAT, and MAPK signaling pathways (Fig. 6, see also Supplemental Table S6). In addition, 46 circRNAs were found to be involved in the regulatory networks regarding both cellular and humoral immune responses.

### Investigation of the protein-coding potential of DEcircRNA

In the 4-day-old comparison group, 27 IRESs and 61 ORFs within DEcircRNAs were discovered; these ORFs were engaged in 23 GO terms such as cellular processes and signaling (Supplemental Table S7A), as well as 107 KEGG pathways such as the insulin signaling pathway and melanogenesis (Supplemental Table S8A). In the 5-day-old comparison group, 26 IRESs and 52 ORFs within DEcircRNAs were identified; these ORFs were relative to 20 functional terms including binding and the response to stimulus (Supplemental Table S7B), as well as 71 pathways including metabolic pathways and the MAPK signaling pathway (Supplemental Table S8B). Additionally, 24 IRESs and 40 ORFs within DEcircRNAs in the 6-day-old comparison group were detected; these ORFs were involved in 17 functional terms such as catalytic activity and cell processes (Supplemental Table S7C), as well as 16 pathways such as endocytosis and apoptosis (Supplemental Table S8C).

### Molecular verification of back-splicing sites within DEcircRNAs

**Fig. 7:**
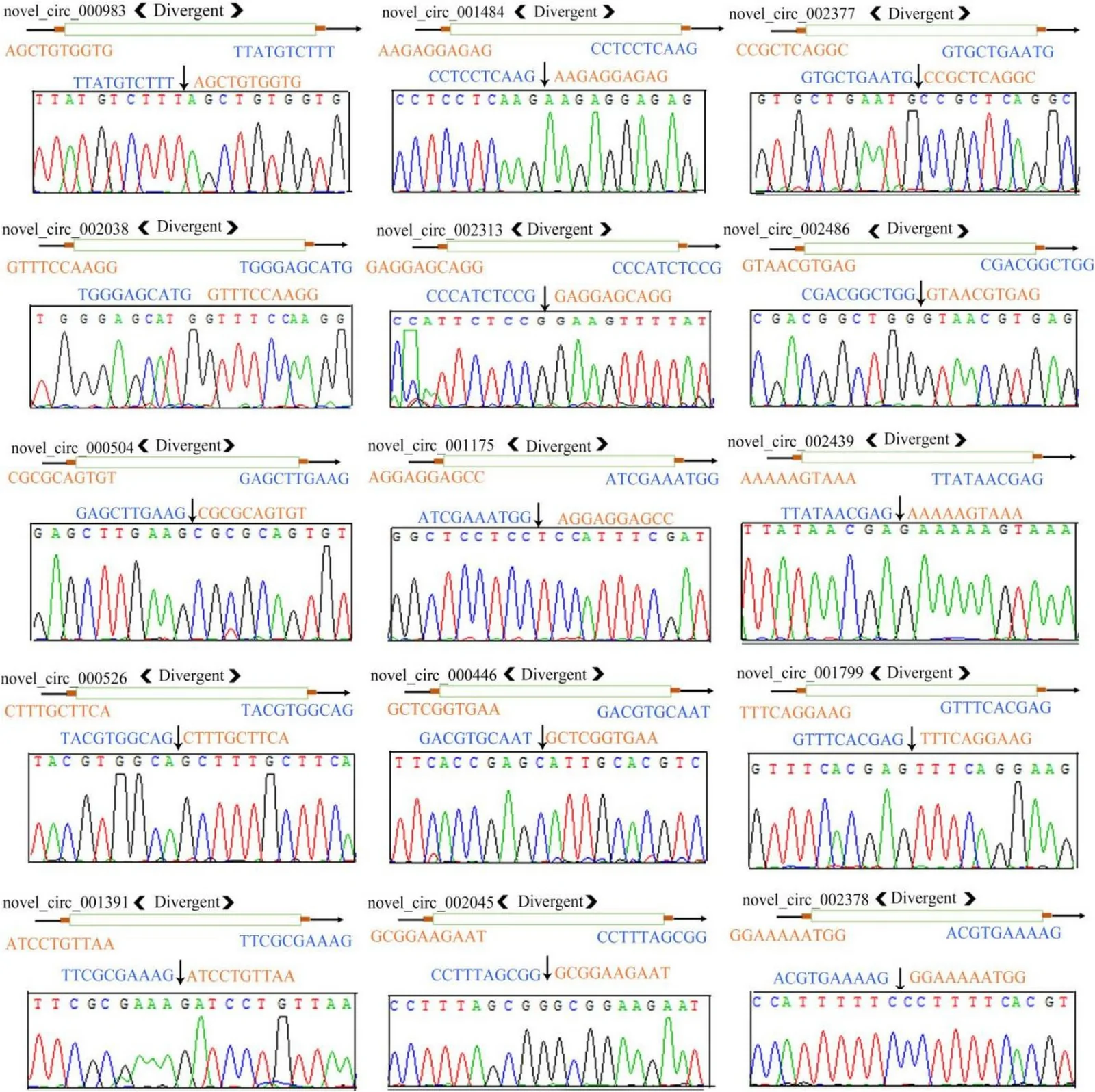
Sanger sequencing of amplification products from 15 circRNAs. “<” and “>” indicate the direction of amplification using divergent primers; “ → ” indicates the transcriptional directions of circRNAs; “↓” indicates the back-splicing sites within circRNAs

Five randomly selected DEcircRNAs from each comparison group were subjected to PCR amplification followed by Sanger sequencing. The Sanger results indicated that the sequences of 15 DEcircRNAs were in accordance with those in the prediction results, based on deep sequencing data. The results confirmed the authenticity of the back-splicing sites in these DEcircRNAs (Fig. 7).

### RT-qPCR detection of DEcircRNAs

**Fig. 8:**
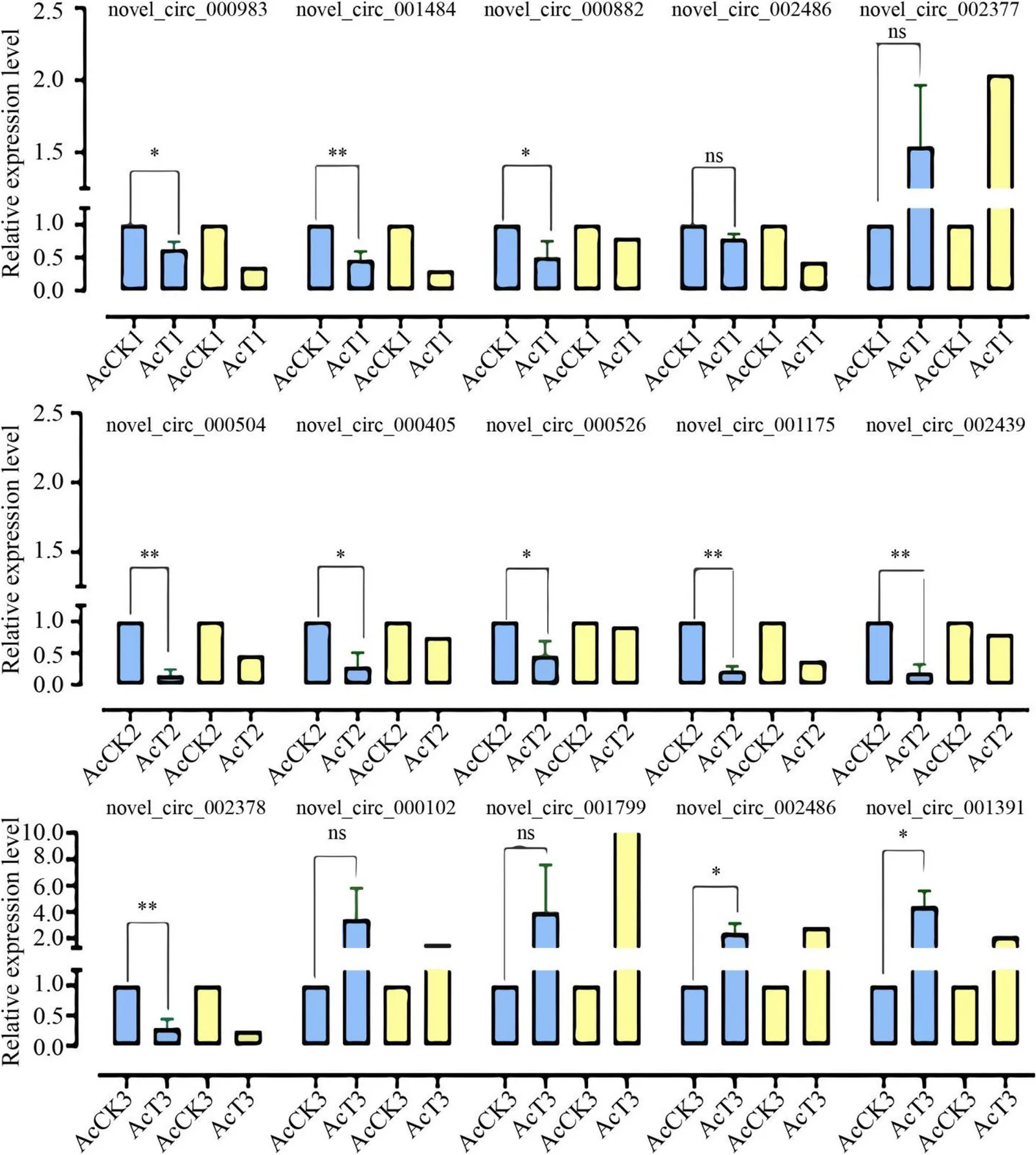
RT-qPCR detection of 15 DEcircRNAs. Blue columns, RT-qPCR; yellow columns, RNA-seq. The experimental data were presented as mean ± SD and subjected to Student’s t-test; ns, P > 0.05; *P < 0.05; **P < 0.01

Further, RT-qPCR of the aforementioned 15 DEcircRNAs was conducted. The results indicated that the expression trends between *A. apis*–inoculated and uninoculated 4-, 5-, and 6-day-old larval guts were in accordance with those in the prediction results, based on deep sequencing data (Fig. 8), thus validating the reliability of the transcriptome datasets used in this work.

## Discussion

### A. c. cerana circRNAs shared similar splicing signal and structural property with other animal species

Our group identified 3178 circRNAs of infected 4–6-day-old larvae and uninfected 4–6-day-old larvae of *A. c. cerana* (Supplemental Table S3). The number of circRNAs identified by previous studies in humans (Memczak et al. 2013), mice (Memczak et al. 2013), nematodes (Memczak et al. 2013), and soybean (Wang et al. 2020a, b) species differed significantly. The number of circRNAs was also significantly different from that of *A. c. cerana* worker larvae identified in this study, indicating that genes of different species can be transcribed to form different amounts of circRNAs. However, differences in library construction methods, bioinformatics algorithms, analysis software setup parameters, individuals of different species, and sequencing tissues in different studies may also be important reasons for the significant differences in the identified number of circRNAs. Additionally, we found that the back-splicing sites of *A. c. cerana* circRNA contained the conserved splicing signal GT/AG. This was similar to splicing signals discovered in other animals such as humans (Liu et al. 2019), mice (Zhang et al. 2021), silkworms (Hu et al. 2018), and *Chiloscyllium plagiosum* (Zhang et al. 2020c), indicating that this splicing signal was conserved among animals. For circRNAs identified in both *A. apis–*infected and uninfected groups, the major splicing signal was GT/AG, which suggested that the *A. apis* infection could not alter this stable splicing signal. Here, it was observed that circRNAs distributed among 301 ~ 400 nt represented the largest group, analogous to those identified in humans (Hossain et al. 2022), mice (Hossain et al. 2022), and rice (Wang et al. 2019). Also, we detected that annotated exonic circRNA was the most abundant type, as shown in Fig. 1B. This is similar to the findings from humans (Tang et al. 2020), *A. m. ligustica* (Xiong et al. 2018), *A. thaliana* (Chen et al. 2017), and *Zea mays* (Ma et al. 2021).

### DEcircRNAs were putative regulators in host response to A. apis infection by controlling the transcription of parental genes

In the present study, 155, 95, and 86 DEcircRNAs were discovered in the 4-, 5-, and 6-day-old comparison groups, respectively (Fig. 1), indicating that *A. apis* infection gave rise to the alteration of the overall expression pattern of circRNAs in *A. c. cerana* worker larval guts. These DEcircRNAs were potential regulators in the larval *A. apis* response. In addition, 5 DEcircRNAs covering two types of circulation, such as novel_circ_001502, novel_circ_002548, novel_circ_002606, novel_circ_002608, and novel_circ_002609 were shared by the 4-, 5-, and 6-day-old comparison groups. It is inferred that these 5 shared DEcircRNAs played critical roles in the host response to *A. apis* infection and thus deserve further investigation.

Accumulating evidence has showed that some circRNAs modulate the transcription of parental genes and further affect an array of biological processes such as immune response and development (Wang et al. 2021; Zhou et al. 2021). In this study, we found that 3178 circRNAs were generated from 1356 parental genes; among these, some parental genes produced only one circRNA, while others produced two or more circRNAs, similar to the reported findings from studies on *Drosophila* (Tay et al. 2017) and silkworms (Gan et al. 2017). In addition, 296 DEcircRNAs were detected as being derived from 225 parental genes. This could mean that a single linear mRNA principally generated only one circRNA, and some could yield two or more circRNAs, suggesting that a complicated circularization mechanism indeed occurred in circRNA biogenesis during *A. apis* infection of *A. c. cerana*. This was annotated to a series of functional terms such as cellular process, metabolic process, cell, organelle, catalytic activity, and binding. In addition, the parental genes mentioned above were also relevant to 184 pathways, including melanogenesis, lysosomes, metabolic pathways, FoxO, hippo, and the MAPK signaling pathway. Together, the results suggest that these DEcircRNAs may have participated in the modulation of the aforementioned life activities by regulating the transcription of corresponding parental genes.

The insulin signaling pathway is evolutionarily conserved and interacts with insect’s innate immunity, while acting synergistically with insect ecdysteroid to induce cellular autophagy and apoptosis in larval tissues, playing a key role in insect growth and development, reproductive metabolism, and resilience (Chen et al. 2017d). In insects such as *B. mori* (Okamoto et al. 2009), *Drosophila melanogaster* (Chen et al. 2022), *Schistocerca gregaria* (Dillen et al. 2016), and *Anopheles gambiae* (Arsic and Guerin 2008), several related insulin-like genes have been identified and shown to be closely associated with growth, development, metabolism, and immune defense. After mutating the gene encoding the insulin receptor in *Drosophila*, Drummond-Barbosa and Spradling (2001) observed that ovarian follicles regressed in *Drosophila* and blocked entry into vitellogenesis. Here, we found 6 and 2 parental genes in the 4- and 5-day-old comparison groups, corresponding to 7 and 3 DEcircRNAs, respectively, that could be annotated to the insulin signaling pathway; however, no parental gene in the 6-day-old comparison group was observed as being annotated to this pathway. The insulin receptor catalyzes the autophosphorylation and activation of the intermediary molecule growth-factor-receptor-linked protein Grb2 and the SOS molecule with guanylate exchange factor activity. This is followed by the activation of the Raf protein, which further activates the MAPK signaling pathway due to its serine/threonine protein kinase activity (Cargnello and Roux 2011). In eukaryotes, the MAPK signaling pathway, a vital pathway response to oxidative stress, transfers extracellular information to the interior of the cell, further regulating cell activities in response to external stimuli (Hotamisligil and Davis 2016). As an important member of the MAPK signaling pathway, MKK is involved in modulating cell growth and the immune response (Kim and Choi 2010). Wang et al. (2018) previously identified an *AccMKK6* gene in *A. c. cerana* and detected that the activities of SOD and POD (peroxidase) were significantly reduced, and the antioxidant capacity of bees was decreased after knockdown of *AccMKK6* in adult workers via RNAi. Here, 3, 1, and 2 parental genes, corresponding to 3, 1, and 2 DEcircRNAs, respectively, could be annotated to the MAPK signaling pathway (Fig. 1G). Collectively, the results demonstrated that these DEcircRNAs were likely to participate in the host response to *A. apis* infection by regulating the transcription of parental genes relative to the three above-mentioned signaling pathways.

### DEcircRNAs potentially regulated antioxidant enzyme–relevant gene expression to participate in host A. apis response

Increasingly, studies have shown that circRNAs are capable of acting as “molecular sponges” for miRNA to regulate the expression of genes and further affect biological processes such as metabolism and immunity (Hansen et al. 2013; Zhao et al. 2017, 2022; Panda 2018). In this current study, 41, 31, and 59 DEcircRNAs in the 4-, 5-, and 6-day-old comparison groups were found to target 9, 26, and 54 DEmiRNAs, respectively (Fig. 2). This is suggestive of the potential of these host DEcircRNAs to act as a “molecular sponge” for miRNAs during the infection process of *A. apis*. We observed the significant downregulation of miR-1277-x in the *A. c. cerana* 6-day-old worker larval gut following *A. apis* infection and found that miR-1277-x putatively targeted a gene belonging to the JAK/STAT signaling pathway. Here, three upregulated circRNAs (novel_circ_003058, novel_circ_003088, and novel_circ_002651) were found to simultaneously target miR-1277-x, further targeting a downstream gene encoding the transcriptional regulator Myc-B. This implied that the *A. apis* infection resulted in the activation of novel_circ_003058, novel_circ_003088, and novel_circ_002651 in the larval guts, which may attenuate the inhibitory effect of miR-1277-x on the expression of *Myc-B*, thereby modulating the Jak-STAT signaling pathway. However, more efforts are needed to verify this speculation.

Insects possess a suite of antioxidant enzymes and antioxidants with small molecular weights, which form a concatenated response to an onslaught of dietary and endogenously produced oxidants. Antioxidant enzymes such as superoxide dismutase, catalase, glutathione transferase, and glutathione reductase have been characterized in insects (Felton and Summers 1995). Ling and Zhang (2013) documented that CAT mRNA was specifically induced in the presence of chlorpyrifos, suggesting that the intensified CAT enzyme activities contributed to enhancing the antioxidant capacity and population growth of *Nilaparvata lugens*. Li et al. (2022) identified 31 cytosolic GST genes in *Spodoptera litura*, including *SlGSTd1*, a GST gene from the delta cluster. They found that silencing *SlGSTd1* significantly increased the cumulative mortality after fenvalerate treatment for 72 h and cyhalothrin treatment for 48, 60, and 72 h. Also, studies have shown that antioxidant enzymes are involved in the response of insects to pathogenic microorganisms (Lalitha et al. 2018). Vivekanandhan et al. (2022) showed, in *S. litura* larvae, that SOD levels were increased when the *Metarhizium flavoviride* conidia concentrations were increased, and the insect antioxidant enzymes played a significant role in ROS (reactive oxygen species) eradication. Various xenobiotics can cause oxidative stress and the production of ROS in insects; Zhang et al. (2023a) detected that after *Beauveria bassiana* infected the larvae of *Spodoptera frugiperda*, the activities of SOD and CAT in the larvae initially increased, followed by a decreasing trend. In a previous study, we found that *A. apis* inoculation of *A. cerana* larvae led to chalkbrood disease, reduced the larval survival rate, and significantly affected the activities of SOD, CAT, GST, and PPO (prophenoloxidae) (Zhang et al. 2023b). This is indicative of the involvement of these four crucial antioxidant enzymes in the host response to *A. apis* infection. Here, 19 DEcircRNAs, 5 DEmiRNAs, and 3 mRNAs shared by the 4-, 5-, and 6-day-old comparison groups were associated with three antioxidant enzymes of great importance, including SOD, CAT, and GST (Fig. 5). This suggested that the DEcircRNA-mediated ceRNA network is potentially engaged in regulating the response of *A. c. cerana* worker larvae to *A. apis* infection. In the near future, functional dissection of key DEcircRNAs (e.g., RNAi via feeding siRNA targeting back-splicing sites) (Ye et al. 2023) and miRNAs (overexpression and knockdown via feeding the mimic and inhibitor) (Wu et al. 2023) will be conducted to explore the ceRNA metabolism underlying the *A. c. cerana* larval response to *A. apis* invasion.

### DEcircRNAs potentially modulated host immune response to *A. apis* infection via ceRNA networks

When they are exposed to pathogens or parasites, insects initiate hemolymph-mediated cellular immune and fat-body-mediated humoral immune responses to defend against infection (Hillyer 2016). Phagocytosis and encapsulation are the classical insect cellular immune pathways, in which phagocytosis mainly engulfs pathogens with small molecular sizes, including bacteria and viruses, while pathogens with larger molecular sizes, such as parasites and nematodes, are removed by encapsulation and colonization (Hillyer 2016). Lemaitre and Hoffmann (2007) reported that large numbers of lamellocytes can be induced to differentiate from hemocyte precursors upon infection of larvae with parasitoid wasp eggs, forming a vesicular complex with melanism or producing ROS to engulf and kill the contents. Apoptosis, as a key component of the cellular immune system, is an active programmed cell death under polygenic control and plays an important role in the cellular response to infections by various pathogens. Zhang et al. (2002) found that infection of *Spodopteralitura* by Autographa californica multicapsid nucleopolyhedro virus (AcMNPV) induced the activation of host cell apoptosis at the early stage to suppress viral propagation and spread. Here, cellular immune-relevant sub-networks were investigated; the results showed that 51 DEcircRNAs in the 4-, 5-, and 6-day-old comparison groups potentially targeted 9 DEmiRNAs and further targeted 31 DEmRNAs. These target DEmRNAs were involved in 6 cellular immune pathways, such as apoptosis, melanogenesis, endocytosis, autophagy (animal), apoptosis (fly), and insect hormone biosynthesis (Fig. 6A). Toll, Imd, and JAK/STAT are three vital signaling pathways, among which, the Toll signaling pathway mainly responds to Gram-positive and fungal infestations, while the Imd signaling pathway mainly responds to Gram-negative infestations (Alejandro et al. 2022). The JAK/STAT signaling pathway is mainly involved in antiviral natural immunity and melanism (Myllymäki and Rämet 2014). It has been suggested that the Toll and Imd signaling pathways regulate insect gut flora and maintain gut immune homeostasis (Zhai et al. 2018; Sun et al. 2019). Both the Toll and Imd signaling pathways result in the activation of Nf-*κ*b transcription factors and translocation into the nucleus, where they upregulate the expression of genes encoding AMPs (antimicrobial peptides) and other genes (Palmer and Jiggins 2015). Lin et al. (2018) analyzed the expression profiles of immune genes in the fourth-instar *Plutella xylostella* larval midgut infected by *Staphylococcus aureus*, *Escherichia coli*, or *Pichia pastoris*. They found that the expression levels of immune genes related to the Toll, Imd, and JAK/STAT signaling pathways were significantly upregulated, which is suggestive of the participation of these three immune pathways in host immunity. Sun et al. (2019) detected that the Toll receptor genes *Toll 1A* and *Toll 5A* in *Anopheles stephensi* were highly activated in response to *B. bassiana* infection; *Toll 1A* and *Toll 5A* regulated the homeostasis of gut microbiota. After suppressing the JAK-STAT signaling pathway by silencing the negative regulator PIAS in *Aedes aegypti* based on RNAi, Souza-Neto et al. (2009) observed that the expression levels of one putative Toll-receptor-associated gene were upregulated, and the host susceptibility to dengue virus infection significantly decreased. In this study, target DEmRNAs were engaged in 6 humoral immune pathways, including the Toll and Imd, Toll-like receptor, NF-kappa B, Jak-STAT, and MAPK signaling pathways (Fig. 6B). In summary, these results demonstrated that corresponding DEcircRNAs and their involved ceRNA network were likely to regulate the expression of the aforementioned immune-pathway-related genes, followed by participation in the response of *A. c. cerana* worker larvae to *A. apis* infection.

### DEcircRNAs potentially modulated host response to *A. apis* invasion by encoding proteins

Unlike linear RNAs, circRNAs lack the 5′ cap and the poly(A) tail; hence, they cannot encode proteins through classical translation mechanisms. However, recent studies have suggested that some circRNAs including IRES elements and ORFs are able to encode small peptides or proteins with a biological function (Shi et al. 2020). Zhang et al. (2022) identified that vSP27, translated from a BmCPV-derived circular RNA, induced the generation of ROS and activated the NF-κB signaling pathway, induced the expression of antimicrobial peptides, and suppressed BmCPV infection. In silkworms, circEgg encoded circEgg-P122, a protein with 122 amino acid residues, and inhibited trimethylation of histone H3 and lysine 9 (Wang et al. 2020a, b). In this current study, 27, 26, and 24 IRES as well as 61, 52, and 40 ORFs were identified in DEcircRNAs in the 4-, 5-, and 6-day-old comparison groups, respectively. This implied that these DEcircRNAs had protein-coding potential. Additionally, the ORFs within DEcircRNAs could be annotated to the cAMP signaling pathway, metabolic pathways, and ABC transporters, in addition to several immune pathways such as the insulin signaling pathway, melanogenesis, phagosomes, lysosomes, the MAPK signaling pathway (fly), apoptosis (fly), and endocytosis. Together, these results indicate that some DEcircRNAs may modulate many aspects during the larval response to *A. apis* infection by encoding related proteins.

**Fig. 9:**
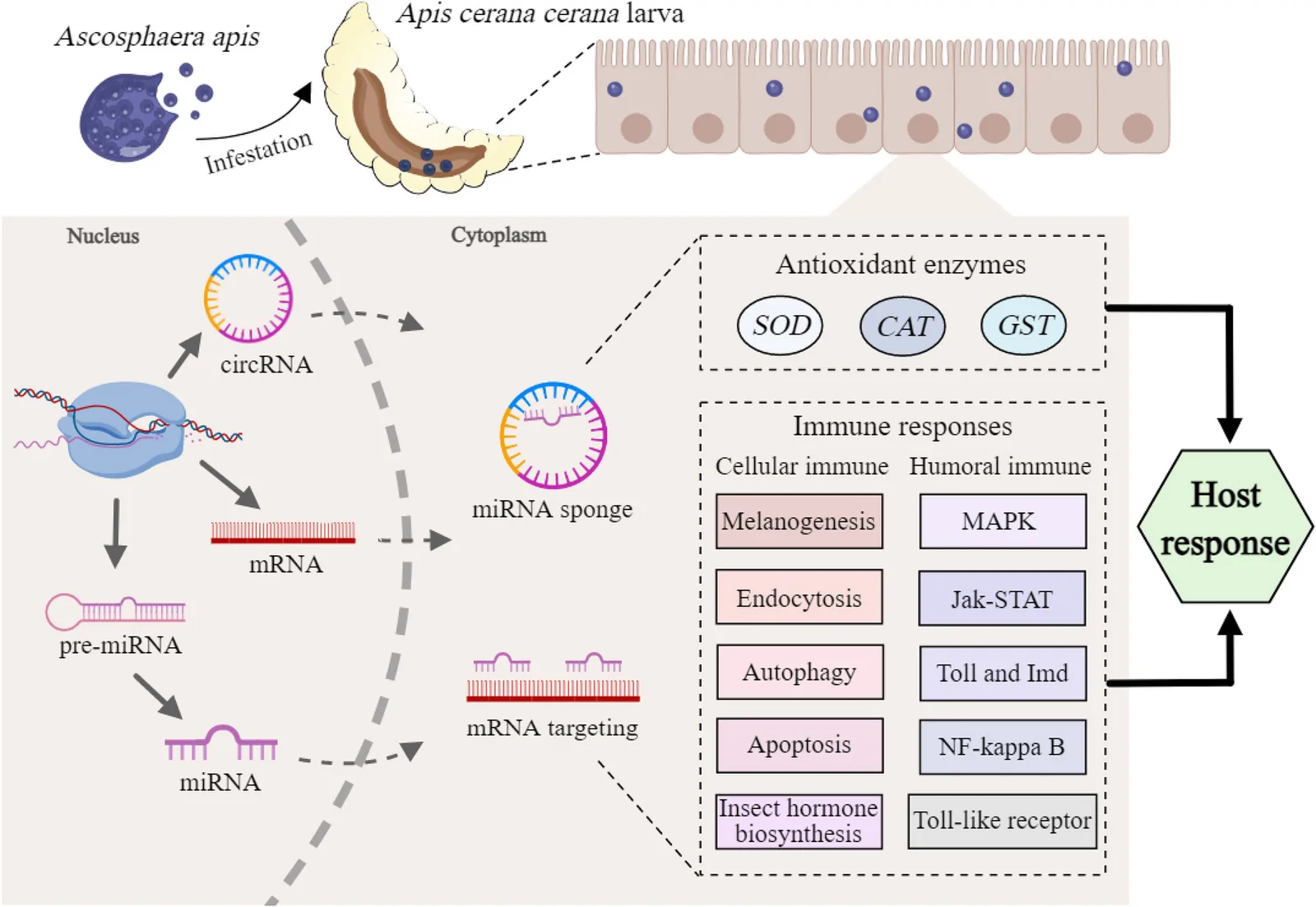
**A** hypothetical schematic diagram of circRNA-mediated immune responses of A. c. cerana larvae to A. apis invasion. This diagram was created with MedPeer website (www.medpeer.cn)

In conclusion, 3178 circRNAs were identified in the *A. c. cerana* worker larval guts. The expression pattern of circRNAs in the larval guts was changed due to *A. apis* invasion; corresponding DEcircRNAs were potentially engaged in host responses including the immune response to *A. apis* infection through versatile mechanisms (Fig. 9), such as the regulation of the transcription of parental genes, absorption of target miRNAs via ceRNA networks, and translation into proteins. The findings from this study lay a foundation for further investigation of the function and mechanism of circRNAs regulating the host *A. apis* response, provide novel and valuable insights into the interactions between *A. cerana* larvae and *A. apis*, and offer candidate biomarkers and molecular targets for the diagnosis and control of chalkbrood disease.

## Supplementary Information

Below is the link to the electronic supplementary material.

Supplementary file1 (PDF 1027 KB)

## Data Availability

All data generated or analyzed during this study are included in this published article.

## References

[CR1] Alejandro AD, Lilia JP, Jesús MB, Henry RM (2022) The IMD and Toll canonical immune pathways of *Triatoma pallidipennis* are preferentially activated by Gram-negative and Gram-positive bacteria respectively but cross-activation also occurs. Parasit Vectors 15:256. 10.1186/s13071-022-05363-y 35821152 10.1186/s13071-022-05363-y PMC9277830

[CR2] Arnberg AC, Van OGJ, Grivell LA, Van BEF, Borst P (1980) Some yeast mitochondrial RNAs are circular. Cell 19:313–319. 10.1016/0092-8674(80)90505-x 6986989 10.1016/0092-8674(80)90505-x

[CR3] Aronstein KA, Murray KD (2010) Chalkbrood disease in honey bees. J Invertebr Pathol 103(Suppl 1):S20–S29. 10.1016/j.jip.2009.06.018 19909969 10.1016/j.jip.2009.06.018

[CR4] Arsic D, Guerin PM (2008) Nutrient content of diet affects the signaling activity of the insulin/target of rapamycin/p70 S6 kinase pathway in the African malaria mosquito * Anopheles gambiae*. J Insect Physiol 54:1226–1235. 10.1016/j.jinsphys.2008.06.003 10.1016/j.jinsphys.2008.06.003 18634792

[CR5] Bai J, Xu Z, Li L, Ma W, Xu L, Ma L (2020) Temporospatial modulation of Lymantria dispar immune system against an entomopathogenic fungal infection. Pest Manag Sci 76:3982–3989. 10.1002/ps.5947 32506667 10.1002/ps.5947

[CR6] Cargnello M, Roux PP (2011) Activation and function of the MAPKs and their substrates the MAPK-activated protein kinases. Microbiol Mol Biol Rev 75:50–83. 10.1128/MMBR.00031-10 21372320 10.1128/MMBR.00031-10 PMC3063353

[CR7] Chen D, Guo R, Xu X, Xiong C, Liang Q, Zheng Y, Luo Q, Zhang Z, Huang Z, Kumar D, Xi W, Zou X, Liu M (2017) Uncovering the immune responses of *Apis mellifera ligustica* larval gut to *Ascosphaera apis* infection utilizing transcriptome sequencing. Gene 621:40–50. 10.1016/j.gene.2017.04.022 28427951 10.1016/j.gene.2017.04.022

[CR8] Chen D, Guo R, Xiong C, Liang Q, Zheng Y, Xu X, Zhang Z, Huang Z, Zhang L, Wang H, Xie Y, Tong X (2017b) Transcriptome of *Apis cerana cerana* larval gut under the stress of *Ascosphaera apis*. Sci Agric Sin 50:2614–2623 (**(In Chinese)**)

[CR9] Chen D, Chen H, Du Y, Zhu Z, Wang J, Geng S, Xiong C, Zheng Y, Hou C, Diao Q, Guo R (2020) Systematic identification of circular RNAs and corresponding regulatory networks unveil their potential roles in the midguts of eastern honeybee workers. Appl Microbiol Biotechnol 104:257–276. 10.1007/s00253-019-10159-9 31754765 10.1007/s00253-019-10159-9

[CR10] Chen G, Cui J, Wang L, Zhu Y, Lu Z, Jin B (2017) Genome-wide identification of circular RNAs in *Arabidopsis thaliana*. Front Plant Sci 8:1678. 10.3389/fpls.2017.01678 29021802 10.3389/fpls.2017.01678 PMC5623955

[CR11] Chen H, Fan X, Zhang W, Ye Y, Cai Z, Zhang K, Zhang K, Fu Z, Chen D, Guo R (2022) Deciphering the circRNA-regulated response of Western honey bee (*Apis mellifera*) workers to microsporidian invasion. Biology (Basel) 11:1285. 10.3390/biology11091285 36138764 10.3390/biology11091285 PMC9495892

[CR12] Chen H, Fu Z, Wang J, Zhu Z, Fan X, Jiang H, Fan Y, Zhou D, Li W, Xiong C, Zheng Y, Xu G, Chen D, Guo R (2020b) Circular RNA response of *Apis cerana cerana* 6-day-old larvae to *Ascosphaera apis* stress. Acta Microbiol Sin 60:2292–2310 (**(In Chinese)**)

[CR13] Chen J, Huang Y, Qi G (2022) LncRNA-IRAR-mediated regulation of insulin receptor transcripts in *Drosophila melanogaster* during nutritional stress. Insect Mol Biol 31:261–272. 10.1111/imb.12756 34923706 10.1111/imb.12756

[CR14] Chen X, Yao H, Ye G (2017d) Research advances on insulin-like peptides and their functions in insects. Chin J Bio Control 33:699–712 (**(In Chinese)**)

[CR15] Dillen S, Chen Z, Vanden BJ (2016) Nutrient-dependent control of short neuropeptide F transcript levels via components of the insulin/IGF signaling pathway in the desert locust *Schistocerca gregaria*. Insect Biochem Mol Biol 68:64–70. 10.1016/j.ibmb.2015.11.007 26631598 10.1016/j.ibmb.2015.11.007

[CR16] Ding SY, Li HY, Li XF, Zhang ZY (2001) Effects of two kinds of transgenic poplar on protective enzymes system in the midgut of larvae of American white moth. J for Res 12:119–122. 10.1007/BF02867209

[CR17] Drummond-Barbosa D, Spradling AC (2001) Stem cells and their progeny respond to nutritional changes during *Drosophila* oogenesis. Dev Biol 231:265–278. 10.1006/dbio.2000.0135 11180967 10.1006/dbio.2000.0135

[CR18] Felton GW, Summers CB (1995) Antioxidant systems in insects. Arch Insect Biochem Physiol 29:187–197. 10.1002/arch.940290208 7606043 10.1002/arch.940290208

[CR19] Gan H, Feng T, Wu Y, Liu C, Xia Q, Cheng T (2017) Identification of circular RNA in the *Bombyx mori* silk gland. Insect Biochem Mol Biol 89:97–106. 10.1016/j.ibmb.2017.09.003 28918159 10.1016/j.ibmb.2017.09.003

[CR20] Guo R, Chen D, Chen H, Xiong C, Zheng Y, Hou C, Du Y, Geng S, Wang H, Ding Z, Yi G (2018) Genome-wide identification of circular RNAs in fungal parasite *Nosema ceranae*. Curr Microbiol 75:1655–1660. 10.1007/s00284-018-1576-z 30269253 10.1007/s00284-018-1576-z

[CR21] Guo R, Chen D, Chen H, Fu Z, Xiong C, Hou C, Zheng Y, Guo Y, Wang H, Du Y, Diao Q (2018) Systematic investigation of circular RNAs in *Ascosphaera apis* a fungal pathogen of honeybee larvae. Gene 678:17–22. 10.1016/j.gene.2018.07.076 30077766 10.1016/j.gene.2018.07.076

[CR22] Guo R, Chen D, Xiong C, Hou C, Zheng Y, Fu Z, Diao Q, Zhang L, Wang H, Hou Z, Li W, Kumar D, Liang Q (2018) Identification of long non-coding RNAs in the chalkbrood disease pathogen *Ascospheara apis*. J Invertebr Pathol 156:1–5. 10.1016/j.jip.2018.06.001 29894727 10.1016/j.jip.2018.06.001

[CR23] Guo R, Chen H, Xiong C, Zheng Y, Fu Z, Xu G, Du Y, Wang H, Geng S, Zhou D, Liu S, Chen D (2018d) Analysis of differentially expressed circular RNAs and their regulation networks during the developmental process of *Apis mellifera ligustica* worker’s midgut. Sci Agric Sin 51:4575–4590 (**(In Chinese)**)

[CR24] Hansen TB, Jensen TI, Clausen BH, Bramsen JB, Finsen B, Damgaard CK, Kjems J (2013) Natural RNA circles function as efficient microRNA sponges. Nature 495:384–348. 10.1038/nature11993 23446346 10.1038/nature11993

[CR25] Hillyer JF (2016) Insect immunology and hematopoiesis. Dev Comp Immunol 58:102–118. 10.1016/j.dci.2015.12.006 26695127 10.1016/j.dci.2015.12.006 PMC4775421

[CR26] Hossain MT, Zhang J, Reza MS, Peng Y, Feng S, Wei Y (2022) Reconstruction of full-length circRNA sequences using chimeric alignment information. Int J Mol Sci 23:6776. 10.3390/ijms23126776 35743218 10.3390/ijms23126776 PMC9223815

[CR27] Hotamisligil GS, Davis RJ (2016) Cell signaling and stress responses. Cold Spring Harb Perspect Biol 8:a006072. 10.1101/cshperspect.a006072 27698029 10.1101/cshperspect.a006072 PMC5046695

[CR28] Hu X, Zhu M, Zhang X, Liu B, Liang Z, Huang L, Xu J, Yu J, Li K, Zar MS, Xue R, Gao G, Gong C (2018) Identification and characterization of circular RNAs in the silkworm midgut following *Bombyx mori* cytoplasmic polyhedrosis virus infection. RNA Biol 15:292–301. 10.1080/15476286.2017.1411461 29268657 10.1080/15476286.2017.1411461 PMC5798952

[CR29] Huang HY, Lin YC, Cui S, Huang Y, Tang Y, Xu J, Bao J, Li Y, Wen J, Zuo H, Wang W, Li J, Ni J, Ruan Y, Li L, Chen Y, Xie Y, Zhu Z, Cai X, Chen X, Yao L, Chen Y, Luo Y, LuXu S, Luo M, Chiu CM, Ma K, Zhu L, Cheng GJ, Bai C, Chiang YC, Wang L, Wei F, Lee TY, Huang HD (2022a) miRTarBase update 2022: an informative resource for experimentally validated miRNA-target interactions. Nucleic Acids Res 50(D1):D222–D230. 10.1093/nar/gkab1079 34850920 10.1093/nar/gkab1079 PMC8728135

[CR30] Huang M, Dong J, Guo H, Xiao M, Wang D (2022) Identification of circular RNAs and corresponding regulatory networks reveals potential roles in the brains of honey bee workers exposed to dinotefuran. Pestic Biochem Physiol 180:104994. 10.1016/j.pestbp.2021.104994 34955187 10.1016/j.pestbp.2021.104994

[CR31] Huang X, Zhang H, Guo R, Wang Q, Liu X, Kuang W, Song H, Liao J, Huang Y, Wang Z (2021) Systematic identification and characterization of circular RNAs involved in flag leaf senescence of rice. Planta 253:26. 10.1007/s00425-020-03544-6 33410920 10.1007/s00425-020-03544-6 PMC7790769

[CR32] Kiełbasa SM, Blüthgen N, Fähling M, Mrowka R (2010) Targetfinder.org: a resource for systematic discovery of transcription factor target genes. Nucleic Acids Res 38(Web Server issue):W233-W238. 10.1093/nar/gkq374 10.1093/nar/gkq374 PMC2896086 20460454

[CR33] Kim EK, Choi EJ (2010) Pathological roles of MAPK signaling pathways in human diseases. Biochim Biophys Acta 1802:396–405. 10.1016/j.bbadis.2009.12.009 20079433 10.1016/j.bbadis.2009.12.009

[CR34] Kristensen LS, Andersen MS, Stagsted LVW, Ebbesen KK, Hansen TB, Kjems J (2019) The biogenesis biology and characterization of circular RNAs. Nat Rev Genet 20:675–691. 10.1038/s41576-019-0158-7 31395983 10.1038/s41576-019-0158-7

[CR35] Lalitha K, Karthi S, Vengateswari G, Karthikraja R, Perumal P, Shivakumar MS (2018) Effect of entomopathogenic nematode of *Heterorhabditis indica* infection on immune and antioxidant system in lepidopteran pest *Spodoptera litura* (Lepidoptera: Noctuidae). J Parasit Dis 42:204–211. 10.1007/s12639-018-0983-1 29844624 10.1007/s12639-018-0983-1 PMC5962492

[CR36] Langmead B, Salzberg SL (2012) Fast gapped-read alignment with Bowtie 2. Nat Methods 9:357–9. 10.1038/nmeth.1923 22388286 10.1038/nmeth.1923 PMC3322381

[CR37] Lemaitre B, Hoffmann J (2007) The host defense of *Drosophila* melanogaster. Annu Rev Immunol 25:697–743. 10.1146/annurev.immunol.25.022106.141615 17201680 10.1146/annurev.immunol.25.022106.141615

[CR38] Li D, Xu L, Liu H, Chen X, Zhou L (2022) Metabolism and antioxidant activity of *SlGSTD1* in *Spodoptera litura* as a detoxification enzyme to pyrethroids. Sci Rep 12:10108. 10.1038/s41598-022-14043-x 35710787 10.1038/s41598-022-14043-x PMC9203748

[CR39] Li F, Zhang L, Li W, Deng J, Zheng J, An M, Lu J, Zhou Y (2015) Circular RNA ITCH has inhibitory effect on ESCC by suppressing the Wnt/β-catenin pathway. Oncotarget 6:6001–13. 10.18632/oncotarget.3469 25749389 10.18632/oncotarget.3469 PMC4467417

[CR40] Lin J, Xia X, Yu XQ, Shen J, Li Y, Lin H, Tang S, Vasseur L, You M (2018) Gene expression profiling provides insights into the immune mechanism of *Plutella xylostella* midgut to microbial infection. Gene 647:21–30. 10.1016/j.gene.2018.01.001 29305978 10.1016/j.gene.2018.01.001

[CR41] Ling S, Zhang H (2013) Influences of chlorpyrifos on antioxidant enzyme activities of *Nilaparvata lugens*. Ecotoxicol Environ Saf 98:187–90. 10.1016/j.ecoenv.2013.08.023 24064262 10.1016/j.ecoenv.2013.08.023

[CR42] Liu Z, Ran Y, Tao C, Li S, Chen J, Yang E (2019) Detection of circular RNA expression and related quantitative trait loci in the human dorsolateral prefrontal cortex. Genome Biol 20:99. 10.1186/s13059-019-1701-8 31109370 10.1186/s13059-019-1701-8 PMC6528256

[CR43] Livak KJ, Schmittgen TD (2001) Analysis of relative gene expression data using real-time quantitative PCR and the 2(-Delta Delta C(T)) Method. Methods 25:402–408. 10.1006/meth.2001.1262 11846609 10.1006/meth.2001.1262

[CR44] Lv Y, Li X, Zhang H, Zou F, Shen B (2022) CircRNA expression profiles in deltamethrin-susceptible and -resistant *Culex pipiens pallens* (Diptera: Culicidae). Comp Biochem Physiol B Biochem Mol Biol 261:110750. 10.1016/j.cbpb.2022.110750 35513264 10.1016/j.cbpb.2022.110750

[CR45] Ma P, Gao S, Zhang HY, Li BY, Zhong HX, Wang YK, Hu HM, Zhang HK, Luo BW, Zhang X, Liu D, Wu L, Gao DJ, Gao SQ, Zhang SZ, Gao SB (2021) Identification and characterization of circRNAs in maize seedlings under deficient nitrogen. Plant Biol (Stuttg) 23:850–860. 10.1111/plb.13280 33932084 10.1111/plb.13280

[CR46] Maamar H, Cabili MN, Rinn J, Raj A (2013) *linc-HOXA1* is a noncoding RNA that represses *Hoxa1* transcription in *cis*. Genes Dev 27:1260–71. 10.1101/gad.217018.113 23723417 10.1101/gad.217018.113 PMC3690399

[CR47] Memczak S, Jens M, Elefsinioti A, Torti F, Krueger J, Rybak A, Maier L, Mackowiak SD, Gregersen LH, Munschauer M, Loewer A, Ziebold U, Landthaler M, Kocks C, le Noble F, Rajewsky N (2013) Circular RNAs are a large class of animal RNAs with regulatory potency. Nature 495:333–338. 10.1038/nature11928 23446348 10.1038/nature11928

[CR48] Myllymäki H, Rämet M (2014) JAK/STAT pathway in *Drosophila* immunity. Scand J Immunol 79:377–385. 10.1111/sji.12170 24673174 10.1111/sji.12170

[CR49] Okamoto N, Yamanaka N, Satake H, Saegusa H, Kataoka H, Mizoguchi A (2009) An ecdysteroid-inducible insulin-like growth factor-like peptide regulates adult development of the silkmoth *Bombyx mori*. FEBS J 276:1221–1232. 10.1111/j.1742-4658.2008.06859.x 19175674 10.1111/j.1742-4658.2008.06859.x

[CR50] Palmer WJ, Jiggins FM (2015) Comparative genomics reveals the origins and diversity of arthropod immune systems. Mol Biol Evol 32:2111–29. 10.1093/molbev/msv093 25908671 10.1093/molbev/msv093 PMC4833078

[CR51] Pamudurti NR, Bartok OJM, Ashwal-Fluss R, Stottmeister C, Ruhe L, Hanan M, Wyler E, Perez-Hernadez D, Ramberger E, Shenzis S, Samson M, Dittmar G, Landthaler M, Chekulaeva M, Rajewsky N, Kadener S (2017) Translation of CircRNAs. Mol Cell 66:9-21.e7. 10.1016/j.molcel.2017.02.021 28344080 10.1016/j.molcel.2017.02.021 PMC5387669

[CR52] Panda AC (2018) Circular RNAs act as miRNA sponges. Adv Exp Med Biol 1087:67–79. 10.1007/978-981-13-1426-1_6 30259358 10.1007/978-981-13-1426-1_6

[CR53] Shi Y, Jia X, Xu J (2020) The new function of circRNA: translation. Clin Transl Oncol 22:2162–2169. 10.1007/s12094-020-02371-1 32449127 10.1007/s12094-020-02371-1

[CR54] Smoot ME, Ono K, Ruscheinski J, Wang PL, Ideker T (2011) Cytoscape 2.8: new features for data integration and network visualization. Bioinformatics 27:431–2. 10.1093/bioinformatics/btq675 21149340 10.1093/bioinformatics/btq675 PMC3031041

[CR55] Souza-Neto JA, Sim SZ, Dimopoulos G (2009) An evolutionary conserved function of the JAK-STAT pathway in anti-dengue defense. PNAS 106:17841–17846. 10.1073/pnas.0905006106 19805194 10.1073/pnas.0905006106 PMC2764916

[CR56] Sun PL, Cui CL, Song HS, Wang SB (2019) Toll receptors are involved in anti-microbial response and gut microbiota homeostasis in the malaria vector *Anopheles stephensi* (Diptera: Culicidae). Acta Entomolo Sin 62:937–947 (**(In Chinese)**)

[CR57] Tan S, Li G, Liu Z, Wang H, Guo X, Xu B (2022) Effects of glyphosate exposure on honeybees. Environ Toxicol Pharmacol 90:103792. 10.1016/j.etap.2021.103792 34971799 10.1016/j.etap.2021.103792

[CR58] Tang M, Kui L, Lu G, Chen W (2020) Disease-associated circular RNAs: from biology to computational identification. Biomed Res Int 2020:6798590. 10.1155/2020/6798590 32908906 10.1155/2020/6798590 PMC7450300

[CR59] Tay ML, Pek JW (2017) Maternally inherited stable intronic sequence RNA triggers a self-reinforcing feedback loop during development. Curr Biol 27:1062–1067. 10.1016/j.cub.2017.02.040 28343963 10.1016/j.cub.2017.02.040

[CR60] Vivekanandhan P, Swathy K, Alford L, Pittarate S, Subala SPRR, Mekchay S, Elangovan D, Krutmuang P (2022) Toxicity of *Metarhizium flavoviride* conidia virulence against *Spodoptera litura* (Lepidoptera: Noctuidae) and its impact on physiological and biochemical activities. Sci Rep 12:16775. 10.1038/s41598-022-20426-x 36202839 10.1038/s41598-022-20426-x PMC9537412

[CR61] Wang H (2021) The mechanism of microRNAs regulating gut development and response to stress by *Apis cerana cerana* larvae in response to the stress of *Ascosphaera apis.* Master’s thesis Fujian Agriculture and Forestry University Fu Zhou China (In Chinese)

[CR62] Wang G, Sun Q, Wang H, Liu H (2021) Identification and characterization of circRNAs in the liver of blunt snout bream (*Megalobrama amblycephala*) infected with *Aeromonas hydrophila*. Dev Comp Immunol 124:104185. 10.1016/j.dci.2021.104185 34174243 10.1016/j.dci.2021.104185

[CR63] Wang X, Wang C, Cui X, Wang L, Liu Z, Xu B, Li H (2018) Molecular mechanism by which *Apis cerana cerana* MKK6 (*AccMKK6*)-mediated MAPK cascades regulate the oxidative stress response. Biosci Rep 38:BSR0181301. 10.1042/BSR20181301 10.1042/BSR20181301 PMC6294647 30442872

[CR64] Wang X, Chang X, Jing Y, Zhao J, Fang Q, Sun M, Zhang Y, Li W, Li Y (2020) Identification and functional prediction of soybean CircRNAs involved in low-temperature responses. J Plant Physiol 250:153188. 10.1016/j.jplph.2020.153188 32450394 10.1016/j.jplph.2020.153188

[CR65] Wang Y, Xiong Z, Li Q, Sun Y, Jin J, Chen H, Zou Y, Huang X, Ding Y (2019) Circular RNA profiling of the rice photo-thermosensitive genic male sterile line Wuxiang S reveals circRNA involved in the fertility transition. BMC Plant Biol 19:340. 10.1186/s12870-019-1944-2 31382873 10.1186/s12870-019-1944-2 PMC6683460

[CR66] Wang Z, Zhang Y, Dai K, Liang Z, Zhu M, Zhang M, Pan J, Hu X, Zhang X, Xue R, Cao G, Gong C (2020b) circEgg regulates histone H3K9me3 by sponging bmo-miR-3391-5p and encod-ing circEgg-P122 protein in the silkworm *Bombyx mori*. Insect Biochem Mol Biol 124:103430. 10.1016/j.ibmb.2020.103430 32585305 10.1016/j.ibmb.2020.103430

[CR67] Weigelt CM, Sehgal R, Tain LS, Cheng J, Eßer J, Pahl A, Dieterich C, Grönke S, Partridge L (2020) An insulin-sensitive circular RNA that regulates lifespan in *Drosophila*. Mol Cell 79:268-279.e5. 10.1016/j.molcel.2020.06.011 32592682 10.1016/j.molcel.2020.06.011 PMC7318944

[CR68] Wu Y, Guo Y, Fan X, Zhao H, Zhang Y, Guo S, Jing X, Liu Z, Zou P, Li Q, Na Z, Zhang K, Chen D, Guo R (2023) ame-miR-34 modulates the larval body weight and immune response of *Apis mellifera* workers to *Ascosphara apis* invasion. Int J Mol Sci 24:1214. 10.3390/ijms24021214 36674732 10.3390/ijms24021214 PMC9863880

[CR69] Wu S, Ling EJ (2009) Phagocytosis, nodulation and encapsulation in cellular immune responses in insects. Acta Entomol Sin 52:791–798 (**(In Chinese)**)

[CR70] Xiong C, Chen H, Chen D, Zheng Y, Fu Z, Xu G, Du Y, Wang H, Geng S, Zhou D, Liu Y, Guo R (2018) Analysis of circular RNAs and their regulatory networks in the midgut of *Apis mellifera ligustica* workers. Acta Entomol Sinica 61:1363–1375 (**(In Chinese)**)

[CR71] Xiong C, Du Y, Wang H, Zheng Y, Fu Z, Wang H, Zhang L, Chen D, Guo R (2019) Unraveling the mechanism regulating the *Ascosphaera apis*-resistance difference between *Apis cerana cerana* and *Apis mellifera ligustica* larvae based on comparative transcriptome analysis. J China Agric Univ 24:106–114 (**(In Chinese)**)

[CR72] Yang Y, Gao X, Zhang M, Yan S, Sun C, Xiao F, Huang N, Yang X, Zhao K, Zhou H, Huang S, Xie B, Zhang N (2018) Novel role of FBXW7 circular RNA in repressing glioma tumorigenesis. J Natl Cancer Inst 110:304–15. 10.1093/jnci/djx166 28903484 10.1093/jnci/djx166 PMC6019044

[CR73] Ye Y, Fan X, Cai Z, Wu Y, Zhang W, Zhao H, Guo S, Feng P, Li Q, Zou P, Chen M, Fan N, Chen D, Guo R (2022) Unveiling the circRNA-mediated immune responses of Western honey bee larvae to *Ascosphaera apis* invasion. Int J Mol Sci 24:613. 10.3390/ijms24010613 36614055 10.3390/ijms24010613 PMC9820429

[CR74] Ye Y, Wang J, Zhang J, Zhang K, Gu X, Yao Y, Ren Z, Zhang Y, Chen D, Guo R (2023) Circular RNA ame_circ_000115 regulates expression of genes in larval gusts of *Apis mellifera ligustica* stressed by *Ascosphaera apis*. Chin J Biotechnol 39:217–230 (**(In Chinese)**) 10.13345/j.cjb.220459 36738212

[CR75] Yuan J, Wang Z, Xing J, Yang Q, Chen XL (2018) Genome-wide Identification and characterization of circular RNAs in the rice blast fungus *Magnaporthe oryzae*. Sci Rep 8:6757. 10.1038/s41598-018-25242-w 29713025 10.1038/s41598-018-25242-w PMC5928111

[CR76] Zhai Z, Huang X, Yin Y (2018) Beyond immunity: the Imd pathway as a coordinator of host defense organismal physiology and behavior. Dev Comp Immunol 83:51–59. 10.1016/j.dci.2017.11.008 29146454 10.1016/j.dci.2017.11.008

[CR77] Zhang C, Teng B, Liu H, Wu C, Wang L, Jin S (2023) Impact of *Beauveria bassiana* on antioxidant enzyme activities and metabolomic profiles of *Spodoptera frugiperda*. J Invertebr Pathol 198:107929. 10.1016/j.jip.2023.107929 37127135 10.1016/j.jip.2023.107929

[CR78] Zhang J, Liu R, Zhu Y, Gong J, Yin S, Sun P, Feng H, Wang Q, Zhao S, Wang Z, Li G (2020) Identification and characterization of circRNAs responsive to methyl jasmonate in *Arabidopsis thaliana*. Int J Mol Sci 21:792. 10.3390/ijms21030792 31991793 10.3390/ijms21030792 PMC7037704

[CR79] Zhang J, Wang H, Wu W, Dong Y, Wang M, Yi D, Zhou Y, Xu Q (2020) Systematic identification and functional analysis of circular RNAs during rice black-streaked dwarf virus infection in the *Laodelphax striatellus* (Fallén) midgut. Front Microbiol 11:588009. 10.3389/fmicb.2020.588009 33117326 10.3389/fmicb.2020.588009 PMC7550742

[CR80] Zhang J, Hou L, Zuo Z, Ji P, Zhang X, Xue Y, Zhao F (2021) Comprehensive profiling of circular RNAs with nanopore sequencing and CIRI-long. Nat Biotechnol 39:836–845. 10.1038/s41587-021-00842-6 33707777 10.1038/s41587-021-00842-6

[CR81] Zhang K, Fu Z, Fan X, Wang Z, Wang S, Guo S, Gao X, Zhao H, Jing X, Zou P, Li Q, Chen D, Guo R (2023) Effect of *Ascosphaera apis* infestation on the activities of four antioxidant enzymes in Asian honey bee larval guts. Antioxidants (Basel) 12:206. 10.3390/antiox12010206 36671067 10.3390/antiox12010206 PMC9854781

[CR82] Zhang P, Yang B, Dai XJ, Pang Y, Zhong J, Su DM (2002) Apoptosis of *Spodoptera litura* cells induced by AcMNPV *ie*^-1^gene. ABBS 06:707–711 (**(In Chinese)**) 12417911

[CR83] Zhang W, Qin P, Gong X, Huang L, Wang C, Chen G, Chen J, Wang L, Lv Z (2020) Identification of circRNAs in the liver of whitespotted bamboo shark (*Chiloscyllium plagiosum*). Front Genet 11:596308. 10.3389/fgene.2020.596308 33362857 10.3389/fgene.2020.596308 PMC7759564

[CR84] Zhang Y, Zhang X, Dai K, Zhu M, Liang Z, Pan J, Zhang Z, Xue R, Cao G, Hu X, Gong C (2022) *Bombyx mori* Akirin hijacks a viral peptide vSP27 encoded by BmCPV circRNA and activates the ROS-NF-κB pathway against viral infection. Int J Biol Macromol 194:223–232. 10.1016/j.ijbiomac.2021.11.201 34875309 10.1016/j.ijbiomac.2021.11.201

[CR85] Zhao J, Wu J, Xu T, Yang Q, He J, Song X (2018) IRESfinder: identifying RNA internal ribosome entry site in eukaryotic cell using framed *k*-mer features. J Genet Genomics 45:403–406. 10.1016/j.jgg.2018.07.006 30054216 10.1016/j.jgg.2018.07.006

[CR86] Zhao W, Cheng Y, Zhang C, You Q, Shen X, Guo W, Jiao Y (2017) Genome-wide identification and characterization of circular RNAs by high throughput sequencing in soybean. Sci Rep 7:5636. 10.1038/s41598-017-05922-9 28717203 10.1038/s41598-017-05922-9 PMC5514102

[CR87] Zhao X, Zhong Y, Wang X, Shen J, An W (2022) Advances in circular RNA and its applications. Int J Med Sci 19:975–985. 10.7150/ijms.71840 35813288 10.7150/ijms.71840 PMC9254372

[CR88] Zhou R, Sanz-Jimenez P, Zhu XT, Feng JW, Shao L, Song JM, Chen LL (2021) Analysis of rice transcriptome reveals the lncRNA/circRNA regulation in tissue development. Rice (N Y) 14:14. 10.1186/s12284-021-00455-2 33507446 10.1186/s12284-021-00455-2 PMC7843763

[CR89] Zhu Z, Wang J, Fan X, Long Q, Chen H, Ye Y, Zhang K, Ren Z, Zhang Y, Niu Q, Chen D, Guo R (2022) CircRNA-regulated immune responses of Asian honey bee workers to microsporidian infection. Front Genet 13:1013239. 10.3389/fgene.2022.1013239 36267412 10.3389/fgene.2022.1013239 PMC9577369

