# Supplementary material for: Dynamics and regulatory role of circRNAs in Asian honey bee larvae following fungal infection
Source: Appl Microbiol Biotechnol. 2024 Mar 13;108:261. doi: 10.1007/s00253-024-13102-9 (PMC10933204; doi:10.1007/s00253-024-13102-9)
Supplement: Supplementary file 1 [file 253_2024_13102_MOESM1_ESM.pdf]

Applied Microbiology and Biotechnology

Dynamics and regulatory role of circRNAs in Asian honey bee larvae following fungal infection

Rui Guo [1,2,3,†,*], Kaiyao Zhang [1,†], He Zang [1], Sijia Guo [1], Xiaoyu Liu1, Xin Jing[1], Yuxuan Song[1], Kunze Li[1], Ying Wu[4], Haibing Jiang[4], Zhongmin Fu[1,2,3], and Dafu Chen [1,2,3]

[1]College of Bee Science and Biomedicine, Fujian Agriculture and Forestry University, Fuzhou 350002, China

[2] National & Local United Engineering Laboratory of Natural Biotoxin, Fuzhou 350002, China

[3]Apiculture Science Institute of Jilin Province, Jilin, Jilin 132000, China

[4]Apitherapy Research Institute of Fujian Province, Fuzhou 350002, China

* Correspondence author: Rui Guo.

E-mail: ruiguo@fafu.edu.cn

Telephone: 15205080780

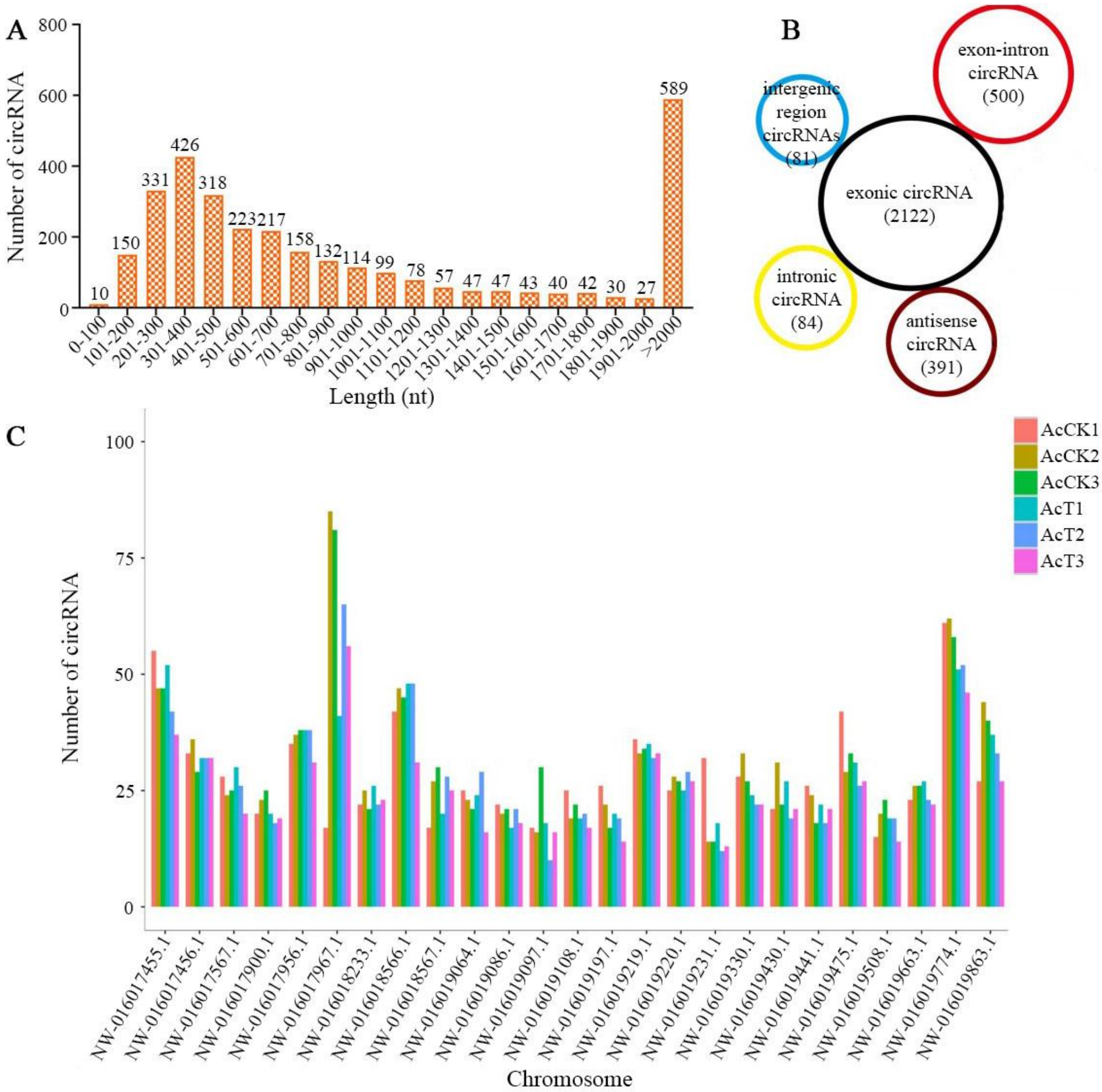

Fig. S1 Characteristics of circRNAs identified in *A. c. cerana*. (**A**) Length distribution; (**B**) types, the numbers of various types of circRNAs are presented in brackets; (**C**) chromosome distribution.

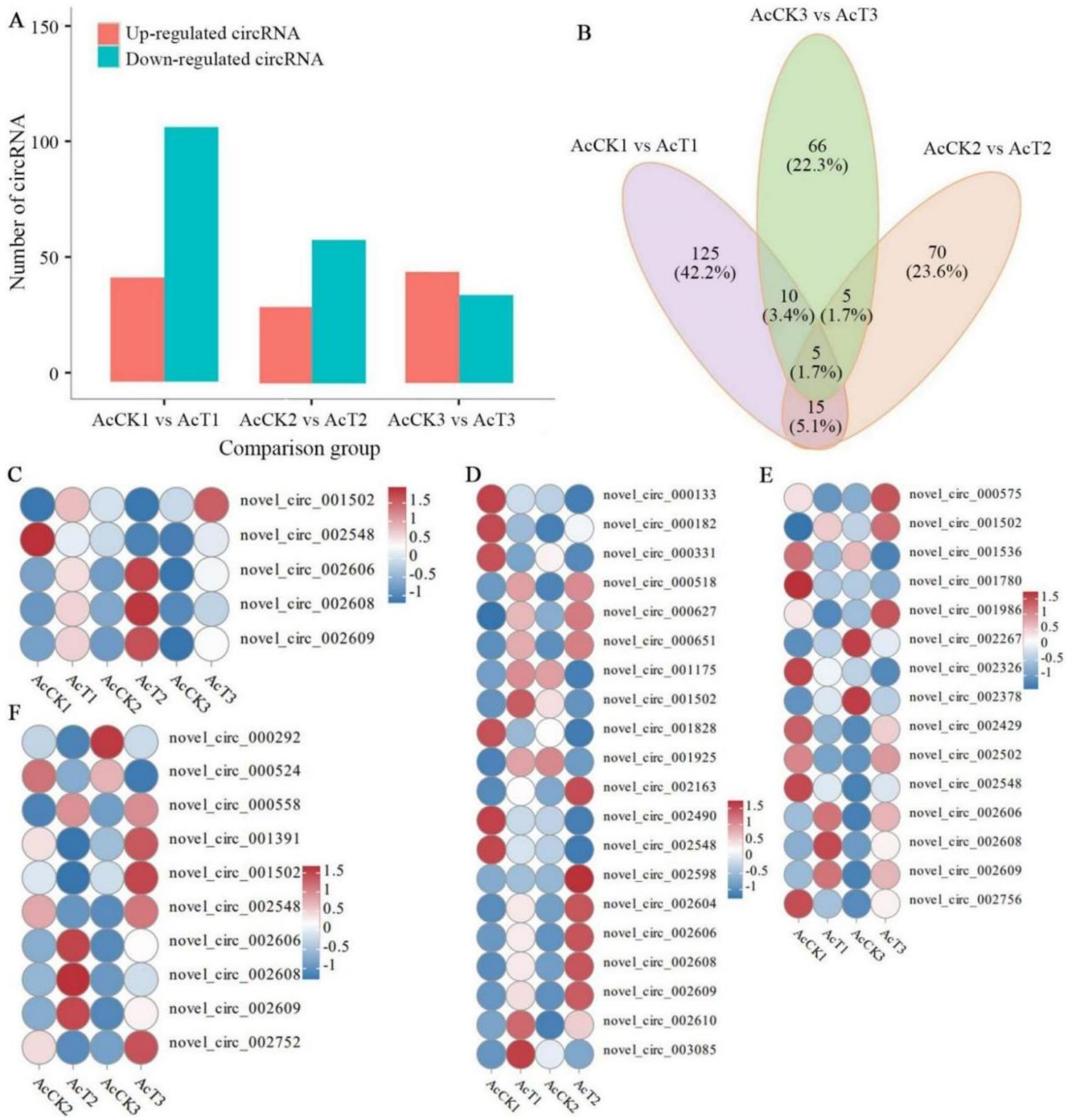

Fig. S2 Expression clustering of DEcircRNAs in three comparison groups. (A) Number statistics of DEcircRNAs in three comparison groups; (B) Venn analysis of DEcircRNAs in three comparison groups; (C) Expression clustering of shared DEcircRNAs by three comparison groups; (D) Expression clustering of shared DEcircRNAs by AcCK1 vs. AcT1 and AcCK2 vs. AcT2 comparison groups; (E) Expression clustering of shared DEcircRNAs by AcCK1 vs. AcT1 and AcCK3 vs. AcT3 comparison groups; (F) Expression clustering of shared DEcircRNAs by AcCK2 vs. AcT2 and AcCK3 vs. AcT3 comparison groups.

Table S1. Detailed information about primers used in this work

Primer	Sequences (5'-3')
novel_circ_000983-F	AGGGACATCGCCCACTCAAT
novel_circ_000983-R	GCCCGCACCACTTCCATCTT
novel_circ_001484-F	CGACTTCCACTCGAGCATCC

novel_circ_001484-R	ACCCCACTTCGCCTATTTACC
novel_circ_002377-F	TGACACAAGCCAGATTGACCG
novel_circ_002377-R	GCTTATCGTGCTACCCTTCTTCA
novel_circ_002486-F	TCAAGGGGTGAACGGCAAGA
novel_circ_002486-R	CGCTCGGCAAAACGGTGAATTTATC
novel_circ_000882-F	ACCACGGGAACCGACGAGACCACAG
novel_circ_000882-R	CCACCGTCCACATTTTT
novel_circ_000504-F	CGAAGATTTTCAAGGGG
novel_circ_000504-R	GACACAACCGAACCGAT
novel_circ_001175-F	GCCACGACAATAAGAAAC
novel_circ_001175-R	GTCAGATGAGCCGAGTTC
novel_circ_002439-F	AGGCATCTTCTTGTCTCG
novel_circ_002439-R	ACCTCTTTCTTGCTGTTC
novel_circ_000405-F	CATTTGTACCACCAATCG
novel_circ_000405-R	GTATCTGTGACATCCGCC
novel_circ_003046-F	ACCCCTGCATTCTTCGT
novel_circ_003046-R	TTTTAGCCCCGTGTTTCT
novel_circ_000526-F	TGTTCATCTTTTTCCCAG
novel_circ_000526-R	TTTCCGCAGTATCGTGTC
novel_circ_002378-F	GCTCTTGGAGATGATTTGGC
novel_circ_002378-R	TTCGTTACATTGATGCTGGTG
novel_circ_001391-F	TAACTGAAACGACCACGAAA
novel_circ_001391-R	ATCTGCTTGTCTATCAGCTGTAC
novel_circ_001799-F	AAAATGGGCACATCGTTGA
novel_circ_001799-R	CGCTCTGCACACCAGAAAT
novel_circ_000102-F	TGGATACGGTGTATTCAGCA
novel_circ_000102-R	GAAGCCAGCCCCTCTAAG
actin-F	TTATATGCCAACACTGTCCTTT
actin-R	AGAATTGATCCACCAATCCA

Table S2. Summary of data quality control

Sample	raw reads	clean reads	Q30	CG content
AcCK1	73830148	73775592	94.65%	47.22%
AcCK2	96586212	96513798	94.21%	46.72%
AcCK3	94552744	94495000	94.89%	49.75%
AcT1	76672564	76593924	93.42%	47.32%

AcT2	90954858	90870608	94.35%	48.50%
AcT3	83418832	83339288	94.64%	51.75%

Table S3. CircRNAs identified in the guts of *A. c. cerana* worker larvae

CircRNA ID	Source gene ID	Chromosome	Strand	Genomic start	Genomic end	Length	Type
novel_circ_000001	NA	NC_014295.1	+	11264	11419	157	Intergenic region
novel_circ_000002	LOC108004004	NW_016017455.1	-	13770	24518	793	Exonic
novel_circ_000003	LOC108002673	NW_016017455.1	-	394477	404435	1470	Exonic
novel_circ_000004	LOC108002673	NW_016017455.1	-	399892	404435	1414	Exonic
novel_circ_000005	LOC108002673	NW_016017455.1	-	402863	404435	1069	Exonic
novel_circ_000006	LOC107997942	NW_016017455.1	+	516715	517332	618	Antisense
novel_circ_000007	LOC108004229	NW_016017455.1	-	566978	567225	248	Exonic
novel_circ_000008	LOC108004253	NW_016017455.1	-	677375	684928	638	Exonic
novel_circ_000009	LOC107993360	NW_016017455.1	-	759482	769881	9808	Exon–intron
novel_circ_000010	LOC107993141	NW_016017455.1	-	798320	801842	1960	Exonic
novel_circ_000011	LOC108002397	NW_016017455.1	-	858002	864529	5894	Exon–intron
novel_circ_000012	LOC108002084	NW_016017455.1	-	884856	886844	673	Exonic
novel_circ_000013	LOC108002084	NW_016017455.1	-	884866	886844	663	Exonic
novel_circ_000014	LOC108002084	NW_016017455.1	-	885677	886844	518	Exonic
novel_circ_000015	LOC108002084	NW_016017455.1	-	893544	895102	386	Exonic
novel_circ_000016	LOC107993886	NW_016017455.1	-	944292	945541	284	Exonic
novel_circ_000017	LOC107993886	NW_016017455.1	-	944292	948558	413	Exonic
novel_circ_000018	LOC108003743	NW_016017455.1	+	1156706	1157381	294	Exonic
novel_circ_000019	LOC108003743	NW_016017455.1	+	1206597	1206731	135	Exonic
novel_circ_000020	LOC108003867	NW_016017455.1	+	1246800	1247510	133	Exonic
novel_circ_000021	LOC108003844	NW_016017455.1	+	1283704	1290380	4677	Exonic
novel_circ_000022	LOC108003884	NW_016017455.1	-	1288134	1332118	43985	Exon–intron
novel_circ_000023	LOC108003844	NW_016017455.1	+	1322428	1322722	215	Exon–intron
novel_circ_000024	LOC107992566	NW_016017455.1	+	1400416	1400975	560	Antisense
novel_circ_000025	LOC107992644	NW_016017455.1	-	1551571	1552234	417	Exonic
novel_circ_000026	LOC107992644	NW_016017455.1	-	1551844	1555302	1032	Exonic
novel_circ_000027	LOC107992415	NW_016017455.1	+	1587799	1588551	559	Exonic
novel_circ_000028	LOC107992415	NW_016017455.1	-	1589859	1590251	393	Antisense
novel_circ_000029	LOC107992415	NW_016017455.1	+	1589960	1590251	292	Exonic
novel_circ_000030	LOC107993282	NW_016017455.1	+	1595838	1596593	685	Exonic
novel_circ_000031	LOC108000325	NW_016017455.1	+	1690534	1697728	425	Exonic
novel_circ_000032	LOC107993624	NW_016017455.1	+	1742711	1756816	1637	Exonic
novel_circ_000033	LOC107993624	NW_016017455.1	+	1756291	1756816	305	Exonic
novel_circ_000034	LOC108001815	NW_016017455.1	+	1909994	1911613	981	Exonic
novel_circ_000035	LOC107993194	NW_016017455.1	+	1986349	1987431	584	Exonic
novel_circ_000036	LOC108004510	NW_016017455.1	+	2108769	2123514	1122	Exonic
novel_circ_000037	LOC108004510	NW_016017455.1	+	2122472	2123514	773	Exonic
novel_circ_000038	LOC107992400	NW_016017455.1	+	2203887	2300530	96007	Exon–intron
novel_circ_000039	LOC108004515	NW_016017455.1	+	2298875	2300530	1552	Exon–intron
novel_circ_000040	LOC108003823	NW_016017455.1	+	2357014	2358076	700	Exonic
novel_circ_000041	LOC108003778	NW_016017455.1	-	2363697	2373068	925	Exonic
novel_circ_000042	LOC108003778	NW_016017455.1	-	2363697	2393551	1330	Exonic
novel_circ_000043	LOC108003778	NW_016017455.1	-	2372297	2373068	292	Exonic

novel_circ_000044	LOC108003778	NW_016017455.1	-	2372297	2393551	697	Exonic
novel_circ_000045	LOC108003778	NW_016017455.1	-	2393147	2393551	405	Exonic
novel_circ_000046	NA	NW_016017455.1	+	2548846	2552074	3229	Intergenic region
novel_circ_000047	LOC108003653	NW_016017455.1	-	2556401	2557166	576	Exonic
novel_circ_000048	LOC108003653	NW_016017455.1	-	2556761	2560465	967	Exonic
novel_circ_000049	LOC108003653	NW_016017455.1	-	2557470	2557971	418	Exonic
novel_circ_000050	LOC108003653	NW_016017455.1	-	2583353	2584739	1387	Exon–intron
novel_circ_000051	LOC108003695	NW_016017455.1	+	2661045	2670682	9161	Exon–intron
novel_circ_000052	LOC107992589	NW_016017455.1	+	2705332	2705497	166	Exonic
novel_circ_000053	LOC107992589	NW_016017455.1	+	2705804	2706155	352	Exon–intron
novel_circ_000054	LOC107992722	NW_016017455.1	+	2734687	2736277	1591	Exon–intron
novel_circ_000055	LOC107993535	NW_016017455.1	-	2767843	2768979	1137	Exonic
novel_circ_000056	LOC107993258	NW_016017455.1	-	2907552	2909073	298	Exonic
novel_circ_000057	LOC107992698	NW_016017455.1	+	3018616	3059577	431	Exonic
novel_circ_000058	LOC107992698	NW_016017455.1	+	3079554	3080633	1080	Exonic
novel_circ_000059	LOC107993015	NW_016017455.1	-	3155030	3157901	2043	Exon–intron
novel_circ_000060	LOC108003935	NW_016017455.1	+	3416415	3417623	858	Exon–intron
novel_circ_000061	LOC108003935	NW_016017455.1	+	3416415	3417947	1082	Exon–intron
novel_circ_000062	LOC108003935	NW_016017455.1	+	3416415	3418625	1346	Exon–intron
novel_circ_000063	LOC108003935	NW_016017455.1	+	3417253	3418625	859	Exonic
novel_circ_000064	LOC108003935	NW_016017455.1	+	3417733	3418352	356	Exonic
novel_circ_000065	LOC108003935	NW_016017455.1	+	3418212	3418625	264	Exonic
novel_circ_000066	LOC108003935	NW_016017455.1	+	3424192	3424731	370	Exonic
novel_circ_000067	LOC107992878	NW_016017455.1	+	3516725	3517300	576	Antisense
novel_circ_000068	LOC108004465	NW_016017455.1	+	3541986	3545187	247	Exonic
novel_circ_000069	LOC108004465	NW_016017455.1	+	3541986	3567051	1758	Exonic
novel_circ_000070	LOC108004465	NW_016017455.1	+	3545091	3567051	1608	Exonic
novel_circ_000071	LOC108004465	NW_016017455.1	+	3555286	3567051	1266	Exonic
novel_circ_000072	LOC108004465	NW_016017455.1	+	3565988	3567051	336	Exonic
novel_circ_000073	LOC108004465	NW_016017455.1	+	3568832	3569985	654	Exonic
novel_circ_000074	LOC107998723	NW_016017455.1	+	3702661	3703755	235	Exonic
novel_circ_000075	LOC107998723	NW_016017455.1	+	3738622	3740224	863	Exonic
novel_circ_000076	LOC108004420	NW_016017455.1	-	3792089	3793672	881	Exonic
novel_circ_000077	LOC108004171	NW_016017455.1	-	3828863	3829218	265	Exonic
novel_circ_000078	LOC107992842	NW_016017455.1	-	4693097	4694873	1648	Exon–intron
novel_circ_000079	LOC107993217	NW_016017455.1	-	5680750	5682072	1323	Exon–intron
novel_circ_000080	LOC107993510	NW_016017455.1	-	5744249	5745418	1170	Exonic
novel_circ_000081	LOC107993510	NW_016017455.1	-	5744258	5745418	1161	Exonic
novel_circ_000082	LOC107993510	NW_016017455.1	-	5744305	5745418	1114	Exonic
novel_circ_000083	LOC107993510	NW_016017455.1	-	5746461	5747342	882	Exonic
novel_circ_000084	LOC107992863	NW_016017455.1	-	5752392	5758850	5589	Exon–intron
novel_circ_000085	LOC107992863	NW_016017455.1	-	5755098	5756704	870	Exonic
novel_circ_000086	LOC107993107	NW_016017455.1	-	5840784	5842477	1694	Exonic
novel_circ_000087	LOC108004445	NW_016017455.1	-	6163196	6164524	488	Exonic
novel_circ_000088	LOC108004445	NW_016017455.1	-	6163216	6262481	605	Exonic
novel_circ_000089	LOC107994273	NW_016017456.1	-	173402	174072	503	Exonic
novel_circ_000090	LOC114577180	NW_016017456.1	+	198675	200971	2297	Exon–intron
novel_circ_000091	LOC114577180	NW_016017456.1	+	198678	200971	2294	Exon–intron
novel_circ_000092	LOC107993932	NW_016017456.1	+	199567	200971	601	Exonic

novel_circ_000093	NA	NW_016017456.1	-	407476	417110	9635	Intergenic region
novel_circ_000094	LOC107995095	NW_016017456.1	+	525208	541174	15139	Exon–intron
novel_circ_000095	LOC107995399	NW_016017456.1	-	791219	794082	2640	Exon–intron
novel_circ_000096	LOC107995399	NW_016017456.1	-	791219	812320	10436	Exon–intron
novel_circ_000097	LOC107995399	NW_016017456.1	-	804525	812320	7796	Exon–intron
novel_circ_000098	LOC107995399	NW_016017456.1	-	811986	814776	2791	Exon–intron
novel_circ_000099	LOC107996366	NW_016017456.1	-	880629	882031	1037	Exonic
novel_circ_000100	LOC107995988	NW_016017456.1	+	1182680	1183016	337	Intronic
novel_circ_000101	LOC107995988	NW_016017456.1	+	1182680	1183034	355	Intronic
novel_circ_000102	LOC107995988	NW_016017456.1	+	1404383	1406972	350	Exonic
novel_circ_000103	LOC107995660	NW_016017456.1	+	1766565	1780639	10712	Exon–intron
novel_circ_000104	LOC107995660	NW_016017456.1	+	1766600	1780639	10677	Exon–intron
novel_circ_000105	LOC107995660	NW_016017456.1	+	1774397	1780639	2880	Exon–intron
novel_circ_000106	LOC107995660	NW_016017456.1	+	1774430	1780639	2847	Exon–intron
novel_circ_000107	LOC107995660	NW_016017456.1	+	1774457	1780639	2820	Exon–intron
novel_circ_000108	LOC107995660	NW_016017456.1	+	1775310	1780639	1967	Exon–intron
novel_circ_000109	LOC107995660	NW_016017456.1	+	1792988	1797094	2066	Exon–intron
novel_circ_000110	LOC107995981	NW_016017456.1	+	1847886	1850131	300	Exonic
novel_circ_000111	LOC107995475	NW_016017456.1	+	2030148	2032029	1082	Exonic
novel_circ_000112	LOC107995475	NW_016017456.1	+	2030148	2034449	3097	Exonic
novel_circ_000113	LOC107995475	NW_016017456.1	+	2030788	2040138	5317	Exonic
novel_circ_000114	LOC107995475	NW_016017456.1	+	2031624	2034449	2273	Exonic
novel_circ_000115	LOC107995475	NW_016017456.1	+	2031889	2040138	4665	Exonic
novel_circ_000116	LOC107994557	NW_016017456.1	-	2533536	2535598	2063	Exonic
novel_circ_000117	LOC107995206	NW_016017456.1	-	2686979	2708335	336	Exonic
novel_circ_000118	LOC107994952	NW_016017456.1	+	2997239	2999850	2533	Exonic
novel_circ_000119	LOC107994952	NW_016017456.1	+	2997239	3000179	2620	Exonic
novel_circ_000120	LOC107994952	NW_016017456.1	+	3000583	3003438	1550	Exonic
novel_circ_000121	LOC107994952	NW_016017456.1	+	3000583	3005861	3255	Exonic
novel_circ_000122	LOC107994918	NW_016017456.1	-	3074764	3079090	2120	Exon–intron
novel_circ_000123	LOC107994918	NW_016017456.1	-	3077027	3079090	726	Exonic
novel_circ_000124	LOC107994918	NW_016017456.1	-	3077854	3079090	588	Exonic
novel_circ_000125	LOC107994918	NW_016017456.1	-	3077854	3092140	779	Exonic
novel_circ_000126	LOC107994918	NW_016017456.1	-	3078512	3079090	392	Exonic
novel_circ_000127	LOC107994925	NW_016017456.1	+	3089801	3090325	441	Exonic
novel_circ_000128	LOC107995000	NW_016017456.1	+	3124041	3124772	470	Exonic
novel_circ_000129	LOC107994102	NW_016017456.1	-	3156540	3157108	443	Exonic
novel_circ_000130	LOC114577081	NW_016017456.1	+	3178846	3179009	164	Exonic
novel_circ_000131	LOC107995300	NW_016017456.1	-	3421456	3421934	386	Exonic
novel_circ_000132	LOC107995253	NW_016017456.1	+	3426392	3427473	674	Exonic
novel_circ_000133	LOC107994792	NW_016017456.1	+	3482166	3485591	1915	Exonic
novel_circ_000134	LOC107994792	NW_016017456.1	+	3484999	3485591	304	Exonic
novel_circ_000135	LOC107996955	NW_016017457.1	-	174760	175428	669	Intronic
novel_circ_000136	LOC107996772	NW_016017457.1	+	273771	277189	2043	Exonic
novel_circ_000137	LOC107996772	NW_016017457.1	+	273771	277247	2101	Exonic
novel_circ_000138	LOC107996852	NW_016017457.1	-	425925	436055	7635	Exon–intron
novel_circ_000139	LOC107996802	NW_016017457.1	+	514662	515276	319	Exonic
novel_circ_000140	LOC107996802	NW_016017457.1	+	514662	518489	478	Exonic
novel_circ_000141	LOC107996503	NW_016017457.1	-	610952	611117	166	Antisense

novel_circ_000142	LOC107996503	NW_016017457.1	+	610984	611228	245	Exonic
novel_circ_000143	LOC107996503	NW_016017457.1	+	610990	611856	792	Exonic
novel_circ_000144	LOC107996503	NW_016017457.1	+	610993	611799	732	Exonic
novel_circ_000145	LOC107996503	NW_016017457.1	+	611002	611849	773	Exonic
novel_circ_000146	LOC107996503	NW_016017457.1	+	611005	611208	204	Exonic
novel_circ_000147	LOC107996503	NW_016017457.1	+	611014	611805	717	Exonic
novel_circ_000148	LOC107996503	NW_016017457.1	+	611026	611228	203	Exonic
novel_circ_000149	LOC107996503	NW_016017457.1	+	611182	611490	234	Exonic
novel_circ_000150	LOC107996503	NW_016017457.1	+	611182	611822	566	Exonic
novel_circ_000151	LOC107996503	NW_016017457.1	-	611362	611765	404	Antisense
novel_circ_000152	LOC107996503	NW_016017457.1	+	611421	611777	357	Exonic
novel_circ_000153	LOC107996503	NW_016017457.1	-	611491	611770	280	Antisense
novel_circ_000154	LOC107996503	NW_016017457.1	-	611494	611829	336	Antisense
novel_circ_000155	LOC107997214	NW_016017457.1	-	622645	622824	180	Antisense
novel_circ_000156	LOC107997624	NW_016017468.1	+	150518	159276	8174	Exon–intron
novel_circ_000157	LOC107997624	NW_016017468.1	+	150862	159276	7830	Exon–intron
novel_circ_000158	LOC107997664	NW_016017468.1	-	210295	210776	334	Exonic
novel_circ_000159	LOC107997844	NW_016017468.1	+	239916	241166	1251	Antisense
novel_circ_000160	LOC107997554	NW_016017468.1	+	244585	248204	2761	Exon–intron
novel_circ_000161	LOC107997758	NW_016017468.1	+	573297	594479	496	Exonic
novel_circ_000162	LOC107999008	NW_016017479.1	-	105216	107882	1695	Exon–intron
novel_circ_000163	LOC107998949	NW_016017479.1	-	161677	162705	828	Exonic
novel_circ_000164	LOC107998324	NW_016017479.1	+	178529	212564	32063	Exon–intron
novel_circ_000165	LOC114577653	NW_016017479.1	+	178782	193672	8924	Exon–intron
novel_circ_000166	LOC114577653	NW_016017479.1	+	178782	193981	9233	Exon–intron
novel_circ_000167	LOC114577653	NW_016017479.1	+	178782	195293	10545	Exon–intron
novel_circ_000168	LOC114577653	NW_016017479.1	+	178782	197526	12778	Exon–intron
novel_circ_000169	LOC107998324	NW_016017479.1	+	178782	205635	24881	Exon–intron
novel_circ_000170	LOC107998324	NW_016017479.1	+	178782	206334	25580	Exon–intron
novel_circ_000171	LOC107998324	NW_016017479.1	+	178782	212564	31810	Exon–intron
novel_circ_000172	LOC107998344	NW_016017479.1	-	185217	185803	509	Exonic
novel_circ_000173	LOC107998344	NW_016017479.1	-	185305	185699	317	Exonic
novel_circ_000174	LOC107998344	NW_016017479.1	-	185370	185814	367	Exonic
novel_circ_000175	LOC114577653	NW_016017479.1	+	193475	195293	1819	Exon–intron
novel_circ_000176	LOC114577653	NW_016017479.1	+	194502	195293	792	Exon–intron
novel_circ_000177	LOC114577653	NW_016017479.1	+	195048	195293	246	Exon–intron
novel_circ_000178	LOC107998324	NW_016017479.1	+	195048	212564	15544	Exon–intron
novel_circ_000179	LOC107998324	NW_016017479.1	-	196418	196797	380	Antisense
novel_circ_000180	LOC107998324	NW_016017479.1	+	199161	199703	543	Exonic
novel_circ_000181	LOC107999178	NW_016017479.1	-	350194	350542	245	Exonic
novel_circ_000182	LOC107999178	NW_016017479.1	-	358910	359324	415	Exonic
novel_circ_000183	LOC107998389	NW_016017479.1	-	416705	419147	2334	Exonic
novel_circ_000184	LOC107998803	NW_016017479.1	-	545806	547432	1627	Antisense
novel_circ_000185	LOC107998870	NW_016017479.1	-	625530	632127	1402	Exonic
novel_circ_000186	LOC107998870	NW_016017479.1	-	628431	628957	406	Exonic
novel_circ_000187	LOC114577727	NW_016017490.1	+	387469	394777	6680	Exon–intron
novel_circ_000188	LOC107999718	NW_016017501.1	-	25381	26776	690	Exonic
novel_circ_000189	LOC107999718	NW_016017501.1	-	25381	31815	861	Exonic
novel_circ_000190	LOC107999718	NW_016017501.1	-	26028	26776	424	Exonic

novel_circ_000191	LOC107999718	NW_016017501.1	-	28314	31815	3502	Exon–intron
novel_circ_000192	LOC107999718	NW_016017501.1	+	131325	131869	545	Antisense
novel_circ_000193	LOC107999767	NW_016017501.1	-	183522	185349	1348	Exonic
novel_circ_000194	LOC107999907	NW_016017501.1	-	272031	272580	380	Exonic
novel_circ_000195	LOC107999907	NW_016017501.1	-	288820	289974	1155	Exon–intron
novel_circ_000196	LOC108001185	NW_016017512.1	+	13728	15797	456	Exonic
novel_circ_000197	LOC108000602	NW_016017512.1	-	216873	217310	438	Antisense
novel_circ_000198	LOC108000548	NW_016017512.1	-	233853	237376	245	Exonic
novel_circ_000199	LOC108001166	NW_016017512.1	-	258772	259528	406	Exonic
novel_circ_000200	LOC108000759	NW_016017512.1	-	285408	286066	455	Exonic
novel_circ_000201	LOC108000771	NW_016017512.1	-	318746	318989	244	Antisense
novel_circ_000202	LOC108001007	NW_016017512.1	-	405154	406098	510	Exonic
novel_circ_000203	LOC108001007	NW_016017512.1	-	405154	410259	663	Exonic
novel_circ_000204	LOC108001007	NW_016017512.1	-	405154	415067	5471	Exon–intron
novel_circ_000205	LOC114577824	NW_016017512.1	-	439899	440067	169	Antisense
novel_circ_000206	LOC114577824	NW_016017512.1	+	440111	440305	195	Exonic
novel_circ_000207	LOC114577824	NW_016017512.1	-	440281	440444	164	Antisense
novel_circ_000208	LOC114577824	NW_016017512.1	+	440327	440613	287	Exon–intron
novel_circ_000209	LOC114577824	NW_016017512.1	+	440551	440710	160	Exon–intron
novel_circ_000210	LOC114577824	NW_016017512.1	+	444101	444281	181	Exonic
novel_circ_000211	LOC108000316	NW_016017512.1	-	474340	475796	1206	Exonic
novel_circ_000212	LOC108001345	NW_016017523.1	-	401248	403338	543	Exonic
novel_circ_000213	LOC108001345	NW_016017523.1	-	401248	424408	771	Exonic
novel_circ_000214	LOC108001840	NW_016017534.1	+	8125	9435	402	Exonic
novel_circ_000215	LOC108001967	NW_016017534.1	+	212149	212368	220	Exonic
novel_circ_000216	LOC108001967	NW_016017534.1	+	226116	226506	391	Exonic
novel_circ_000217	LOC108001967	NW_016017534.1	-	226128	226471	344	Antisense
novel_circ_000218	LOC108001967	NW_016017534.1	+	226413	228015	1603	Exonic
novel_circ_000219	LOC108001967	NW_016017534.1	+	227331	227556	226	Exonic
novel_circ_000220	LOC108001967	NW_016017534.1	-	228163	228439	277	Antisense
novel_circ_000221	LOC108002168	NW_016017534.1	+	265053	265692	640	Antisense
novel_circ_000222	NA	NW_016017534.1	-	418771	419063	293	Intergenic region
novel_circ_000223	LOC108001865	NW_016017534.1	-	457556	480808	4415	Exon–intron
novel_circ_000224	LOC108001865	NW_016017534.1	-	480659	482825	312	Exonic
novel_circ_000225	LOC108001865	NW_016017534.1	-	480659	483319	477	Exonic
novel_circ_000226	LOC108001865	NW_016017534.1	-	482664	483319	327	Exonic
novel_circ_000227	LOC108001865	NW_016017534.1	-	489532	490063	321	Exonic
novel_circ_000228	LOC108002506	NW_016017545.1	+	129789	180256	662	Exonic
novel_circ_000229	LOC108002506	NW_016017545.1	+	175811	176194	384	Exonic
novel_circ_000230	LOC108002506	NW_016017545.1	+	203702	205473	377	Exonic
novel_circ_000231	LOC108002637	NW_016017545.1	+	274989	275394	406	Intronic
novel_circ_000232	LOC108002549	NW_016017545.1	-	532724	544943	314	Exonic
novel_circ_000233	LOC108002549	NW_016017545.1	-	532724	571767	448	Exonic
novel_circ_000234	LOC108003352	NW_016017556.1	+	43160	43573	414	Exonic
novel_circ_000235	LOC108003352	NW_016017556.1	-	43251	43498	248	Antisense
novel_circ_000236	LOC108002838	NW_016017556.1	+	156403	167478	10463	Exon–intron
novel_circ_000237	LOC108003097	NW_016017556.1	-	195008	195250	243	Antisense
novel_circ_000238	LOC108003324	NW_016017556.1	-	455222	458301	503	Exonic
novel_circ_000239	LOC108003306	NW_016017556.1	+	587476	588405	510	Exonic

novel_circ_000240	LOC108003306	NW_016017556.1	-	591638	593687	2050	Antisense
novel_circ_000241	LOC108003564	NW_016017567.1	-	192341	195261	837	Exonic
novel_circ_000242	LOC108003564	NW_016017567.1	-	192752	195261	636	Exonic
novel_circ_000243	LOC108003564	NW_016017567.1	-	199305	200160	696	Exonic
novel_circ_000244	LOC108003603	NW_016017567.1	-	525349	525569	221	Intronic
novel_circ_000245	LOC108003603	NW_016017567.1	-	525349	527050	1702	Exon–intron
novel_circ_000246	LOC108003627	NW_016017567.1	+	822300	823496	497	Exonic
novel_circ_000247	LOC108003627	NW_016017567.1	+	823137	823496	360	Exonic
novel_circ_000248	LOC108003572	NW_016017567.1	-	885529	886681	290	Exonic
novel_circ_000249	LOC114578224	NW_016017567.1	+	935520	936170	579	Exonic
novel_circ_000250	LOC114578224	NW_016017567.1	+	935520	944910	757	Exonic
novel_circ_000251	LOC108003471	NW_016017567.1	+	1244278	1249042	2466	Exonic
novel_circ_000252	LOC108003540	NW_016017567.1	+	1445413	1446136	473	Exonic
novel_circ_000253	LOC108003569	NW_016017567.1	-	1581187	1581525	339	Exonic
novel_circ_000254	LOC108003569	NW_016017567.1	-	1581187	1583863	837	Exonic
novel_circ_000255	LOC108003569	NW_016017567.1	-	1581187	1587589	1635	Exonic
novel_circ_000256	LOC108003569	NW_016017567.1	-	1581187	1593397	1863	Exonic
novel_circ_000257	LOC108003569	NW_016017567.1	-	1582831	1593397	1524	Exonic
novel_circ_000258	LOC108003569	NW_016017567.1	-	1586930	1587589	393	Exonic
novel_circ_000259	LOC108003569	NW_016017567.1	-	1586930	1593397	621	Exonic
novel_circ_000260	LOC108003569	NW_016017567.1	-	1635780	1637073	1294	Exonic
novel_circ_000261	LOC108003566	NW_016017567.1	+	1750222	1751487	1266	Antisense
novel_circ_000262	LOC108003566	NW_016017567.1	-	1751238	1754927	1757	Exonic
novel_circ_000263	LOC108003566	NW_016017567.1	-	1751293	1752152	608	Exonic
novel_circ_000264	LOC108003566	NW_016017567.1	+	1751832	1752146	315	Antisense
novel_circ_000265	LOC108003566	NW_016017567.1	+	1751882	1754451	2570	Antisense
novel_circ_000266	LOC108003566	NW_016017567.1	+	1751930	1754366	2437	Antisense
novel_circ_000267	LOC108003566	NW_016017567.1	-	1754597	1759012	389	Exonic
novel_circ_000268	LOC108003566	NW_016017567.1	+	1754655	1763711	9057	Antisense
novel_circ_000269	LOC108003566	NW_016017567.1	-	1754805	1759149	318	Exonic
novel_circ_000270	LOC108003566	NW_016017567.1	-	1754821	1759083	236	Exonic
novel_circ_000271	LOC108003566	NW_016017567.1	+	1759008	1759253	246	Antisense
novel_circ_000272	LOC108003605	NW_016017567.1	+	1918599	1995802	77204	Exon–intron
novel_circ_000273	LOC108003544	NW_016017567.1	-	1931205	1932425	338	Exonic
novel_circ_000274	LOC108003578	NW_016017567.1	+	2015361	2015754	394	Exonic
novel_circ_000275	LOC108003578	NW_016017567.1	+	2015361	2015760	400	Exon–intron
novel_circ_000276	LOC108003639	NW_016017567.1	-	2269221	2270599	1379	Exonic
novel_circ_000277	LOC108003614	NW_016017567.1	+	2499235	2499516	282	Exonic
novel_circ_000278	LOC108003479	NW_016017567.1	-	2560409	2560768	360	Exonic
novel_circ_000279	LOC108003568	NW_016017567.1	+	2706956	2709136	1121	Exon–intron
novel_circ_000280	LOC108003568	NW_016017567.1	+	2706956	2711438	944	Exonic
novel_circ_000281	LOC108003634	NW_016017567.1	+	2762062	2762331	270	Antisense
novel_circ_000282	LOC108003534	NW_016017567.1	+	2841231	2843190	1441	Exonic
novel_circ_000283	LOC108003536	NW_016017567.1	+	2863522	2863719	198	Antisense
novel_circ_000284	LOC108003597	NW_016017567.1	+	3150889	3151348	366	Exonic
novel_circ_000285	LOC108003484	NW_016017567.1	+	3240424	3242024	1183	Exonic
novel_circ_000286	LOC108003484	NW_016017567.1	+	3240424	3246110	2099	Exonic
novel_circ_000287	LOC108003484	NW_016017567.1	+	3240424	3246122	2111	Exon–intron
novel_circ_000288	LOC108003484	NW_016017567.1	+	3240424	3247649	2627	Exonic

novel_circ_000289	LOC108003484	NW_016017567.1	+	3244942	3246110	916	Exonic
novel_circ_000290	LOC108003484	NW_016017567.1	-	3246614	3247646	1033	Antisense
novel_circ_000291	LOC108003484	NW_016017567.1	+	3246627	3247649	528	Exonic
novel_circ_000292	LOC108003484	NW_016017567.1	+	3246629	3247649	526	Exonic
novel_circ_000293	LOC108003684	NW_016017568.1	-	6726	7186	326	Exonic
novel_circ_000294	LOC108003706	NW_016017577.1	-	4235	4544	310	Exonic
novel_circ_000295	LOC108003706	NW_016017577.1	-	4235	5118	726	Exonic
novel_circ_000296	NA	NW_016017579.1	+	23081	31779	8699	Intergenic region
novel_circ_000297	LOC108003722	NW_016017579.1	+	31565	38732	7075	Exon–intron
novel_circ_000298	LOC108003721	NW_016017579.1	+	170895	172887	1162	Exonic
novel_circ_000299	LOC108003721	NW_016017579.1	+	170895	173742	1428	Exonic
novel_circ_000300	NA	NW_016017579.1	+	251509	251755	247	Intergenic region
novel_circ_000301	NA	NW_016017579.1	+	307187	308479	1293	Intergenic region
novel_circ_000302	NA	NW_016017579.1	+	307187	309701	2515	Intergenic region
novel_circ_000303	NA	NW_016017579.1	+	308272	309701	1430	Intergenic region
novel_circ_000304	NA	NW_016017579.1	-	399928	404161	4234	Intergenic region
novel_circ_000305	LOC108003717	NW_016017579.1	-	489208	489503	296	Exonic
novel_circ_000306	LOC108003717	NW_016017579.1	-	504274	554874	3384	Exonic
novel_circ_000307	LOC108003717	NW_016017579.1	-	532957	554874	2548	Exonic
novel_circ_000308	LOC108003717	NW_016017579.1	-	549929	554874	4946	Exon–intron
novel_circ_000309	LOC108003728	NW_016017590.1	+	98689	99010	322	Exonic
novel_circ_000310	LOC108003728	NW_016017590.1	+	204284	206182	1899	Exon–intron
novel_circ_000311	LOC108003728	NW_016017590.1	+	204284	211018	6735	Exon–intron
novel_circ_000312	LOC108003728	NW_016017590.1	+	261008	267063	6056	Exon–intron
novel_circ_000313	LOC108003729	NW_016017590.1	+	557904	558890	387	Exonic
novel_circ_000314	LOC108003750	NW_016017601.1	+	23201	27033	445	Exonic
novel_circ_000315	LOC108003768	NW_016017601.1	+	137664	165989	23084	Exon–intron
novel_circ_000316	LOC108003765	NW_016017601.1	-	265533	266947	1415	Exon–intron
novel_circ_000317	LOC108003765	NW_016017601.1	-	265533	268036	2504	Exon–intron
novel_circ_000318	LOC108003765	NW_016017601.1	-	335382	340323	4942	Exon–intron
novel_circ_000319	LOC108003763	NW_016017601.1	+	411185	419755	4056	Exonic
novel_circ_000320	LOC108003774	NW_016017601.1	+	489901	496413	4485	Exon–intron
novel_circ_000321	LOC108003804	NW_016017612.1	-	8009	11133	533	Exonic
novel_circ_000322	LOC108003804	NW_016017612.1	-	9735	11133	423	Exonic
novel_circ_000323	LOC108003804	NW_016017612.1	-	15926	17560	1635	Intronic
novel_circ_000324	LOC108003814	NW_016017612.1	+	304678	307148	726	Exonic
novel_circ_000325	LOC108003821	NW_016017612.1	-	389493	389817	325	Exonic
novel_circ_000326	LOC108003826	NW_016017612.1	+	443990	446746	1343	Exonic
novel_circ_000327	LOC108003822	NW_016017612.1	+	564192	566779	1080	Exonic
novel_circ_000328	LOC108003822	NW_016017612.1	+	565704	566779	705	Exonic
novel_circ_000329	LOC108003835	NW_016017623.1	+	30765	33272	830	Exonic
novel_circ_000330	LOC108003835	NW_016017623.1	+	32095	33272	647	Exonic
novel_circ_000331	LOC108003857	NW_016017623.1	+	291902	293075	442	Exonic
novel_circ_000332	LOC108003869	NW_016017634.1	+	46409	47274	393	Exonic
novel_circ_000333	NA	NW_016017634.1	+	61426	61579	154	Intergenic region
novel_circ_000334	LOC108003891	NW_016017634.1	-	235583	240806	2718	Exonic
novel_circ_000335	LOC108003907	NW_016017634.1	+	306964	307179	216	Exon–intron
novel_circ_000336	LOC108003902	NW_016017634.1	+	335954	358651	21072	Exon–intron
novel_circ_000337	LOC114578262	NW_016017634.1	+	396476	397442	967	Exon–intron

novel_circ_000338	LOC114578261	NW_016017634.1	+	425431	425896	466	Exonic
novel_circ_000339	LOC108003879	NW_016017634.1	+	426915	427960	1046	Antisense
novel_circ_000340	LOC108003899	NW_016017634.1	-	486969	489834	537	Exonic
novel_circ_000341	LOC108003899	NW_016017634.1	-	486969	533917	29303	Exon–intron
novel_circ_000342	LOC108003872	NW_016017634.1	-	486969	546338	39925	Exon–intron
novel_circ_000343	LOC108003872	NW_016017634.1	-	489501	546338	37393	Exon–intron
novel_circ_000344	LOC108003872	NW_016017634.1	-	526183	529929	394	Exonic
novel_circ_000345	LOC108003872	NW_016017634.1	-	526183	533917	558	Exonic
novel_circ_000346	LOC108003872	NW_016017634.1	-	526183	546338	711	Exonic
novel_circ_000347	LOC108003872	NW_016017634.1	-	526188	533917	553	Exonic
novel_circ_000348	LOC108003872	NW_016017634.1	-	526188	546338	706	Exonic
novel_circ_000349	LOC108003872	NW_016017634.1	-	526296	533917	445	Exonic
novel_circ_000350	LOC108003872	NW_016017634.1	-	526296	546338	598	Exonic
novel_circ_000351	LOC108003872	NW_016017634.1	-	529772	533917	322	Exonic
novel_circ_000352	LOC108003872	NW_016017634.1	-	529772	546338	475	Exonic
novel_circ_000353	LOC108003872	NW_016017634.1	-	533754	546338	317	Exonic
novel_circ_000354	LOC108003939	NW_016017645.1	+	178447	178697	251	Intronic
novel_circ_000355	LOC108003938	NW_016017645.1	-	236533	237049	517	Exonic
novel_circ_000356	LOC108003938	NW_016017645.1	-	245604	248964	1003	Exonic
novel_circ_000357	LOC108003938	NW_016017645.1	-	248628	248964	337	Exonic
novel_circ_000358	LOC108003938	NW_016017645.1	-	282565	302393	597	Exonic
novel_circ_000359	LOC108003938	NW_016017645.1	-	301999	302393	395	Exonic
novel_circ_000360	LOC108003939	NW_016017645.1	+	356220	373543	17324	Exon–intron
novel_circ_000361	LOC108003947	NW_016017656.1	-	15114	26069	903	Exonic
novel_circ_000362	LOC108003947	NW_016017656.1	-	15422	26069	595	Exonic
novel_circ_000363	LOC108003947	NW_016017656.1	-	25043	26069	417	Exonic
novel_circ_000364	LOC108003950	NW_016017656.1	+	326358	333294	6389	Exon–intron
novel_circ_000365	LOC108003953	NW_016017665.1	-	3700	4860	1161	Antisense
novel_circ_000366	LOC108003954	NW_016017667.1	-	34311	34829	286	Exonic
novel_circ_000367	LOC108003954	NW_016017667.1	-	227899	228352	454	Intronic
novel_circ_000368	LOC108003957	NW_016017670.1	+	4713	4995	203	Exonic
novel_circ_000369	LOC108004049	NW_016017678.1	+	48982	49421	361	Exonic
novel_circ_000370	LOC108004049	NW_016017678.1	+	48982	51408	666	Exonic
novel_circ_000371	LOC108004049	NW_016017678.1	+	70224	73701	3478	Exon–intron
novel_circ_000372	LOC108004042	NW_016017678.1	+	236077	237266	1190	Exonic
novel_circ_000373	LOC108004042	NW_016017678.1	+	236077	237592	1516	Exonic
novel_circ_000374	LOC108004042	NW_016017678.1	+	236345	236742	398	Exonic
novel_circ_000375	LOC108004020	NW_016017678.1	-	525911	527066	832	Exonic
novel_circ_000376	LOC108004024	NW_016017678.1	-	575907	576294	388	Exonic
novel_circ_000377	LOC108004060	NW_016017678.1	-	611703	614465	696	Exonic
novel_circ_000378	LOC108003997	NW_016017678.1	+	660320	691409	3432	Exonic
novel_circ_000379	LOC108003997	NW_016017678.1	+	689395	691409	834	Exonic
novel_circ_000380	LOC108004002	NW_016017678.1	-	749906	751565	1336	Exonic
novel_circ_000381	LOC108004007	NW_016017678.1	-	833808	836736	930	Exonic
novel_circ_000382	LOC108003971	NW_016017678.1	-	892986	893764	380	Exonic
novel_circ_000383	LOC108004015	NW_016017678.1	+	1472802	1478423	241	Exonic
novel_circ_000384	LOC108004015	NW_016017678.1	+	1472802	1486228	8046	Exon–intron
novel_circ_000385	LOC108003973	NW_016017678.1	+	1730937	1731133	197	Intronic
novel_circ_000386	LOC108003973	NW_016017678.1	+	1794240	1797755	463	Exonic

novel_circ_000387	LOC108003973	NW_016017678.1	+	1794240	1800615	765	Exonic
novel_circ_000388	LOC108003973	NW_016017678.1	+	1794240	1802304	1043	Exonic
novel_circ_000389	LOC108003973	NW_016017678.1	+	1797529	1800615	529	Exonic
novel_circ_000390	LOC108003973	NW_016017678.1	+	1797529	1802304	807	Exonic
novel_circ_000391	LOC108004003	NW_016017678.1	-	1919852	1924589	812	Exonic
novel_circ_000392	LOC108004003	NW_016017678.1	-	1979374	1982278	956	Exonic
novel_circ_000393	LOC108004003	NW_016017678.1	-	1979374	2058640	1259	Exonic
novel_circ_000394	LOC108004003	NW_016017678.1	-	1981522	1982278	757	Exonic
novel_circ_000395	LOC108004003	NW_016017678.1	-	2058435	2058640	206	Exonic
novel_circ_000396	LOC108004113	NW_016017679.1	+	47631	49051	642	Exonic
novel_circ_000397	LOC108004067	NW_016017679.1	+	117855	119022	874	Exonic
novel_circ_000398	LOC108004067	NW_016017679.1	+	124486	126068	511	Exonic
novel_circ_000399	LOC108004100	NW_016017679.1	-	228872	229738	867	Intronic
novel_circ_000400	LOC108004068	NW_016017679.1	+	264405	277848	10880	Exon–intron
novel_circ_000401	LOC108004068	NW_016017679.1	+	264405	277865	10897	Exon–intron
novel_circ_000402	LOC108004068	NW_016017679.1	+	264405	278496	10988	Exon–intron
novel_circ_000403	LOC108004068	NW_016017679.1	-	276642	277797	1156	Antisense
novel_circ_000404	LOC108004086	NW_016017679.1	-	420930	430749	9574	Exon–intron
novel_circ_000405	LOC108004096	NW_016017679.1	+	468523	479755	10853	Exon–intron
novel_circ_000406	LOC108004114	NW_016017680.1	-	2239	3445	401	Exonic
novel_circ_000407	LOC108004115	NW_016017682.1	+	2614	4745	325	Exonic
novel_circ_000408	LOC108004115	NW_016017682.1	+	3952	4745	234	Exonic
novel_circ_000409	LOC108004150	NW_016017690.1	+	33020	33397	378	Antisense
novel_circ_000410	LOC108004150	NW_016017690.1	+	33126	36532	3407	Antisense
novel_circ_000411	LOC108004150	NW_016017690.1	-	35913	37000	710	Exonic
novel_circ_000412	LOC108004150	NW_016017690.1	-	36264	36440	177	Exonic
novel_circ_000413	LOC108004150	NW_016017690.1	+	36323	36515	193	Antisense
novel_circ_000414	LOC108004150	NW_016017690.1	+	36500	36949	450	Antisense
novel_circ_000415	LOC108004124	NW_016017690.1	-	219570	220097	528	Antisense
novel_circ_000416	LOC108004124	NW_016017690.1	-	220058	220367	310	Antisense
novel_circ_000417	LOC108004127	NW_016017690.1	+	237217	238100	884	Intronic
novel_circ_000418	LOC108004127	NW_016017690.1	+	237217	250446	13230	Intronic
novel_circ_000419	LOC108004127	NW_016017690.1	+	238408	238560	153	Intronic
novel_circ_000420	LOC108004123	NW_016017690.1	-	262776	263574	799	Exonic
novel_circ_000421	LOC108004127	NW_016017690.1	+	263616	263878	263	Intronic
novel_circ_000422	LOC108004119	NW_016017690.1	-	271052	271418	367	Antisense
novel_circ_000423	LOC108004132	NW_016017690.1	+	473786	475411	521	Exonic
novel_circ_000424	LOC108004151	NW_016017700.1	-	3040	5377	1526	Exonic
novel_circ_000425	LOC108004151	NW_016017700.1	-	3879	5377	941	Exonic
novel_circ_000426	LOC108004151	NW_016017700.1	-	4650	5377	530	Exonic
novel_circ_000427	LOC108004152	NW_016017701.1	-	121037	121855	819	Exonic
novel_circ_000428	LOC108004152	NW_016017701.1	-	184211	185692	1482	Exonic
novel_circ_000429	LOC108004152	NW_016017701.1	-	212960	215256	563	Exonic
novel_circ_000430	LOC108004161	NW_016017701.1	+	380607	381650	1044	Intronic
novel_circ_000431	LOC108004157	NW_016017701.1	-	381100	382597	1115	Exonic
novel_circ_000432	LOC108004173	NW_016017701.1	+	414631	414823	193	Exonic
novel_circ_000433	LOC108004170	NW_016017701.1	-	421471	423270	398	Exonic
novel_circ_000434	LOC108004189	NW_016017710.1	+	3067	4619	1001	Exonic
novel_circ_000435	LOC108004189	NW_016017710.1	+	3067	4866	1076	Exonic

novel_circ_000436	LOC108004198	NW_016017712.1	+	410015	410922	717	Exonic
novel_circ_000437	LOC108004202	NW_016017712.1	-	422736	423816	779	Exonic
novel_circ_000438	LOC108004191	NW_016017712.1	+	464562	464748	187	Exonic
novel_circ_000439	LOC108004215	NW_016017723.1	-	311459	334847	23389	Exon–intron
novel_circ_000440	LOC108004215	NW_016017723.1	-	333941	334847	907	Intronic
novel_circ_000441	LOC108004221	NW_016017730.1	+	544	2071	564	Exonic
novel_circ_000442	LOC108004222	NW_016017733.1	-	2650	6058	248	Exonic
novel_circ_000443	NA	NW_016017734.1	+	464959	466860	1902	Intergenic region
novel_circ_000444	NA	NW_016017734.1	+	464959	471690	6732	Intergenic region
novel_circ_000445	LOC108004244	NW_016017745.1	+	13027	14330	573	Exonic
novel_circ_000446	LOC108004244	NW_016017745.1	+	13027	41397	915	Exonic
novel_circ_000447	LOC108004244	NW_016017745.1	+	13027	89856	1038	Exonic
novel_circ_000448	LOC108004244	NW_016017745.1	+	41056	41397	342	Exonic
novel_circ_000449	LOC108004244	NW_016017745.1	+	170868	174774	3907	Exon–intron
novel_circ_000450	LOC108004278	NW_016017745.1	-	212656	219180	6031	Exon–intron
novel_circ_000451	LOC108004278	NW_016017745.1	-	212656	219394	6245	Exon–intron
novel_circ_000452	LOC108004268	NW_016017745.1	+	296159	297014	194	Exon–intron
novel_circ_000453	LOC108004249	NW_016017745.1	-	342328	342595	268	Exonic
novel_circ_000454	LOC108004260	NW_016017745.1	+	413834	414258	425	Exonic
novel_circ_000455	LOC108004260	NW_016017745.1	+	428794	429689	647	Exonic
novel_circ_000456	LOC108004260	NW_016017745.1	+	428794	431093	956	Exonic
novel_circ_000457	LOC108004261	NW_016017745.1	-	449817	451553	751	Exonic
novel_circ_000458	LOC108004261	NW_016017745.1	-	454837	455594	327	Exonic
novel_circ_000459	LOC108004303	NW_016017756.1	-	77567	79460	774	Exonic
novel_circ_000460	LOC108004303	NW_016017756.1	-	80023	81481	724	Exonic
novel_circ_000461	LOC108004303	NW_016017756.1	+	90280	98768	8489	Antisense
novel_circ_000462	LOC108004299	NW_016017756.1	+	241063	244928	241	Exonic
novel_circ_000463	LOC108004299	NW_016017756.1	+	241063	256316	552	Exonic
novel_circ_000464	LOC108004299	NW_016017756.1	+	241063	257496	756	Exonic
novel_circ_000465	LOC108004299	NW_016017756.1	+	241063	268367	1213	Exonic
novel_circ_000466	LOC108004299	NW_016017756.1	+	244853	256316	387	Exonic
novel_circ_000467	LOC108004299	NW_016017756.1	+	244853	257496	591	Exonic
novel_circ_000468	LOC108004299	NW_016017756.1	+	256006	257496	515	Exonic
novel_circ_000469	LOC108004299	NW_016017756.1	+	267729	268367	457	Exonic
novel_circ_000470	NA	NW_016017756.1	-	353130	354760	1631	Intergenic region
novel_circ_000471	LOC108004334	NW_016017767.1	-	251250	252313	258	Exonic
novel_circ_000472	LOC108004341	NW_016017767.1	-	449238	450950	1076	Exonic
novel_circ_000473	LOC108004341	NW_016017767.1	-	457115	457934	266	Exonic
novel_circ_000474	LOC108004367	NW_016017778.1	+	36545	43617	626	Exonic
novel_circ_000475	LOC108004367	NW_016017778.1	+	36545	46171	2133	Exonic
novel_circ_000476	LOC108004367	NW_016017778.1	+	48765	49616	852	Intronic
novel_circ_000477	LOC108004365	NW_016017778.1	+	98979	99470	344	Exonic
novel_circ_000478	LOC108004361	NW_016017778.1	+	171417	172039	256	Exonic
novel_circ_000479	LOC108004361	NW_016017778.1	+	171879	173347	724	Exonic
novel_circ_000480	LOC114576798	NW_016017789.1	+	100159	106304	5965	Exon–intron
novel_circ_000481	LOC108004474	NW_016017789.1	-	546688	554400	903	Exonic
novel_circ_000482	LOC108004424	NW_016017789.1	+	726310	726570	261	Antisense
novel_circ_000483	LOC108004424	NW_016017789.1	-	747920	759572	1852	Exonic
novel_circ_000484	LOC108004424	NW_016017789.1	-	747920	767453	2542	Exonic

novel_circ_000485	LOC108004424	NW_016017789.1	-	757442	767453	1519	Exonic
novel_circ_000486	LOC108004424	NW_016017789.1	-	763754	764754	339	Exonic
novel_circ_000487	LOC108004424	NW_016017789.1	-	763754	767453	690	Exonic
novel_circ_000488	LOC108004424	NW_016017789.1	-	974584	976408	1825	Exonic
novel_circ_000489	LOC108004463	NW_016017789.1	-	1456832	1457399	266	Exonic
novel_circ_000490	LOC108004397	NW_016017789.1	+	1930460	1932347	526	Exonic
novel_circ_000491	LOC108004397	NW_016017789.1	+	1930460	1934392	616	Exonic
novel_circ_000492	LOC108004397	NW_016017789.1	+	1971048	1972698	572	Exonic
novel_circ_000493	LOC108004505	NW_016017790.1	+	40304	44716	4413	Exon–intron
novel_circ_000494	LOC108004516	NW_016017790.1	-	273826	284130	639	Exonic
novel_circ_000495	LOC108004516	NW_016017790.1	-	273932	284130	533	Exonic
novel_circ_000496	LOC108004516	NW_016017790.1	-	283770	284130	361	Exonic
novel_circ_000497	LOC107992409	NW_016017801.1	-	179489	179775	287	Exonic
novel_circ_000498	NA	NW_016017801.1	+	245573	246116	544	Intergenic region
novel_circ_000499	LOC107992413	NW_016017801.1	+	421439	421737	299	Antisense
novel_circ_000500	LOC107992447	NW_016017812.1	-	89239	91276	631	Exonic
novel_circ_000501	LOC107992422	NW_016017812.1	-	185446	199748	14303	Exon–intron
novel_circ_000502	LOC107992440	NW_016017812.1	+	383397	384137	480	Exonic
novel_circ_000503	LOC107992440	NW_016017812.1	+	383397	385438	805	Exonic
novel_circ_000504	LOC107992423	NW_016017812.1	+	396783	397380	598	Exonic
novel_circ_000505	LOC107992423	NW_016017812.1	+	396783	397703	809	Exonic
novel_circ_000506	LOC107992453	NW_016017820.1	+	1164	2237	1074	Exon–intron
novel_circ_000507	LOC107992453	NW_016017820.1	+	4456	5027	572	Exon–intron
novel_circ_000508	LOC107992502	NW_016017823.1	+	30475	30651	177	Antisense
novel_circ_000509	LOC107992495	NW_016017823.1	-	138558	145387	5343	Exon–intron
novel_circ_000510	LOC107992495	NW_016017823.1	-	154074	154908	444	Exonic
novel_circ_000511	NA	NW_016017823.1	+	164994	165209	216	Intergenic region
novel_circ_000512	LOC107992457	NW_016017823.1	+	206203	206996	794	Exonic
novel_circ_000513	LOC107992457	NW_016017823.1	+	209592	210460	802	Exonic
novel_circ_000514	LOC107992497	NW_016017823.1	-	305953	307284	1063	Exonic
novel_circ_000515	LOC107992469	NW_016017823.1	-	336809	340175	2601	Exonic
novel_circ_000516	LOC107992474	NW_016017823.1	+	350129	352240	1562	Exonic
novel_circ_000517	LOC107992460	NW_016017823.1	+	390511	391196	686	Exonic
novel_circ_000518	LOC107992482	NW_016017823.1	+	395758	396198	331	Exonic
novel_circ_000519	LOC107992482	NW_016017823.1	+	395758	396512	524	Exonic
novel_circ_000520	LOC107992482	NW_016017823.1	+	395758	398088	1433	Exonic
novel_circ_000521	LOC107992482	NW_016017823.1	+	395758	398557	1675	Exonic
novel_circ_000522	LOC107992482	NW_016017823.1	+	396921	398088	909	Exonic
novel_circ_000523	LOC107992483	NW_016017823.1	+	400125	400608	484	Antisense
novel_circ_000524	LOC107992480	NW_016017823.1	+	418611	419785	620	Exonic
novel_circ_000525	LOC107992480	NW_016017823.1	-	419655	420297	643	Antisense
novel_circ_000526	LOC107992487	NW_016017823.1	+	442502	443207	552	Exonic
novel_circ_000527	LOC107992509	NW_016017830.1	-	2169	3406	375	Exonic
novel_circ_000528	LOC107992509	NW_016017830.1	-	2187	3406	357	Exonic
novel_circ_000529	LOC107992509	NW_016017830.1	-	2652	3406	200	Exonic
novel_circ_000530	LOC107992510	NW_016017834.1	-	325956	329221	3266	Exon–intron
novel_circ_000531	LOC107992514	NW_016017834.1	-	413011	417263	979	Exonic
novel_circ_000532	LOC107992514	NW_016017834.1	-	426714	440329	1450	Exonic
novel_circ_000533	LOC107992514	NW_016017834.1	-	438306	440329	311	Exonic

novel_circ_000534	NA	NW_016017845.1	-	44724	45243	520	Intergenic region
novel_circ_000535	NA	NW_016017845.1	-	44778	45243	466	Intergenic region
novel_circ_000536	LOC107992543	NW_016017845.1	-	146297	146513	217	Exonic
novel_circ_000537	LOC107992527	NW_016017845.1	+	192556	195985	551	Exonic
novel_circ_000538	NA	NW_016017845.1	+	306742	307109	368	Intergenic region
novel_circ_000539	LOC107992568	NW_016017856.1	+	303158	307628	4471	Antisense
novel_circ_000540	LOC107992562	NW_016017856.1	+	380913	382183	1072	Exonic
novel_circ_000541	LOC107992562	NW_016017856.1	+	380913	389305	6001	Exon–intron
novel_circ_000542	LOC107992562	NW_016017856.1	+	380913	389797	6493	Exon–intron
novel_circ_000543	LOC107992562	NW_016017856.1	+	380913	394881	4671	Exonic
novel_circ_000544	LOC107992562	NW_016017856.1	+	383440	387877	3501	Exonic
novel_circ_000545	LOC107992612	NW_016017867.1	+	94134	95650	1517	Antisense
novel_circ_000546	LOC107992595	NW_016017867.1	-	175717	178965	202	Exonic
novel_circ_000547	LOC107992595	NW_016017867.1	-	175717	186982	2456	Exonic
novel_circ_000548	LOC107992595	NW_016017867.1	-	178833	186982	2387	Exonic
novel_circ_000549	LOC107992595	NW_016017867.1	-	178833	193363	3397	Exonic
novel_circ_000550	LOC107992595	NW_016017867.1	-	183318	186982	2254	Exonic
novel_circ_000551	LOC107992595	NW_016017867.1	-	183318	193363	3264	Exonic
novel_circ_000552	LOC107992595	NW_016017867.1	-	183318	214190	4311	Exonic
novel_circ_000553	LOC107992595	NW_016017867.1	-	189924	193363	1010	Exonic
novel_circ_000554	LOC107992595	NW_016017867.1	-	191789	193363	974	Exonic
novel_circ_000555	LOC107992695	NW_016017889.1	+	51275	51857	357	Exonic
novel_circ_000556	LOC107992684	NW_016017889.1	-	319919	332620	906	Exonic
novel_circ_000557	LOC107992684	NW_016017889.1	-	366590	376313	9724	Exon–intron
novel_circ_000558	LOC107992808	NW_016017900.1	-	2540	30945	577	Exonic
novel_circ_000559	LOC107992808	NW_016017900.1	-	17240	30945	405	Exonic
novel_circ_000560	LOC107992839	NW_016017900.1	-	244921	245642	722	Exon–intron
novel_circ_000561	LOC107992844	NW_016017900.1	-	557787	558832	561	Exonic
novel_circ_000562	NA	NW_016017900.1	-	605032	605750	719	Intergenic region
novel_circ_000563	NA	NW_016017900.1	+	605316	605507	192	Intergenic region
novel_circ_000564	LOC107992760	NW_016017900.1	+	678045	678537	347	Exonic
novel_circ_000565	LOC107992760	NW_016017900.1	+	678045	694240	648	Exonic
novel_circ_000566	LOC107992763	NW_016017900.1	-	730727	735816	5090	Exon–intron
novel_circ_000567	LOC107992763	NW_016017900.1	-	743065	743446	295	Exonic
novel_circ_000568	LOC107992770	NW_016017900.1	-	799917	801032	464	Exonic
novel_circ_000569	LOC107992720	NW_016017900.1	-	911001	911162	162	Exonic
novel_circ_000570	LOC107992828	NW_016017900.1	-	948700	950317	137	Exonic
novel_circ_000571	LOC107992774	NW_016017900.1	-	964027	989174	6054	Exon–intron
novel_circ_000572	LOC107992741	NW_016017900.1	-	1086111	1090813	4703	Exon–intron
novel_circ_000573	LOC107992741	NW_016017900.1	+	1133851	1134415	565	Antisense
novel_circ_000574	LOC107992818	NW_016017900.1	-	1367340	1372070	485	Exonic
novel_circ_000575	LOC107992818	NW_016017900.1	-	1369198	1372070	348	Exonic
novel_circ_000576	LOC107992818	NW_016017900.1	-	1382411	1382978	568	Exonic
novel_circ_000577	LOC107992818	NW_016017900.1	-	1382730	1387066	381	Exonic
novel_circ_000578	LOC107992818	NW_016017900.1	-	1426481	1429011	2531	Intronic
novel_circ_000579	LOC107992783	NW_016017900.1	-	1823778	1823986	209	Intronic
novel_circ_000580	LOC107992783	NW_016017900.1	+	1823778	1823986	209	Antisense
novel_circ_000581	LOC107992782	NW_016017900.1	-	1846925	1857389	1617	Exonic
novel_circ_000582	LOC107992749	NW_016017900.1	-	2046378	2046730	353	Exonic

novel_circ_000583	LOC107992750	NW_016017900.1	+	2147709	2148183	475	Antisense
novel_circ_000584	LOC107992786	NW_016017900.1	-	2192757	2193106	350	Exonic
novel_circ_000585	LOC107992715	NW_016017900.1	+	2596968	2597893	926	Antisense
novel_circ_000586	LOC107992717	NW_016017900.1	+	2723010	2723682	673	Exonic
novel_circ_000587	LOC107992715	NW_016017900.1	-	2730348	2730617	270	Intronic
novel_circ_000588	NA	NW_016017900.1	+	2805951	2806235	285	Intergenic region
novel_circ_000589	LOC107992804	NW_016017900.1	+	2862689	2871447	8759	Exon–intron
novel_circ_000590	LOC107992804	NW_016017900.1	+	2864518	2871447	6930	Intronic
novel_circ_000591	LOC107992804	NW_016017900.1	+	2870922	2871447	526	Intronic
novel_circ_000592	LOC107992804	NW_016017900.1	+	2870922	2876026	4849	Exon–intron
novel_circ_000593	LOC107992756	NW_016017900.1	-	2919334	2919975	642	Exonic
novel_circ_000594	LOC107992891	NW_016017901.1	+	163583	165990	2408	Exonic
novel_circ_000595	LOC107992884	NW_016017901.1	-	295666	316870	781	Exonic
novel_circ_000596	NA	NW_016017912.1	+	26187	35010	8824	Intergenic region
novel_circ_000597	LOC107992898	NW_016017912.1	+	138750	147875	909	Exonic
novel_circ_000598	LOC107992898	NW_016017912.1	+	141905	147875	5971	Exon–intron
novel_circ_000599	LOC107992899	NW_016017912.1	+	349132	350982	1779	Exon–intron
novel_circ_000600	LOC107992918	NW_016017923.1	+	39088	40519	961	Exonic
novel_circ_000601	NA	NW_016017923.1	-	330468	362397	31930	Intergenic region
novel_circ_000602	NA	NW_016017945.1	-	21302	21607	306	Intergenic region
novel_circ_000603	LOC107992956	NW_016017945.1	-	35432	36135	503	Exonic
novel_circ_000604	LOC107992942	NW_016017945.1	-	118144	118329	186	Exonic
novel_circ_000605	LOC114576884	NW_016017945.1	+	128197	130711	2515	Exonic
novel_circ_000606	LOC107992941	NW_016017945.1	+	234462	251113	657	Exonic
novel_circ_000607	LOC107992981	NW_016017956.1	-	73256	73787	387	Exonic
novel_circ_000608	LOC107992967	NW_016017956.1	-	91411	91708	298	Exon–intron
novel_circ_000609	LOC107992960	NW_016017956.1	+	109254	174346	28756	Exon–intron
novel_circ_000610	LOC107992960	NW_016017956.1	+	109254	195008	28906	Exon–intron
novel_circ_000611	LOC107992960	NW_016017956.1	+	109254	206104	29232	Exon–intron
novel_circ_000612	LOC107992960	NW_016017956.1	+	116045	137526	21482	Exon–intron
novel_circ_000613	LOC107992960	NW_016017956.1	+	116045	174346	21965	Exon–intron
novel_circ_000614	LOC107992960	NW_016017956.1	+	116045	195008	22115	Exon–intron
novel_circ_000615	LOC107992960	NW_016017956.1	+	116045	206104	22441	Exon–intron
novel_circ_000616	LOC107992960	NW_016017956.1	+	116045	211678	22691	Exon–intron
novel_circ_000617	LOC107992960	NW_016017956.1	+	137434	172685	414	Exonic
novel_circ_000618	LOC107992960	NW_016017956.1	+	137434	174346	576	Exonic
novel_circ_000619	LOC107992960	NW_016017956.1	+	137434	195008	726	Exonic
novel_circ_000620	LOC107992960	NW_016017956.1	+	137434	206104	1052	Exonic
novel_circ_000621	LOC107992960	NW_016017956.1	+	137434	211678	1302	Exonic
novel_circ_000622	LOC107992960	NW_016017956.1	+	137434	217416	1447	Exonic
novel_circ_000623	LOC107992960	NW_016017956.1	+	137434	218602	1710	Exonic
novel_circ_000624	LOC107992960	NW_016017956.1	+	137434	220412	1872	Exonic
novel_circ_000625	LOC107992960	NW_016017956.1	+	137434	222196	3656	Exon–intron
novel_circ_000626	LOC107992960	NW_016017956.1	+	172340	195008	658	Exon–intron
novel_circ_000627	LOC107992960	NW_016017956.1	+	172365	172685	321	Exonic
novel_circ_000628	LOC107992960	NW_016017956.1	+	172365	174346	483	Exonic
novel_circ_000629	LOC107992960	NW_016017956.1	+	172365	195008	633	Exonic
novel_circ_000630	LOC107992960	NW_016017956.1	+	172365	206104	959	Exonic
novel_circ_000631	LOC107992960	NW_016017956.1	+	172365	211678	1209	Exonic

novel_circ_000632	LOC107992960	NW_016017956.1	+	172365	217416	1354	Exonic
novel_circ_000633	LOC107992960	NW_016017956.1	+	172365	218602	1617	Exonic
novel_circ_000634	LOC107992960	NW_016017956.1	+	172365	220412	1779	Exonic
novel_circ_000635	LOC107992960	NW_016017956.1	+	172365	236229	4286	Exonic
novel_circ_000636	LOC107992960	NW_016017956.1	+	172365	237632	4874	Exonic
novel_circ_000637	LOC107992960	NW_016017956.1	+	174185	195008	312	Exonic
novel_circ_000638	LOC107992960	NW_016017956.1	+	174185	206104	638	Exonic
novel_circ_000639	LOC107992960	NW_016017956.1	+	194859	206104	476	Exonic
novel_circ_000640	LOC107992960	NW_016017956.1	+	194859	211678	726	Exonic
novel_circ_000641	LOC107992960	NW_016017956.1	+	205779	206104	326	Exonic
novel_circ_000642	LOC107992960	NW_016017956.1	+	205779	211678	576	Exonic
novel_circ_000643	LOC107992960	NW_016017956.1	+	205779	217416	721	Exonic
novel_circ_000644	LOC107992960	NW_016017956.1	+	205804	206047	244	Exonic
novel_circ_000645	LOC107992960	NW_016017956.1	-	205819	217412	11594	Antisense
novel_circ_000646	LOC107992960	NW_016017956.1	+	211429	217416	395	Exonic
novel_circ_000647	LOC107992960	NW_016017956.1	+	211429	218602	658	Exonic
novel_circ_000648	LOC107992960	NW_016017956.1	+	211429	220412	820	Exonic
novel_circ_000649	LOC107992960	NW_016017956.1	+	217272	218602	408	Exonic
novel_circ_000650	LOC107992960	NW_016017956.1	+	217272	222196	2354	Exon–intron
novel_circ_000651	LOC107992960	NW_016017956.1	+	218340	220412	425	Exonic
novel_circ_000652	LOC107992960	NW_016017956.1	+	232874	233657	379	Exonic
novel_circ_000653	LOC107992985	NW_016017956.1	-	253663	254424	762	Exonic
novel_circ_000654	LOC107992962	NW_016017956.1	-	327139	327292	154	Exonic
novel_circ_000655	LOC107992993	NW_016017957.1	-	540	2643	823	Exonic
novel_circ_000656	LOC107993024	NW_016017967.1	-	200836	204006	3171	Antisense
novel_circ_000657	LOC107993024	NW_016017967.1	+	200922	201331	410	Exonic
novel_circ_000658	LOC107993024	NW_016017967.1	-	200976	202957	1982	Antisense
novel_circ_000659	LOC107993024	NW_016017967.1	-	200990	201145	156	Antisense
novel_circ_000660	LOC107993024	NW_016017967.1	+	201001	202105	483	Exonic
novel_circ_000661	LOC107993024	NW_016017967.1	-	201001	202685	1685	Antisense
novel_circ_000662	LOC107993024	NW_016017967.1	+	201001	203661	1951	Exonic
novel_circ_000663	LOC107993024	NW_016017967.1	+	201001	203954	2244	Exonic
novel_circ_000664	LOC107993024	NW_016017967.1	-	201004	203661	2658	Antisense
novel_circ_000665	LOC107993024	NW_016017967.1	+	201004	203661	1948	Exonic
novel_circ_000666	LOC107993024	NW_016017967.1	-	201014	203512	2499	Antisense
novel_circ_000667	LOC107993024	NW_016017967.1	-	201032	203855	2824	Antisense
novel_circ_000668	LOC107993024	NW_016017967.1	-	201043	203701	2659	Antisense
novel_circ_000669	LOC107993024	NW_016017967.1	+	201052	203600	1839	Exonic
novel_circ_000670	LOC107993024	NW_016017967.1	+	201052	203617	1856	Exonic
novel_circ_000671	LOC107993024	NW_016017967.1	-	201052	203617	2566	Antisense
novel_circ_000672	LOC107993024	NW_016017967.1	+	201052	203937	2176	Exonic
novel_circ_000673	LOC107993024	NW_016017967.1	+	201052	203938	2177	Exonic
novel_circ_000674	LOC107993024	NW_016017967.1	-	201057	203875	2819	Antisense
novel_circ_000675	LOC107993024	NW_016017967.1	+	201061	203697	1927	Exonic
novel_circ_000676	LOC107993024	NW_016017967.1	+	201094	202164	449	Exonic
novel_circ_000677	LOC107993024	NW_016017967.1	+	201094	202587	872	Exonic
novel_circ_000678	LOC107993024	NW_016017967.1	+	201120	202160	419	Exonic
novel_circ_000679	LOC107993024	NW_016017967.1	-	201132	202378	1247	Antisense
novel_circ_000680	LOC107993024	NW_016017967.1	-	201139	203875	2737	Antisense

novel_circ_000681	LOC107993024	NW_016017967.1	-	201143	202164	1022	Antisense
novel_circ_000682	LOC107993024	NW_016017967.1	+	201152	202399	626	Exonic
novel_circ_000683	LOC107993024	NW_016017967.1	-	201152	202488	1337	Antisense
novel_circ_000684	LOC107993024	NW_016017967.1	-	201152	203617	2466	Antisense
novel_circ_000685	LOC107993024	NW_016017967.1	-	201155	202138	984	Antisense
novel_circ_000686	LOC107993024	NW_016017967.1	+	201199	202401	581	Exonic
novel_circ_000687	LOC107993024	NW_016017967.1	+	202051	202482	432	Exonic
novel_circ_000688	LOC107993024	NW_016017967.1	-	202057	202399	343	Antisense
novel_circ_000689	LOC107993024	NW_016017967.1	+	202061	202499	439	Exonic
novel_circ_000690	LOC107993024	NW_016017967.1	-	202061	202499	439	Antisense
novel_circ_000691	LOC107993024	NW_016017967.1	+	202068	202428	361	Exonic
novel_circ_000692	LOC107993024	NW_016017967.1	-	202071	202492	422	Antisense
novel_circ_000693	LOC107993024	NW_016017967.1	-	202074	202491	418	Antisense
novel_circ_000694	LOC107993024	NW_016017967.1	-	202075	202491	417	Antisense
novel_circ_000695	LOC107993024	NW_016017967.1	+	202080	202410	331	Exonic
novel_circ_000696	LOC107993024	NW_016017967.1	-	202085	203988	1904	Antisense
novel_circ_000697	LOC107993024	NW_016017967.1	+	202088	202434	347	Exonic
novel_circ_000698	LOC107993024	NW_016017967.1	+	202088	202491	404	Exonic
novel_circ_000699	LOC107993024	NW_016017967.1	-	202088	202491	404	Antisense
novel_circ_000700	LOC107993024	NW_016017967.1	-	202088	202495	408	Antisense
novel_circ_000701	LOC107993024	NW_016017967.1	+	202090	202488	399	Exonic
novel_circ_000702	LOC107993024	NW_016017967.1	+	202090	202495	406	Exonic
novel_circ_000703	LOC107993024	NW_016017967.1	-	202093	202488	396	Antisense
novel_circ_000704	LOC107993024	NW_016017967.1	+	202093	202488	396	Exonic
novel_circ_000705	LOC107993024	NW_016017967.1	+	202093	202495	403	Exonic
novel_circ_000706	LOC107993024	NW_016017967.1	-	202103	202417	315	Antisense
novel_circ_000707	LOC107993024	NW_016017967.1	-	202109	202399	291	Antisense
novel_circ_000708	LOC107993024	NW_016017967.1	+	202109	202399	291	Exonic
novel_circ_000709	LOC107993024	NW_016017967.1	+	202112	202392	281	Exonic
novel_circ_000710	LOC107993024	NW_016017967.1	+	202112	202399	288	Exonic
novel_circ_000711	LOC107993024	NW_016017967.1	-	202112	202399	288	Antisense
novel_circ_000712	LOC107993024	NW_016017967.1	+	202120	202425	306	Exonic
novel_circ_000713	LOC107993024	NW_016017967.1	+	202122	202417	296	Exonic
novel_circ_000714	LOC107993024	NW_016017967.1	-	202125	202428	304	Antisense
novel_circ_000715	LOC107993024	NW_016017967.1	-	202125	202432	308	Antisense
novel_circ_000716	LOC107993024	NW_016017967.1	+	202125	202432	308	Exonic
novel_circ_000717	LOC107993024	NW_016017967.1	+	202136	202401	266	Exonic
novel_circ_000718	LOC107993024	NW_016017967.1	-	202136	202431	296	Antisense
novel_circ_000719	LOC107993024	NW_016017967.1	-	202144	202437	294	Antisense
novel_circ_000720	LOC107993024	NW_016017967.1	+	202148	202401	254	Exonic
novel_circ_000721	LOC107993024	NW_016017967.1	+	202148	202410	263	Exonic
novel_circ_000722	LOC107993024	NW_016017967.1	+	202157	202448	292	Exonic
novel_circ_000723	LOC107993024	NW_016017967.1	-	202165	202417	253	Antisense
novel_circ_000724	LOC107993024	NW_016017967.1	+	202165	202417	253	Exonic
novel_circ_000725	LOC107993024	NW_016017967.1	+	202165	202425	261	Exonic
novel_circ_000726	LOC107993024	NW_016017967.1	-	202165	202425	261	Antisense
novel_circ_000727	LOC107993024	NW_016017967.1	-	202165	202432	268	Antisense
novel_circ_000728	LOC107993024	NW_016017967.1	+	202165	202456	292	Exonic
novel_circ_000729	LOC107993024	NW_016017967.1	+	202235	202513	279	Exonic

novel_circ_000730	LOC107993024	NW_016017967.1	+	202321	202584	264	Exonic
novel_circ_000731	LOC107993024	NW_016017967.1	-	202433	203855	1423	Antisense
novel_circ_000732	LOC107993024	NW_016017967.1	-	202453	203106	654	Antisense
novel_circ_000733	LOC107993024	NW_016017967.1	-	202453	203617	1165	Antisense
novel_circ_000734	LOC107993024	NW_016017967.1	+	202484	203624	1053	Exonic
novel_circ_000735	LOC107993024	NW_016017967.1	-	202487	203868	1382	Antisense
novel_circ_000736	LOC107993024	NW_016017967.1	-	202489	203661	1173	Antisense
novel_circ_000737	LOC107993024	NW_016017967.1	+	202496	203624	1041	Exonic
novel_circ_000738	LOC107993024	NW_016017967.1	+	202499	203620	1034	Exonic
novel_circ_000739	LOC107993024	NW_016017967.1	-	202506	203607	1102	Antisense
novel_circ_000740	LOC107993024	NW_016017967.1	-	202507	203701	1195	Antisense
novel_circ_000741	LOC107993024	NW_016017967.1	-	202508	203617	1110	Antisense
novel_circ_000742	LOC107993024	NW_016017967.1	+	202508	203617	1022	Exonic
novel_circ_000743	LOC107993024	NW_016017967.1	+	202514	202713	200	Exonic
novel_circ_000744	LOC107993024	NW_016017967.1	-	202546	204019	1474	Antisense
novel_circ_000745	LOC107993024	NW_016017967.1	-	202594	203457	864	Antisense
novel_circ_000746	LOC107993024	NW_016017967.1	+	202603	202873	271	Exonic
novel_circ_000747	LOC107993024	NW_016017967.1	+	202605	202855	251	Exonic
novel_circ_000748	LOC107993024	NW_016017967.1	-	202616	202835	220	Antisense
novel_circ_000749	LOC107993024	NW_016017967.1	-	202634	202994	361	Antisense
novel_circ_000750	LOC107993024	NW_016017967.1	-	202634	203071	438	Antisense
novel_circ_000751	LOC107993024	NW_016017967.1	+	202634	203071	438	Exonic
novel_circ_000752	LOC107993024	NW_016017967.1	+	202634	203199	566	Exonic
novel_circ_000753	LOC107993024	NW_016017967.1	-	202637	202858	222	Antisense
novel_circ_000754	LOC107993024	NW_016017967.1	+	202656	202994	339	Exonic
novel_circ_000755	LOC107993024	NW_016017967.1	-	202656	203199	544	Antisense
novel_circ_000756	LOC107993024	NW_016017967.1	-	202656	203990	1335	Antisense
novel_circ_000757	LOC107993024	NW_016017967.1	-	202668	202920	253	Antisense
novel_circ_000758	LOC107993024	NW_016017967.1	-	202679	203071	393	Antisense
novel_circ_000759	LOC107993024	NW_016017967.1	+	202707	202979	273	Exonic
novel_circ_000760	LOC107993024	NW_016017967.1	-	202727	202994	268	Antisense
novel_circ_000761	LOC107993024	NW_016017967.1	+	202742	202994	253	Exonic
novel_circ_000762	LOC107993024	NW_016017967.1	+	202742	203628	799	Exonic
novel_circ_000763	LOC107993024	NW_016017967.1	+	202747	202930	184	Exonic
novel_circ_000764	LOC107993024	NW_016017967.1	-	202801	203148	348	Antisense
novel_circ_000765	LOC107993024	NW_016017967.1	+	202861	203161	301	Exonic
novel_circ_000766	LOC107993024	NW_016017967.1	+	202877	203136	260	Exonic
novel_circ_000767	LOC107993024	NW_016017967.1	+	202879	203161	283	Exonic
novel_circ_000768	LOC107993024	NW_016017967.1	+	202882	203161	280	Exonic
novel_circ_000769	LOC107993024	NW_016017967.1	-	202882	203161	280	Antisense
novel_circ_000770	LOC107993024	NW_016017967.1	+	202890	203074	185	Exonic
novel_circ_000771	LOC107993024	NW_016017967.1	-	202890	203074	185	Antisense
novel_circ_000772	LOC107993024	NW_016017967.1	+	202898	203136	239	Exonic
novel_circ_000773	LOC107993024	NW_016017967.1	-	202907	203056	150	Antisense
novel_circ_000774	LOC107993024	NW_016017967.1	+	202982	203628	559	Exonic
novel_circ_000775	LOC107993024	NW_016017967.1	+	203025	203661	549	Exonic
novel_circ_000776	LOC107993024	NW_016017967.1	-	203200	203617	418	Antisense
novel_circ_000777	LOC107993024	NW_016017967.1	-	203370	203678	309	Antisense
novel_circ_000778	LOC107993024	NW_016017967.1	+	203370	203873	504	Exonic

novel_circ_000779	LOC107993024	NW_016017967.1	-	203503	203875	373	Antisense
novel_circ_000780	LOC107993024	NW_016017967.1	-	203604	204045	442	Antisense
novel_circ_000781	LOC107993024	NW_016017967.1	+	203608	203978	371	Exonic
novel_circ_000782	LOC107993024	NW_016017967.1	+	203618	203855	238	Exonic
novel_circ_000783	LOC107993024	NW_016017967.1	-	203618	203855	238	Antisense
novel_circ_000784	LOC107993024	NW_016017967.1	-	203618	203988	371	Antisense
novel_circ_000785	LOC107993024	NW_016017967.1	+	203618	203989	372	Exonic
novel_circ_000786	LOC107993024	NW_016017967.1	-	203618	203989	372	Antisense
novel_circ_000787	LOC107993024	NW_016017967.1	-	203618	203990	373	Antisense
novel_circ_000788	LOC107993024	NW_016017967.1	+	203618	203990	373	Exonic
novel_circ_000789	LOC107993024	NW_016017967.1	+	203621	203978	358	Exonic
novel_circ_000790	LOC107993024	NW_016017967.1	+	203623	203990	368	Exonic
novel_circ_000791	LOC107993024	NW_016017967.1	-	203629	203978	350	Antisense
novel_circ_000792	LOC107993024	NW_016017967.1	-	203651	203989	339	Antisense
novel_circ_000793	LOC107993024	NW_016017967.1	+	203658	203986	329	Exonic
novel_circ_000794	LOC107993024	NW_016017967.1	+	203662	203975	314	Exonic
novel_circ_000795	LOC107993024	NW_016017967.1	+	203662	203978	317	Exonic
novel_circ_000796	LOC107993024	NW_016017967.1	-	203662	203978	317	Antisense
novel_circ_000797	LOC107993024	NW_016017967.1	+	203676	203989	314	Exonic
novel_circ_000798	LOC107993024	NW_016017967.1	-	203676	203990	315	Antisense
novel_circ_000799	LOC107993024	NW_016017967.1	+	203676	203990	315	Exonic
novel_circ_000800	LOC107993024	NW_016017967.1	-	203678	204051	374	Antisense
novel_circ_000801	LOC107993024	NW_016017967.1	-	203679	203989	311	Antisense
novel_circ_000802	LOC107993024	NW_016017967.1	+	203679	203989	311	Exonic
novel_circ_000803	LOC107993024	NW_016017967.1	+	203679	203990	312	Exonic
novel_circ_000804	LOC107993024	NW_016017967.1	-	203679	203990	312	Antisense
novel_circ_000805	LOC107993024	NW_016017967.1	+	203702	203925	224	Exonic
novel_circ_000806	LOC107993024	NW_016017967.1	-	203702	203925	224	Antisense
novel_circ_000807	LOC107993024	NW_016017967.1	-	203702	203978	277	Antisense
novel_circ_000808	LOC107993024	NW_016017967.1	+	203702	203978	277	Exonic
novel_circ_000809	LOC107993024	NW_016017967.1	-	203702	203988	287	Antisense
novel_circ_000810	LOC107993024	NW_016017967.1	+	203702	203989	288	Exonic
novel_circ_000811	LOC107993024	NW_016017967.1	+	203702	203990	289	Exonic
novel_circ_000812	LOC107993024	NW_016017967.1	-	203702	204003	302	Antisense
novel_circ_000813	LOC107993024	NW_016017967.1	-	203702	204042	341	Antisense
novel_circ_000814	LOC107993024	NW_016017967.1	+	203702	204042	341	Exonic
novel_circ_000815	LOC107993039	NW_016017967.1	+	359494	361472	1979	Antisense
novel_circ_000816	LOC107993039	NW_016017967.1	+	359539	360209	671	Antisense
novel_circ_000817	LOC107993039	NW_016017967.1	+	359927	361340	1414	Antisense
novel_circ_000818	LOC107993039	NW_016017967.1	+	359927	361514	1588	Antisense
novel_circ_000819	LOC107993039	NW_016017967.1	-	359939	361529	680	Exonic
novel_circ_000820	LOC107993039	NW_016017967.1	+	359939	361529	1591	Antisense
novel_circ_000821	LOC107993039	NW_016017967.1	-	359969	361487	608	Exonic
novel_circ_000822	LOC107993039	NW_016017967.1	+	360015	361465	1451	Antisense
novel_circ_000823	LOC107993039	NW_016017967.1	-	360130	361542	502	Exonic
novel_circ_000824	LOC107993039	NW_016017967.1	+	360159	361468	1310	Antisense
novel_circ_000825	LOC107993039	NW_016017967.1	-	361196	361542	347	Exonic
novel_circ_000826	LOC107993039	NW_016017967.1	+	361223	361542	320	Antisense
novel_circ_000827	LOC107993039	NW_016017967.1	-	361223	361542	320	Exonic

novel_circ_000828	LOC107993039	NW_016017967.1	+	361223	361568	346	Antisense
novel_circ_000829	LOC107993039	NW_016017967.1	+	361245	361533	289	Antisense
novel_circ_000830	LOC107993039	NW_016017967.1	-	361253	361581	329	Exonic
novel_circ_000831	LOC107993039	NW_016017967.1	-	361259	361581	323	Exonic
novel_circ_000832	LOC107993049	NW_016017978.1	-	294713	297571	2859	Antisense
novel_circ_000833	LOC107993057	NW_016017978.1	-	378904	379315	266	Exonic
novel_circ_000834	LOC107993072	NW_016017989.1	+	172285	172606	322	Exonic
novel_circ_000835	LOC107993064	NW_016017989.1	+	333184	336827	507	Exonic
novel_circ_000836	LOC107993096	NW_016018000.1	+	46326	46689	364	Exonic
novel_circ_000837	LOC107993120	NW_016018000.1	+	100915	102157	879	Exonic
novel_circ_000838	LOC107993111	NW_016018000.1	+	156220	158609	2390	Antisense
novel_circ_000839	LOC107993111	NW_016018000.1	-	158527	159123	510	Exonic
novel_circ_000840	LOC107993111	NW_016018000.1	-	247384	256599	408	Exonic
novel_circ_000841	LOC107993119	NW_016018000.1	-	282199	282816	618	Exonic
novel_circ_000842	LOC107993101	NW_016018000.1	-	311971	314619	2649	Exonic
novel_circ_000843	LOC107993124	NW_016018000.1	+	360689	361265	577	Exonic
novel_circ_000844	LOC107993192	NW_016018011.1	-	514831	517119	643	Exonic
novel_circ_000845	LOC107993192	NW_016018011.1	-	514831	521159	873	Exonic
novel_circ_000846	LOC107993192	NW_016018011.1	-	514831	522236	989	Exonic
novel_circ_000847	LOC107993192	NW_016018011.1	-	514831	527924	1306	Exonic
novel_circ_000848	LOC107993192	NW_016018011.1	-	520930	527924	663	Exonic
novel_circ_000849	LOC107993192	NW_016018011.1	-	527525	527924	317	Exonic
novel_circ_000850	LOC107993192	NW_016018011.1	-	664474	666478	2005	Intronic
novel_circ_000851	LOC107993192	NW_016018011.1	-	741792	742311	520	Exonic
novel_circ_000852	LOC107993159	NW_016018011.1	+	758838	759058	221	Exonic
novel_circ_000853	LOC107993215	NW_016018011.1	-	772283	790795	1575	Exon–intron
novel_circ_000854	NA	NW_016018011.1	+	988770	1000365	11596	Intergenic region
novel_circ_000855	LOC107993200	NW_016018011.1	+	1865703	1866726	619	Exonic
novel_circ_000856	LOC107993180	NW_016018011.1	-	2089630	2089832	203	Exonic
novel_circ_000857	LOC107993176	NW_016018011.1	-	2385205	2386681	1172	Exonic
novel_circ_000858	LOC107993156	NW_016018011.1	-	2426857	2427331	475	Exon–intron
novel_circ_000859	LOC107993146	NW_016018011.1	+	2800553	2814196	3196	Exonic
novel_circ_000860	LOC107993146	NW_016018011.1	+	2859174	2866576	4491	Exonic
novel_circ_000861	LOC107993146	NW_016018011.1	+	2868780	2869729	357	Exonic
novel_circ_000862	LOC107993146	NW_016018011.1	-	2919907	2920092	186	Antisense
novel_circ_000863	LOC107993233	NW_016018012.1	-	8804	22808	1034	Exon–intron
novel_circ_000864	LOC107993235	NW_016018012.1	-	56853	59670	981	Exonic
novel_circ_000865	LOC107993235	NW_016018012.1	-	71722	75950	1894	Exonic
novel_circ_000866	LOC107993235	NW_016018012.1	-	73467	74177	527	Exonic
novel_circ_000867	LOC107993235	NW_016018012.1	-	74321	75950	730	Exonic
novel_circ_000868	LOC107993236	NW_016018012.1	+	111969	113157	406	Exonic
novel_circ_000869	LOC107993230	NW_016018012.1	-	148034	148231	198	Antisense
novel_circ_000870	LOC107993230	NW_016018012.1	+	179888	181856	297	Exon–intron
novel_circ_000871	LOC107993246	NW_016018023.1	+	199839	223113	3575	Exonic
novel_circ_000872	LOC107993246	NW_016018023.1	+	213107	216608	1845	Exonic
novel_circ_000873	LOC107993246	NW_016018023.1	+	222984	231014	298	Exonic
novel_circ_000874	LOC107993272	NW_016018034.1	-	168939	169963	705	Exonic
novel_circ_000875	LOC107993310	NW_016018045.1	-	9331	11666	1073	Exonic
novel_circ_000876	LOC107993301	NW_016018045.1	-	91185	95869	433	Exonic

novel_circ_000877	LOC107993301	NW_016018045.1	-	95706	95869	164	Exonic
novel_circ_000878	LOC107993301	NW_016018045.1	-	158031	158728	698	Intronic
novel_circ_000879	LOC107993301	NW_016018045.1	-	158031	159559	1529	Intronic
novel_circ_000880	LOC107993308	NW_016018045.1	-	199518	201227	1380	Exonic
novel_circ_000881	LOC107993308	NW_016018045.1	-	199877	201227	1107	Exonic
novel_circ_000882	LOC107993312	NW_016018045.1	-	277040	279392	523	Exonic
novel_circ_000883	LOC107993305	NW_016018045.1	-	326970	329233	586	Exonic
novel_circ_000884	LOC107993305	NW_016018045.1	-	326970	332279	803	Exonic
novel_circ_000885	LOC107993316	NW_016018046.1	-	2680	3468	358	Exonic
novel_circ_000886	NA	NW_016018056.1	-	97455	98034	581	Intergenic region
novel_circ_000887	LOC107993343	NW_016018067.1	+	247016	247251	236	Exon–intron
novel_circ_000888	LOC107993351	NW_016018067.1	-	314757	315009	253	Exonic
novel_circ_000889	LOC107993351	NW_016018067.1	-	314757	315509	439	Exonic
novel_circ_000890	LOC107993351	NW_016018067.1	+	314765	315518	754	Antisense
novel_circ_000891	LOC107993351	NW_016018067.1	-	314765	315518	440	Exonic
novel_circ_000892	LOC107993351	NW_016018067.1	-	315336	315509	174	Exonic
novel_circ_000893	LOC107993351	NW_016018067.1	+	315349	315575	227	Antisense
novel_circ_000894	LOC107993345	NW_016018067.1	-	342096	344385	945	Exonic
novel_circ_000895	LOC107993361	NW_016018069.1	-	3613	4278	504	Exonic
novel_circ_000896	LOC107993367	NW_016018078.1	+	222729	231108	481	Exonic
novel_circ_000897	LOC114576921	NW_016018089.1	+	21398	21550	153	Antisense
novel_circ_000898	LOC107993399	NW_016018089.1	+	159565	161243	811	Exonic
novel_circ_000899	LOC107993428	NW_016018100.1	+	57237	58025	321	Exonic
novel_circ_000900	LOC107993429	NW_016018100.1	+	130970	136798	5829	Exon–intron
novel_circ_000901	LOC107993429	NW_016018100.1	+	134748	199369	3566	Exon–intron
novel_circ_000902	LOC107993429	NW_016018100.1	+	136056	199369	2258	Exon–intron
novel_circ_000903	LOC107993429	NW_016018100.1	+	199038	199369	332	Exonic
novel_circ_000904	LOC107993429	NW_016018100.1	+	222078	223027	721	Exonic
novel_circ_000905	LOC107993447	NW_016018111.1	-	93498	94752	1255	Exonic
novel_circ_000906	LOC107993447	NW_016018111.1	-	116116	116988	339	Exonic
novel_circ_000907	LOC107993447	NW_016018111.1	-	123377	132903	1338	Exonic
novel_circ_000908	LOC107993447	NW_016018111.1	-	126576	127549	974	Exonic
novel_circ_000909	NA	NW_016018122.1	-	4194	4571	378	Intergenic region
novel_circ_000910	LOC107993529	NW_016018122.1	-	338583	339304	722	Antisense
novel_circ_000911	LOC107993529	NW_016018122.1	+	340312	340714	322	Exonic
novel_circ_000912	LOC107993529	NW_016018122.1	+	340312	340719	327	Exonic
novel_circ_000913	LOC107993478	NW_016018122.1	-	1842016	1842994	530	Exonic
novel_circ_000914	LOC107993478	NW_016018122.1	-	1842016	1844865	1137	Exonic
novel_circ_000915	LOC107993478	NW_016018122.1	-	1879077	1897100	18024	Exon–intron
novel_circ_000916	LOC107993487	NW_016018122.1	+	2009990	2010663	674	Exonic
novel_circ_000917	LOC107993499	NW_016018122.1	+	2217216	2223090	1592	Exonic
novel_circ_000918	LOC107993513	NW_016018122.1	-	2849543	2852871	693	Exonic
novel_circ_000919	LOC107993547	NW_016018123.1	-	102898	105851	2954	Exon–intron
novel_circ_000920	LOC107993542	NW_016018123.1	+	279777	280284	420	Exonic
novel_circ_000921	LOC107993544	NW_016018123.1	+	297928	298543	286	Exonic
novel_circ_000922	LOC107993544	NW_016018123.1	+	297928	311315	2221	Exonic
novel_circ_000923	LOC107993557	NW_016018134.1	-	86228	86479	252	Exonic
novel_circ_000924	LOC107993554	NW_016018134.1	-	229935	231685	471	Exonic
novel_circ_000925	LOC107993563	NW_016018134.1	+	310768	312925	1735	Exonic

novel_circ_000926	LOC107993563	NW_016018134.1	+	314647	314798	152	Exonic
novel_circ_000927	LOC107993563	NW_016018134.1	+	315953	316207	255	Exonic
novel_circ_000928	LOC107993574	NW_016018145.1	-	11001	22937	3763	Exon–intron
novel_circ_000929	LOC107993574	NW_016018145.1	-	41899	50305	1010	Exonic
novel_circ_000930	LOC107993580	NW_016018150.1	+	800	3864	2491	Exon–intron
novel_circ_000931	LOC107993580	NW_016018150.1	-	1692	1892	201	Antisense
novel_circ_000932	LOC107993586	NW_016018156.1	-	155522	156221	700	Exon–intron
novel_circ_000933	LOC107993586	NW_016018156.1	-	155522	159364	3843	Exon–intron
novel_circ_000934	LOC114576948	NW_016018156.1	-	196232	197731	1500	Exon–intron
novel_circ_000935	LOC107993600	NW_016018167.1	+	76905	82614	528	Exonic
novel_circ_000936	LOC107993600	NW_016018167.1	+	82332	83027	382	Exonic
novel_circ_000937	LOC107993600	NW_016018167.1	+	85832	86253	315	Exonic
novel_circ_000938	NA	NW_016018178.1	+	34746	36123	1378	Intergenic region
novel_circ_000939	NA	NW_016018178.1	-	53179	57494	4316	Intergenic region
novel_circ_000940	LOC107993614	NW_016018178.1	+	202031	203420	430	Exonic
novel_circ_000941	LOC107993629	NW_016018189.1	-	1618	18258	15245	Exon–intron
novel_circ_000942	LOC107993629	NW_016018189.1	-	1618	18262	15249	Exon–intron
novel_circ_000943	LOC107993656	NW_016018200.1	+	99985	102175	1774	Exonic
novel_circ_000944	LOC107993656	NW_016018200.1	+	99985	105910	2335	Exonic
novel_circ_000945	LOC107993656	NW_016018200.1	+	105034	105910	561	Exonic
novel_circ_000946	LOC107993656	NW_016018200.1	+	108793	112115	717	Exonic
novel_circ_000947	LOC107993657	NW_016018200.1	-	116401	118950	1094	Exonic
novel_circ_000948	LOC107993655	NW_016018200.1	-	155601	163426	1032	Exonic
novel_circ_000949	LOC107993671	NW_016018202.1	-	3552	4723	1172	Exonic
novel_circ_000950	LOC107993687	NW_016018211.1	+	17373	20748	1912	Exonic
novel_circ_000951	LOC107993687	NW_016018211.1	+	17373	22124	2465	Exonic
novel_circ_000952	LOC107993687	NW_016018211.1	+	17373	22676	2837	Exonic
novel_circ_000953	LOC107993694	NW_016018211.1	-	28703	28879	177	Exonic
novel_circ_000954	LOC107993690	NW_016018211.1	+	35923	39614	840	Exonic
novel_circ_000955	LOC107993677	NW_016018211.1	+	169767	176165	2162	Exonic
novel_circ_000956	LOC107993704	NW_016018222.1	-	125030	125642	370	Exonic
novel_circ_000957	LOC107993704	NW_016018222.1	-	125030	125691	419	Exonic
novel_circ_000958	LOC107993706	NW_016018222.1	-	156561	164337	242	Exonic
novel_circ_000959	LOC107993717	NW_016018222.1	-	261726	262250	525	Exonic
novel_circ_000960	LOC107993717	NW_016018222.1	-	261726	277269	712	Exonic
novel_circ_000961	LOC107993721	NW_016018232.1	-	665	1042	300	Exonic
novel_circ_000962	LOC107993804	NW_016018233.1	-	56217	57401	1185	Intronic
novel_circ_000963	LOC107993804	NW_016018233.1	-	56236	57401	1166	Intronic
novel_circ_000964	LOC107993806	NW_016018233.1	-	102840	104495	938	Exonic
novel_circ_000965	LOC107993806	NW_016018233.1	-	103380	104495	662	Exonic
novel_circ_000966	LOC107993806	NW_016018233.1	-	104011	104495	406	Exonic
novel_circ_000967	LOC107993806	NW_016018233.1	-	133618	133894	277	Exonic
novel_circ_000968	LOC107993806	NW_016018233.1	-	133618	139654	415	Exonic
novel_circ_000969	NA	NW_016018233.1	+	310462	311596	1135	Intergenic region
novel_circ_000970	LOC107993787	NW_016018233.1	-	369388	372551	1062	Exonic
novel_circ_000971	LOC107993787	NW_016018233.1	-	369388	375796	2733	Exon–intron
novel_circ_000972	LOC107993787	NW_016018233.1	-	369921	375796	2551	Exon–intron
novel_circ_000973	LOC107993787	NW_016018233.1	-	372276	374670	821	Exon–intron
novel_circ_000974	LOC107993787	NW_016018233.1	-	372276	375796	1947	Exon–intron

novel_circ_000975	LOC107993787	NW_016018233.1	-	374126	375796	1671	Exon–intron
novel_circ_000976	LOC107993786	NW_016018233.1	-	384725	385001	277	Exonic
novel_circ_000977	LOC107993786	NW_016018233.1	-	391135	402157	345	Exonic
novel_circ_000978	LOC107993779	NW_016018233.1	+	522594	524495	1357	Exonic
novel_circ_000979	LOC107993776	NW_016018233.1	-	579163	581652	2490	Exonic
novel_circ_000980	LOC107993750	NW_016018233.1	-	716492	717560	818	Exonic
novel_circ_000981	LOC107993750	NW_016018233.1	-	743274	750055	1136	Exonic
novel_circ_000982	LOC107993750	NW_016018233.1	-	743274	754446	1799	Exonic
novel_circ_000983	LOC107993750	NW_016018233.1	-	753253	754446	444	Exonic
novel_circ_000984	LOC107993751	NW_016018233.1	+	781237	781825	291	Exonic
novel_circ_000985	LOC107993792	NW_016018233.1	+	1035160	1036162	506	Exonic
novel_circ_000986	LOC107993792	NW_016018233.1	+	1035160	1036941	859	Exonic
novel_circ_000987	LOC107993792	NW_016018233.1	+	1036079	1036941	437	Exonic
novel_circ_000988	LOC107993812	NW_016018233.1	-	1316565	1317181	617	Exon–intron
novel_circ_000989	LOC107993726	NW_016018233.1	+	1576232	1577039	741	Exonic
novel_circ_000990	LOC107993726	NW_016018233.1	+	1576232	1584659	961	Exonic
novel_circ_000991	LOC107993726	NW_016018233.1	+	1617625	1619142	661	Exonic
novel_circ_000992	LOC107993726	NW_016018233.1	+	1618767	1621087	607	Exonic
novel_circ_000993	LOC107993726	NW_016018233.1	+	1618767	1622766	808	Exonic
novel_circ_000994	LOC107993729	NW_016018233.1	+	1699714	1700156	443	Exonic
novel_circ_000995	LOC107993727	NW_016018233.1	+	2030819	2035157	1125	Exonic
novel_circ_000996	LOC107993727	NW_016018233.1	+	2031457	2035157	1003	Exonic
novel_circ_000997	LOC107993727	NW_016018233.1	+	2050289	2053462	968	Exonic
novel_circ_000998	LOC107993727	NW_016018233.1	+	2050289	2054512	1380	Exonic
novel_circ_000999	LOC107993727	NW_016018233.1	+	2110498	2115818	337	Exonic
novel_circ_001000	LOC107993877	NW_016018245.1	-	95257	96181	531	Exonic
novel_circ_001001	LOC107993894	NW_016018245.1	+	124226	124562	337	Exonic
novel_circ_001002	LOC107993855	NW_016018245.1	+	192035	209734	17700	Exon–intron
novel_circ_001003	LOC107993856	NW_016018245.1	-	196629	218544	20753	Exon–intron
novel_circ_001004	LOC107993910	NW_016018256.1	+	30208	31669	1462	Exonic
novel_circ_001005	LOC107993914	NW_016018256.1	-	70106	74165	3744	Exon–intron
novel_circ_001006	LOC107993913	NW_016018256.1	-	73212	86892	13681	Exon–intron
novel_circ_001007	LOC107993913	NW_016018256.1	-	76427	86892	10466	Exon–intron
novel_circ_001008	LOC107993913	NW_016018256.1	-	78623	79647	1025	Exonic
novel_circ_001009	LOC107993913	NW_016018256.1	+	79679	79945	267	Antisense
novel_circ_001010	LOC107993913	NW_016018256.1	-	81240	88537	7298	Intronic
novel_circ_001011	LOC107993913	NW_016018256.1	+	81305	81829	525	Antisense
novel_circ_001012	LOC107993913	NW_016018256.1	-	99062	110737	3585	Exonic
novel_circ_001013	LOC107993940	NW_016018256.1	+	197183	198959	1575	Exonic
novel_circ_001014	LOC107993940	NW_016018256.1	+	197183	199739	2159	Exonic
novel_circ_001015	LOC107993938	NW_016018256.1	-	226978	229595	413	Exonic
novel_circ_001016	LOC107993938	NW_016018256.1	-	231418	234654	809	Exonic
novel_circ_001017	LOC107993938	NW_016018256.1	-	234301	234646	346	Exonic
novel_circ_001018	LOC107993938	NW_016018256.1	-	234301	234654	354	Exonic
novel_circ_001019	LOC107993921	NW_016018256.1	-	274704	276473	1524	Exonic
novel_circ_001020	LOC107993964	NW_016018278.1	+	91790	94425	1638	Exonic
novel_circ_001021	LOC107993959	NW_016018278.1	-	290238	291040	803	Antisense
novel_circ_001022	LOC107993980	NW_016018280.1	+	1270	1919	414	Exon–intron
novel_circ_001023	LOC114577003	NW_016018290.1	-	1809	3301	728	Exonic

novel_circ_001024	LOC107993988	NW_016018293.1	+	2255	3691	367	Exonic
novel_circ_001025	LOC107993995	NW_016018300.1	-	9327	19610	961	Exonic
novel_circ_001026	LOC107993995	NW_016018300.1	-	30201	32039	1097	Exonic
novel_circ_001027	LOC107993995	NW_016018300.1	-	34078	35653	777	Exonic
novel_circ_001028	LOC107993995	NW_016018300.1	-	141615	142097	483	Exon–intron
novel_circ_001029	LOC107994002	NW_016018311.1	+	69953	71213	970	Exonic
novel_circ_001030	LOC107994002	NW_016018311.1	+	69953	72262	1444	Exonic
novel_circ_001031	LOC107994002	NW_016018311.1	+	71055	72262	633	Exonic
novel_circ_001032	LOC107994002	NW_016018311.1	+	90933	91923	546	Exonic
novel_circ_001033	LOC107994002	NW_016018311.1	+	91191	94796	679	Exonic
novel_circ_001034	LOC107994002	NW_016018311.1	+	108830	109132	303	Exonic
novel_circ_001035	LOC107994021	NW_016018311.1	+	230657	231206	382	Exonic
novel_circ_001036	LOC107994046	NW_016018322.1	+	15072	20384	4868	Exon–intron
novel_circ_001037	LOC107994052	NW_016018322.1	-	154094	158689	4596	Antisense
novel_circ_001038	LOC107994052	NW_016018322.1	+	154094	158689	3798	Exon–intron
novel_circ_001039	LOC107994052	NW_016018322.1	+	154098	158450	3555	Exon–intron
novel_circ_001040	LOC107994052	NW_016018322.1	+	154098	158692	3797	Exon–intron
novel_circ_001041	LOC107994052	NW_016018322.1	+	154098	158876	3981	Exon–intron
novel_circ_001042	LOC107994052	NW_016018322.1	+	154101	158692	3794	Exon–intron
novel_circ_001043	LOC107994034	NW_016018322.1	+	193489	196265	1218	Exonic
novel_circ_001044	LOC107994034	NW_016018322.1	+	195363	196612	886	Exonic
novel_circ_001045	LOC107994062	NW_016018333.1	+	134807	136738	1932	Exonic
novel_circ_001046	LOC107994060	NW_016018333.1	-	143916	146583	2451	Exonic
novel_circ_001047	LOC107994173	NW_016018344.1	+	119670	126211	3993	Exonic
novel_circ_001048	LOC107994173	NW_016018344.1	+	124081	126211	423	Exonic
novel_circ_001049	LOC107994173	NW_016018344.1	+	124081	127032	572	Exonic
novel_circ_001050	LOC107994173	NW_016018344.1	+	126884	127032	149	Exonic
novel_circ_001051	LOC107994067	NW_016018344.1	+	429178	433679	3673	Exonic
novel_circ_001052	LOC107994067	NW_016018344.1	+	682992	685268	312	Exonic
novel_circ_001053	LOC107994179	NW_016018344.1	+	951879	952211	333	Exonic
novel_circ_001054	LOC107994179	NW_016018344.1	+	951879	956699	669	Exonic
novel_circ_001055	LOC107994179	NW_016018344.1	+	956522	960510	974	Exonic
novel_circ_001056	LOC107994103	NW_016018344.1	+	997573	1001283	1079	Exonic
novel_circ_001057	LOC107994069	NW_016018344.1	+	1071151	1077720	6570	Antisense
novel_circ_001058	LOC107994069	NW_016018344.1	+	1071151	1077738	6588	Antisense
novel_circ_001059	LOC107994069	NW_016018344.1	+	1071151	1077742	6592	Antisense
novel_circ_001060	LOC107994136	NW_016018344.1	+	1817009	1817240	232	Antisense
novel_circ_001061	LOC107994136	NW_016018344.1	+	1819135	1822467	3333	Antisense
novel_circ_001062	LOC107994074	NW_016018344.1	+	1862715	1863503	342	Exonic
novel_circ_001063	LOC107994074	NW_016018344.1	+	1863434	1863903	391	Exonic
novel_circ_001064	LOC107994074	NW_016018344.1	-	1864021	1864356	336	Antisense
novel_circ_001065	LOC107994155	NW_016018344.1	+	1900102	1905644	1952	Exonic
novel_circ_001066	LOC107994112	NW_016018344.1	-	2037183	2050983	13801	Exon–intron
novel_circ_001067	LOC107994078	NW_016018344.1	-	2078990	2081432	1400	Exonic
novel_circ_001068	LOC107994079	NW_016018344.1	+	2105138	2105708	571	Antisense
novel_circ_001069	LOC107994172	NW_016018344.1	-	2135368	2146392	7228	Exon–intron
novel_circ_001070	LOC107994194	NW_016018345.1	+	191738	193339	1602	Exonic
novel_circ_001071	LOC107994194	NW_016018345.1	+	191738	193344	1607	Exonic
novel_circ_001072	LOC107994194	NW_016018345.1	+	191738	196433	4696	Exon–intron

novel_circ_001073	LOC107994200	NW_016018355.1	+	419	4027	3396	Exon–intron
novel_circ_001074	LOC107994204	NW_016018356.1	+	169024	169476	453	Exonic
novel_circ_001075	LOC107994219	NW_016018367.1	-	7569	20322	11145	Exon–intron
novel_circ_001076	LOC107994228	NW_016018367.1	-	52592	58950	6359	Antisense
novel_circ_001077	LOC107994228	NW_016018367.1	+	53935	54223	289	Exonic
novel_circ_001078	LOC107994227	NW_016018367.1	-	61391	62335	630	Exonic
novel_circ_001079	LOC107994239	NW_016018389.1	-	182287	195389	433	Exon–intron
novel_circ_001080	LOC107994239	NW_016018389.1	-	182287	208876	616	Exonic
novel_circ_001081	LOC107994239	NW_016018389.1	-	182287	208887	627	Exonic
novel_circ_001082	LOC107994239	NW_016018389.1	-	182287	208899	639	Exonic
novel_circ_001083	LOC107994239	NW_016018389.1	-	195251	208887	333	Exonic
novel_circ_001084	LOC107994239	NW_016018389.1	-	198320	198481	162	Intronic
novel_circ_001085	LOC107994239	NW_016018389.1	-	208679	208887	209	Exonic
novel_circ_001086	LOC107994244	NW_016018400.1	-	29290	31787	942	Exon–intron
novel_circ_001087	LOC107994244	NW_016018400.1	-	29425	31787	807	Exon–intron
novel_circ_001088	LOC107994244	NW_016018400.1	-	29476	31787	756	Exon–intron
novel_circ_001089	LOC107994259	NW_016018411.1	+	123599	126043	914	Exonic
novel_circ_001090	LOC107994263	NW_016018411.1	-	131831	133717	845	Exonic
novel_circ_001091	LOC107994263	NW_016018411.1	-	133489	136899	643	Exonic
novel_circ_001092	LOC107994268	NW_016018418.1	+	1548	2472	546	Exonic
novel_circ_001093	LOC107994275	NW_016018422.1	-	44789	58902	253	Exonic
novel_circ_001094	LOC107994275	NW_016018422.1	-	117942	118414	170	Exonic
novel_circ_001095	LOC107994275	NW_016018422.1	-	117942	135806	390	Exonic
novel_circ_001096	LOC107994286	NW_016018422.1	+	180758	180978	221	Exonic
novel_circ_001097	LOC107994283	NW_016018422.1	-	197091	199035	1503	Exonic
novel_circ_001098	LOC107994283	NW_016018422.1	-	200568	203055	650	Exonic
novel_circ_001099	LOC107994283	NW_016018422.1	-	200568	205421	901	Exonic
novel_circ_001100	LOC107994303	NW_016018444.1	+	206913	207065	153	Intronic
novel_circ_001101	LOC107994303	NW_016018444.1	+	208566	208685	120	Intronic
novel_circ_001102	LOC107994302	NW_016018444.1	-	208566	208685	120	Exonic
novel_circ_001103	LOC107994303	NW_016018444.1	+	209427	209627	201	Intronic
novel_circ_001104	LOC107994307	NW_016018455.1	-	124975	125372	398	Antisense
novel_circ_001105	LOC107994407	NW_016018455.1	+	455759	462621	357	Exonic
novel_circ_001106	LOC107994399	NW_016018455.1	-	575024	575431	305	Exonic
novel_circ_001107	LOC107994313	NW_016018455.1	+	690837	691101	265	Exonic
novel_circ_001108	LOC107994408	NW_016018455.1	+	922841	924424	375	Exonic
novel_circ_001109	LOC107994408	NW_016018455.1	+	922841	931908	1898	Exonic
novel_circ_001110	LOC107994408	NW_016018455.1	+	922841	936617	2188	Exonic
novel_circ_001111	LOC107994408	NW_016018455.1	+	922841	950366	2537	Exonic
novel_circ_001112	LOC107994408	NW_016018455.1	+	950018	950366	349	Exonic
novel_circ_001113	LOC107994432	NW_016018455.1	-	978576	980640	923	Exonic
novel_circ_001114	LOC107994441	NW_016018455.1	-	1397665	1397792	128	Exonic
novel_circ_001115	LOC107994363	NW_016018455.1	+	1438410	1438578	169	Antisense
novel_circ_001116	LOC107994421	NW_016018455.1	-	1471228	1472282	827	Exonic
novel_circ_001117	LOC107994484	NW_016018455.1	+	1519079	1537610	5586	Exon–intron
novel_circ_001118	LOC107994461	NW_016018455.1	+	1586953	1587287	335	Antisense
novel_circ_001119	LOC107994347	NW_016018455.1	+	2010461	2010615	155	Exonic
novel_circ_001120	LOC107994338	NW_016018455.1	-	2012462	2014572	496	Exonic
novel_circ_001121	LOC107994480	NW_016018455.1	-	2124032	2124409	302	Exonic

novel_circ_001122	LOC107994397	NW_016018455.1	+	2426701	2427367	482	Exonic
novel_circ_001123	LOC107994397	NW_016018455.1	+	2426701	2427627	742	Exonic
novel_circ_001124	LOC107994397	NW_016018455.1	+	2426701	2428907	1903	Exonic
novel_circ_001125	LOC107994327	NW_016018455.1	-	2442006	2443065	1060	Exonic
novel_circ_001126	LOC107994327	NW_016018455.1	+	2442140	2442550	411	Antisense
novel_circ_001127	LOC107994327	NW_016018455.1	-	2442972	2443390	419	Exonic
novel_circ_001128	LOC107994327	NW_016018455.1	-	2449140	2449532	393	Exonic
novel_circ_001129	LOC107994327	NW_016018455.1	-	2499769	2500796	705	Exonic
novel_circ_001130	LOC107994516	NW_016018467.1	-	49160	49953	508	Exonic
novel_circ_001131	LOC107994516	NW_016018467.1	-	50233	50751	401	Exonic
novel_circ_001132	LOC107994521	NW_016018467.1	-	186733	188216	487	Exonic
novel_circ_001133	NA	NW_016018478.1	+	150733	150913	181	Intergenic region
novel_circ_001134	LOC107994550	NW_016018487.1	-	578	2047	1177	Exonic
novel_circ_001135	LOC107994550	NW_016018487.1	-	847	2047	1117	Exonic
novel_circ_001136	LOC107994552	NW_016018489.1	-	57425	58978	1554	Intronic
novel_circ_001137	LOC107994552	NW_016018489.1	-	137305	140631	3199	Exon–intron
novel_circ_001138	LOC107994566	NW_016018500.1	+	3291	8733	353	Exonic
novel_circ_001139	LOC107994566	NW_016018500.1	+	3291	11757	871	Exonic
novel_circ_001140	LOC107994566	NW_016018500.1	+	8549	11757	703	Exonic
novel_circ_001141	LOC107994568	NW_016018500.1	+	57795	58327	401	Exonic
novel_circ_001142	LOC107994579	NW_016018511.1	+	115625	115950	326	Antisense
novel_circ_001143	NA	NW_016018522.1	+	170320	199896	29577	Intergenic region
novel_circ_001144	LOC107994613	NW_016018533.1	+	19338	19970	633	Exonic
novel_circ_001145	LOC107994613	NW_016018533.1	+	19338	24168	4831	Exon–intron
novel_circ_001146	LOC107994618	NW_016018533.1	-	61443	85681	6567	Exon–intron
novel_circ_001147	LOC107994618	NW_016018533.1	-	85282	85681	400	Intronic
novel_circ_001148	LOC107994623	NW_016018533.1	-	109692	116206	6429	Exon–intron
novel_circ_001149	LOC107994632	NW_016018544.1	-	71683	72257	431	Exonic
novel_circ_001150	LOC107994632	NW_016018544.1	-	72033	75056	439	Exonic
novel_circ_001151	LOC107994632	NW_016018544.1	-	74843	75056	214	Exonic
novel_circ_001152	LOC107994632	NW_016018544.1	-	74843	77150	394	Exonic
novel_circ_001153	LOC107994632	NW_016018544.1	-	90171	91158	988	Intronic
novel_circ_001154	LOC107994632	NW_016018544.1	-	109601	110282	682	Exon–intron
novel_circ_001155	LOC107994632	NW_016018544.1	-	110067	125359	649	Exonic
novel_circ_001156	LOC107994632	NW_016018544.1	-	110067	126379	790	Exonic
novel_circ_001157	LOC107994632	NW_016018544.1	-	124927	126379	574	Exonic
novel_circ_001158	LOC107994632	NW_016018544.1	-	124927	188494	2400	Exon–intron
novel_circ_001159	LOC114577087	NW_016018566.1	+	163726	164376	651	Exon–intron
novel_circ_001160	LOC114577087	NW_016018566.1	+	163726	164717	992	Exon–intron
novel_circ_001161	LOC107994871	NW_016018566.1	+	464608	466833	1824	Exonic
novel_circ_001162	LOC107994781	NW_016018566.1	-	549732	551694	504	Exonic
novel_circ_001163	LOC107994781	NW_016018566.1	-	551301	551694	316	Exonic
novel_circ_001164	LOC107994844	NW_016018566.1	+	1750042	1750573	532	Exon–intron
novel_circ_001165	LOC107994843	NW_016018566.1	+	1768018	1770621	1456	Exonic
novel_circ_001166	LOC107994917	NW_016018566.1	+	1834623	1835456	581	Exonic
novel_circ_001167	LOC107994917	NW_016018566.1	+	1838442	1839122	348	Exonic
novel_circ_001168	LOC107994917	NW_016018566.1	+	1838442	1839858	459	Exonic
novel_circ_001169	LOC107994803	NW_016018566.1	+	1924104	1929756	5653	Exon–intron
novel_circ_001170	LOC107994803	NW_016018566.1	+	1924104	1929894	5791	Exon–intron

novel_circ_001171	LOC107994806	NW_016018566.1	+	1951184	1951746	182	Exonic
novel_circ_001172	LOC107994726	NW_016018566.1	+	1957611	1957943	333	Exonic
novel_circ_001173	LOC107994726	NW_016018566.1	+	1957611	1962881	1271	Exonic
novel_circ_001174	LOC107994726	NW_016018566.1	+	1957611	1976168	2035	Exonic
novel_circ_001175	LOC107994726	NW_016018566.1	+	1972335	1972743	409	Exonic
novel_circ_001176	LOC107994726	NW_016018566.1	+	1972335	1976168	578	Exonic
novel_circ_001177	LOC107994667	NW_016018566.1	-	2390347	2410243	17965	Exon–intron
novel_circ_001178	LOC107994931	NW_016018566.1	-	2498433	2498601	169	Exon–intron
novel_circ_001179	LOC107994850	NW_016018566.1	-	2655462	2656168	707	Exonic
novel_circ_001180	LOC107994933	NW_016018566.1	+	2943502	2946054	1742	Exonic
novel_circ_001181	LOC107994885	NW_016018566.1	-	3187292	3187977	686	Exonic
novel_circ_001182	LOC107994885	NW_016018566.1	-	3188932	3190551	1008	Exonic
novel_circ_001183	LOC107994885	NW_016018566.1	-	3189973	3190502	261	Exonic
novel_circ_001184	LOC107994949	NW_016018566.1	+	3289806	3290604	403	Exonic
novel_circ_001185	LOC107994797	NW_016018566.1	+	3376557	3379011	1130	Exonic
novel_circ_001186	LOC107994797	NW_016018566.1	+	3376557	3411211	1772	Exonic
novel_circ_001187	LOC107994797	NW_016018566.1	+	3408054	3411211	272	Exonic
novel_circ_001188	LOC107994797	NW_016018566.1	+	3472064	3476236	290	Exonic
novel_circ_001189	LOC107994797	NW_016018566.1	+	3472064	3482666	449	Exonic
novel_circ_001190	LOC107994823	NW_016018566.1	+	3541344	3543296	752	Exonic
novel_circ_001191	LOC107994823	NW_016018566.1	+	3541344	3547441	917	Exonic
novel_circ_001192	LOC107994823	NW_016018566.1	+	3541344	3587604	1817	Exonic
novel_circ_001193	LOC107994823	NW_016018566.1	+	3586705	3587604	900	Exonic
novel_circ_001194	LOC107994859	NW_016018566.1	-	3624991	3628585	3595	Antisense
novel_circ_001195	LOC107994859	NW_016018566.1	+	3625141	3627230	314	Exon–intron
novel_circ_001196	LOC107994859	NW_016018566.1	-	3625143	3627247	2105	Antisense
novel_circ_001197	LOC107994859	NW_016018566.1	+	3625143	3627247	329	Exon–intron
novel_circ_001198	LOC107994859	NW_016018566.1	+	3625146	3627247	326	Exonic
novel_circ_001199	LOC107994859	NW_016018566.1	+	3625150	3627209	284	Exonic
novel_circ_001200	LOC107994859	NW_016018566.1	-	3625158	3627246	2089	Antisense
novel_circ_001201	LOC107994859	NW_016018566.1	-	3627344	3628677	1334	Antisense
novel_circ_001202	LOC107994859	NW_016018566.1	-	3627538	3628425	888	Antisense
novel_circ_001203	LOC107994859	NW_016018566.1	-	3627538	3628639	1102	Antisense
novel_circ_001204	LOC107994859	NW_016018566.1	-	3627538	3628647	1110	Antisense
novel_circ_001205	LOC107994859	NW_016018566.1	-	3627651	3628942	1292	Antisense
novel_circ_001206	LOC107994859	NW_016018566.1	-	3627658	3628560	903	Antisense
novel_circ_001207	LOC107994859	NW_016018566.1	-	3627665	3628580	916	Antisense
novel_circ_001208	LOC107994859	NW_016018566.1	+	3627769	3628439	671	Exonic
novel_circ_001209	LOC107994859	NW_016018566.1	-	3627857	3628443	587	Antisense
novel_circ_001210	LOC107994859	NW_016018566.1	+	3627901	3628439	539	Exonic
novel_circ_001211	LOC107994859	NW_016018566.1	+	3627916	3628391	476	Exonic
novel_circ_001212	LOC107994859	NW_016018566.1	+	3627957	3628307	351	Exonic
novel_circ_001213	LOC107994859	NW_016018566.1	+	3627966	3628380	415	Exonic
novel_circ_001214	LOC107994859	NW_016018566.1	-	3627976	3628215	240	Antisense
novel_circ_001215	LOC107994859	NW_016018566.1	-	3627987	3628272	286	Antisense
novel_circ_001216	LOC107994859	NW_016018566.1	-	3628586	3629222	637	Antisense
novel_circ_001217	LOC107994859	NW_016018566.1	+	3628588	3628773	186	Exonic
novel_circ_001218	LOC107994859	NW_016018566.1	-	3628591	3629057	467	Antisense
novel_circ_001219	LOC107994859	NW_016018566.1	-	3628596	3628942	347	Antisense

novel_circ_001220	LOC107994826	NW_016018566.1	+	3751907	3753619	1713	Antisense
novel_circ_001221	LOC107994826	NW_016018566.1	+	3752312	3753120	809	Antisense
novel_circ_001222	LOC107994907	NW_016018566.1	+	3796007	3796446	440	Exonic
novel_circ_001223	LOC107994907	NW_016018566.1	+	3796007	3810847	1017	Exonic
novel_circ_001224	LOC107994810	NW_016018566.1	+	3953848	3954520	583	Exonic
novel_circ_001225	LOC107994675	NW_016018566.1	+	4049806	4050373	117	Exonic
novel_circ_001226	LOC107994675	NW_016018566.1	+	4102633	4103335	264	Exonic
novel_circ_001227	LOC107994970	NW_016018566.1	-	4224849	4228597	1247	Exonic
novel_circ_001228	LOC107994765	NW_016018566.1	-	4363544	4363791	248	Exonic
novel_circ_001229	LOC107994680	NW_016018566.1	+	4370930	4371511	446	Exonic
novel_circ_001230	LOC107994839	NW_016018566.1	+	4505653	4508000	1851	Exonic
novel_circ_001231	LOC107994839	NW_016018566.1	+	4505653	4513589	2152	Exonic
novel_circ_001232	LOC107994839	NW_016018566.1	+	4505653	4528618	5065	Exonic
novel_circ_001233	LOC107994839	NW_016018566.1	+	4513289	4513589	301	Exonic
novel_circ_001234	LOC107994839	NW_016018566.1	+	4513289	4520440	1083	Exonic
novel_circ_001235	LOC107994839	NW_016018566.1	+	4513289	4528618	3214	Exonic
novel_circ_001236	LOC107994839	NW_016018566.1	+	4519822	4520440	619	Exonic
novel_circ_001237	LOC107994839	NW_016018566.1	+	4519822	4525642	2625	Exonic
novel_circ_001238	NA	NW_016018566.1	+	4733505	4735498	1994	Intergenic region
novel_circ_001239	LOC107994951	NW_016018566.1	+	4963065	4967471	306	Exonic
novel_circ_001240	LOC107994951	NW_016018566.1	+	4970923	4973492	1134	Exonic
novel_circ_001241	LOC107994778	NW_016018566.1	+	5237946	5243476	5531	Exon–intron
novel_circ_001242	LOC107994778	NW_016018566.1	+	5519522	5523736	609	Exonic
novel_circ_001243	LOC107994778	NW_016018566.1	+	5519522	5525275	702	Exonic
novel_circ_001244	LOC107994819	NW_016018566.1	-	5581882	5638057	3108	Exonic
novel_circ_001245	LOC107994819	NW_016018566.1	-	5585082	5587471	901	Exonic
novel_circ_001246	LOC107994819	NW_016018566.1	-	5620776	5622500	1725	Intronic
novel_circ_001247	LOC107994900	NW_016018566.1	-	5655975	5657072	1098	Exonic
novel_circ_001248	NA	NW_016018567.1	-	45392	45730	339	Intergenic region
novel_circ_001249	LOC107995162	NW_016018567.1	+	197304	197637	222	Exonic
novel_circ_001250	LOC107995090	NW_016018567.1	-	227993	229723	1731	Exonic
novel_circ_001251	LOC107995056	NW_016018567.1	+	283021	283189	169	Exonic
novel_circ_001252	LOC107995056	NW_016018567.1	-	283055	285331	2277	Antisense
novel_circ_001253	LOC107995056	NW_016018567.1	+	283281	283568	149	Exonic
novel_circ_001254	LOC107995056	NW_016018567.1	+	283538	285365	1480	Exonic
novel_circ_001255	LOC107995056	NW_016018567.1	-	283661	283998	338	Antisense
novel_circ_001256	LOC107995056	NW_016018567.1	-	283681	284967	1287	Antisense
novel_circ_001257	LOC107995056	NW_016018567.1	-	283938	284613	676	Antisense
novel_circ_001258	LOC107995056	NW_016018567.1	-	284455	285339	885	Antisense
novel_circ_001259	LOC107995056	NW_016018567.1	+	284547	286188	1406	Exonic
novel_circ_001260	LOC107995056	NW_016018567.1	+	285266	285560	295	Exonic
novel_circ_001261	LOC107995056	NW_016018567.1	+	285324	285473	150	Exonic
novel_circ_001262	LOC107995056	NW_016018567.1	-	285413	285813	401	Antisense
novel_circ_001263	LOC107995056	NW_016018567.1	+	285474	285788	240	Exonic
novel_circ_001264	LOC107995056	NW_016018567.1	+	285720	286040	321	Exonic
novel_circ_001265	LOC107995056	NW_016018567.1	+	285747	286019	273	Exonic
novel_circ_001266	LOC107995056	NW_016018567.1	+	285747	286040	294	Exonic
novel_circ_001267	LOC107995056	NW_016018567.1	-	285825	286340	516	Antisense
novel_circ_001268	LOC107995056	NW_016018567.1	+	285968	286186	219	Exonic

novel_circ_001269	LOC107995056	NW_016018567.1	+	285998	286184	187	Exonic
novel_circ_001270	LOC107995056	NW_016018567.1	+	286003	286188	186	Exonic
novel_circ_001271	LOC107995056	NW_016018567.1	+	286003	286189	187	Exonic
novel_circ_001272	LOC107995056	NW_016018567.1	+	286004	286189	186	Exonic
novel_circ_001273	LOC107995056	NW_016018567.1	+	286011	286196	186	Exonic
novel_circ_001274	LOC107995056	NW_016018567.1	+	286073	286370	298	Exonic
novel_circ_001275	LOC107995058	NW_016018567.1	+	293354	293826	410	Exonic
novel_circ_001276	LOC107995057	NW_016018567.1	+	315572	324820	8586	Exon–intron
novel_circ_001277	LOC107995057	NW_016018567.1	+	316349	316790	350	Exonic
novel_circ_001278	LOC107995057	NW_016018567.1	+	332783	334925	1778	Exonic
novel_circ_001279	LOC107995111	NW_016018567.1	-	354443	358371	526	Exonic
novel_circ_001280	LOC107995111	NW_016018567.1	-	357263	358371	198	Exonic
novel_circ_001281	LOC107995111	NW_016018567.1	-	357716	358371	656	Exon–intron
novel_circ_001282	LOC107995138	NW_016018567.1	+	368839	370441	913	Exonic
novel_circ_001283	LOC107995163	NW_016018567.1	+	443174	450637	316	Exonic
novel_circ_001284	LOC107995163	NW_016018567.1	+	450386	450637	252	Exonic
novel_circ_001285	LOC107995183	NW_016018567.1	-	828790	829531	367	Exon–intron
novel_circ_001286	LOC107995183	NW_016018567.1	-	830040	830453	338	Exonic
novel_circ_001287	LOC107995082	NW_016018567.1	+	875775	876966	370	Exonic
novel_circ_001288	LOC107995082	NW_016018567.1	+	878561	879022	384	Exonic
novel_circ_001289	LOC107995050	NW_016018567.1	-	1034585	1036647	1565	Exonic
novel_circ_001290	LOC107995031	NW_016018567.1	-	1245044	1246402	1118	Exonic
novel_circ_001291	LOC107995031	NW_016018567.1	-	1247780	1251631	465	Exonic
novel_circ_001292	LOC107995124	NW_016018567.1	-	1313402	1314019	618	Antisense
novel_circ_001293	LOC107995119	NW_016018567.1	-	1329713	1330625	654	Exonic
novel_circ_001294	LOC107995119	NW_016018567.1	-	1384543	1390472	5930	Exon–intron
novel_circ_001295	LOC107995119	NW_016018567.1	-	1386986	1390472	3487	Exon–intron
novel_circ_001296	LOC107995119	NW_016018567.1	-	1387532	1390472	2941	Exon–intron
novel_circ_001297	LOC107995077	NW_016018567.1	+	1779670	1786112	1314	Exonic
novel_circ_001298	LOC107995077	NW_016018567.1	+	1800439	1806382	3187	Exon–intron
novel_circ_001299	LOC107995149	NW_016018567.1	+	1887515	1888433	677	Exonic
novel_circ_001300	LOC107995149	NW_016018567.1	+	1887518	1888433	674	Exonic
novel_circ_001301	LOC107995149	NW_016018567.1	+	1917716	1918042	327	Exonic
novel_circ_001302	LOC107995194	NW_016018579.1	+	147836	156649	3443	Exonic
novel_circ_001303	LOC107995194	NW_016018579.1	+	150489	151582	364	Exonic
novel_circ_001304	LOC107995194	NW_016018579.1	+	150489	156649	3323	Exonic
novel_circ_001305	LOC107995213	NW_016018601.1	-	5955	82890	27077	Exon–intron
novel_circ_001306	LOC107995230	NW_016018612.1	+	110566	110888	323	Exonic
novel_circ_001307	LOC107995239	NW_016018634.1	+	24157	34910	9826	Exon–intron
novel_circ_001308	LOC107995239	NW_016018634.1	+	25272	34910	9639	Exon–intron
novel_circ_001309	LOC107995239	NW_016018634.1	+	74891	76981	543	Exonic
novel_circ_001310	LOC107995239	NW_016018634.1	+	74891	83659	2987	Exon–intron
novel_circ_001311	LOC107995239	NW_016018634.1	+	76711	96241	845	Exonic
novel_circ_001312	LOC107995246	NW_016018656.1	-	32588	41162	243	Exonic
novel_circ_001313	LOC107995246	NW_016018656.1	-	99595	103080	2877	Exonic
novel_circ_001314	LOC107995246	NW_016018656.1	-	99595	137690	3712	Exon–intron
novel_circ_001315	LOC107995246	NW_016018656.1	-	110020	137690	539	Exon–intron
novel_circ_001316	LOC107995327	NW_016018678.1	-	47319	49063	1745	Antisense
novel_circ_001317	LOC107995327	NW_016018678.1	-	47359	48303	945	Antisense

novel_circ_001318	LOC107995327	NW_016018678.1	-	47704	47862	159	Antisense
novel_circ_001319	LOC107995327	NW_016018678.1	-	48177	48410	234	Antisense
novel_circ_001320	LOC107995327	NW_016018678.1	+	88419	92363	3466	Exonic
novel_circ_001321	LOC107995336	NW_016018678.1	-	116847	117202	290	Exonic
novel_circ_001322	LOC107995298	NW_016018678.1	-	246966	247800	835	Antisense
novel_circ_001323	LOC107995298	NW_016018678.1	-	261575	261934	360	Antisense
novel_circ_001324	LOC107995323	NW_016018678.1	-	313662	314044	383	Antisense
novel_circ_001325	LOC107995313	NW_016018678.1	-	546083	550833	4751	Intronic
novel_circ_001326	LOC107995313	NW_016018678.1	-	799714	801409	1696	Exon–intron
novel_circ_001327	LOC107995324	NW_016018678.1	-	865138	866708	1073	Exonic
novel_circ_001328	LOC107995269	NW_016018678.1	+	1213904	1217306	1259	Exon–intron
novel_circ_001329	LOC107995287	NW_016018678.1	-	1293144	1293852	420	Exonic
novel_circ_001330	LOC107995287	NW_016018678.1	-	1319544	1319764	221	Exonic
novel_circ_001331	LOC107995285	NW_016018678.1	+	1402537	1405683	1030	Exonic
novel_circ_001332	LOC107995285	NW_016018678.1	+	1404430	1420629	636	Exonic
novel_circ_001333	LOC114577197	NW_016018678.1	+	2041016	2041998	983	Exon–intron
novel_circ_001334	LOC107995293	NW_016018678.1	-	2080988	2081402	415	Exonic
novel_circ_001335	LOC107995296	NW_016018678.1	+	2086558	2087040	387	Exonic
novel_circ_001336	LOC107995296	NW_016018678.1	+	2086558	2096000	1106	Exonic
novel_circ_001337	LOC107995341	NW_016018678.1	-	2151440	2152333	351	Exonic
novel_circ_001338	LOC107995341	NW_016018678.1	-	2151440	2166793	917	Exonic
novel_circ_001339	NA	NW_016018690.1	+	39825	44199	4375	Intergenic region
novel_circ_001340	LOC114577207	NW_016018690.1	+	105818	107159	1342	Exonic
novel_circ_001341	LOC107995397	NW_016018690.1	-	113636	115109	1474	Intronic
novel_circ_001342	LOC107995397	NW_016018690.1	-	114621	115109	489	Intronic
novel_circ_001343	LOC107995393	NW_016018690.1	-	144638	146130	760	Exonic
novel_circ_001344	LOC107995430	NW_016018712.1	-	37640	38122	308	Exonic
novel_circ_001345	LOC107995430	NW_016018712.1	-	37640	38538	403	Exonic
novel_circ_001346	LOC107995435	NW_016018716.1	+	1541	2040	500	Exonic
novel_circ_001347	LOC107995439	NW_016018734.1	-	13045	42317	2111	Exonic
novel_circ_001348	LOC107995445	NW_016018745.1	+	15479	15821	343	Exonic
novel_circ_001349	LOC107995458	NW_016018756.1	+	46986	52782	1074	Exonic
novel_circ_001350	LOC107995527	NW_016018789.1	+	58278	60664	1653	Exonic
novel_circ_001351	LOC107995537	NW_016018789.1	-	78510	79157	499	Exonic
novel_circ_001352	LOC107995534	NW_016018789.1	-	137487	137726	240	Exonic
novel_circ_001353	LOC107995538	NW_016018789.1	+	850956	851493	538	Exonic
novel_circ_001354	LOC107995538	NW_016018789.1	+	850956	862649	747	Exonic
novel_circ_001355	LOC107995538	NW_016018789.1	+	862441	862649	209	Exonic
novel_circ_001356	LOC107995539	NW_016018789.1	+	1114625	1114882	258	Exonic
novel_circ_001357	LOC107995517	NW_016018789.1	-	1256237	1256427	191	Intronic
novel_circ_001358	LOC107995486	NW_016018789.1	+	1285460	1293290	7277	Exon–intron
novel_circ_001359	LOC107995556	NW_016018789.1	-	1290067	1290791	306	Exon–intron
novel_circ_001360	LOC107995556	NW_016018789.1	-	1290070	1290791	303	Exonic
novel_circ_001361	LOC107995510	NW_016018789.1	-	1620605	1621836	725	Exonic
novel_circ_001362	LOC107995510	NW_016018789.1	-	1683135	1685915	296	Exonic
novel_circ_001363	LOC107995510	NW_016018789.1	-	1683135	1685918	299	Exonic
novel_circ_001364	LOC107995510	NW_016018789.1	-	1683135	1689013	512	Exonic
novel_circ_001365	LOC107995510	NW_016018789.1	-	1683135	1690941	788	Exonic
novel_circ_001366	LOC107995510	NW_016018789.1	-	1688801	1690941	489	Exonic

novel_circ_001367	LOC107995510	NW_016018789.1	-	1690666	1690941	276	Exonic
novel_circ_001368	LOC107995510	NW_016018789.1	-	1811361	1853788	611	Exonic
novel_circ_001369	LOC107995510	NW_016018789.1	+	1837484	1837878	395	Antisense
novel_circ_001370	LOC107995510	NW_016018789.1	-	1985633	2050240	64608	Exon–intron
novel_circ_001371	LOC107995510	NW_016018789.1	+	1995058	1996689	1632	Antisense
novel_circ_001372	LOC107995510	NW_016018789.1	-	2045417	2050240	4824	Exon–intron
novel_circ_001373	LOC107995510	NW_016018789.1	-	2098281	2101225	2945	Exon–intron
novel_circ_001374	LOC114576763	NW_016018823.1	-	27821	32355	4535	Exon–intron
novel_circ_001375	LOC114576763	NW_016018823.1	-	27821	41027	13207	Exon–intron
novel_circ_001376	LOC114576763	NW_016018823.1	-	27821	54567	446	Exonic
novel_circ_001377	LOC107995581	NW_016018845.1	-	23113	30575	583	Exonic
novel_circ_001378	LOC107995595	NW_016018867.1	+	80067	90895	10829	Exon–intron
novel_circ_001379	LOC107995600	NW_016018889.1	+	67013	68067	413	Exonic
novel_circ_001380	LOC107995600	NW_016018889.1	+	67013	69304	605	Exonic
novel_circ_001381	LOC107995610	NW_016018889.1	+	109887	111054	831	Exonic
novel_circ_001382	LOC107995680	NW_016018900.1	+	81736	84733	455	Exonic
novel_circ_001383	LOC107995680	NW_016018900.1	-	84580	98786	14207	Antisense
novel_circ_001384	LOC107995680	NW_016018900.1	+	98491	99890	1400	Exon–intron
novel_circ_001385	LOC107995661	NW_016018900.1	+	137774	138628	855	Intronic
novel_circ_001386	LOC107995651	NW_016018900.1	-	170558	172428	576	Exonic
novel_circ_001387	LOC107995644	NW_016018900.1	-	325779	326409	311	Exonic
novel_circ_001388	LOC107995644	NW_016018900.1	-	360625	361743	704	Exonic
novel_circ_001389	LOC107995644	NW_016018900.1	-	360625	366759	1149	Exonic
novel_circ_001390	LOC107995644	NW_016018900.1	-	366315	366759	445	Exonic
novel_circ_001391	LOC107995620	NW_016018900.1	+	586736	588247	1281	Exonic
novel_circ_001392	LOC107995620	NW_016018900.1	+	589985	591266	818	Exonic
novel_circ_001393	LOC107995622	NW_016018900.1	-	623491	624210	375	Exonic
novel_circ_001394	LOC107995623	NW_016018900.1	-	1175517	1176013	497	Exonic
novel_circ_001395	LOC107995623	NW_016018900.1	-	1178741	1181937	375	Exonic
novel_circ_001396	LOC107995623	NW_016018900.1	-	1183087	1187368	3427	Exonic
novel_circ_001397	LOC107995623	NW_016018900.1	-	1324170	1324606	437	Exonic
novel_circ_001398	LOC114576762	NW_016018900.1	-	1388888	1389615	486	Exonic
novel_circ_001399	LOC107995636	NW_016018900.1	-	1399234	1399548	315	Exonic
novel_circ_001400	LOC107995633	NW_016018900.1	-	1413086	1414577	1492	Exonic
novel_circ_001401	LOC107995633	NW_016018900.1	-	1417339	1419747	2409	Exon–intron
novel_circ_001402	LOC107995633	NW_016018900.1	-	1422136	1422484	349	Exonic
novel_circ_001403	LOC107995633	NW_016018900.1	-	1455410	1456572	695	Exonic
novel_circ_001404	LOC107995718	NW_016018934.1	-	56560	57881	424	Exonic
novel_circ_001405	LOC107995718	NW_016018934.1	-	63288	65225	650	Exonic
novel_circ_001406	LOC107995718	NW_016018934.1	-	64395	68014	634	Exonic
novel_circ_001407	LOC107995718	NW_016018934.1	-	73846	75991	331	Exonic
novel_circ_001408	LOC107995721	NW_016018945.1	-	3894	21324	497	Exonic
novel_circ_001409	LOC107995720	NW_016018945.1	+	38937	40023	349	Exonic
novel_circ_001410	LOC107995727	NW_016018967.1	+	1712	23422	792	Exonic
novel_circ_001411	LOC107995727	NW_016018967.1	+	19805	23422	447	Exonic
novel_circ_001412	LOC107995786	NW_016019011.1	+	50134	55631	396	Exonic
novel_circ_001413	LOC107995786	NW_016019011.1	+	55418	64097	513	Exonic
novel_circ_001414	LOC107995786	NW_016019011.1	+	55418	65468	651	Exonic
novel_circ_001415	LOC107995786	NW_016019011.1	+	63441	64097	299	Exonic

novel_circ_001416	LOC107995786	NW_016019011.1	+	63441	65468	437	Exonic
novel_circ_001417	LOC107995785	NW_016019011.1	+	71324	71568	245	Antisense
novel_circ_001418	LOC107995801	NW_016019011.1	+	151059	170012	458	Exonic
novel_circ_001419	LOC107995801	NW_016019011.1	+	152338	173291	492	Exonic
novel_circ_001420	LOC107995892	NW_016019011.1	+	224087	224679	294	Exonic
novel_circ_001421	LOC107995979	NW_016019011.1	-	242141	242715	439	Exonic
novel_circ_001422	LOC107995905	NW_016019011.1	-	258020	260725	2706	Exon–intron
novel_circ_001423	LOC107995905	NW_016019011.1	-	262716	264082	835	Exonic
novel_circ_001424	LOC107995941	NW_016019011.1	+	301627	302751	1125	Antisense
novel_circ_001425	LOC107995962	NW_016019011.1	+	354098	355415	495	Exonic
novel_circ_001426	LOC107995962	NW_016019011.1	+	355714	356217	504	Exonic
novel_circ_001427	LOC107996015	NW_016019011.1	-	402761	404231	1242	Exonic
novel_circ_001428	LOC107995742	NW_016019011.1	-	833760	834263	504	Antisense
novel_circ_001429	LOC107995872	NW_016019011.1	+	973997	981400	1438	Exon–intron
novel_circ_001430	LOC107995872	NW_016019011.1	+	1001913	1036988	412	Exonic
novel_circ_001431	LOC107995818	NW_016019011.1	-	1391297	1392100	584	Exonic
novel_circ_001432	LOC107995818	NW_016019011.1	-	1391297	1394197	738	Exonic
novel_circ_001433	LOC107995749	NW_016019011.1	+	1660800	1672506	376	Exonic
novel_circ_001434	LOC107995857	NW_016019011.1	+	1854726	1859831	1797	Exonic
novel_circ_001435	LOC107995857	NW_016019011.1	+	1856238	1859831	1652	Exonic
novel_circ_001436	LOC107995857	NW_016019011.1	+	1858043	1859831	1011	Exonic
novel_circ_001437	LOC107995857	NW_016019011.1	+	1863956	1866080	884	Exonic
novel_circ_001438	LOC107996023	NW_016019023.1	+	72884	73119	236	Exonic
novel_circ_001439	LOC107996034	NW_016019045.1	-	21438	22163	726	Exonic
novel_circ_001440	LOC107996037	NW_016019047.1	-	15550	18985	1803	Exonic
novel_circ_001441	LOC107996042	NW_016019049.1	+	53892	54219	328	Exonic
novel_circ_001442	LOC107996042	NW_016019049.1	+	53892	56325	614	Exonic
novel_circ_001443	NA	NW_016019050.1	-	48423	50022	1600	Intergenic region
novel_circ_001444	LOC107996043	NW_016019050.1	+	51206	51618	413	Antisense
novel_circ_001445	LOC107996050	NW_016019052.1	-	30991	34880	3119	Exon–intron
novel_circ_001446	LOC107996050	NW_016019052.1	-	33483	34880	865	Exonic
novel_circ_001447	LOC107996055	NW_016019053.1	+	123769	129096	4019	Exon–intron
novel_circ_001448	LOC107996102	NW_016019053.1	+	154119	156487	1711	Exonic
novel_circ_001449	LOC107996056	NW_016019053.1	+	216751	217510	580	Exonic
novel_circ_001450	LOC107996080	NW_016019053.1	-	302863	306581	3327	Exonic
novel_circ_001451	LOC107996129	NW_016019053.1	+	621244	622237	724	Exonic
novel_circ_001452	LOC107996135	NW_016019053.1	+	678304	679529	705	Exonic
novel_circ_001453	LOC107996135	NW_016019053.1	+	692641	693478	411	Exonic
novel_circ_001454	LOC107996059	NW_016019053.1	-	1066775	1067333	559	Exonic
novel_circ_001455	LOC107996108	NW_016019053.1	+	1202440	1203378	477	Exonic
novel_circ_001456	LOC107996114	NW_016019053.1	-	1386069	1386732	664	Antisense
novel_circ_001457	LOC107996114	NW_016019053.1	+	1386073	1388318	1553	Exonic
novel_circ_001458	LOC107996114	NW_016019053.1	+	1386587	1387069	322	Exonic
novel_circ_001459	LOC107996114	NW_016019053.1	-	1387136	1387779	644	Antisense
novel_circ_001460	LOC107996073	NW_016019053.1	-	1405326	1434902	13681	Exonic
novel_circ_001461	LOC107996073	NW_016019053.1	-	1420122	1434902	2499	Exonic
novel_circ_001462	LOC107996073	NW_016019053.1	-	1426584	1434902	1116	Exonic
novel_circ_001463	LOC107996144	NW_016019053.1	-	1833937	1836948	651	Exonic
novel_circ_001464	LOC107996144	NW_016019053.1	-	1836521	1836948	428	Exonic

novel_circ_001465	LOC107996069	NW_016019053.1	+	1905265	1907851	929	Exonic
novel_circ_001466	LOC107996069	NW_016019053.1	+	1905265	1913488	1027	Exonic
novel_circ_001467	LOC107996152	NW_016019057.1	-	2593	6076	3482	Exonic
novel_circ_001468	LOC107996154	NW_016019061.1	-	1205	2655	1338	Exon–intron
novel_circ_001469	LOC107996159	NW_016019063.1	-	28463	29183	529	Exonic
novel_circ_001470	NA	NW_016019064.1	+	475978	477710	1733	Intergenic region
novel_circ_001471	LOC107996211	NW_016019064.1	+	783123	804356	1496	Exon–intron
novel_circ_001472	LOC107996211	NW_016019064.1	+	783123	826380	386	Exonic
novel_circ_001473	LOC107996211	NW_016019064.1	+	803085	848297	318	Exonic
novel_circ_001474	LOC107996211	NW_016019064.1	+	803085	872868	832	Exonic
novel_circ_001475	LOC107996211	NW_016019064.1	+	826272	848297	265	Exonic
novel_circ_001476	LOC107996211	NW_016019064.1	+	826272	854563	446	Exonic
novel_circ_001477	LOC107996211	NW_016019064.1	+	826272	856080	555	Exonic
novel_circ_001478	LOC107996211	NW_016019064.1	+	854383	856080	290	Exonic
novel_circ_001479	LOC107996211	NW_016019064.1	+	872690	879983	3400	Exon–intron
novel_circ_001480	LOC107996162	NW_016019064.1	+	897967	899726	1760	Exonic
novel_circ_001481	LOC107996218	NW_016019064.1	-	1171385	1171608	224	Exonic
novel_circ_001482	LOC107996186	NW_016019064.1	-	1318211	1318777	331	Exonic
novel_circ_001483	LOC107996185	NW_016019064.1	+	1403019	1403446	428	Exonic
novel_circ_001484	LOC107996185	NW_016019064.1	+	1403019	1404614	1252	Exonic
novel_circ_001485	LOC107996185	NW_016019064.1	+	1403019	1423685	1853	Exonic
novel_circ_001486	LOC107996185	NW_016019064.1	+	1403019	1443738	2209	Exonic
novel_circ_001487	LOC107996185	NW_016019064.1	+	1422884	1424352	1054	Exon–intron
novel_circ_001488	LOC107996164	NW_016019064.1	+	1559443	1564372	4930	Exon–intron
novel_circ_001489	LOC107996164	NW_016019064.1	+	1584286	1595079	1117	Exonic
novel_circ_001490	LOC107996164	NW_016019064.1	+	1590105	1590996	386	Exonic
novel_circ_001491	LOC107996164	NW_016019064.1	+	1590105	1595079	1039	Exonic
novel_circ_001492	LOC107996164	NW_016019064.1	+	1594284	1595079	536	Exonic
novel_circ_001493	LOC107996164	NW_016019064.1	+	1594284	1617169	1846	Exonic
novel_circ_001494	LOC107996164	NW_016019064.1	+	1610135	1611207	904	Exonic
novel_circ_001495	LOC107996164	NW_016019064.1	+	1610138	1611207	901	Exonic
novel_circ_001496	LOC107996164	NW_016019064.1	+	1616547	1617169	406	Exonic
novel_circ_001497	LOC107996164	NW_016019064.1	+	1616547	1619701	1365	Exonic
novel_circ_001498	LOC107996164	NW_016019064.1	+	1618256	1619701	959	Exonic
novel_circ_001499	LOC107996164	NW_016019064.1	+	1619355	1620204	437	Exonic
novel_circ_001500	LOC107996167	NW_016019064.1	+	1627233	1628159	368	Exonic
novel_circ_001501	LOC114577315	NW_016019064.1	+	1632080	1632716	230	Exonic
novel_circ_001502	LOC107996165	NW_016019064.1	-	1633180	1635297	2118	Antisense
novel_circ_001503	LOC107996171	NW_016019064.1	+	1649110	1652790	971	Exonic
novel_circ_001504	LOC107996233	NW_016019064.1	+	1705808	1707043	924	Exonic
novel_circ_001505	LOC107996214	NW_016019064.1	-	1756419	1797768	341	Exonic
novel_circ_001506	LOC107996214	NW_016019064.1	+	1762068	1762712	645	Antisense
novel_circ_001507	LOC107996214	NW_016019064.1	+	1762069	1762714	646	Antisense
novel_circ_001508	LOC107996214	NW_016019064.1	+	1762069	1763597	1529	Antisense
novel_circ_001509	LOC107996301	NW_016019075.1	-	65066	69678	875	Exonic
novel_circ_001510	LOC107996319	NW_016019075.1	-	298759	304865	3011	Exonic
novel_circ_001511	LOC107996300	NW_016019075.1	-	516741	518260	636	Exonic
novel_circ_001512	LOC107996248	NW_016019075.1	-	616163	616585	423	Intronic
novel_circ_001513	LOC107996328	NW_016019075.1	-	654480	690795	1870	Exonic

novel_circ_001514	LOC107996328	NW_016019075.1	-	663116	690795	1703	Exonic
novel_circ_001515	LOC107996328	NW_016019075.1	-	683733	690795	1562	Exonic
novel_circ_001516	LOC107996352	NW_016019075.1	+	719748	808963	70152	Exon–intron
novel_circ_001517	LOC107996250	NW_016019075.1	+	1044160	1044687	528	Antisense
novel_circ_001518	LOC107996346	NW_016019075.1	-	1089457	1103341	13885	Exon–intron
novel_circ_001519	LOC107996346	NW_016019075.1	-	1095400	1103341	7942	Exon–intron
novel_circ_001520	LOC107996281	NW_016019075.1	-	1270838	1272015	576	Exonic
novel_circ_001521	LOC107996281	NW_016019075.1	-	1271701	1277914	1277	Exonic
novel_circ_001522	LOC107996281	NW_016019075.1	-	1274897	1277001	622	Exonic
novel_circ_001523	LOC107996281	NW_016019075.1	-	1274897	1277914	785	Exonic
novel_circ_001524	LOC107996281	NW_016019075.1	-	1275346	1276603	390	Exonic
novel_circ_001525	LOC107996281	NW_016019075.1	-	1276428	1277001	286	Exonic
novel_circ_001526	LOC107996281	NW_016019075.1	-	1276428	1286498	1239	Exonic
novel_circ_001527	LOC107996281	NW_016019075.1	-	1277752	1279745	470	Exonic
novel_circ_001528	LOC107996281	NW_016019075.1	-	1285106	1286498	306	Exonic
novel_circ_001529	LOC107996327	NW_016019075.1	-	1598647	1599243	597	Intronic
novel_circ_001530	LOC107996257	NW_016019075.1	-	1759443	1759858	354	Exon–intron
novel_circ_001531	NA	NW_016019086.1	-	2851	3365	515	Intergenic region
novel_circ_001532	LOC107996458	NW_016019086.1	+	103198	104148	807	Exonic
novel_circ_001533	LOC107996464	NW_016019086.1	+	139016	139201	186	Exonic
novel_circ_001534	LOC107996375	NW_016019086.1	+	237649	242636	4988	Exonic
novel_circ_001535	LOC107996375	NW_016019086.1	+	275039	278241	959	Exonic
novel_circ_001536	LOC107996375	NW_016019086.1	+	275039	299670	1122	Exonic
novel_circ_001537	LOC107996375	NW_016019086.1	+	275039	303392	1420	Exonic
novel_circ_001538	LOC107996375	NW_016019086.1	+	275039	305696	1722	Exonic
novel_circ_001539	LOC107996375	NW_016019086.1	+	275039	307911	1935	Exonic
novel_circ_001540	LOC107996375	NW_016019086.1	+	277117	299670	730	Exonic
novel_circ_001541	LOC107996375	NW_016019086.1	+	277117	305696	1330	Exonic
novel_circ_001542	LOC107996375	NW_016019086.1	+	303221	305696	474	Exonic
novel_circ_001543	LOC107996481	NW_016019086.1	+	341774	353073	5980	Exon–intron
novel_circ_001544	LOC107996428	NW_016019086.1	+	434155	435794	503	Exonic
novel_circ_001545	LOC107996470	NW_016019086.1	+	502609	502774	166	Exonic
novel_circ_001546	LOC107996440	NW_016019086.1	+	526730	527679	435	Exonic
novel_circ_001547	LOC107996417	NW_016019086.1	-	575907	578143	1337	Exonic
novel_circ_001548	LOC107996417	NW_016019086.1	-	576556	577477	776	Exonic
novel_circ_001549	LOC107996491	NW_016019086.1	-	771412	772539	991	Exonic
novel_circ_001550	LOC107996475	NW_016019086.1	+	775369	778706	1045	Exonic
novel_circ_001551	LOC107996475	NW_016019086.1	-	775445	779214	3770	Antisense
novel_circ_001552	LOC107996410	NW_016019086.1	-	918130	920738	1019	Exonic
novel_circ_001553	LOC107996410	NW_016019086.1	-	949229	950018	790	Exonic
novel_circ_001554	LOC107996486	NW_016019086.1	-	1049837	1055141	407	Exonic
novel_circ_001555	LOC107996486	NW_016019086.1	-	1049837	1062349	622	Exonic
novel_circ_001556	LOC107996486	NW_016019086.1	-	1105495	1105979	268	Exonic
novel_circ_001557	LOC107996377	NW_016019086.1	+	1234370	1243261	7794	Exon–intron
novel_circ_001558	LOC107996449	NW_016019086.1	-	1272282	1277508	941	Exonic
novel_circ_001559	LOC107996449	NW_016019086.1	-	1276946	1277508	563	Exonic
novel_circ_001560	LOC107996434	NW_016019086.1	+	1391740	1394258	224	Exonic
novel_circ_001561	LOC107996434	NW_016019086.1	+	1391740	1401625	6985	Exon–intron
novel_circ_001562	LOC114577344	NW_016019086.1	+	1508250	1525383	17134	Exon–intron

novel_circ_001563	LOC107996460	NW_016019086.1	+	1650293	1653045	2666	Exonic
novel_circ_001564	LOC107996518	NW_016019097.1	-	318895	319390	496	Exonic
novel_circ_001565	LOC107996561	NW_016019097.1	-	426174	426684	304	Exonic
novel_circ_001566	LOC107996592	NW_016019097.1	-	567385	570302	543	Exonic
novel_circ_001567	LOC107996574	NW_016019097.1	-	648475	649345	415	Exonic
novel_circ_001568	LOC107996577	NW_016019097.1	-	666771	671479	3883	Exon–intron
novel_circ_001569	LOC107996569	NW_016019097.1	-	854198	857763	1902	Exonic
novel_circ_001570	LOC107996539	NW_016019097.1	+	1117866	1119886	476	Exonic
novel_circ_001571	LOC107996539	NW_016019097.1	+	1119746	1123838	1715	Exonic
novel_circ_001572	LOC107996539	NW_016019097.1	+	1120899	1123838	1032	Exonic
novel_circ_001573	LOC107996539	NW_016019097.1	+	1121598	1123356	620	Exonic
novel_circ_001574	LOC107996539	NW_016019097.1	+	1123084	1124298	736	Exon–intron
novel_circ_001575	LOC107996539	NW_016019097.1	+	1123180	1124298	640	Exonic
novel_circ_001576	LOC107996582	NW_016019097.1	+	1389582	1392727	572	Exonic
novel_circ_001577	LOC107996582	NW_016019097.1	+	1398726	1398924	199	Exonic
novel_circ_001578	LOC107996593	NW_016019097.1	+	1420854	1425285	323	Exonic
novel_circ_001579	LOC107996593	NW_016019097.1	+	1420854	1428365	419	Exonic
novel_circ_001580	LOC107996593	NW_016019097.1	+	1420854	1431248	534	Exonic
novel_circ_001581	LOC107996552	NW_016019097.1	-	1444058	1446038	677	Exonic
novel_circ_001582	LOC107996550	NW_016019097.1	+	1473105	1473575	299	Exonic
novel_circ_001583	LOC107996550	NW_016019097.1	+	1473105	1480244	1705	Exonic
novel_circ_001584	LOC107996550	NW_016019097.1	+	1473105	1481233	1789	Exonic
novel_circ_001585	LOC107996550	NW_016019097.1	+	1473105	1499563	5060	Exonic
novel_circ_001586	LOC107996550	NW_016019097.1	+	1480083	1488241	1022	Exonic
novel_circ_001587	LOC107996586	NW_016019097.1	-	1597734	1598333	600	Antisense
novel_circ_001588	LOC107996586	NW_016019097.1	+	1598287	1600042	996	Exonic
novel_circ_001589	LOC107996586	NW_016019097.1	+	1598326	1601841	2073	Exonic
novel_circ_001590	LOC107996586	NW_016019097.1	-	1598822	1599523	702	Antisense
novel_circ_001591	LOC107996586	NW_016019097.1	-	1599447	1600713	1267	Antisense
novel_circ_001592	LOC107996586	NW_016019097.1	+	1599447	1600713	771	Exonic
novel_circ_001593	LOC107996586	NW_016019097.1	+	1599454	1600782	833	Exonic
novel_circ_001594	LOC107996586	NW_016019097.1	+	1599509	1600687	683	Exonic
novel_circ_001595	LOC107996586	NW_016019097.1	+	1599512	1600501	593	Exonic
novel_circ_001596	LOC107996586	NW_016019097.1	-	1599586	1600501	916	Antisense
novel_circ_001597	LOC107996586	NW_016019097.1	-	1600224	1600679	456	Antisense
novel_circ_001598	LOC107996586	NW_016019097.1	-	1600291	1600494	204	Antisense
novel_circ_001599	LOC107996586	NW_016019097.1	+	1600679	1601073	313	Exonic
novel_circ_001600	LOC107996586	NW_016019097.1	+	1600694	1601089	314	Exonic
novel_circ_001601	LOC107996586	NW_016019097.1	+	1600694	1601870	776	Exonic
novel_circ_001602	LOC107996586	NW_016019097.1	+	1600702	1601820	718	Exonic
novel_circ_001603	LOC107996586	NW_016019097.1	-	1600710	1601959	1250	Antisense
novel_circ_001604	LOC107996586	NW_016019097.1	-	1600971	1601870	900	Antisense
novel_circ_001605	LOC107996586	NW_016019097.1	+	1601180	1601807	397	Exonic
novel_circ_001606	LOC107996586	NW_016019097.1	-	1601190	1601788	599	Antisense
novel_circ_001607	LOC107996586	NW_016019097.1	+	1601194	1601791	367	Exonic
novel_circ_001608	LOC107996586	NW_016019097.1	+	1601194	1601824	400	Exonic
novel_circ_001609	LOC107996586	NW_016019097.1	+	1601197	1601774	347	Exonic
novel_circ_001610	LOC107996525	NW_016019097.1	-	1645650	1647072	315	Exonic
novel_circ_001611	LOC107996525	NW_016019097.1	-	1645650	1673934	657	Exonic

novel_circ_001612	LOC107996901	NW_016019108.1	+	10863	11476	376	Exonic
novel_circ_001613	LOC107996620	NW_016019108.1	+	212605	216466	553	Exonic
novel_circ_001614	LOC107996621	NW_016019108.1	-	260836	262379	332	Exonic
novel_circ_001615	LOC107996622	NW_016019108.1	+	498871	504233	4494	Exon–intron
novel_circ_001616	LOC114577381	NW_016019108.1	+	564636	565058	423	Exonic
novel_circ_001617	LOC107996848	NW_016019108.1	-	672889	673531	643	Exonic
novel_circ_001618	LOC107996848	NW_016019108.1	-	679336	679754	419	Exonic
novel_circ_001619	LOC107996803	NW_016019108.1	-	1047997	1058625	451	Exonic
novel_circ_001620	LOC107996803	NW_016019108.1	-	1047997	1059588	589	Exonic
novel_circ_001621	LOC107996803	NW_016019108.1	-	1057811	1058625	280	Exonic
novel_circ_001622	LOC107996803	NW_016019108.1	-	1057811	1059588	418	Exonic
novel_circ_001623	LOC107996882	NW_016019108.1	-	1165911	1166193	283	Antisense
novel_circ_001624	LOC107996808	NW_016019108.1	-	1176277	1177819	431	Exonic
novel_circ_001625	LOC107996838	NW_016019108.1	-	1231411	1232682	887	Exonic
novel_circ_001626	LOC107996778	NW_016019108.1	+	1313545	1314599	650	Exonic
novel_circ_001627	LOC107996778	NW_016019108.1	+	1313545	1316289	1985	Exonic
novel_circ_001628	LOC107996893	NW_016019108.1	-	1988242	1990038	809	Exonic
novel_circ_001629	LOC107996637	NW_016019108.1	-	2347793	2348878	1086	Exon–intron
novel_circ_001630	LOC107996881	NW_016019108.1	+	2499338	2499547	210	Exonic
novel_circ_001631	NA	NW_016019108.1	-	2894558	2896800	2243	Intergenic region
novel_circ_001632	LOC107996738	NW_016019108.1	+	3335266	3336621	446	Exonic
novel_circ_001633	LOC107996738	NW_016019108.1	+	3335266	3338985	752	Exonic
novel_circ_001634	LOC107996810	NW_016019108.1	+	3656807	3658842	2036	Exonic
novel_circ_001635	LOC107996784	NW_016019108.1	+	4720548	4721121	574	Intronic
novel_circ_001636	LOC107996782	NW_016019108.1	+	4769193	4769483	291	Exonic
novel_circ_001637	LOC107996870	NW_016019108.1	+	4829958	4832570	371	Exonic
novel_circ_001638	LOC107996815	NW_016019108.1	-	4849770	4859532	1699	Exonic
novel_circ_001639	LOC107996815	NW_016019108.1	+	4851557	4852559	1003	Antisense
novel_circ_001640	LOC107996815	NW_016019108.1	-	4892057	4896618	4562	Exon–intron
novel_circ_001641	LOC107996907	NW_016019108.1	-	4969148	4971791	1224	Exonic
novel_circ_001642	LOC107996907	NW_016019108.1	-	4970449	4971791	1069	Exonic
novel_circ_001643	LOC107996776	NW_016019108.1	-	5176118	5176680	427	Exonic
novel_circ_001644	LOC107996875	NW_016019108.1	+	5196530	5200191	856	Exonic
novel_circ_001645	LOC107996875	NW_016019108.1	+	5196530	5201177	1030	Exonic
novel_circ_001646	LOC107996788	NW_016019108.1	-	5228232	5229153	271	Exonic
novel_circ_001647	LOC107996648	NW_016019108.1	-	5387163	5388549	291	Exonic
novel_circ_001648	LOC107996648	NW_016019108.1	-	5387163	5425273	15844	Exon–intron
novel_circ_001649	LOC107996648	NW_016019108.1	-	5403596	5425273	15553	Exon–intron
novel_circ_001650	LOC107996648	NW_016019108.1	-	5409413	5410231	382	Exonic
novel_circ_001651	LOC107996648	NW_016019108.1	-	5409413	5425273	15424	Exon–intron
novel_circ_001652	LOC107996970	NW_016019109.1	-	133921	135128	959	Exonic
novel_circ_001653	LOC107996959	NW_016019109.1	+	186501	194186	7199	Exon–intron
novel_circ_001654	LOC107996959	NW_016019109.1	+	186501	194193	7206	Exon–intron
novel_circ_001655	LOC107996959	NW_016019109.1	+	186501	194537	7550	Exon–intron
novel_circ_001656	LOC114577415	NW_016019109.1	-	1275513	1276644	470	Exon–intron
novel_circ_001657	LOC107996951	NW_016019109.1	+	1588675	1589049	375	Antisense
novel_circ_001658	LOC107996951	NW_016019109.1	-	1595440	1603812	8373	Exon–intron
novel_circ_001659	LOC107996951	NW_016019109.1	-	1595440	1609510	14071	Exon–intron
novel_circ_001660	LOC107996966	NW_016019109.1	-	1650721	1656792	3979	Exon–intron

novel_circ_001661	NA	NW_016019112.1	-	15468	15726	259	Intergenic region
novel_circ_001662	LOC107997013	NW_016019119.1	-	5975	10556	1319	Exonic
novel_circ_001663	LOC107997013	NW_016019119.1	-	5975	10690	1453	Exonic
novel_circ_001664	LOC107997116	NW_016019120.1	-	42376	68626	21586	Exon–intron
novel_circ_001665	LOC107997070	NW_016019120.1	+	535758	587856	1195	Exonic
novel_circ_001666	LOC107997070	NW_016019120.1	+	535758	595375	1777	Exonic
novel_circ_001667	LOC107997070	NW_016019120.1	+	535758	607130	4416	Exonic
novel_circ_001668	LOC107997070	NW_016019120.1	+	586772	587856	507	Exonic
novel_circ_001669	LOC107997070	NW_016019120.1	+	586772	597999	1193	Exonic
novel_circ_001670	LOC107997070	NW_016019120.1	+	586772	607130	3728	Exonic
novel_circ_001671	LOC107997070	NW_016019120.1	+	586772	618845	4034	Exonic
novel_circ_001672	LOC107997070	NW_016019120.1	+	586772	626308	5140	Exonic
novel_circ_001673	LOC107997070	NW_016019120.1	+	603022	603857	629	Exonic
novel_circ_001674	LOC107997072	NW_016019120.1	-	669249	670209	961	Exonic
novel_circ_001675	LOC107997072	NW_016019120.1	-	676484	679494	612	Exonic
novel_circ_001676	LOC107997084	NW_016019120.1	+	884604	885094	352	Exonic
novel_circ_001677	LOC107997092	NW_016019120.1	-	901294	902615	1322	Antisense
novel_circ_001678	LOC107997090	NW_016019120.1	+	1172895	1173648	754	Exonic
novel_circ_001679	LOC107997129	NW_016019120.1	+	1316908	1319561	1943	Exonic
novel_circ_001680	LOC107997049	NW_016019120.1	-	1329588	1329948	361	Exonic
novel_circ_001681	LOC107997067	NW_016019120.1	-	1483545	1484595	408	Exonic
novel_circ_001682	LOC107997067	NW_016019120.1	-	1483545	1487416	666	Exonic
novel_circ_001683	LOC107997067	NW_016019120.1	-	1563012	1563834	666	Exonic
novel_circ_001684	NA	NW_016019121.1	+	10003	11270	1268	Intergenic region
novel_circ_001685	LOC107997154	NW_016019131.1	+	55910	57067	1054	Exonic
novel_circ_001686	LOC107997238	NW_016019131.1	+	146899	148208	1226	Exonic
novel_circ_001687	LOC107997224	NW_016019131.1	-	640769	641348	354	Exonic
novel_circ_001688	LOC107997226	NW_016019131.1	-	675958	676772	347	Exonic
novel_circ_001689	LOC107997212	NW_016019131.1	+	793852	797091	522	Exonic
novel_circ_001690	LOC107997212	NW_016019131.1	+	796640	797091	452	Exonic
novel_circ_001691	LOC107997212	NW_016019131.1	+	796640	800670	593	Exonic
novel_circ_001692	LOC107997195	NW_016019131.1	-	849287	858793	9507	Intronic
novel_circ_001693	LOC107997195	NW_016019131.1	+	852229	852435	207	Antisense
novel_circ_001694	LOC107997162	NW_016019131.1	+	941582	945842	333	Exonic
novel_circ_001695	LOC107997218	NW_016019131.1	+	1005203	1006454	632	Exonic
novel_circ_001696	LOC107997223	NW_016019131.1	+	1051914	1056095	661	Exonic
novel_circ_001697	LOC107997163	NW_016019131.1	+	1404000	1406179	910	Exonic
novel_circ_001698	LOC107997163	NW_016019131.1	+	1404000	1409105	1230	Exon–intron
novel_circ_001699	NA	NW_016019131.1	+	1442002	1443076	1075	Intergenic region
novel_circ_001700	NA	NW_016019131.1	-	1596077	1596390	314	Intergenic region
novel_circ_001701	NA	NW_016019131.1	+	1596077	1596390	314	Intergenic region
novel_circ_001702	LOC107997318	NW_016019142.1	-	33544	33994	372	Exonic
novel_circ_001703	LOC107997269	NW_016019142.1	-	188678	192215	218	Exonic
novel_circ_001704	LOC107997269	NW_016019142.1	-	211634	216960	5327	Exon–intron
novel_circ_001705	LOC107997269	NW_016019142.1	-	211634	227791	5382	Exon–intron
novel_circ_001706	LOC107997269	NW_016019142.1	-	260096	260993	318	Exonic
novel_circ_001707	LOC107997278	NW_016019142.1	-	508416	516634	5024	Exonic
novel_circ_001708	LOC107997278	NW_016019142.1	-	511850	512140	215	Exonic
novel_circ_001709	LOC107997278	NW_016019142.1	-	517388	518257	479	Exonic

novel_circ_001710	LOC107997278	NW_016019142.1	-	523617	524553	622	Exonic
novel_circ_001711	LOC107997278	NW_016019142.1	-	523617	530627	811	Exonic
novel_circ_001712	LOC107997341	NW_016019142.1	+	697675	698047	373	Exonic
novel_circ_001713	LOC107997257	NW_016019142.1	+	1023130	1046352	293	Exonic
novel_circ_001714	LOC107997257	NW_016019142.1	+	1069036	1070052	485	Exonic
novel_circ_001715	LOC107997290	NW_016019142.1	+	1151881	1154427	2547	Intronic
novel_circ_001716	LOC107997290	NW_016019142.1	+	1196197	1201149	621	Exonic
novel_circ_001717	LOC107997290	NW_016019142.1	+	1196197	1202253	789	Exonic
novel_circ_001718	LOC107997290	NW_016019142.1	+	1200032	1202253	654	Exonic
novel_circ_001719	LOC107997312	NW_016019142.1	+	1241393	1245670	3592	Exonic
novel_circ_001720	LOC107997259	NW_016019142.1	+	1344083	1345101	702	Exonic
novel_circ_001721	LOC107997401	NW_016019153.1	-	149599	158737	566	Exonic
novel_circ_001722	LOC107997417	NW_016019153.1	+	813253	835801	3990	Exon–intron
novel_circ_001723	LOC107997417	NW_016019153.1	+	813253	836228	4417	Exon–intron
novel_circ_001724	LOC107997417	NW_016019153.1	+	813313	835801	3930	Exon–intron
novel_circ_001725	LOC107997417	NW_016019153.1	+	818647	821921	3275	Intronic
novel_circ_001726	LOC107997448	NW_016019153.1	+	863316	863654	339	Intronic
novel_circ_001727	LOC107997448	NW_016019153.1	+	863316	865211	1896	Exon–intron
novel_circ_001728	LOC107997448	NW_016019153.1	+	863316	870525	2476	Exon–intron
novel_circ_001729	LOC107997448	NW_016019153.1	+	863316	878174	2714	Exon–intron
novel_circ_001730	LOC107997448	NW_016019153.1	+	863323	863654	332	Intronic
novel_circ_001731	LOC107997448	NW_016019153.1	+	863323	865211	1889	Exon–intron
novel_circ_001732	LOC107997448	NW_016019153.1	+	863323	878174	2707	Exon–intron
novel_circ_001733	LOC107997448	NW_016019153.1	-	863457	870487	7031	Antisense
novel_circ_001734	LOC107997448	NW_016019153.1	+	864933	878174	1097	Exonic
novel_circ_001735	LOC107997448	NW_016019153.1	+	868317	868924	399	Exonic
novel_circ_001736	LOC107997448	NW_016019153.1	+	868317	870525	580	Exonic
novel_circ_001737	LOC107997448	NW_016019153.1	+	868317	876900	6955	Exon–intron
novel_circ_001738	LOC107997448	NW_016019153.1	+	868317	878174	818	Exonic
novel_circ_001739	LOC107997448	NW_016019153.1	-	868379	870457	2079	Antisense
novel_circ_001740	LOC107997448	NW_016019153.1	+	875632	878174	2543	Exon–intron
novel_circ_001741	NA	NW_016019153.1	-	887079	887429	351	Intergenic region
novel_circ_001742	NA	NW_016019153.1	+	948337	948687	351	Intergenic region
novel_circ_001743	LOC107997376	NW_016019153.1	+	948337	982987	34651	Antisense
novel_circ_001744	LOC107997458	NW_016019153.1	+	1051730	1052717	445	Exonic
novel_circ_001745	LOC107997446	NW_016019153.1	+	1103648	1107426	2949	Exon–intron
novel_circ_001746	LOC107997446	NW_016019153.1	+	1104621	1107426	2703	Exon–intron
novel_circ_001747	LOC107997380	NW_016019153.1	+	1388711	1404929	3810	Exonic
novel_circ_001748	LOC107997553	NW_016019164.1	-	65712	73685	7475	Exon–intron
novel_circ_001749	LOC107997520	NW_016019164.1	+	118376	120400	2025	Intronic
novel_circ_001750	LOC107997499	NW_016019164.1	+	619189	621706	336	Exonic
novel_circ_001751	LOC107997499	NW_016019164.1	+	642446	644922	816	Exonic
novel_circ_001752	LOC107997489	NW_016019164.1	-	1169104	1173807	1146	Exonic
novel_circ_001753	LOC107997489	NW_016019164.1	-	1171534	1173807	1005	Exonic
novel_circ_001754	LOC107997492	NW_016019164.1	+	1188853	1189440	310	Exonic
novel_circ_001755	LOC107997537	NW_016019164.1	+	1335252	1335984	472	Exonic
novel_circ_001756	LOC107997670	NW_016019175.1	+	134214	135342	1129	Exonic
novel_circ_001757	LOC107997641	NW_016019175.1	+	212185	212935	639	Exonic
novel_circ_001758	LOC107997656	NW_016019175.1	-	265867	266922	1056	Antisense

novel_circ_001759	LOC107997656	NW_016019175.1	+	265869	266923	728	Exonic
novel_circ_001760	LOC107997640	NW_016019175.1	-	324155	325290	444	Exonic
novel_circ_001761	LOC107997613	NW_016019175.1	+	468922	472274	1802	Exonic
novel_circ_001762	LOC107997579	NW_016019175.1	-	631632	637289	265	Exonic
novel_circ_001763	LOC107997582	NW_016019175.1	-	677613	679746	2013	Exon–intron
novel_circ_001764	LOC107997582	NW_016019175.1	-	677613	682173	3865	Exon–intron
novel_circ_001765	LOC107997582	NW_016019175.1	-	679087	679746	539	Exonic
novel_circ_001766	LOC107997582	NW_016019175.1	-	679087	682173	2391	Exonic
novel_circ_001767	LOC107997586	NW_016019175.1	-	780935	781122	188	Exonic
novel_circ_001768	LOC107997586	NW_016019175.1	-	781531	781692	162	Exonic
novel_circ_001769	LOC107997622	NW_016019175.1	-	796548	797730	973	Exonic
novel_circ_001770	LOC107997602	NW_016019175.1	+	949519	950858	179	Exonic
novel_circ_001771	LOC107997602	NW_016019175.1	+	949519	962743	542	Exonic
novel_circ_001772	LOC107997602	NW_016019175.1	+	949519	980269	2584	Exonic
novel_circ_001773	LOC107997602	NW_016019175.1	+	975381	1010536	3595	Exonic
novel_circ_001774	LOC107997632	NW_016019175.1	-	1064439	1069039	729	Exon–intron
novel_circ_001775	LOC107997643	NW_016019175.1	-	1142566	1145011	745	Exonic
novel_circ_001776	LOC107997643	NW_016019175.1	-	1142566	1162348	1168	Exonic
novel_circ_001777	LOC107997643	NW_016019175.1	-	1161926	1162348	423	Exonic
novel_circ_001778	LOC107997643	NW_016019175.1	-	1161926	1162859	934	Exon–intron
novel_circ_001779	LOC107997643	NW_016019175.1	-	1245318	1256317	1072	Exonic
novel_circ_001780	LOC107997643	NW_016019175.1	-	1256016	1256317	302	Exonic
novel_circ_001781	LOC107997692	NW_016019180.1	-	171	415	245	Exonic
novel_circ_001782	LOC107997744	NW_016019186.1	-	11917	13331	1172	Exonic
novel_circ_001783	LOC107997735	NW_016019186.1	+	24283	25658	366	Exon–intron
novel_circ_001784	LOC114577528	NW_016019186.1	+	348366	349141	776	Exon–intron
novel_circ_001785	LOC107997746	NW_016019186.1	-	574600	574944	267	Exonic
novel_circ_001786	LOC107997729	NW_016019186.1	-	692730	696756	4027	Intronic
novel_circ_001787	LOC107997728	NW_016019186.1	+	717285	717922	555	Exonic
novel_circ_001788	LOC107997699	NW_016019186.1	+	745963	746131	169	Exonic
novel_circ_001789	LOC107997705	NW_016019186.1	+	772857	773011	155	Antisense
novel_circ_001790	LOC107997743	NW_016019186.1	-	1226594	1241126	1320	Exonic
novel_circ_001791	LOC107997743	NW_016019186.1	-	1237298	1237975	351	Exonic
novel_circ_001792	LOC107997743	NW_016019186.1	-	1237298	1241126	528	Exonic
novel_circ_001793	LOC107997743	NW_016019186.1	-	1237298	1249424	615	Exonic
novel_circ_001794	LOC107997743	NW_016019186.1	-	1237298	1279239	687	Exonic
novel_circ_001795	LOC107997897	NW_016019197.1	+	57521	59066	998	Exonic
novel_circ_001796	LOC107997910	NW_016019197.1	-	226865	227039	175	Exonic
novel_circ_001797	LOC107997781	NW_016019197.1	+	310310	320442	7145	Exon–intron
novel_circ_001798	LOC107997781	NW_016019197.1	+	312628	314214	1011	Exonic
novel_circ_001799	LOC107997783	NW_016019197.1	-	369005	389709	273	Exonic
novel_circ_001800	LOC107997877	NW_016019197.1	-	519760	520434	539	Exonic
novel_circ_001801	LOC107997877	NW_016019197.1	-	519760	521421	672	Exonic
novel_circ_001802	LOC107997785	NW_016019197.1	+	667137	669316	371	Exonic
novel_circ_001803	LOC107997785	NW_016019197.1	+	669150	675349	443	Exonic
novel_circ_001804	LOC107997785	NW_016019197.1	+	693769	694089	321	Exonic
novel_circ_001805	LOC107997842	NW_016019197.1	-	753430	761671	7706	Exon–intron
novel_circ_001806	LOC107997788	NW_016019197.1	+	776173	778770	2319	Exon–intron
novel_circ_001807	LOC107997812	NW_016019197.1	+	889136	889908	392	Exonic

novel_circ_001808	LOC107997811	NW_016019197.1	-	897681	920436	6192	Exon–intron
novel_circ_001809	LOC107997811	NW_016019197.1	-	899229	916477	16746	Exon–intron
novel_circ_001810	LOC107997811	NW_016019197.1	-	900774	916645	15369	Exon–intron
novel_circ_001811	LOC107997811	NW_016019197.1	-	901785	916645	14358	Exon–intron
novel_circ_001812	LOC107997811	NW_016019197.1	-	901852	916645	14291	Exon–intron
novel_circ_001813	LOC107997811	NW_016019197.1	-	902077	916645	14066	Exon–intron
novel_circ_001814	LOC107997811	NW_016019197.1	-	903545	916645	12598	Exon–intron
novel_circ_001815	LOC107997811	NW_016019197.1	-	903952	916645	12191	Exon–intron
novel_circ_001816	LOC107997811	NW_016019197.1	-	904571	916645	11572	Exon–intron
novel_circ_001817	LOC107997811	NW_016019197.1	-	914021	916645	2122	Exonic
novel_circ_001818	LOC107997811	NW_016019197.1	-	914021	928626	3130	Exonic
novel_circ_001819	LOC107997811	NW_016019197.1	-	914713	915606	735	Exonic
novel_circ_001820	LOC107997811	NW_016019197.1	-	914713	916645	1505	Exonic
novel_circ_001821	LOC107997811	NW_016019197.1	-	915260	916645	1117	Exonic
novel_circ_001822	LOC107997811	NW_016019197.1	-	915814	916645	770	Exonic
novel_circ_001823	LOC107997811	NW_016019197.1	-	926193	928626	1008	Exonic
novel_circ_001824	LOC107997811	NW_016019197.1	-	927635	928626	753	Exonic
novel_circ_001825	LOC107997905	NW_016019197.1	+	1109256	1109917	408	Exonic
novel_circ_001826	LOC107997823	NW_016019197.1	-	1336036	1355962	4555	Exon–intron
novel_circ_001827	LOC107997823	NW_016019197.1	-	1336036	1356187	4644	Exon–intron
novel_circ_001828	LOC107997823	NW_016019197.1	-	1353341	1356187	2711	Exon–intron
novel_circ_001829	LOC107997823	NW_016019197.1	-	1355670	1356187	382	Exonic
novel_circ_001830	LOC107997932	NW_016019198.1	+	1682	2201	375	Exonic
novel_circ_001831	LOC107997932	NW_016019198.1	+	1682	3488	1435	Exonic
novel_circ_001832	LOC107997932	NW_016019198.1	+	1682	5050	2997	Exon–intron
novel_circ_001833	LOC107997932	NW_016019198.1	+	1682	5327	3274	Exon–intron
novel_circ_001834	LOC107997935	NW_016019205.1	+	10823	11868	887	Exonic
novel_circ_001835	LOC114577552	NW_016019206.1	+	742	858	117	Antisense
novel_circ_001836	LOC107998004	NW_016019208.1	+	180644	181245	391	Exonic
novel_circ_001837	LOC107998004	NW_016019208.1	-	181627	183049	1423	Antisense
novel_circ_001838	LOC107997992	NW_016019208.1	-	778082	780422	1475	Exonic
novel_circ_001839	LOC107997979	NW_016019208.1	+	799939	800628	424	Exonic
novel_circ_001840	LOC107997953	NW_016019208.1	-	1133155	1135195	1145	Exon–intron
novel_circ_001841	LOC107998017	NW_016019208.1	-	1144706	1146674	1293	Exonic
novel_circ_001842	LOC107998009	NW_016019208.1	-	1402432	1403117	270	Exonic
novel_circ_001843	LOC107998050	NW_016019209.1	+	10349	12455	835	Exonic
novel_circ_001844	LOC107998249	NW_016019219.1	-	627	1946	1313	Exonic
novel_circ_001845	LOC107998249	NW_016019219.1	-	26046	32448	634	Exonic
novel_circ_001846	LOC107998249	NW_016019219.1	-	26046	38475	6661	Exon–intron
novel_circ_001847	LOC107998249	NW_016019219.1	-	32013	32448	436	Exonic
novel_circ_001848	LOC107998053	NW_016019219.1	-	79688	80337	402	Exonic
novel_circ_001849	LOC107998118	NW_016019219.1	+	613409	616558	3150	Antisense
novel_circ_001850	LOC107998118	NW_016019219.1	-	617963	619130	986	Exonic
novel_circ_001851	LOC107998118	NW_016019219.1	-	623027	625093	1281	Exonic
novel_circ_001852	LOC107998118	NW_016019219.1	-	624029	625093	837	Exonic
novel_circ_001853	LOC107998187	NW_016019219.1	-	637014	638255	993	Exonic
novel_circ_001854	LOC107998096	NW_016019219.1	+	697294	697767	375	Exonic
novel_circ_001855	LOC107998096	NW_016019219.1	+	822790	825205	563	Exonic
novel_circ_001856	LOC107998096	NW_016019219.1	+	822790	825589	690	Exonic

novel_circ_001857	LOC107998190	NW_016019219.1	-	906629	907065	437	Exonic
novel_circ_001858	LOC107998058	NW_016019219.1	+	1168932	1169984	578	Exonic
novel_circ_001859	LOC107998058	NW_016019219.1	+	1183770	1185372	259	Exonic
novel_circ_001860	LOC107998151	NW_016019219.1	-	1373169	1375374	461	Exonic
novel_circ_001861	LOC107998151	NW_016019219.1	-	1383695	1386144	334	Exonic
novel_circ_001862	LOC107998151	NW_016019219.1	-	1426696	1427924	669	Exonic
novel_circ_001863	LOC107998144	NW_016019219.1	-	1787103	1788306	920	Exonic
novel_circ_001864	LOC107998223	NW_016019219.1	-	1829151	1829849	529	Exonic
novel_circ_001865	LOC107998137	NW_016019219.1	-	2020734	2021630	689	Exonic
novel_circ_001866	LOC107998137	NW_016019219.1	+	2021657	2022460	804	Antisense
novel_circ_001867	LOC107998159	NW_016019219.1	+	2109395	2110176	626	Exonic
novel_circ_001868	LOC107998159	NW_016019219.1	-	2112786	2113884	1099	Antisense
novel_circ_001869	LOC107998159	NW_016019219.1	+	2112786	2113884	726	Exonic
novel_circ_001870	LOC107998159	NW_016019219.1	+	2115045	2115398	354	Exonic
novel_circ_001871	LOC107998103	NW_016019219.1	+	2222253	2224525	2273	Intronic
novel_circ_001872	LOC107998101	NW_016019219.1	-	2386806	2396814	1538	Exonic
novel_circ_001873	LOC107998101	NW_016019219.1	-	2386806	2398613	2480	Exonic
novel_circ_001874	LOC107998101	NW_016019219.1	-	2397387	2398613	1227	Exon–intron
novel_circ_001875	LOC107998101	NW_016019219.1	-	2397672	2398613	942	Exonic
novel_circ_001876	LOC107998101	NW_016019219.1	-	2462944	2465109	2166	Exon–intron
novel_circ_001877	LOC107998112	NW_016019219.1	+	2798130	2798542	264	Exonic
novel_circ_001878	LOC107998112	NW_016019219.1	+	2798130	2800904	615	Exonic
novel_circ_001879	LOC107998112	NW_016019219.1	+	2930778	2934574	3797	Intronic
novel_circ_001880	LOC107998112	NW_016019219.1	+	2968345	2980812	831	Exonic
novel_circ_001881	LOC107998112	NW_016019219.1	+	2975995	2980812	630	Exonic
novel_circ_001882	LOC107998112	NW_016019219.1	+	2975995	2984501	787	Exonic
novel_circ_001883	LOC107998112	NW_016019219.1	+	2980667	2984501	303	Exonic
novel_circ_001884	LOC107998112	NW_016019219.1	+	2980670	2984501	300	Exonic
novel_circ_001885	NA	NW_016019219.1	-	3178202	3178369	168	Intergenic region
novel_circ_001886	LOC107998154	NW_016019219.1	-	3186046	3186609	564	Exonic
novel_circ_001887	LOC107998182	NW_016019219.1	-	3393086	3430225	1779	Exonic
novel_circ_001888	LOC107998182	NW_016019219.1	-	3414832	3416643	482	Exonic
novel_circ_001889	LOC107998182	NW_016019219.1	-	3415936	3416643	278	Exonic
novel_circ_001890	LOC107998163	NW_016019219.1	+	3594162	3595968	1015	Exonic
novel_circ_001891	LOC107998121	NW_016019219.1	+	3669255	3670080	532	Exonic
novel_circ_001892	LOC107998066	NW_016019219.1	+	3728615	3733782	4101	Exonic
novel_circ_001893	LOC107998201	NW_016019219.1	+	3853067	3855075	664	Exonic
novel_circ_001894	LOC107998201	NW_016019219.1	+	3853067	3856472	1152	Exonic
novel_circ_001895	LOC107998191	NW_016019219.1	-	3910251	3911917	1172	Exonic
novel_circ_001896	LOC107998176	NW_016019219.1	+	4243198	4247163	1460	Exonic
novel_circ_001897	LOC107998199	NW_016019219.1	-	4286101	4290573	1280	Exonic
novel_circ_001898	LOC107998104	NW_016019219.1	-	4415402	4415561	160	Exonic
novel_circ_001899	LOC107998104	NW_016019219.1	-	4536416	4538765	468	Exonic
novel_circ_001900	LOC114577573	NW_016019219.1	+	4765837	4805967	36615	Exon–intron
novel_circ_001901	LOC107998119	NW_016019219.1	+	4957249	4959740	1025	Exonic
novel_circ_001902	LOC107998119	NW_016019219.1	+	4960260	4962123	1775	Exonic
novel_circ_001903	LOC107998119	NW_016019219.1	+	4960260	4962877	1900	Exonic
novel_circ_001904	NA	NW_016019219.1	+	4982690	4983321	632	Intergenic region
novel_circ_001905	NA	NW_016019219.1	+	4982690	4984042	1353	Intergenic region

novel_circ_001906	NA	NW_016019219.1	+	4982690	4988380	5691	Intergenic region
novel_circ_001907	NA	NW_016019219.1	+	4982690	5053232	70543	Intergenic region
novel_circ_001908	LOC114577608	NW_016019220.1	+	113422	113678	257	Exonic
novel_circ_001909	LOC107998320	NW_016019220.1	-	315824	316269	446	Exonic
novel_circ_001910	LOC107998319	NW_016019220.1	+	551117	554219	442	Exonic
novel_circ_001911	LOC107998319	NW_016019220.1	+	564700	565147	448	Exonic
novel_circ_001912	LOC107998302	NW_016019220.1	-	763960	767165	2571	Exonic
novel_circ_001913	LOC107998287	NW_016019220.1	+	1003409	1004274	866	Exonic
novel_circ_001914	LOC107998328	NW_016019220.1	-	1047245	1049785	2541	Antisense
novel_circ_001915	LOC107998308	NW_016019220.1	-	1215011	1217766	445	Exonic
novel_circ_001916	LOC107998284	NW_016019220.1	+	1262862	1263089	228	Exonic
novel_circ_001917	LOC107998284	NW_016019220.1	+	1263037	1263264	228	Exonic
novel_circ_001918	LOC107998284	NW_016019220.1	+	1263145	1263372	228	Exonic
novel_circ_001919	LOC107998284	NW_016019220.1	+	1263145	1263600	456	Exonic
novel_circ_001920	LOC107998284	NW_016019220.1	+	1263145	1264284	1140	Exonic
novel_circ_001921	LOC107998284	NW_016019220.1	+	1263206	1263889	684	Exonic
novel_circ_001922	LOC107998284	NW_016019220.1	-	1263206	1263889	684	Antisense
novel_circ_001923	LOC107998284	NW_016019220.1	+	1263218	1263673	456	Exonic
novel_circ_001924	LOC107998284	NW_016019220.1	+	1263373	1264284	912	Exonic
novel_circ_001925	LOC107998284	NW_016019220.1	+	1263493	1263720	228	Exonic
novel_circ_001926	LOC107998284	NW_016019220.1	+	1263546	1263773	228	Exonic
novel_circ_001927	LOC107998284	NW_016019220.1	-	1263630	1264085	456	Antisense
novel_circ_001928	LOC107998284	NW_016019220.1	+	1263630	1264085	456	Exonic
novel_circ_001929	LOC107998284	NW_016019220.1	-	1263630	1264313	684	Antisense
novel_circ_001930	LOC107998284	NW_016019220.1	+	1263638	1264093	456	Exonic
novel_circ_001931	LOC107998284	NW_016019220.1	-	1263638	1264093	456	Antisense
novel_circ_001932	LOC107998284	NW_016019220.1	-	1263638	1264321	684	Antisense
novel_circ_001933	LOC107998284	NW_016019220.1	+	1263721	1263948	228	Exonic
novel_circ_001934	LOC107998284	NW_016019220.1	+	1263774	1264001	228	Exonic
novel_circ_001935	LOC107998284	NW_016019220.1	-	1263858	1264085	228	Antisense
novel_circ_001936	LOC107998284	NW_016019220.1	+	1263949	1264176	228	Exonic
novel_circ_001937	LOC107998284	NW_016019220.1	+	1263954	1264181	228	Exonic
novel_circ_001938	LOC107998284	NW_016019220.1	+	1264057	1264284	228	Exonic
novel_circ_001939	LOC107998283	NW_016019220.1	-	1425825	1462893	4931	Exon–intron
novel_circ_001940	LOC107998283	NW_016019220.1	-	1425883	1462893	4873	Exon–intron
novel_circ_001941	LOC107998283	NW_016019220.1	-	1429255	1452752	1298	Exonic
novel_circ_001942	LOC107998283	NW_016019220.1	-	1449367	1462893	2178	Exonic
novel_circ_001943	LOC107998335	NW_016019221.1	-	2737	11803	6615	Exon–intron
novel_circ_001944	LOC107998399	NW_016019231.1	-	18849	20273	645	Exonic
novel_circ_001945	LOC107998379	NW_016019231.1	+	535381	536120	740	Exonic
novel_circ_001946	LOC107998379	NW_016019231.1	+	597175	624764	27590	Exon–intron
novel_circ_001947	LOC107998379	NW_016019231.1	+	624599	624764	166	Exonic
novel_circ_001948	LOC107998379	NW_016019231.1	+	727481	728960	1480	Intronic
novel_circ_001949	LOC107998379	NW_016019231.1	+	746692	746989	298	Exonic
novel_circ_001950	LOC107998379	NW_016019231.1	+	746692	747194	503	Exon–intron
novel_circ_001951	LOC107998379	NW_016019231.1	+	746692	750549	510	Exonic
novel_circ_001952	LOC107998375	NW_016019231.1	+	1124208	1126018	836	Exonic
novel_circ_001953	LOC107998375	NW_016019231.1	+	1124208	1127807	978	Exonic
novel_circ_001954	LOC107998401	NW_016019231.1	+	1178818	1179425	398	Exonic

novel_circ_001955	LOC107998401	NW_016019231.1	+	1178924	1179425	292	Exonic
novel_circ_001956	LOC107998401	NW_016019231.1	+	1183061	1184066	589	Exonic
novel_circ_001957	LOC107998364	NW_016019231.1	+	1206440	1207536	382	Exonic
novel_circ_001958	LOC107998347	NW_016019231.1	+	1269002	1269322	321	Exonic
novel_circ_001959	LOC107998347	NW_016019231.1	-	1269123	1269311	189	Antisense
novel_circ_001960	LOC107998347	NW_016019231.1	-	1269123	1269401	279	Antisense
novel_circ_001961	LOC107998347	NW_016019231.1	-	1269141	1269329	189	Antisense
novel_circ_001962	LOC107998347	NW_016019231.1	-	1269141	1269419	279	Antisense
novel_circ_001963	LOC107998347	NW_016019231.1	-	1269159	1269257	99	Antisense
novel_circ_001964	LOC107998347	NW_016019231.1	-	1269159	1269347	189	Antisense
novel_circ_001965	LOC107998347	NW_016019231.1	-	1269204	1269482	279	Antisense
novel_circ_001966	LOC107998347	NW_016019231.1	-	1269240	1269419	180	Antisense
novel_circ_001967	LOC107998347	NW_016019231.1	-	1269240	1269518	279	Antisense
novel_circ_001968	LOC107998347	NW_016019231.1	-	1269258	1269437	180	Antisense
novel_circ_001969	LOC107998347	NW_016019231.1	-	1269267	1269446	180	Antisense
novel_circ_001970	LOC107998347	NW_016019231.1	-	1269285	1269374	90	Antisense
novel_circ_001971	LOC107998347	NW_016019231.1	-	1269285	1269482	198	Antisense
novel_circ_001972	LOC107998347	NW_016019231.1	-	1269330	1269518	189	Antisense
novel_circ_001973	LOC107998347	NW_016019231.1	+	1274306	1274422	117	Exonic
novel_circ_001974	LOC107998347	NW_016019231.1	-	1274306	1274422	117	Antisense
novel_circ_001975	LOC107998347	NW_016019231.1	-	1274315	1274431	117	Antisense
novel_circ_001976	LOC107998371	NW_016019231.1	+	1364398	1365944	628	Exonic
novel_circ_001977	LOC107998371	NW_016019231.1	+	1365675	1377824	1301	Exonic
novel_circ_001978	LOC107998371	NW_016019231.1	+	1377260	1378676	924	Exonic
novel_circ_001979	LOC107998372	NW_016019231.1	-	1386894	1387300	407	Antisense
novel_circ_001980	LOC107998372	NW_016019231.1	-	1387657	1388326	670	Antisense
novel_circ_001981	LOC107998407	NW_016019233.1	-	2451	4371	1563	Exonic
novel_circ_001982	LOC107998464	NW_016019242.1	+	23851	25845	383	Exonic
novel_circ_001983	LOC107998457	NW_016019242.1	-	68663	69794	388	Exonic
novel_circ_001984	LOC107998457	NW_016019242.1	-	68663	70157	751	Exonic
novel_circ_001985	LOC107998414	NW_016019242.1	+	143075	143815	366	Exonic
novel_circ_001986	LOC107998414	NW_016019242.1	+	143075	147648	735	Exonic
novel_circ_001987	LOC107998414	NW_016019242.1	+	143075	162133	835	Exonic
novel_circ_001988	LOC107998414	NW_016019242.1	+	143075	233776	14640	Exon–intron
novel_circ_001989	LOC107998414	NW_016019242.1	+	231252	233776	2525	Exon–intron
novel_circ_001990	LOC107998431	NW_016019242.1	-	246937	247265	329	Antisense
novel_circ_001991	LOC107998431	NW_016019242.1	+	321803	322488	612	Exonic
novel_circ_001992	LOC107998431	NW_016019242.1	-	324221	325425	1205	Antisense
novel_circ_001993	LOC107998433	NW_016019242.1	-	367371	367689	319	Antisense
novel_circ_001994	LOC107998439	NW_016019242.1	+	846171	850847	1684	Exonic
novel_circ_001995	NA	NW_016019242.1	+	891206	892265	1060	Intergenic region
novel_circ_001996	LOC107998447	NW_016019242.1	-	897076	900606	2655	Exonic
novel_circ_001997	LOC107998447	NW_016019242.1	-	897967	900606	1764	Exonic
novel_circ_001998	LOC107998447	NW_016019242.1	-	899370	900606	944	Exonic
novel_circ_001999	LOC107998470	NW_016019242.1	+	1250518	1256720	276	Exonic
novel_circ_002000	LOC107998557	NW_016019253.1	+	193725	195007	1126	Exon–intron
novel_circ_002001	LOC107998540	NW_016019253.1	-	238701	239862	837	Exonic
novel_circ_002002	LOC107998540	NW_016019253.1	-	238701	241019	1326	Exonic
novel_circ_002003	LOC107998565	NW_016019253.1	+	420112	422094	1983	Exon–intron

novel_circ_002004	LOC107998565	NW_016019253.1	-	426198	429955	3758	Antisense
novel_circ_002005	LOC107998560	NW_016019253.1	+	470253	471217	174	Exonic
novel_circ_002006	LOC107998560	NW_016019253.1	+	470253	474998	338	Exonic
novel_circ_002007	LOC107998561	NW_016019253.1	-	478948	487534	8090	Exon–intron
novel_circ_002008	LOC107998561	NW_016019253.1	-	479018	487534	8020	Exon–intron
novel_circ_002009	LOC107998561	NW_016019253.1	-	486082	487534	956	Exonic
novel_circ_002010	LOC107998596	NW_016019253.1	+	616041	617867	667	Exonic
novel_circ_002011	LOC107998502	NW_016019253.1	-	817676	819563	1223	Exonic
novel_circ_002012	LOC107998502	NW_016019253.1	-	817941	819563	1028	Exonic
novel_circ_002013	LOC107998496	NW_016019253.1	+	874203	879119	4681	Exon–intron
novel_circ_002014	LOC107998515	NW_016019253.1	-	1034038	1038837	4800	Antisense
novel_circ_002015	LOC107998510	NW_016019253.1	-	1064525	1073567	6393	Exonic
novel_circ_002016	LOC107998510	NW_016019253.1	-	1064525	1087384	8735	Exonic
novel_circ_002017	LOC107998510	NW_016019253.1	-	1082226	1087384	2342	Exonic
novel_circ_002018	LOC107998516	NW_016019253.1	-	1117084	1120509	566	Exonic
novel_circ_002019	LOC107998516	NW_016019253.1	-	1118031	1120509	459	Exonic
novel_circ_002020	LOC107998512	NW_016019253.1	+	1220112	1228625	8159	Exon–intron
novel_circ_002021	LOC107998512	NW_016019253.1	+	1220112	1228765	8299	Exon–intron
novel_circ_002022	LOC107998548	NW_016019253.1	+	1244196	1246184	328	Exonic
novel_circ_002023	LOC107998548	NW_016019253.1	+	1244196	1260484	795	Exonic
novel_circ_002024	LOC107998548	NW_016019253.1	+	1257689	1260484	467	Exonic
novel_circ_002025	LOC107998548	NW_016019253.1	+	1259249	1260484	363	Exonic
novel_circ_002026	LOC107998559	NW_016019253.1	-	1328635	1329824	15	Exonic
novel_circ_002027	LOC114577641	NW_016019253.1	+	1340654	1341677	490	Exonic
novel_circ_002028	LOC107998618	NW_016019264.1	-	889865	893087	2407	Exonic
novel_circ_002029	LOC107998609	NW_016019264.1	+	1167583	1173623	2308	Exon–intron
novel_circ_002030	LOC107998626	NW_016019264.1	+	1207761	1209660	318	Exonic
novel_circ_002031	LOC107998634	NW_016019266.1	+	4424	6617	1541	Exonic
novel_circ_002032	LOC107998727	NW_016019275.1	+	206596	207239	644	Exonic
novel_circ_002033	LOC107998668	NW_016019275.1	-	420751	421808	1058	Exonic
novel_circ_002034	LOC107998671	NW_016019275.1	+	469590	470518	854	Exon–intron
novel_circ_002035	LOC107998746	NW_016019275.1	+	546704	549975	201	Exonic
novel_circ_002036	LOC107998746	NW_016019275.1	+	546704	559754	873	Exonic
novel_circ_002037	LOC107998746	NW_016019275.1	+	549900	558185	474	Exonic
novel_circ_002038	LOC107998746	NW_016019275.1	+	557607	558185	398	Exonic
novel_circ_002039	LOC107998746	NW_016019275.1	+	557607	559754	672	Exonic
novel_circ_002040	LOC107998700	NW_016019275.1	-	584270	584741	388	Exonic
novel_circ_002041	LOC107998700	NW_016019275.1	-	584318	584741	340	Exonic
novel_circ_002042	LOC107998647	NW_016019275.1	-	781426	784179	117	Exonic
novel_circ_002043	LOC107998710	NW_016019275.1	-	838717	842085	303	Exonic
novel_circ_002044	LOC107998728	NW_016019275.1	-	947067	948039	819	Exonic
novel_circ_002045	LOC107998728	NW_016019275.1	-	947067	948758	1267	Exonic
novel_circ_002046	LOC107998680	NW_016019275.1	+	968613	969249	637	Antisense
novel_circ_002047	LOC107998683	NW_016019275.1	+	992235	992638	404	Exonic
novel_circ_002048	LOC107998683	NW_016019275.1	+	992235	994297	535	Exonic
novel_circ_002049	LOC107998655	NW_016019275.1	-	1114956	1115968	426	Exonic
novel_circ_002050	LOC107998655	NW_016019275.1	-	1114956	1155614	632	Exonic
novel_circ_002051	LOC107998693	NW_016019275.1	+	1217451	1217938	488	Exonic
novel_circ_002052	NA	NW_016019275.1	-	1223359	1224215	857	Intergenic region

novel_circ_002053	LOC107998747	NW_016019275.1	-	1297674	1301303	332	Exonic
novel_circ_002054	LOC107998742	NW_016019275.1	-	1330959	1341101	10143	Exon–intron
novel_circ_002055	LOC107998835	NW_016019286.1	+	12456	12654	199	Intronic
novel_circ_002056	LOC114577681	NW_016019286.1	-	396276	396651	376	Exon–intron
novel_circ_002057	LOC114577681	NW_016019286.1	-	409919	410248	330	Exonic
novel_circ_002058	LOC114577681	NW_016019286.1	-	410050	410234	185	Exonic
novel_circ_002059	LOC107998821	NW_016019286.1	-	467150	467942	540	Exonic
novel_circ_002060	LOC107998821	NW_016019286.1	-	467150	468226	738	Exonic
novel_circ_002061	LOC107998795	NW_016019286.1	+	552032	561894	1739	Exonic
novel_circ_002062	LOC107998795	NW_016019286.1	+	559016	559474	324	Exonic
novel_circ_002063	LOC107998795	NW_016019286.1	+	559016	561894	1514	Exonic
novel_circ_002064	LOC107998838	NW_016019286.1	+	649610	650883	734	Exonic
novel_circ_002065	LOC107998838	NW_016019286.1	+	649610	651700	1176	Exonic
novel_circ_002066	LOC107998819	NW_016019286.1	+	879435	879870	436	Exonic
novel_circ_002067	LOC107998779	NW_016019286.1	-	890534	892098	1402	Exonic
novel_circ_002068	LOC107998779	NW_016019286.1	-	891071	892098	963	Exonic
novel_circ_002069	LOC107998814	NW_016019286.1	-	894840	920078	24698	Exon–intron
novel_circ_002070	LOC107998815	NW_016019286.1	-	919004	920078	1075	Exonic
novel_circ_002071	LOC107998781	NW_016019286.1	+	968566	969515	506	Exonic
novel_circ_002072	LOC107998781	NW_016019286.1	+	968566	969752	743	Exonic
novel_circ_002073	LOC107998789	NW_016019286.1	-	1016425	1016590	166	Exonic
novel_circ_002074	LOC107998782	NW_016019286.1	+	1159029	1159204	176	Exonic
novel_circ_002075	LOC107998782	NW_016019286.1	+	1161060	1161664	394	Exonic
novel_circ_002076	LOC107998782	NW_016019286.1	+	1161060	1162845	986	Exonic
novel_circ_002077	LOC107998782	NW_016019286.1	+	1162254	1162845	592	Exonic
novel_circ_002078	LOC107998930	NW_016019297.1	+	27949	28331	383	Exonic
novel_circ_002079	LOC107998930	NW_016019297.1	+	141184	141422	239	Exonic
novel_circ_002080	LOC107998930	NW_016019297.1	+	141184	145940	316	Exonic
novel_circ_002081	LOC107998930	NW_016019297.1	+	141184	146547	406	Exonic
novel_circ_002082	LOC107998930	NW_016019297.1	+	141184	149531	596	Exonic
novel_circ_002083	LOC107998877	NW_016019297.1	-	309686	320580	6978	Exonic
novel_circ_002084	LOC107998877	NW_016019297.1	-	309763	320580	9388	Exon–intron
novel_circ_002085	LOC107998877	NW_016019297.1	-	312214	330172	11811	Exon–intron
novel_circ_002086	LOC107998877	NW_016019297.1	-	312220	330172	11805	Exon–intron
novel_circ_002087	LOC107998974	NW_016019297.1	-	431683	432366	418	Exonic
novel_circ_002088	LOC107998923	NW_016019297.1	-	562346	563548	912	Exonic
novel_circ_002089	LOC107998920	NW_016019297.1	-	703462	704395	750	Exonic
novel_circ_002090	LOC107998920	NW_016019297.1	-	703997	705083	610	Exonic
novel_circ_002091	LOC107998938	NW_016019297.1	+	768479	769089	611	Exonic
novel_circ_002092	LOC107998975	NW_016019297.1	-	1026420	1030192	1639	Exonic
novel_circ_002093	LOC107998975	NW_016019297.1	-	1026523	1030192	1536	Exonic
novel_circ_002094	LOC107999010	NW_016019297.1	+	1037897	1039227	717	Exonic
novel_circ_002095	LOC107998916	NW_016019297.1	+	1103823	1104673	851	Exonic
novel_circ_002096	LOC107999021	NW_016019301.1	+	5111	7413	1573	Exonic
novel_circ_002097	LOC107999060	NW_016019308.1	-	342165	342550	267	Exonic
novel_circ_002098	LOC107999051	NW_016019308.1	+	427355	429762	2217	Exonic
novel_circ_002099	LOC107999052	NW_016019308.1	+	456937	458938	1139	Exonic
novel_circ_002100	LOC107999048	NW_016019308.1	+	844333	847577	956	Exonic
novel_circ_002101	LOC107999048	NW_016019308.1	+	846743	847577	495	Exonic

novel_circ_002102	LOC107999044	NW_016019308.1	+	901325	902475	1151	Antisense
novel_circ_002103	LOC107999044	NW_016019308.1	+	901909	902475	567	Antisense
novel_circ_002104	LOC107999044	NW_016019308.1	-	1087656	1123051	353	Exonic
novel_circ_002105	LOC107999033	NW_016019308.1	-	1158850	1160546	704	Exonic
novel_circ_002106	LOC107999033	NW_016019308.1	-	1159599	1160546	606	Exonic
novel_circ_002107	LOC107999081	NW_016019310.1	+	4243	5393	968	Exonic
novel_circ_002108	LOC107999103	NW_016019319.1	+	107383	108368	325	Exonic
novel_circ_002109	LOC107999180	NW_016019319.1	-	160504	161368	865	Antisense
novel_circ_002110	LOC107999180	NW_016019319.1	-	161346	162782	1437	Antisense
novel_circ_002111	LOC107999167	NW_016019319.1	-	196031	197309	672	Exonic
novel_circ_002112	NA	NW_016019319.1	-	336447	336664	218	Intergenic region
novel_circ_002113	LOC107999106	NW_016019319.1	-	369054	369391	338	Antisense
novel_circ_002114	LOC107999143	NW_016019319.1	-	484570	485849	575	Exonic
novel_circ_002115	LOC107999327	NW_016019330.1	+	42096	42381	286	Exonic
novel_circ_002116	LOC107999210	NW_016019330.1	+	246745	257009	7737	Exon–intron
novel_circ_002117	LOC107999212	NW_016019330.1	+	432885	439638	6680	Exon–intron
novel_circ_002118	LOC107999443	NW_016019330.1	-	718193	718674	482	Exonic
novel_circ_002119	LOC107999339	NW_016019330.1	+	907562	910676	834	Exonic
novel_circ_002120	LOC107999390	NW_016019330.1	+	1036197	1036415	219	Exonic
novel_circ_002121	LOC107999330	NW_016019330.1	+	1373689	1374092	404	Antisense
novel_circ_002122	LOC107999423	NW_016019330.1	+	1442571	1445983	2638	Exonic
novel_circ_002123	LOC107999220	NW_016019330.1	+	1533671	1534225	555	Exonic
novel_circ_002124	LOC107999220	NW_016019330.1	+	1533671	1537860	691	Exonic
novel_circ_002125	LOC107999315	NW_016019330.1	+	1602331	1603242	340	Exonic
novel_circ_002126	LOC107999315	NW_016019330.1	+	1602331	1610135	2440	Exonic
novel_circ_002127	LOC107999391	NW_016019330.1	+	1658491	1663711	364	Exonic
novel_circ_002128	LOC107999336	NW_016019330.1	-	1673402	1698820	11633	Exon–intron
novel_circ_002129	LOC107999336	NW_016019330.1	-	1673402	1698849	11662	Exon–intron
novel_circ_002130	LOC107999336	NW_016019330.1	-	1674015	1698820	11020	Exon–intron
novel_circ_002131	LOC107999336	NW_016019330.1	-	1674015	1698849	11049	Exon–intron
novel_circ_002132	LOC107999336	NW_016019330.1	-	1683769	1698849	1376	Exonic
novel_circ_002133	LOC107999421	NW_016019330.1	+	1706814	1708252	1439	Antisense
novel_circ_002134	LOC107999388	NW_016019330.1	-	1742113	1745342	2782	Exonic
novel_circ_002135	LOC107999247	NW_016019330.1	-	1758065	1758684	425	Exonic
novel_circ_002136	LOC107999222	NW_016019330.1	-	1800638	1821321	896	Exonic
novel_circ_002137	LOC107999222	NW_016019330.1	-	1810147	1811166	456	Exonic
novel_circ_002138	LOC107999465	NW_016019330.1	+	2031238	2031635	398	Antisense
novel_circ_002139	LOC107999224	NW_016019330.1	+	2444519	2445139	348	Exonic
novel_circ_002140	LOC107999224	NW_016019330.1	+	2489847	2491359	439	Exonic
novel_circ_002141	LOC107999418	NW_016019330.1	+	2679305	2680240	299	Exonic
novel_circ_002142	LOC107999225	NW_016019330.1	+	2742248	2743042	300	Exonic
novel_circ_002143	LOC107999333	NW_016019330.1	-	2830496	2830759	264	Exonic
novel_circ_002144	LOC107999303	NW_016019330.1	-	2903741	2906152	2412	Antisense
novel_circ_002145	LOC107999303	NW_016019330.1	-	2905764	2906841	1078	Antisense
novel_circ_002146	LOC107999303	NW_016019330.1	+	2906628	2906939	312	Exonic
novel_circ_002147	LOC107999303	NW_016019330.1	-	2906628	2906939	312	Antisense
novel_circ_002148	LOC107999471	NW_016019330.1	+	3054891	3057200	550	Exonic
novel_circ_002149	LOC107999359	NW_016019330.1	+	3061145	3062113	446	Exonic
novel_circ_002150	LOC107999359	NW_016019330.1	+	3062902	3063480	344	Exonic

novel_circ_002151	LOC107999358	NW_016019330.1	+	3077189	3079830	888	Exonic
novel_circ_002152	LOC107999358	NW_016019330.1	+	3077330	3077821	310	Exonic
novel_circ_002153	LOC107999358	NW_016019330.1	+	3077607	3080593	1005	Exonic
novel_circ_002154	LOC107999358	NW_016019330.1	+	3082041	3090614	4302	Exonic
novel_circ_002155	LOC107999358	NW_016019330.1	+	3083164	3084469	870	Exonic
novel_circ_002156	LOC107999358	NW_016019330.1	-	3083259	3083786	528	Antisense
novel_circ_002157	LOC107999358	NW_016019330.1	-	3085411	3086175	765	Antisense
novel_circ_002158	LOC107999358	NW_016019330.1	+	3086017	3087057	698	Exonic
novel_circ_002159	LOC107999358	NW_016019330.1	+	3087749	3087920	172	Exon–intron
novel_circ_002160	LOC107999358	NW_016019330.1	-	3090017	3090169	153	Antisense
novel_circ_002161	LOC107999228	NW_016019330.1	-	3103249	3112107	3657	Exon–intron
novel_circ_002162	LOC107999286	NW_016019330.1	-	3143987	3145777	985	Exonic
novel_circ_002163	LOC107999286	NW_016019330.1	-	3143987	3145820	1028	Exonic
novel_circ_002164	LOC107999286	NW_016019330.1	-	3147699	3148035	337	Exon–intron
novel_circ_002165	LOC107999287	NW_016019330.1	+	3191831	3207371	15541	Antisense
novel_circ_002166	LOC107999287	NW_016019330.1	+	3191831	3207895	16065	Antisense
novel_circ_002167	LOC107999331	NW_016019330.1	-	4253544	4257496	1427	Exonic
novel_circ_002168	LOC107999366	NW_016019330.1	-	4294112	4294673	439	Exonic
novel_circ_002169	NA	NW_016019331.1	+	164935	165277	343	Intergenic region
novel_circ_002170	LOC107999479	NW_016019331.1	+	289555	291016	728	Exonic
novel_circ_002171	LOC107999512	NW_016019331.1	-	402459	407626	1198	Exonic
novel_circ_002172	LOC107999512	NW_016019331.1	-	406318	408650	909	Exonic
novel_circ_002173	LOC107999512	NW_016019331.1	-	407186	407626	328	Exonic
novel_circ_002174	LOC107999512	NW_016019331.1	-	413295	415135	274	Exonic
novel_circ_002175	LOC107999482	NW_016019331.1	-	471375	471722	253	Exonic
novel_circ_002176	LOC107999482	NW_016019331.1	-	472359	473472	291	Exonic
novel_circ_002177	LOC107999499	NW_016019331.1	-	515254	516640	376	Exonic
novel_circ_002178	LOC107999499	NW_016019331.1	-	515254	519944	1528	Exonic
novel_circ_002179	LOC107999499	NW_016019331.1	-	516440	527670	5673	Exon–intron
novel_circ_002180	LOC107999516	NW_016019331.1	-	593429	593841	319	Exonic
novel_circ_002181	LOC107999505	NW_016019331.1	-	964480	966186	1216	Exonic
novel_circ_002182	LOC107999505	NW_016019331.1	-	965691	966186	415	Exonic
novel_circ_002183	LOC107999505	NW_016019331.1	-	966561	968389	793	Exonic
novel_circ_002184	LOC107999505	NW_016019331.1	-	1004815	1007566	698	Exonic
novel_circ_002185	LOC107999505	NW_016019331.1	-	1004815	1010710	831	Exonic
novel_circ_002186	LOC107999505	NW_016019331.1	-	1004815	1023496	2514	Exon–intron
novel_circ_002187	LOC107999505	NW_016019331.1	-	1004815	1023521	2539	Exon–intron
novel_circ_002188	LOC107999525	NW_016019331.1	-	1071851	1074161	1774	Exonic
novel_circ_002189	LOC107999525	NW_016019331.1	-	1140078	1141254	1177	Exonic
novel_circ_002190	LOC107999520	NW_016019331.1	+	1180813	1195250	1157	Exonic
novel_circ_002191	LOC107999520	NW_016019331.1	-	1180813	1195250	14438	Antisense
novel_circ_002192	LOC107999520	NW_016019331.1	+	1180813	1244926	1992	Exonic
novel_circ_002193	LOC107999520	NW_016019331.1	+	1194468	1231174	1350	Exonic
novel_circ_002194	LOC107999520	NW_016019331.1	+	1194468	1237021	1491	Exonic
novel_circ_002195	LOC107999520	NW_016019331.1	+	1194468	1244926	1618	Exonic
novel_circ_002196	LOC107999520	NW_016019331.1	+	1230604	1231174	571	Exon–intron
novel_circ_002197	LOC107999520	NW_016019331.1	+	1230604	1237021	712	Exon–intron
novel_circ_002198	LOC107999520	NW_016019331.1	+	1236881	1244926	268	Exonic
novel_circ_002199	LOC107999597	NW_016019342.1	+	42755	63444	6098	Exon–intron

novel_circ_002200	LOC107999613	NW_016019342.1	-	133917	134881	681	Exonic
novel_circ_002201	LOC107999610	NW_016019342.1	-	165032	165942	706	Exonic
novel_circ_002202	LOC107999599	NW_016019342.1	+	228261	240687	690	Exonic
novel_circ_002203	LOC107999599	NW_016019342.1	+	239952	240687	365	Exonic
novel_circ_002204	LOC107999593	NW_016019342.1	-	356491	362326	2113	Exonic
novel_circ_002205	LOC107999592	NW_016019342.1	-	437114	439056	246	Exonic
novel_circ_002206	LOC107999588	NW_016019342.1	+	625257	637143	8642	Exon–intron
novel_circ_002207	LOC107999607	NW_016019342.1	-	666073	670855	986	Exonic
novel_circ_002208	LOC107999571	NW_016019342.1	+	967510	969070	1235	Exonic
novel_circ_002209	LOC107999571	NW_016019342.1	+	967510	970476	2071	Exonic
novel_circ_002210	LOC107999622	NW_016019347.1	+	6646	7774	582	Exonic
novel_circ_002211	LOC107999670	NW_016019353.1	-	129926	134162	2016	Exonic
novel_circ_002212	LOC107999670	NW_016019353.1	-	132746	133134	328	Exonic
novel_circ_002213	LOC107999670	NW_016019353.1	-	132746	134162	936	Exonic
novel_circ_002214	LOC107999690	NW_016019353.1	+	202451	203176	726	Exonic
novel_circ_002215	LOC107999690	NW_016019353.1	+	202451	205422	1451	Exonic
novel_circ_002216	LOC107999690	NW_016019353.1	+	214808	215926	639	Exonic
novel_circ_002217	LOC107999690	NW_016019353.1	+	237905	238732	828	Intronic
novel_circ_002218	LOC107999690	NW_016019353.1	+	259281	260115	835	Intronic
novel_circ_002219	LOC107999717	NW_016019353.1	-	297717	303177	460	Exonic
novel_circ_002220	LOC107999693	NW_016019353.1	+	365977	367114	462	Exonic
novel_circ_002221	LOC107999704	NW_016019353.1	-	427475	427844	370	Antisense
novel_circ_002222	LOC107999704	NW_016019353.1	+	445039	446628	1590	Intronic
novel_circ_002223	LOC107999704	NW_016019353.1	+	445052	446628	1577	Intronic
novel_circ_002224	LOC107999704	NW_016019353.1	+	470628	472097	242	Exonic
novel_circ_002225	LOC107999704	NW_016019353.1	+	470628	478602	440	Exonic
novel_circ_002226	LOC107999704	NW_016019353.1	+	470628	482479	540	Exonic
novel_circ_002227	LOC107999704	NW_016019353.1	+	470628	483782	740	Exonic
novel_circ_002228	LOC107999704	NW_016019353.1	+	485612	487513	510	Exonic
novel_circ_002229	LOC107999647	NW_016019353.1	+	649490	652881	379	Exonic
novel_circ_002230	LOC107999683	NW_016019353.1	-	794767	797264	1745	Exonic
novel_circ_002231	LOC107999686	NW_016019353.1	-	845134	850470	250	Exonic
novel_circ_002232	LOC107999703	NW_016019353.1	+	1008532	1008703	172	Exonic
novel_circ_002233	LOC114577778	NW_016019353.1	+	1047572	1054829	4502	Exon–intron
novel_circ_002234	LOC107999727	NW_016019353.1	-	1100126	1100281	156	Antisense
novel_circ_002235	LOC107999724	NW_016019353.1	-	1114795	1115170	376	Antisense
novel_circ_002236	LOC107999782	NW_016019364.1	+	39751	42120	883	Exonic
novel_circ_002237	LOC107999742	NW_016019364.1	-	58309	68202	9765	Exon–intron
novel_circ_002238	LOC114577784	NW_016019364.1	+	95853	96976	1124	Exon–intron
novel_circ_002239	LOC107999744	NW_016019364.1	+	166572	167164	295	Exonic
novel_circ_002240	LOC107999744	NW_016019364.1	+	166572	168828	890	Exonic
novel_circ_002241	LOC107999744	NW_016019364.1	+	166572	175733	4036	Exonic
novel_circ_002242	LOC107999744	NW_016019364.1	+	180571	181703	257	Exonic
novel_circ_002243	LOC107999758	NW_016019364.1	+	239271	239936	253	Exonic
novel_circ_002244	LOC107999758	NW_016019364.1	+	239271	242149	1287	Exonic
novel_circ_002245	LOC107999758	NW_016019364.1	+	241958	242350	309	Exonic
novel_circ_002246	LOC107999758	NW_016019364.1	+	244541	245090	454	Exonic
novel_circ_002247	LOC107999758	NW_016019364.1	+	246800	247015	216	Exonic
novel_circ_002248	LOC107999758	NW_016019364.1	+	246800	247804	760	Exonic

novel_circ_002249	LOC107999760	NW_016019364.1	+	273947	300078	976	Exon–intron
novel_circ_002250	LOC107999760	NW_016019364.1	+	299203	302267	1153	Exonic
novel_circ_002251	LOC107999760	NW_016019364.1	+	299203	306876	2841	Exonic
novel_circ_002252	LOC107999760	NW_016019364.1	+	299203	308061	3033	Exonic
novel_circ_002253	LOC107999760	NW_016019364.1	+	299203	328754	16905	Exon–intron
novel_circ_002254	LOC107999760	NW_016019364.1	+	299888	308061	4260	Exon–intron
novel_circ_002255	LOC114577783	NW_016019364.1	+	502096	503193	1098	Exon–intron
novel_circ_002256	LOC107999766	NW_016019364.1	-	616668	617653	391	Exonic
novel_circ_002257	LOC107999768	NW_016019364.1	-	740588	746421	5834	Exon–intron
novel_circ_002258	NA	NW_016019364.1	-	745659	746421	763	Intergenic region
novel_circ_002259	LOC107999764	NW_016019364.1	+	932239	933016	778	Exon–intron
novel_circ_002260	LOC107999827	NW_016019375.1	-	639585	639891	307	Antisense
novel_circ_002261	LOC107999825	NW_016019375.1	-	659113	659325	213	Antisense
novel_circ_002262	LOC107999825	NW_016019375.1	-	659143	659308	166	Antisense
novel_circ_002263	LOC107999825	NW_016019375.1	+	665192	665515	324	Exonic
novel_circ_002264	LOC107999825	NW_016019375.1	-	665195	665865	671	Antisense
novel_circ_002265	LOC107999825	NW_016019375.1	-	665199	665380	182	Antisense
novel_circ_002266	LOC107999825	NW_016019375.1	-	665208	665580	373	Antisense
novel_circ_002267	LOC107999825	NW_016019375.1	+	665494	665676	183	Exonic
novel_circ_002268	LOC107999825	NW_016019375.1	+	665516	665693	178	Exonic
novel_circ_002269	LOC107999825	NW_016019375.1	+	665698	665856	159	Exonic
novel_circ_002270	LOC107999825	NW_016019375.1	-	665733	665930	198	Antisense
novel_circ_002271	LOC107999825	NW_016019375.1	+	665943	667004	1062	Exon–intron
novel_circ_002272	LOC107999825	NW_016019375.1	+	665952	667004	1053	Exon–intron
novel_circ_002273	LOC107999825	NW_016019375.1	+	666015	667004	990	Exon–intron
novel_circ_002274	LOC107999825	NW_016019375.1	+	666054	667004	951	Exon–intron
novel_circ_002275	LOC107999817	NW_016019375.1	-	687908	691683	3776	Exon–intron
novel_circ_002276	LOC107999813	NW_016019375.1	-	1050853	1051966	357	Exonic
novel_circ_002277	LOC107999813	NW_016019375.1	-	1050853	1056960	631	Exonic
novel_circ_002278	LOC107999813	NW_016019375.1	-	1110076	1111717	1168	Exon–intron
novel_circ_002279	LOC114577809	NW_016019386.1	-	292383	298105	4447	Exon–intron
novel_circ_002280	LOC107999834	NW_016019386.1	-	292383	299842	5860	Exon–intron
novel_circ_002281	LOC107999834	NW_016019386.1	-	292383	301572	5945	Exon–intron
novel_circ_002282	LOC114577809	NW_016019386.1	+	293734	294083	350	Antisense
novel_circ_002283	LOC114577809	NW_016019386.1	-	293734	298105	4293	Exon–intron
novel_circ_002284	LOC107999834	NW_016019386.1	-	299706	301572	222	Exonic
novel_circ_002285	LOC107999834	NW_016019386.1	-	371951	372680	559	Exon–intron
novel_circ_002286	LOC107999893	NW_016019386.1	-	393967	394850	388	Exonic
novel_circ_002287	LOC107999893	NW_016019386.1	-	394507	394850	212	Exonic
novel_circ_002288	LOC107999910	NW_016019386.1	-	441993	442926	589	Exonic
novel_circ_002289	LOC107999835	NW_016019386.1	+	470794	471466	368	Exonic
novel_circ_002290	LOC107999904	NW_016019386.1	+	556688	557614	823	Exonic
novel_circ_002291	LOC107999904	NW_016019386.1	+	556688	558848	1621	Exonic
novel_circ_002292	LOC107999904	NW_016019386.1	+	556688	562877	2690	Exonic
novel_circ_002293	LOC107999904	NW_016019386.1	+	561550	562877	985	Exonic
novel_circ_002294	LOC107999871	NW_016019386.1	+	586184	586454	195	Exonic
novel_circ_002295	LOC107999871	NW_016019386.1	+	586184	586461	202	Exonic
novel_circ_002296	LOC107999871	NW_016019386.1	+	586184	586463	204	Exon–intron
novel_circ_002297	LOC107999877	NW_016019386.1	+	946593	947498	291	Exonic

novel_circ_002298	LOC107999877	NW_016019386.1	+	991991	1021681	18180	Exon–intron
novel_circ_002299	LOC107999877	NW_016019386.1	+	1001097	1012395	8325	Exon–intron
novel_circ_002300	LOC107999877	NW_016019386.1	+	1001097	1021229	8622	Exon–intron
novel_circ_002301	LOC107999877	NW_016019386.1	+	1008144	1012060	1127	Exonic
novel_circ_002302	LOC107999877	NW_016019386.1	+	1009480	1009764	285	Exonic
novel_circ_002303	LOC107999877	NW_016019386.1	+	1009480	1012395	891	Exonic
novel_circ_002304	LOC107999877	NW_016019386.1	+	1011537	1012395	582	Exonic
novel_circ_002305	LOC107999877	NW_016019386.1	+	1011537	1021229	879	Exonic
novel_circ_002306	LOC107999837	NW_016019386.1	+	1030908	1034082	3175	Exon–intron
novel_circ_002307	LOC107999864	NW_016019386.1	-	1067124	1068231	930	Exonic
novel_circ_002308	LOC107999864	NW_016019386.1	-	1067489	1068231	663	Exonic
novel_circ_002309	LOC107999864	NW_016019386.1	-	1067489	1070612	810	Exonic
novel_circ_002310	LOC107999909	NW_016019386.1	-	1134308	1134468	161	Exonic
novel_circ_002311	LOC107999918	NW_016019388.1	+	2534	5831	1577	Exon–intron
novel_circ_002312	LOC107999918	NW_016019388.1	+	4831	5263	433	Exonic
novel_circ_002313	LOC107999918	NW_016019388.1	+	4831	5831	716	Exonic
novel_circ_002314	LOC107999926	NW_016019393.1	+	5106	8847	467	Exonic
novel_circ_002315	LOC107999927	NW_016019397.1	-	2734	4355	1622	Exonic
novel_circ_002316	LOC107999927	NW_016019397.1	-	9304	10956	859	Exonic
novel_circ_002317	LOC107999982	NW_016019397.1	+	29531	32976	1262	Exonic
novel_circ_002318	LOC107999982	NW_016019397.1	+	34935	36833	1389	Exon–intron
novel_circ_002319	LOC107999982	NW_016019397.1	+	34948	36833	1376	Exonic
novel_circ_002320	LOC108000017	NW_016019397.1	+	93528	95081	1068	Exonic
novel_circ_002321	LOC107999979	NW_016019397.1	-	133008	149416	16060	Exon–intron
novel_circ_002322	LOC107999978	NW_016019397.1	-	148823	163927	1433	Exonic
novel_circ_002323	LOC107999978	NW_016019397.1	-	149109	163927	1147	Exonic
novel_circ_002324	LOC114577815	NW_016019397.1	-	262083	271584	9502	Exon–intron
novel_circ_002325	LOC114577815	NW_016019397.1	-	265910	271584	5675	Exon–intron
novel_circ_002326	LOC114577815	NW_016019397.1	-	267408	271584	4177	Exon–intron
novel_circ_002327	LOC107999933	NW_016019397.1	+	320590	321413	749	Exonic
novel_circ_002328	LOC107999995	NW_016019397.1	-	392559	398685	548	Exonic
novel_circ_002329	LOC107999995	NW_016019397.1	-	392559	402664	1047	Exonic
novel_circ_002330	LOC107999997	NW_016019397.1	-	527650	532335	556	Exonic
novel_circ_002331	LOC107999997	NW_016019397.1	-	531716	532335	276	Exonic
novel_circ_002332	LOC108000039	NW_016019397.1	+	743261	744953	1590	Exon–intron
novel_circ_002333	LOC107999943	NW_016019397.1	-	828247	829099	853	Antisense
novel_circ_002334	LOC107999943	NW_016019397.1	-	829316	829487	172	Antisense
novel_circ_002335	LOC107999943	NW_016019397.1	+	829317	829695	296	Exonic
novel_circ_002336	LOC107999943	NW_016019397.1	+	830201	831348	630	Exon–intron
novel_circ_002337	LOC107999969	NW_016019397.1	+	847448	847602	155	Antisense
novel_circ_002338	LOC108000026	NW_016019397.1	+	913364	913931	376	Exonic
novel_circ_002339	LOC108000026	NW_016019397.1	-	913627	915059	1433	Antisense
novel_circ_002340	LOC108000026	NW_016019397.1	-	913642	915435	1794	Antisense
novel_circ_002341	LOC108000026	NW_016019397.1	-	913836	914910	1075	Antisense
novel_circ_002342	LOC108000026	NW_016019397.1	+	914215	914787	249	Exonic
novel_circ_002343	LOC108000028	NW_016019397.1	-	1015035	1027415	1160	Exonic
novel_circ_002344	LOC108000029	NW_016019397.1	+	1027182	1029698	2517	Exon–intron
novel_circ_002345	LOC108000059	NW_016019399.1	+	1018	1953	671	Exonic
novel_circ_002346	LOC108000078	NW_016019408.1	+	208469	261848	50232	Exon–intron

novel_circ_002347	LOC108000095	NW_016019408.1	+	632689	634431	359	Exonic
novel_circ_002348	LOC108000095	NW_016019408.1	+	632689	640985	2914	Exonic
novel_circ_002349	LOC108000089	NW_016019408.1	+	742917	744357	984	Exonic
novel_circ_002350	LOC108000102	NW_016019408.1	-	955600	958635	2750	Exon–intron
novel_circ_002351	LOC108000123	NW_016019419.1	-	48256	50761	839	Exonic
novel_circ_002352	LOC108000123	NW_016019419.1	-	64781	70136	773	Exonic
novel_circ_002353	LOC108000151	NW_016019419.1	+	242014	260216	18203	Exon–intron
novel_circ_002354	LOC108000109	NW_016019419.1	+	338750	339330	448	Exonic
novel_circ_002355	LOC108000109	NW_016019419.1	+	338750	345793	6911	Exon–intron
novel_circ_002356	LOC108000147	NW_016019419.1	+	417745	418140	314	Exonic
novel_circ_002357	LOC108000127	NW_016019419.1	+	558686	561873	2244	Exonic
novel_circ_002358	LOC108000141	NW_016019419.1	-	598181	604456	6276	Exon–intron
novel_circ_002359	NA	NW_016019430.1	-	43878	44257	380	Intergenic region
novel_circ_002360	LOC108000203	NW_016019430.1	-	43878	52436	6046	Exon–intron
novel_circ_002361	LOC108000203	NW_016019430.1	-	48016	52436	1908	Exonic
novel_circ_002362	LOC108000196	NW_016019430.1	-	239052	240513	1462	Exon–intron
novel_circ_002363	NA	NW_016019430.1	-	239472	240513	1042	Intergenic region
novel_circ_002364	LOC108000189	NW_016019430.1	+	292967	301333	8367	Exon–intron
novel_circ_002365	LOC108000201	NW_016019430.1	+	514314	515291	663	Exonic
novel_circ_002366	LOC108000201	NW_016019430.1	+	514314	517090	913	Exonic
novel_circ_002367	LOC108000201	NW_016019430.1	+	514314	518155	1193	Exonic
novel_circ_002368	LOC108000201	NW_016019430.1	+	514314	529855	1693	Exonic
novel_circ_002369	LOC108000201	NW_016019430.1	+	514314	532774	1941	Exonic
novel_circ_002370	LOC108000201	NW_016019430.1	+	516841	518155	530	Exonic
novel_circ_002371	LOC108000201	NW_016019430.1	+	517999	541085	9216	Exon–intron
novel_circ_002372	LOC108000201	NW_016019430.1	+	519299	520936	500	Exon–intron
novel_circ_002373	LOC108000201	NW_016019430.1	+	519299	529855	500	Exonic
novel_circ_002374	LOC114577832	NW_016019430.1	-	685860	686171	312	Exon–intron
novel_circ_002375	LOC108000176	NW_016019430.1	-	690094	690260	167	Exonic
novel_circ_002376	LOC108000176	NW_016019430.1	-	690228	690729	502	Exonic
novel_circ_002377	LOC108000176	NW_016019430.1	-	694364	695281	312	Exonic
novel_circ_002378	LOC108000173	NW_016019430.1	+	789557	790493	656	Exonic
novel_circ_002379	LOC108000176	NW_016019430.1	-	832003	832243	241	Exonic
novel_circ_002380	LOC108000176	NW_016019430.1	-	870011	872184	2174	Intronic
novel_circ_002381	LOC108000176	NW_016019430.1	-	879134	880695	1562	Exon–intron
novel_circ_002382	LOC108000176	NW_016019430.1	-	880533	887363	3379	Exon–intron
novel_circ_002383	NA	NW_016019430.1	-	921781	923506	1726	Intergenic region
novel_circ_002384	LOC108000168	NW_016019430.1	-	946339	948679	357	Exonic
novel_circ_002385	LOC108000168	NW_016019430.1	-	948479	954555	896	Exon–intron
novel_circ_002386	LOC108000170	NW_016019430.1	+	960522	962084	1091	Exonic
novel_circ_002387	LOC108000170	NW_016019430.1	+	961096	964401	1311	Exonic
novel_circ_002388	LOC108000170	NW_016019430.1	+	964004	964401	322	Exonic
novel_circ_002389	LOC108000170	NW_016019430.1	+	964004	964457	378	Exon–intron
novel_circ_002390	LOC108000170	NW_016019430.1	+	965012	965368	357	Exonic
novel_circ_002391	LOC108000182	NW_016019430.1	-	966287	966529	243	Intronic
novel_circ_002392	LOC114577829	NW_016019430.1	-	1002230	1002399	170	Exonic
novel_circ_002393	LOC108000184	NW_016019430.1	-	1004390	1009350	1242	Exonic
novel_circ_002394	LOC108000184	NW_016019430.1	-	1006645	1009350	771	Exonic
novel_circ_002395	LOC108000212	NW_016019430.1	+	1049066	1050757	752	Exonic

novel_circ_002396	LOC108000488	NW_016019441.1	-	11273	14529	3257	Antisense
novel_circ_002397	LOC108000225	NW_016019441.1	+	157711	160424	352	Exonic
novel_circ_002398	LOC108000225	NW_016019441.1	+	160302	189553	730	Exonic
novel_circ_002399	LOC108000225	NW_016019441.1	+	179214	193259	8768	Exon–intron
novel_circ_002400	LOC108000449	NW_016019441.1	-	217562	220694	330	Exonic
novel_circ_002401	LOC108000476	NW_016019441.1	-	300398	301207	810	Antisense
novel_circ_002402	LOC108000454	NW_016019441.1	-	303183	303392	210	Exonic
novel_circ_002403	LOC108000454	NW_016019441.1	-	305995	307719	671	Exonic
novel_circ_002404	LOC108000454	NW_016019441.1	+	306084	306257	174	Antisense
novel_circ_002405	LOC108000369	NW_016019441.1	-	341401	341926	526	Exonic
novel_circ_002406	LOC108000369	NW_016019441.1	-	345959	347288	1128	Exonic
novel_circ_002407	LOC108000369	NW_016019441.1	-	345959	350473	1266	Exonic
novel_circ_002408	LOC108000369	NW_016019441.1	+	353580	353747	168	Antisense
novel_circ_002409	LOC108000369	NW_016019441.1	-	354149	354543	395	Exonic
novel_circ_002410	LOC108000337	NW_016019441.1	-	429455	433053	3599	Exon–intron
novel_circ_002411	LOC108000480	NW_016019441.1	-	462097	462297	201	Antisense
novel_circ_002412	LOC108000480	NW_016019441.1	+	462097	462297	201	Exonic
novel_circ_002413	LOC108000480	NW_016019441.1	+	462116	462318	203	Exon–intron
novel_circ_002414	LOC108000362	NW_016019441.1	+	518256	521004	2442	Exonic
novel_circ_002415	LOC108000457	NW_016019441.1	-	1198699	1201491	651	Exonic
novel_circ_002416	LOC108000343	NW_016019441.1	+	1756641	1758123	381	Exonic
novel_circ_002417	LOC108000352	NW_016019441.1	+	1834119	1843296	264	Exonic
novel_circ_002418	LOC108000443	NW_016019441.1	+	1953290	1954160	381	Exonic
novel_circ_002419	LOC108000329	NW_016019441.1	-	2324524	2326468	1147	Exonic
novel_circ_002420	LOC108000329	NW_016019441.1	-	2350256	2352079	427	Exonic
novel_circ_002421	LOC108000310	NW_016019441.1	+	2981449	2981857	409	Exonic
novel_circ_002422	LOC108000310	NW_016019441.1	+	2981449	2997669	517	Exonic
novel_circ_002423	LOC108000242	NW_016019441.1	+	3126778	3130381	189	Exonic
novel_circ_002424	LOC108000242	NW_016019441.1	+	3163843	3165472	1630	Exonic
novel_circ_002425	LOC108000254	NW_016019441.1	-	4046865	4047951	717	Exonic
novel_circ_002426	LOC108000332	NW_016019441.1	+	4130178	4132015	638	Exonic
novel_circ_002427	LOC108000332	NW_016019441.1	+	4130178	4139645	1923	Exonic
novel_circ_002428	LOC108000332	NW_016019441.1	+	4133465	4134029	251	Exonic
novel_circ_002429	LOC108000332	NW_016019441.1	+	4133465	4139645	1212	Exonic
novel_circ_002430	LOC108000332	NW_016019441.1	+	4139230	4139645	226	Exonic
novel_circ_002431	LOC108000332	NW_016019441.1	+	4146376	4161040	3310	Exonic
novel_circ_002432	LOC108000332	NW_016019441.1	+	4160836	4164840	1024	Exonic
novel_circ_002433	LOC108000402	NW_016019441.1	+	4239720	4241289	526	Exonic
novel_circ_002434	LOC108000374	NW_016019441.1	-	4281092	4283603	2051	Exonic
novel_circ_002435	LOC108000393	NW_016019441.1	-	4385182	4387442	1072	Exonic
novel_circ_002436	NA	NW_016019441.1	-	4593595	4598829	5235	Intergenic region
novel_circ_002437	NA	NW_016019441.1	-	4594529	4598829	4301	Intergenic region
novel_circ_002438	NA	NW_016019441.1	-	4596812	4598829	2018	Intergenic region
novel_circ_002439	LOC108000581	NW_016019442.1	+	42963	51080	8118	Exon–intron
novel_circ_002440	LOC108000557	NW_016019442.1	-	122209	122909	701	Intronic
novel_circ_002441	LOC108000569	NW_016019442.1	+	360053	361827	1613	Exon–intron
novel_circ_002442	LOC108000569	NW_016019442.1	+	361391	361827	275	Exonic
novel_circ_002443	LOC108000569	NW_016019442.1	+	361391	363738	818	Exonic
novel_circ_002444	LOC108000569	NW_016019442.1	+	362846	363738	543	Exonic

novel_circ_002445	LOC108000547	NW_016019442.1	+	481712	485016	316	Exonic
novel_circ_002446	LOC108000547	NW_016019442.1	+	484825	491125	377	Exonic
novel_circ_002447	LOC108000525	NW_016019442.1	+	633419	634183	348	Exon–intron
novel_circ_002448	LOC108000525	NW_016019442.1	-	633597	635070	1474	Antisense
novel_circ_002449	LOC108000589	NW_016019442.1	-	739150	739622	347	Exonic
novel_circ_002450	LOC108000589	NW_016019442.1	-	739150	740045	501	Exonic
novel_circ_002451	LOC108000575	NW_016019442.1	+	870790	871572	783	Antisense
novel_circ_002452	LOC108000575	NW_016019442.1	+	871068	871481	414	Antisense
novel_circ_002453	LOC108000565	NW_016019442.1	-	925805	925960	156	Antisense
novel_circ_002454	LOC108000531	NW_016019442.1	-	1013504	1014000	497	Exonic
novel_circ_002455	LOC108000531	NW_016019442.1	+	1013681	1014050	370	Antisense
novel_circ_002456	LOC108000684	NW_016019453.1	-	196604	197294	251	Exonic
novel_circ_002457	LOC108000684	NW_016019453.1	-	196604	208175	358	Exonic
novel_circ_002458	LOC108000652	NW_016019453.1	+	285295	303654	936	Exonic
novel_circ_002459	LOC108000656	NW_016019453.1	+	536003	536180	178	Exonic
novel_circ_002460	LOC108000656	NW_016019453.1	-	536025	536454	430	Antisense
novel_circ_002461	LOC108000656	NW_016019453.1	+	538522	539143	543	Exonic
novel_circ_002462	LOC108000688	NW_016019453.1	-	605057	614680	8426	Exon–intron
novel_circ_002463	LOC108000688	NW_016019453.1	-	605599	614680	7884	Exon–intron
novel_circ_002464	LOC108000697	NW_016019453.1	-	697595	698018	331	Exonic
novel_circ_002465	LOC108000632	NW_016019453.1	-	743520	744403	650	Exonic
novel_circ_002466	LOC108000710	NW_016019454.1	+	4989	7853	754	Exonic
novel_circ_002467	LOC108000772	NW_016019464.1	-	34308	37014	676	Exonic
novel_circ_002468	LOC108000772	NW_016019464.1	-	35860	37014	555	Exonic
novel_circ_002469	LOC108000772	NW_016019464.1	-	38407	38610	204	Exon–intron
novel_circ_002470	LOC108000765	NW_016019464.1	+	287451	295420	2038	Exonic
novel_circ_002471	LOC108000757	NW_016019464.1	-	449053	452139	3087	Exon–intron
novel_circ_002472	LOC108000757	NW_016019464.1	-	449102	452139	3038	Exon–intron
novel_circ_002473	LOC108000731	NW_016019464.1	-	548456	549510	1055	Exon–intron
novel_circ_002474	LOC108000773	NW_016019464.1	+	789060	799765	2132	Exonic
novel_circ_002475	LOC108000773	NW_016019464.1	+	798568	799395	652	Exonic
novel_circ_002476	LOC108000751	NW_016019464.1	+	823203	830721	1755	Exonic
novel_circ_002477	LOC108000751	NW_016019464.1	+	823203	843307	2280	Exonic
novel_circ_002478	NA	NW_016019474.1	-	3316	3530	215	Intergenic region
novel_circ_002479	LOC108000796	NW_016019475.1	+	147620	150736	649	Exonic
novel_circ_002480	LOC108000798	NW_016019475.1	+	147620	166742	16952	Exon–intron
novel_circ_002481	LOC108000796	NW_016019475.1	+	150269	150736	468	Exonic
novel_circ_002482	LOC108000798	NW_016019475.1	+	162642	163050	409	Exonic
novel_circ_002483	LOC108000798	NW_016019475.1	+	162642	171519	3985	Exonic
novel_circ_002484	LOC108000798	NW_016019475.1	+	164904	165489	503	Exonic
novel_circ_002485	LOC108000798	NW_016019475.1	+	164904	166742	1521	Exonic
novel_circ_002486	LOC108000798	NW_016019475.1	+	167626	168515	814	Exonic
novel_circ_002487	LOC108000823	NW_016019475.1	+	314245	315879	283	Exonic
novel_circ_002488	LOC108000823	NW_016019475.1	+	314245	316492	591	Exonic
novel_circ_002489	LOC108000823	NW_016019475.1	+	314245	334048	2134	Exonic
novel_circ_002490	LOC108000823	NW_016019475.1	+	315730	316492	458	Exonic
novel_circ_002491	LOC108000823	NW_016019475.1	+	315730	327508	582	Exonic
novel_circ_002492	LOC108000823	NW_016019475.1	+	332008	334048	1143	Exonic
novel_circ_002493	LOC108000859	NW_016019475.1	+	376399	377402	561	Exonic

novel_circ_002494	LOC108000859	NW_016019475.1	+	378978	379203	226	Exonic
novel_circ_002495	LOC108000800	NW_016019475.1	+	388805	389950	359	Exonic
novel_circ_002496	LOC108000800	NW_016019475.1	+	413392	413722	331	Exonic
novel_circ_002497	LOC108000800	NW_016019475.1	+	413392	415974	564	Exonic
novel_circ_002498	LOC108000839	NW_016019475.1	-	452883	453580	552	Exonic
novel_circ_002499	LOC108000839	NW_016019475.1	-	452883	481845	3051	Exonic
novel_circ_002500	LOC108000839	NW_016019475.1	-	453515	454059	243	Exonic
novel_circ_002501	LOC108000839	NW_016019475.1	-	471242	471754	254	Exonic
novel_circ_002502	LOC108000839	NW_016019475.1	-	471242	471758	258	Exonic
novel_circ_002503	LOC108000839	NW_016019475.1	-	471242	476299	590	Exonic
novel_circ_002504	LOC108000839	NW_016019475.1	-	471618	481845	651	Exonic
novel_circ_002505	LOC108000839	NW_016019475.1	-	471618	496478	14312	Exon–intron
novel_circ_002506	LOC108000839	NW_016019475.1	-	475968	476299	332	Exonic
novel_circ_002507	LOC108000839	NW_016019475.1	-	475968	481845	510	Exonic
novel_circ_002508	LOC108000839	NW_016019475.1	-	475968	496478	14171	Exon–intron
novel_circ_002509	LOC114577924	NW_016019475.1	-	493481	499544	5092	Exon–intron
novel_circ_002510	LOC108000826	NW_016019475.1	+	556761	569584	12257	Exon–intron
novel_circ_002511	LOC108000826	NW_016019475.1	+	556761	569772	12445	Exon–intron
novel_circ_002512	LOC108000838	NW_016019475.1	+	752724	753814	618	Exonic
novel_circ_002513	LOC108000838	NW_016019475.1	+	752724	761786	924	Exonic
novel_circ_002514	LOC108000838	NW_016019475.1	+	752724	761795	933	Exonic
novel_circ_002515	LOC108000838	NW_016019475.1	+	752724	763650	1197	Exonic
novel_circ_002516	LOC108000838	NW_016019475.1	+	753429	753814	262	Exonic
novel_circ_002517	LOC108000838	NW_016019475.1	+	761630	763650	430	Exonic
novel_circ_002518	LOC108000838	NW_016019475.1	+	761630	764915	1695	Exonic
novel_circ_002519	LOC108000838	NW_016019475.1	+	763387	764915	1529	Exonic
novel_circ_002520	LOC108000836	NW_016019475.1	+	770848	775275	2585	Exonic
novel_circ_002521	LOC108000805	NW_016019475.1	-	844669	846186	1159	Exon–intron
novel_circ_002522	LOC108000805	NW_016019475.1	-	845176	846186	652	Exonic
novel_circ_002523	LOC108000854	NW_016019475.1	-	891821	892336	414	Exonic
novel_circ_002524	LOC108000834	NW_016019475.1	+	988392	988451	60	Antisense
novel_circ_002525	LOC108000834	NW_016019475.1	-	988400	988899	165	Exonic
novel_circ_002526	LOC108000834	NW_016019475.1	+	988400	988899	500	Antisense
novel_circ_002527	LOC108000834	NW_016019475.1	-	988400	988989	255	Exonic
novel_circ_002528	LOC108000834	NW_016019475.1	+	988415	988929	515	Antisense
novel_circ_002529	LOC108000913	NW_016019486.1	+	46981	50959	791	Exonic
novel_circ_002530	LOC108000913	NW_016019486.1	+	201975	203875	426	Exonic
novel_circ_002531	LOC108000913	NW_016019486.1	+	201975	222142	700	Exonic
novel_circ_002532	LOC108000909	NW_016019486.1	-	337352	352941	270	Exonic
novel_circ_002533	LOC108000918	NW_016019487.1	+	176	3440	616	Exonic
novel_circ_002534	LOC108000921	NW_016019494.1	-	337	1899	631	Exonic
novel_circ_002535	LOC108000921	NW_016019494.1	-	337	2086	703	Exonic
novel_circ_002536	LOC108000926	NW_016019497.1	+	33418	34069	652	Exonic
novel_circ_002537	LOC108000936	NW_016019497.1	+	152790	153735	276	Exonic
novel_circ_002538	LOC108000936	NW_016019497.1	+	152790	154295	480	Exonic
novel_circ_002539	LOC108000936	NW_016019497.1	+	152790	155820	604	Exonic
novel_circ_002540	LOC108000936	NW_016019497.1	+	152790	155829	613	Exon–intron
novel_circ_002541	LOC114577933	NW_016019497.1	-	285822	298471	276	Exonic
novel_circ_002542	LOC114577933	NW_016019497.1	-	322297	323678	1382	Exonic

novel_circ_002543	LOC108000937	NW_016019497.1	-	512159	513739	281	Exonic
novel_circ_002544	LOC108000940	NW_016019497.1	-	795146	802388	2071	Exonic
novel_circ_002545	LOC108000938	NW_016019497.1	+	941756	942351	371	Exonic
novel_circ_002546	LOC108001011	NW_016019508.1	+	77770	79232	809	Exonic
novel_circ_002547	LOC108001011	NW_016019508.1	+	78414	79232	741	Exonic
novel_circ_002548	LOC108001011	NW_016019508.1	+	95288	95775	488	Exonic
novel_circ_002549	LOC108000955	NW_016019508.1	+	131832	134366	2271	Exonic
novel_circ_002550	LOC108000955	NW_016019508.1	+	131832	146471	14155	Exon–intron
novel_circ_002551	LOC108001000	NW_016019508.1	-	165119	166148	1030	Exon–intron
novel_circ_002552	LOC108001029	NW_016019508.1	-	395615	396605	918	Exonic
novel_circ_002553	LOC108001018	NW_016019508.1	-	509102	519611	8361	Exon–intron
novel_circ_002554	LOC108001008	NW_016019508.1	+	525139	526412	803	Exonic
novel_circ_002555	LOC108001033	NW_016019508.1	-	560781	563255	1209	Exonic
novel_circ_002556	LOC108001033	NW_016019508.1	-	560781	564023	1435	Exonic
novel_circ_002557	LOC108001033	NW_016019508.1	-	561396	561822	341	Exonic
novel_circ_002558	LOC108001033	NW_016019508.1	-	561396	563255	942	Exonic
novel_circ_002559	LOC108001033	NW_016019508.1	-	561396	564023	1168	Exonic
novel_circ_002560	LOC108001033	NW_016019508.1	-	563139	564023	343	Exonic
novel_circ_002561	LOC108001033	NW_016019508.1	-	563139	565513	564	Exonic
novel_circ_002562	LOC108001033	NW_016019508.1	-	563798	565513	447	Exonic
novel_circ_002563	LOC108000956	NW_016019508.1	+	599641	599965	325	Exonic
novel_circ_002564	LOC108000974	NW_016019508.1	+	629347	629562	216	Exonic
novel_circ_002565	LOC108000974	NW_016019508.1	-	629455	630537	1083	Antisense
novel_circ_002566	LOC108001012	NW_016019508.1	+	704306	705964	1659	Exon–intron
novel_circ_002567	LOC108001012	NW_016019508.1	+	704306	727262	589	Exonic
novel_circ_002568	LOC108000995	NW_016019508.1	-	738155	740766	2019	Exonic
novel_circ_002569	LOC108000995	NW_016019508.1	-	740955	743818	2017	Exonic
novel_circ_002570	LOC108000995	NW_016019508.1	-	743042	743818	462	Exonic
novel_circ_002571	LOC108000995	NW_016019508.1	-	743644	743818	175	Exonic
novel_circ_002572	LOC108000994	NW_016019508.1	-	766463	767031	404	Exonic
novel_circ_002573	LOC108001013	NW_016019508.1	-	783068	783495	352	Exonic
novel_circ_002574	LOC108001004	NW_016019508.1	-	846517	848884	1583	Exonic
novel_circ_002575	LOC108001004	NW_016019508.1	-	847808	848884	834	Exonic
novel_circ_002576	LOC108001004	NW_016019508.1	-	857845	859133	532	Exonic
novel_circ_002577	LOC108001006	NW_016019508.1	+	945580	945957	378	Antisense
novel_circ_002578	LOC108001050	NW_016019513.1	-	4034	4482	353	Exonic
novel_circ_002579	LOC108001060	NW_016019519.1	-	582928	592926	9802	Exon–intron
novel_circ_002580	LOC108001060	NW_016019519.1	-	582928	592945	9821	Exon–intron
novel_circ_002581	LOC108001070	NW_016019519.1	+	604392	606018	1627	Antisense
novel_circ_002582	LOC108001070	NW_016019519.1	-	605644	608894	1268	Exonic
novel_circ_002583	LOC108001070	NW_016019519.1	-	650557	650830	274	Exonic
novel_circ_002584	LOC108001070	NW_016019519.1	-	656916	659577	2146	Exonic
novel_circ_002585	LOC108001083	NW_016019519.1	+	769904	770557	552	Exonic
novel_circ_002586	LOC108001083	NW_016019519.1	+	771939	772859	789	Exonic
novel_circ_002587	LOC108001083	NW_016019519.1	+	771939	773238	971	Exonic
novel_circ_002588	LOC108001083	NW_016019519.1	+	780980	781149	170	Exonic
novel_circ_002589	LOC108001100	NW_016019519.1	-	797538	819042	21190	Exon–intron
novel_circ_002590	LOC108001100	NW_016019519.1	-	797754	819042	20974	Exon–intron
novel_circ_002591	LOC108001100	NW_016019519.1	-	810189	819042	8539	Exon–intron

novel_circ_002592	LOC108001079	NW_016019519.1	-	864127	865058	480	Exonic
novel_circ_002593	LOC108001079	NW_016019519.1	-	865247	865594	262	Exonic
novel_circ_002594	LOC108001121	NW_016019519.1	-	949867	958816	7021	Exon–intron
novel_circ_002595	LOC108001132	NW_016019522.1	+	3494	6261	1960	Exonic
novel_circ_002596	LOC108001152	NW_016019530.1	-	119027	119161	135	Exonic
novel_circ_002597	LOC108001153	NW_016019530.1	+	119027	119161	135	Intronic
novel_circ_002598	LOC108001153	NW_016019530.1	+	119027	119368	342	Intronic
novel_circ_002599	LOC108001152	NW_016019530.1	-	119034	119102	69	Exonic
novel_circ_002600	LOC108001153	NW_016019530.1	+	119034	119102	69	Intronic
novel_circ_002601	LOC108001152	NW_016019530.1	-	119035	119169	135	Exonic
novel_circ_002602	LOC108001153	NW_016019530.1	+	119035	119169	135	Intronic
novel_circ_002603	LOC108001152	NW_016019530.1	-	119104	119169	66	Exonic
novel_circ_002604	LOC108001152	NW_016019530.1	-	119107	119310	204	Exonic
novel_circ_002605	LOC108001153	NW_016019530.1	+	119107	119310	204	Intronic
novel_circ_002606	LOC108001152	NW_016019530.1	-	119107	119379	273	Exonic
novel_circ_002607	LOC108001153	NW_016019530.1	+	119108	119311	204	Intronic
novel_circ_002608	LOC108001152	NW_016019530.1	-	119108	119311	204	Exonic
novel_circ_002609	LOC108001152	NW_016019530.1	-	119108	119380	273	Exonic
novel_circ_002610	LOC108001153	NW_016019530.1	+	119108	119380	273	Intronic
novel_circ_002611	LOC108001153	NW_016019530.1	+	119120	119182	63	Intronic
novel_circ_002612	LOC108001152	NW_016019530.1	-	119120	119182	63	Exonic
novel_circ_002613	LOC108001153	NW_016019530.1	+	153409	153858	450	Exon–intron
novel_circ_002614	NA	NW_016019530.1	-	260219	260406	188	Intergenic region
novel_circ_002615	LOC108001149	NW_016019530.1	-	262450	266666	888	Exonic
novel_circ_002616	LOC108001200	NW_016019530.1	+	368295	390707	9602	Exon–intron
novel_circ_002617	LOC108001200	NW_016019530.1	+	368295	390735	9630	Exon–intron
novel_circ_002618	LOC108001221	NW_016019530.1	-	417217	417694	401	Exonic
novel_circ_002619	LOC108001218	NW_016019530.1	+	511416	511869	454	Exonic
novel_circ_002620	LOC108001172	NW_016019530.1	+	568027	568318	292	Exonic
novel_circ_002621	LOC108001178	NW_016019530.1	-	630047	659801	458	Exonic
novel_circ_002622	LOC108001178	NW_016019530.1	-	659550	660458	909	Exon–intron
novel_circ_002623	LOC108001209	NW_016019530.1	+	808334	810490	890	Exonic
novel_circ_002624	LOC108001230	NW_016019541.1	+	110134	114294	4161	Exon–intron
novel_circ_002625	LOC108001231	NW_016019541.1	+	160754	167218	6376	Exon–intron
novel_circ_002626	LOC108001231	NW_016019541.1	+	166990	167218	229	Intronic
novel_circ_002627	LOC108001231	NW_016019541.1	+	166990	168683	1694	Intronic
novel_circ_002628	LOC108001248	NW_016019541.1	-	192628	226316	32951	Exon–intron
novel_circ_002629	LOC108001250	NW_016019541.1	+	209718	215649	5932	Exon–intron
novel_circ_002630	LOC108001252	NW_016019541.1	+	489924	490529	606	Exonic
novel_circ_002631	LOC108001252	NW_016019541.1	+	500377	504306	340	Exonic
novel_circ_002632	LOC108001366	NW_016019552.1	-	22103	27271	3579	Exon–intron
novel_circ_002633	LOC108001367	NW_016019552.1	-	101823	107846	5707	Exon–intron
novel_circ_002634	LOC108001402	NW_016019552.1	+	174382	174610	229	Exonic
novel_circ_002635	LOC108001342	NW_016019552.1	+	262927	278684	14295	Exon–intron
novel_circ_002636	LOC108001342	NW_016019552.1	+	262931	278684	14291	Exon–intron
novel_circ_002637	LOC108001343	NW_016019552.1	-	271531	277785	401	Exonic
novel_circ_002638	LOC108001343	NW_016019552.1	-	310008	312681	389	Exonic
novel_circ_002639	NA	NW_016019552.1	+	378066	378341	276	Intergenic region
novel_circ_002640	NA	NW_016019552.1	+	378069	378256	188	Intergenic region

novel_circ_002641	NA	NW_016019552.1	+	378079	378326	248	Intergenic region
novel_circ_002642	NA	NW_016019552.1	+	378082	378260	179	Intergenic region
novel_circ_002643	NA	NW_016019552.1	-	378094	378260	167	Intergenic region
novel_circ_002644	NA	NW_016019552.1	-	378100	378259	160	Intergenic region
novel_circ_002645	NA	NW_016019552.1	+	378102	378256	155	Intergenic region
novel_circ_002646	NA	NW_016019552.1	-	378107	378267	161	Intergenic region
novel_circ_002647	LOC108001369	NW_016019552.1	-	533009	533829	821	Exonic
novel_circ_002648	LOC108001418	NW_016019552.1	+	560240	560395	156	Exonic
novel_circ_002649	LOC108001395	NW_016019552.1	+	616171	619246	2946	Exon–intron
novel_circ_002650	LOC108001419	NW_016019552.1	-	1844988	1846451	645	Exonic
novel_circ_002651	LOC108001331	NW_016019552.1	+	2337070	2347657	9063	Exon–intron
novel_circ_002652	LOC108001331	NW_016019552.1	+	2337070	2349205	9306	Exon–intron
novel_circ_002653	LOC108001413	NW_016019552.1	+	2885055	2902544	765	Exonic
novel_circ_002654	LOC108001413	NW_016019552.1	+	2897639	2902544	572	Exonic
novel_circ_002655	LOC108001413	NW_016019552.1	+	2902049	2902544	312	Exonic
novel_circ_002656	LOC108001349	NW_016019552.1	-	2930029	2943639	2436	Exonic
novel_circ_002657	LOC108001349	NW_016019552.1	-	2940697	2943639	872	Exonic
novel_circ_002658	LOC108001352	NW_016019552.1	+	3608386	3610492	807	Exonic
novel_circ_002659	LOC108001352	NW_016019552.1	+	3631174	3632440	1267	Exon–intron
novel_circ_002660	LOC108001387	NW_016019552.1	-	3920915	3922086	530	Exonic
novel_circ_002661	LOC108001387	NW_016019552.1	-	3920915	3927952	660	Exonic
novel_circ_002662	LOC114577982	NW_016019552.1	+	4025250	4025521	272	Exonic
novel_circ_002663	LOC108001483	NW_016019553.1	-	123169	132356	995	Exonic
novel_circ_002664	LOC108001483	NW_016019553.1	-	131570	132356	485	Exonic
novel_circ_002665	LOC108001439	NW_016019553.1	+	180882	181352	264	Exonic
novel_circ_002666	LOC108001439	NW_016019553.1	+	180882	181398	310	Exonic
novel_circ_002667	LOC108001430	NW_016019553.1	+	297411	325200	17425	Exon–intron
novel_circ_002668	LOC108001454	NW_016019553.1	+	390840	392049	984	Exonic
novel_circ_002669	LOC108001454	NW_016019553.1	+	390840	392754	1566	Exonic
novel_circ_002670	LOC108001469	NW_016019553.1	+	527987	532971	2391	Exonic
novel_circ_002671	LOC108001469	NW_016019553.1	+	532845	533144	300	Exonic
novel_circ_002672	LOC108001520	NW_016019553.1	-	538382	539273	734	Exon–intron
novel_circ_002673	LOC108001520	NW_016019553.1	-	538386	539273	730	Exonic
novel_circ_002674	LOC108001512	NW_016019553.1	+	577721	577991	271	Exonic
novel_circ_002675	LOC108001512	NW_016019553.1	+	615332	617023	446	Exonic
novel_circ_002676	LOC108001512	NW_016019553.1	+	615332	629009	7180	Exon–intron
novel_circ_002677	NA	NW_016019553.1	-	665123	665741	619	Intergenic region
novel_circ_002678	NA	NW_016019553.1	+	665268	665480	213	Intergenic region
novel_circ_002679	LOC108001488	NW_016019553.1	+	669869	670926	1058	Exon–intron
novel_circ_002680	LOC108001468	NW_016019553.1	-	740435	741658	1224	Antisense
novel_circ_002681	LOC108001521	NW_016019556.1	-	1395	2271	356	Exonic
novel_circ_002682	LOC108001541	NW_016019564.1	+	41046	41965	708	Exonic
novel_circ_002683	LOC108001538	NW_016019564.1	+	75536	79062	302	Exonic
novel_circ_002684	LOC108001537	NW_016019564.1	+	270722	272798	1661	Exonic
novel_circ_002685	LOC108001537	NW_016019564.1	+	270722	274569	1806	Exonic
novel_circ_002686	LOC108001525	NW_016019564.1	-	301194	301493	300	Antisense
novel_circ_002687	LOC108001532	NW_016019564.1	+	774130	807788	523	Exonic
novel_circ_002688	LOC108001532	NW_016019564.1	+	805919	807788	280	Exonic
novel_circ_002689	LOC108001529	NW_016019564.1	-	856491	864811	8321	Exon–intron

novel_circ_002690	LOC108001529	NW_016019564.1	-	894318	895720	551	Exonic
novel_circ_002691	LOC108001544	NW_016019568.1	+	792	1508	406	Exonic
novel_circ_002692	LOC108001544	NW_016019568.1	+	792	2613	527	Exonic
novel_circ_002693	NA	NW_016019575.1	+	28806	56856	28051	Intergenic region
novel_circ_002694	LOC108001566	NW_016019586.1	+	113721	114505	597	Exonic
novel_circ_002695	LOC108001561	NW_016019586.1	+	178530	179629	560	Exonic
novel_circ_002696	LOC108001561	NW_016019586.1	+	178530	195063	882	Exonic
novel_circ_002697	LOC108001561	NW_016019586.1	+	194742	195063	322	Exonic
novel_circ_002698	LOC108001570	NW_016019586.1	+	306512	308870	705	Exonic
novel_circ_002699	LOC108001570	NW_016019586.1	+	308230	308870	412	Exonic
novel_circ_002700	LOC108001569	NW_016019586.1	-	389236	391322	967	Exonic
novel_circ_002701	LOC108001569	NW_016019586.1	-	389991	391322	723	Exonic
novel_circ_002702	LOC108001569	NW_016019586.1	-	390980	394274	1215	Exonic
novel_circ_002703	LOC108001569	NW_016019586.1	-	392096	394274	872	Exonic
novel_circ_002704	LOC108001569	NW_016019586.1	-	399574	404485	3026	Exonic
novel_circ_002705	LOC108001569	NW_016019586.1	-	401065	403278	1872	Exonic
novel_circ_002706	LOC108001569	NW_016019586.1	-	401065	404485	2021	Exonic
novel_circ_002707	LOC108001558	NW_016019586.1	+	815764	816101	338	Exonic
novel_circ_002708	LOC108001657	NW_016019597.1	-	42474	43343	870	Exonic
novel_circ_002709	LOC108001641	NW_016019597.1	-	100949	103155	1368	Exonic
novel_circ_002710	LOC108001641	NW_016019597.1	-	102184	103155	768	Exonic
novel_circ_002711	LOC108001641	NW_016019597.1	-	102666	103155	403	Exonic
novel_circ_002712	LOC108001650	NW_016019597.1	-	178189	182493	577	Exonic
novel_circ_002713	LOC108001607	NW_016019597.1	+	311543	311739	197	Antisense
novel_circ_002714	LOC108001636	NW_016019597.1	-	743428	744588	443	Exonic
novel_circ_002715	LOC108001636	NW_016019597.1	-	743428	746730	709	Exonic
novel_circ_002716	LOC108001697	NW_016019608.1	-	104726	107138	1064	Exonic
novel_circ_002717	LOC108001735	NW_016019608.1	+	157967	159319	1015	Exonic
novel_circ_002718	LOC108001735	NW_016019608.1	+	158621	159319	699	Exonic
novel_circ_002719	LOC108001687	NW_016019608.1	+	247880	248715	836	Antisense
novel_circ_002720	LOC108001688	NW_016019608.1	+	256355	257032	678	Antisense
novel_circ_002721	LOC108001671	NW_016019608.1	+	366944	369485	1237	Exonic
novel_circ_002722	LOC108001671	NW_016019608.1	+	425931	431989	5613	Exon–intron
novel_circ_002723	LOC108001671	NW_016019608.1	-	464285	464481	197	Antisense
novel_circ_002724	LOC108001671	NW_016019608.1	-	465873	466194	322	Antisense
novel_circ_002725	LOC108001717	NW_016019608.1	-	493068	493441	309	Exonic
novel_circ_002726	LOC108001702	NW_016019608.1	+	517044	520099	734	Exonic
novel_circ_002727	LOC108001736	NW_016019608.1	+	546122	549135	430	Exonic
novel_circ_002728	LOC108001732	NW_016019608.1	+	574141	574933	332	Exonic
novel_circ_002729	LOC108001718	NW_016019608.1	+	655270	657941	778	Exonic
novel_circ_002730	LOC108001667	NW_016019608.1	+	705648	708844	307	Exonic
novel_circ_002731	LOC108001750	NW_016019622.1	-	3750	3966	217	Exonic
novel_circ_002732	LOC108001828	NW_016019630.1	-	27221	33710	477	Exonic
novel_circ_002733	LOC108001828	NW_016019630.1	-	27221	33714	481	Exonic
novel_circ_002734	LOC108001834	NW_016019630.1	-	109062	110074	787	Exonic
novel_circ_002735	LOC108001786	NW_016019630.1	-	294638	296310	328	Exonic
novel_circ_002736	LOC108001794	NW_016019630.1	-	342023	347603	463	Exonic
novel_circ_002737	LOC108001839	NW_016019630.1	-	485901	487472	1572	Intronic
novel_circ_002738	LOC108001839	NW_016019630.1	-	486098	487472	1375	Intronic

novel_circ_002739	LOC108001826	NW_016019630.1	+	522876	523713	681	Exonic
novel_circ_002740	LOC108001802	NW_016019630.1	-	570867	573940	448	Exonic
novel_circ_002741	LOC108001803	NW_016019630.1	-	603811	605564	1754	Exon–intron
novel_circ_002742	LOC108001838	NW_016019630.1	+	695257	695881	473	Exonic
novel_circ_002743	LOC108001820	NW_016019630.1	+	703489	704605	327	Exonic
novel_circ_002744	LOC108001820	NW_016019630.1	+	712044	713538	928	Exonic
novel_circ_002745	LOC108001862	NW_016019634.1	+	3051	3484	434	Exon–intron
novel_circ_002746	LOC108001868	NW_016019641.1	+	15539	32396	2713	Exon–intron
novel_circ_002747	LOC108001869	NW_016019641.1	+	32043	53148	17078	Exon–intron
novel_circ_002748	LOC108001896	NW_016019641.1	+	57949	65677	2269	Exonic
novel_circ_002749	LOC108001896	NW_016019641.1	+	63157	65677	1008	Exonic
novel_circ_002750	LOC108001879	NW_016019641.1	-	111439	111852	414	Antisense
novel_circ_002751	LOC108001884	NW_016019641.1	-	116628	118092	262	Exonic
novel_circ_002752	LOC108001884	NW_016019641.1	-	116628	121806	335	Exonic
novel_circ_002753	LOC108001881	NW_016019641.1	-	171722	187062	15263	Exon–intron
novel_circ_002754	LOC108001882	NW_016019641.1	-	198004	198712	709	Antisense
novel_circ_002755	LOC108001889	NW_016019641.1	+	258344	258729	386	Exonic
novel_circ_002756	LOC108001888	NW_016019641.1	+	294034	294523	490	Exonic
novel_circ_002757	LOC108001908	NW_016019641.1	-	348758	349677	580	Exonic
novel_circ_002758	LOC108001871	NW_016019641.1	-	475278	476180	473	Exonic
novel_circ_002759	LOC108001871	NW_016019641.1	-	476000	477574	801	Exonic
novel_circ_002760	LOC108001871	NW_016019641.1	-	476000	477601	828	Exonic
novel_circ_002761	LOC108001871	NW_016019641.1	-	476000	486705	1580	Exonic
novel_circ_002762	LOC108001913	NW_016019641.1	-	680718	682020	905	Exonic
novel_circ_002763	LOC108001962	NW_016019652.1	+	8355	13314	4960	Exon–intron
novel_circ_002764	NA	NW_016019652.1	+	47285	47755	471	Intergenic region
novel_circ_002765	LOC108001957	NW_016019652.1	+	75648	93527	17880	Exon–intron
novel_circ_002766	LOC108001957	NW_016019652.1	+	102451	105178	2728	Exon–intron
novel_circ_002767	LOC108001957	NW_016019652.1	+	125513	127455	259	Exonic
novel_circ_002768	LOC108001946	NW_016019652.1	+	174150	179733	5458	Exon–intron
novel_circ_002769	LOC108001956	NW_016019652.1	-	203673	205565	396	Exonic
novel_circ_002770	LOC108001956	NW_016019652.1	-	203673	210999	491	Exonic
novel_circ_002771	LOC108001958	NW_016019652.1	+	543809	545043	867	Exonic
novel_circ_002772	LOC108001958	NW_016019652.1	+	543809	550057	4505	Exon–intron
novel_circ_002773	NA	NW_016019652.1	-	578820	579014	195	Intergenic region
novel_circ_002774	LOC108001950	NW_016019652.1	+	579381	595547	16167	Exon–intron
novel_circ_002775	LOC108002090	NW_016019663.1	-	293995	294358	364	Exonic
novel_circ_002776	LOC108002050	NW_016019663.1	-	442050	446216	910	Exonic
novel_circ_002777	LOC108002130	NW_016019663.1	+	562429	562787	279	Exonic
novel_circ_002778	LOC108002065	NW_016019663.1	-	648429	663621	14983	Exon–intron
novel_circ_002779	LOC108002065	NW_016019663.1	-	648429	663849	15211	Exon–intron
novel_circ_002780	LOC108002113	NW_016019663.1	+	817324	818788	1465	Exon–intron
novel_circ_002781	LOC108002111	NW_016019663.1	+	858938	859154	217	Exonic
novel_circ_002782	LOC108002113	NW_016019663.1	+	868210	868510	301	Intronic
novel_circ_002783	LOC108002108	NW_016019663.1	-	1045103	1050057	2188	Exonic
novel_circ_002784	LOC108002108	NW_016019663.1	-	1045103	1051372	2365	Exonic
novel_circ_002785	LOC108002108	NW_016019663.1	-	1046954	1063032	4318	Exonic
novel_circ_002786	LOC108002108	NW_016019663.1	-	1057075	1058215	787	Exonic
novel_circ_002787	LOC108002160	NW_016019663.1	+	1170896	1171117	222	Antisense

novel_circ_002788	LOC108002165	NW_016019663.1	+	1189168	1190696	938	Exonic
novel_circ_002789	LOC114578074	NW_016019663.1	-	1266659	1273249	5673	Exon–intron
novel_circ_002790	LOC108002117	NW_016019663.1	-	1352618	1353586	465	Exonic
novel_circ_002791	LOC108002133	NW_016019663.1	+	1480281	1480434	154	Exonic
novel_circ_002792	LOC108002101	NW_016019663.1	-	1509279	1509580	302	Exonic
novel_circ_002793	LOC108002101	NW_016019663.1	-	1509279	1519104	1015	Exonic
novel_circ_002794	LOC108002101	NW_016019663.1	-	1518392	1519104	713	Exonic
novel_circ_002795	LOC108002176	NW_016019663.1	+	1564001	1565133	851	Exonic
novel_circ_002796	LOC108002079	NW_016019663.1	+	1727172	1779394	634	Exonic
novel_circ_002797	LOC108002078	NW_016019663.1	-	1799824	1806236	916	Exonic
novel_circ_002798	LOC108002138	NW_016019663.1	+	1858983	1859561	487	Exonic
novel_circ_002799	LOC108002073	NW_016019663.1	-	1877883	1882933	1857	Exonic
novel_circ_002800	LOC108002073	NW_016019663.1	-	1878071	1882933	1788	Exonic
novel_circ_002801	LOC114578072	NW_016019663.1	-	1976118	1976903	258	Exon–intron
novel_circ_002802	LOC108002081	NW_016019663.1	+	2104183	2105052	577	Exonic
novel_circ_002803	LOC108002081	NW_016019663.1	+	2104183	2108513	2162	Exonic
novel_circ_002804	LOC108002081	NW_016019663.1	+	2105977	2107241	898	Exonic
novel_circ_002805	LOC108001990	NW_016019663.1	+	2328589	2330813	1790	Exonic
novel_circ_002806	LOC108002061	NW_016019663.1	+	2501954	2503194	1241	Exonic
novel_circ_002807	LOC108002061	NW_016019663.1	+	2613002	2622280	9279	Exon–intron
novel_circ_002808	LOC108002045	NW_016019663.1	-	2761062	2761918	694	Exonic
novel_circ_002809	LOC108002046	NW_016019663.1	+	2897566	2900767	451	Exonic
novel_circ_002810	LOC108002049	NW_016019663.1	-	3078085	3079000	465	Exon–intron
novel_circ_002811	LOC108002048	NW_016019663.1	+	3139175	3140955	1488	Exon–intron
novel_circ_002812	LOC108002038	NW_016019663.1	-	3282444	3283274	831	Antisense
novel_circ_002813	LOC108002040	NW_016019663.1	-	3286575	3287771	521	Exonic
novel_circ_002814	LOC108002041	NW_016019663.1	-	3317214	3318493	652	Exonic
novel_circ_002815	LOC108002041	NW_016019663.1	-	3317214	3318496	655	Exonic
novel_circ_002816	LOC108002059	NW_016019663.1	+	3386519	3465669	79151	Exon–intron
novel_circ_002817	LOC108002059	NW_016019663.1	+	3498859	3562668	1238	Exonic
novel_circ_002818	LOC108002059	NW_016019663.1	+	3522881	3544420	725	Exonic
novel_circ_002819	LOC108002060	NW_016019663.1	-	3534579	3535399	454	Exonic
novel_circ_002820	LOC108002060	NW_016019663.1	-	3540078	3540410	333	Exonic
novel_circ_002821	LOC108002059	NW_016019663.1	+	3544314	3562668	477	Exonic
novel_circ_002822	LOC108002059	NW_016019663.1	+	3562050	3562668	370	Exonic
novel_circ_002823	LOC108002059	NW_016019663.1	+	3587118	3587847	348	Exonic
novel_circ_002824	LOC108002036	NW_016019663.1	-	3760730	3761337	323	Exonic
novel_circ_002825	LOC108002150	NW_016019663.1	-	3894933	3904560	7550	Exon–intron
novel_circ_002826	NA	NW_016019663.1	-	4089195	4090795	1601	Intergenic region
novel_circ_002827	LOC108002258	NW_016019666.1	-	236	1936	684	Exonic
novel_circ_002828	LOC108002258	NW_016019666.1	-	236	4374	1014	Exonic
novel_circ_002829	LOC108002262	NW_016019675.1	-	16996	18479	785	Exonic
novel_circ_002830	LOC108002262	NW_016019675.1	-	17653	18479	423	Exonic
novel_circ_002831	LOC108002313	NW_016019675.1	-	348136	348451	316	Exonic
novel_circ_002832	LOC108002298	NW_016019675.1	-	406536	407068	423	Exonic
novel_circ_002833	LOC108002298	NW_016019675.1	-	406536	408463	532	Exonic
novel_circ_002834	LOC108002298	NW_016019675.1	-	406536	409653	743	Exonic
novel_circ_002835	LOC108002278	NW_016019675.1	+	787560	787816	257	Exon–intron
novel_circ_002836	LOC108002323	NW_016019675.1	-	787689	787874	186	Exonic

novel_circ_002837	LOC108002323	NW_016019675.1	-	788030	789575	844	Exonic
novel_circ_002838	LOC108002310	NW_016019675.1	-	815656	816616	367	Exonic
novel_circ_002839	LOC108002339	NW_016019685.1	-	2994	3591	598	Antisense
novel_circ_002840	LOC108002344	NW_016019686.1	-	206281	207323	612	Exonic
novel_circ_002841	LOC108002344	NW_016019686.1	-	224925	250037	670	Exonic
novel_circ_002842	LOC108002344	NW_016019686.1	+	508494	508847	354	Antisense
novel_circ_002843	LOC108002344	NW_016019686.1	+	508494	508882	389	Antisense
novel_circ_002844	LOC108002344	NW_016019686.1	+	508494	508888	395	Antisense
novel_circ_002845	LOC108002361	NW_016019686.1	-	638732	639872	425	Exonic
novel_circ_002846	NA	NW_016019686.1	-	678047	689435	11389	Intergenic region
novel_circ_002847	LOC108002348	NW_016019686.1	+	706171	708110	1940	Antisense
novel_circ_002848	LOC108002348	NW_016019686.1	-	720170	720928	759	Exonic
novel_circ_002849	LOC108002350	NW_016019686.1	+	825859	827916	695	Exonic
novel_circ_002850	LOC108002373	NW_016019691.1	-	4853	7181	1092	Exonic
novel_circ_002851	LOC108002373	NW_016019691.1	-	5542	6122	404	Exonic
novel_circ_002852	LOC114578104	NW_016019697.1	-	183819	189121	2434	Exon–intron
novel_circ_002853	LOC114578104	NW_016019697.1	-	197391	201383	590	Exonic
novel_circ_002854	LOC108002378	NW_016019697.1	-	244347	244964	307	Exonic
novel_circ_002855	LOC108002378	NW_016019697.1	-	249405	258321	621	Exonic
novel_circ_002856	LOC108002378	NW_016019697.1	-	254989	255522	272	Exonic
novel_circ_002857	LOC108002378	NW_016019697.1	-	254989	258321	448	Exonic
novel_circ_002858	LOC108002378	NW_016019697.1	-	274257	336348	626	Exonic
novel_circ_002859	LOC108002378	NW_016019697.1	-	305661	308457	270	Exonic
novel_circ_002860	LOC108002378	NW_016019697.1	-	305661	315950	404	Exonic
novel_circ_002861	LOC108002378	NW_016019697.1	-	305661	336348	522	Exonic
novel_circ_002862	LOC108002378	NW_016019697.1	-	308360	336348	350	Exonic
novel_circ_002863	LOC108002378	NW_016019697.1	-	315817	336348	252	Exonic
novel_circ_002864	LOC108002405	NW_016019697.1	+	497701	504590	6277	Exon–intron
novel_circ_002865	LOC108002405	NW_016019697.1	+	499409	504590	4569	Exon–intron
novel_circ_002866	LOC108002406	NW_016019697.1	-	567610	567759	150	Exonic
novel_circ_002867	LOC108002442	NW_016019708.1	-	17149	43399	26251	Exon–intron
novel_circ_002868	LOC108002442	NW_016019708.1	-	18050	43399	25350	Exon–intron
novel_circ_002869	LOC108002442	NW_016019708.1	-	29270	30875	1606	Intronic
novel_circ_002870	LOC108002442	NW_016019708.1	-	29270	31886	2617	Intronic
novel_circ_002871	LOC108002442	NW_016019708.1	-	29270	43399	14130	Intronic
novel_circ_002872	LOC108002419	NW_016019708.1	+	412157	413131	975	Intronic
novel_circ_002873	LOC108002419	NW_016019708.1	+	559165	559998	360	Exonic
novel_circ_002874	LOC108002419	NW_016019708.1	+	559165	561549	1002	Exonic
novel_circ_002875	LOC108002419	NW_016019708.1	+	559165	577893	15613	Exon–intron
novel_circ_002876	LOC108002419	NW_016019708.1	+	559165	578074	15794	Exon–intron
novel_circ_002877	LOC108002419	NW_016019708.1	+	560908	561549	642	Exonic
novel_circ_002878	LOC108002419	NW_016019708.1	+	560908	564405	1765	Exonic
novel_circ_002879	LOC108002419	NW_016019708.1	+	573699	577893	4195	Intronic
novel_circ_002880	LOC108002419	NW_016019708.1	+	573699	577897	4199	Intronic
novel_circ_002881	LOC108002419	NW_016019708.1	+	573699	578074	4376	Intronic
novel_circ_002882	LOC108002419	NW_016019708.1	+	577672	582121	1818	Exon–intron
novel_circ_002883	LOC108002428	NW_016019708.1	+	677945	678321	151	Exonic
novel_circ_002884	LOC108002492	NW_016019719.1	-	367187	367511	325	Exonic
novel_circ_002885	LOC108002491	NW_016019719.1	-	497548	498556	649	Exonic

novel_circ_002886	LOC108002477	NW_016019719.1	-	513522	523041	9033	Exon–intron
novel_circ_002887	LOC108002477	NW_016019719.1	-	513669	523041	8886	Exon–intron
novel_circ_002888	LOC108002500	NW_016019719.1	+	558769	559576	808	Exon–intron
novel_circ_002889	LOC108002465	NW_016019719.1	-	576042	576504	463	Exonic
novel_circ_002890	LOC108002448	NW_016019719.1	+	651171	651822	552	Exonic
novel_circ_002891	LOC108002448	NW_016019719.1	+	651171	652432	711	Exonic
novel_circ_002892	LOC108002459	NW_016019719.1	+	660523	661088	248	Exonic
novel_circ_002893	LOC108002505	NW_016019721.1	-	5434	6521	869	Exonic
novel_circ_002894	LOC108002511	NW_016019727.1	-	3797	4608	384	Exonic
novel_circ_002895	LOC108002523	NW_016019730.1	-	291811	312322	842	Exonic
novel_circ_002896	LOC108002523	NW_016019730.1	-	352305	374398	648	Exonic
novel_circ_002897	LOC108002523	NW_016019730.1	-	352305	391425	996	Exonic
novel_circ_002898	LOC108002523	NW_016019730.1	-	388128	391425	348	Exonic
novel_circ_002899	LOC114578114	NW_016019730.1	-	658095	660391	760	Exonic
novel_circ_002900	LOC108002515	NW_016019730.1	-	729995	737871	3450	Exon–intron
novel_circ_002901	LOC108002515	NW_016019730.1	-	735187	736003	817	Exonic
novel_circ_002902	LOC108002536	NW_016019738.1	+	3247	3833	307	Exonic
novel_circ_002903	LOC108002567	NW_016019741.1	+	47546	48745	391	Exonic
novel_circ_002904	LOC108002567	NW_016019741.1	-	48684	50033	1350	Antisense
novel_circ_002905	LOC108002567	NW_016019741.1	+	49315	50056	500	Exonic
novel_circ_002906	LOC108002566	NW_016019741.1	-	100139	106554	303	Exonic
novel_circ_002907	LOC108002555	NW_016019741.1	+	176329	178218	1890	Exon–intron
novel_circ_002908	LOC108002562	NW_016019741.1	-	357265	358974	1710	Exonic
novel_circ_002909	LOC108002595	NW_016019752.1	-	309497	309651	155	Exonic
novel_circ_002910	LOC108002604	NW_016019752.1	-	434193	448672	12890	Exon–intron
novel_circ_002911	LOC108002611	NW_016019762.1	-	5255	7116	1247	Exon–intron
novel_circ_002912	LOC108002665	NW_016019763.1	+	439193	441634	2184	Exonic
novel_circ_002913	LOC108002631	NW_016019763.1	-	557525	557944	352	Exonic
novel_circ_002914	LOC108002638	NW_016019763.1	-	579018	579620	352	Exonic
novel_circ_002915	LOC108002811	NW_016019774.1	+	166914	167254	341	Exonic
novel_circ_002916	LOC108002769	NW_016019774.1	+	315203	318615	3413	Intronic
novel_circ_002917	LOC108002769	NW_016019774.1	+	315203	324691	9489	Exon–intron
novel_circ_002918	LOC108002769	NW_016019774.1	+	315203	329791	10715	Exon–intron
novel_circ_002919	LOC108002769	NW_016019774.1	+	352366	360334	388	Exonic
novel_circ_002920	LOC108002769	NW_016019774.1	+	352366	361373	735	Exonic
novel_circ_002921	LOC108002769	NW_016019774.1	+	359755	360334	259	Exonic
novel_circ_002922	LOC108002769	NW_016019774.1	+	360145	361373	537	Exonic
novel_circ_002923	LOC108002769	NW_016019774.1	+	360145	365001	876	Exonic
novel_circ_002924	LOC108002769	NW_016019774.1	+	360145	365280	1155	Exon–intron
novel_circ_002925	LOC108002769	NW_016019774.1	+	361027	365280	965	Exon–intron
novel_circ_002926	LOC108002769	NW_016019774.1	+	363085	365280	618	Exon–intron
novel_circ_002927	LOC108002715	NW_016019774.1	-	397532	398254	723	Exonic
novel_circ_002928	LOC108002709	NW_016019774.1	+	410099	410451	353	Exonic
novel_circ_002929	LOC108002713	NW_016019774.1	-	503496	503817	322	Exonic
novel_circ_002930	LOC108002713	NW_016019774.1	-	503496	504431	486	Exonic
novel_circ_002931	LOC108002713	NW_016019774.1	-	503496	505857	825	Exonic
novel_circ_002932	LOC108002713	NW_016019774.1	-	503667	504374	258	Exonic
novel_circ_002933	LOC108002713	NW_016019774.1	-	504268	505857	503	Exonic
novel_circ_002934	LOC108002928	NW_016019774.1	+	638479	639287	547	Exonic

novel_circ_002935	LOC108002907	NW_016019774.1	+	672199	673243	204	Exonic
novel_circ_002936	LOC108002833	NW_016019774.1	-	683961	687025	1832	Exonic
novel_circ_002937	LOC108002836	NW_016019774.1	-	895400	904590	9191	Antisense
novel_circ_002938	LOC108002836	NW_016019774.1	+	1211048	1211465	347	Exonic
novel_circ_002939	LOC108002686	NW_016019774.1	-	1268278	1269107	449	Exonic
novel_circ_002940	LOC108002686	NW_016019774.1	-	1280886	1282030	312	Exonic
novel_circ_002941	LOC108002826	NW_016019774.1	-	1351935	1354433	515	Exonic
novel_circ_002942	LOC108002826	NW_016019774.1	-	1355281	1356249	395	Exonic
novel_circ_002943	LOC108002801	NW_016019774.1	+	1548931	1551628	1177	Exonic
novel_circ_002944	LOC108002801	NW_016019774.1	+	1548931	1583613	2745	Exonic
novel_circ_002945	LOC108002801	NW_016019774.1	-	1550084	1551460	1377	Antisense
novel_circ_002946	LOC108002801	NW_016019774.1	+	1581069	1581760	443	Exonic
novel_circ_002947	LOC108002801	NW_016019774.1	+	1581069	1583613	857	Exonic
novel_circ_002948	LOC108002801	NW_016019774.1	+	1583200	1585481	1042	Exonic
novel_circ_002949	LOC108002801	NW_016019774.1	+	1583997	1584420	424	Exonic
novel_circ_002950	LOC108002801	NW_016019774.1	+	1583997	1585481	628	Exonic
novel_circ_002951	LOC108002801	NW_016019774.1	+	1586667	1588508	1530	Exonic
novel_circ_002952	LOC108002801	NW_016019774.1	+	1589072	1589822	549	Exonic
novel_circ_002953	LOC108002801	NW_016019774.1	-	1592886	1593258	373	Antisense
novel_circ_002954	LOC108002871	NW_016019774.1	+	1798822	1800105	694	Exonic
novel_circ_002955	LOC108002794	NW_016019774.1	+	1818597	1829054	10458	Exon–intron
novel_circ_002956	LOC108002793	NW_016019774.1	+	1820742	1820952	211	Antisense
novel_circ_002957	LOC108002792	NW_016019774.1	+	1845021	1845514	494	Exonic
novel_circ_002958	LOC108002792	NW_016019774.1	+	1909731	1910324	594	Exonic
novel_circ_002959	LOC108002792	NW_016019774.1	+	1909731	1912064	651	Exonic
novel_circ_002960	LOC108002824	NW_016019774.1	+	1965787	1967538	1184	Exonic
novel_circ_002961	LOC108002824	NW_016019774.1	+	1966570	1967538	721	Exonic
novel_circ_002962	LOC108002821	NW_016019774.1	-	1970673	1972181	783	Exonic
novel_circ_002963	LOC108002693	NW_016019774.1	-	1994899	1996783	607	Exonic
novel_circ_002964	LOC108002693	NW_016019774.1	-	1995493	1996783	411	Exonic
novel_circ_002965	LOC108002693	NW_016019774.1	-	2025704	2039333	100	Exonic
novel_circ_002966	LOC108002752	NW_016019774.1	-	2104749	2106416	713	Exonic
novel_circ_002967	LOC108002754	NW_016019774.1	+	2138099	2140296	453	Exonic
novel_circ_002968	LOC108002747	NW_016019774.1	+	2193956	2194630	379	Exon–intron
novel_circ_002969	LOC108002696	NW_016019774.1	+	2351073	2352042	970	Exon–intron
novel_circ_002970	LOC108002696	NW_016019774.1	+	2352494	2359550	6533	Exon–intron
novel_circ_002971	LOC108002696	NW_016019774.1	+	2358584	2359550	443	Exonic
novel_circ_002972	LOC108002696	NW_016019774.1	+	2359203	2359550	348	Exonic
novel_circ_002973	LOC108002696	NW_016019774.1	+	2372525	2373533	512	Exonic
novel_circ_002974	LOC108002696	NW_016019774.1	+	2372525	2373566	545	Exonic
novel_circ_002975	LOC108002696	NW_016019774.1	+	2372561	2373566	509	Exonic
novel_circ_002976	LOC108002865	NW_016019774.1	-	2410507	2419864	397	Exonic
novel_circ_002977	LOC108002698	NW_016019774.1	+	2682716	2691498	8411	Exon–intron
novel_circ_002978	LOC108002698	NW_016019774.1	+	2690705	2691498	422	Exonic
novel_circ_002979	LOC108002829	NW_016019774.1	+	2720132	2722645	2387	Exonic
novel_circ_002980	LOC108002839	NW_016019774.1	+	2775449	2777805	450	Exonic
novel_circ_002981	LOC108002839	NW_016019774.1	+	2775449	2779915	930	Exonic
novel_circ_002982	LOC108002839	NW_016019774.1	+	2776904	2779915	710	Exonic
novel_circ_002983	LOC108002839	NW_016019774.1	+	2779251	2779653	270	Exonic

novel_circ_002984	LOC108002839	NW_016019774.1	+	2779251	2779915	432	Exonic
novel_circ_002985	LOC108002701	NW_016019774.1	-	2807374	2807559	186	Exonic
novel_circ_002986	LOC108002701	NW_016019774.1	-	2807386	2808036	651	Exonic
novel_circ_002987	LOC108002701	NW_016019774.1	-	2814404	2815315	467	Exonic
novel_circ_002988	LOC108002841	NW_016019774.1	-	2872356	2877832	4673	Exon–intron
novel_circ_002989	LOC108002841	NW_016019774.1	-	2872356	2878940	5115	Exon–intron
novel_circ_002990	LOC108002841	NW_016019774.1	-	2872356	2879024	5199	Exon–intron
novel_circ_002991	LOC108002841	NW_016019774.1	-	2872401	2878940	5070	Exon–intron
novel_circ_002992	LOC108002841	NW_016019774.1	-	2872776	2878940	4695	Exon–intron
novel_circ_002993	LOC108002841	NW_016019774.1	-	2892002	2896886	579	Exonic
novel_circ_002994	LOC108002841	NW_016019774.1	-	2893203	2896886	425	Exonic
novel_circ_002995	LOC108002843	NW_016019774.1	-	2953684	2958371	4201	Exonic
novel_circ_002996	LOC108002703	NW_016019774.1	-	2992572	2993799	766	Exonic
novel_circ_002997	LOC108002703	NW_016019774.1	-	2992572	2996983	892	Exonic
novel_circ_002998	LOC108002703	NW_016019774.1	-	3009014	3009466	453	Exonic
novel_circ_002999	LOC108002845	NW_016019774.1	-	3079722	3086029	4354	Exonic
novel_circ_003000	LOC108002845	NW_016019774.1	-	3082603	3084311	453	Exonic
novel_circ_003001	LOC108002845	NW_016019774.1	-	3084132	3086029	1396	Exonic
novel_circ_003002	LOC108002845	NW_016019774.1	-	3084677	3086029	1216	Exonic
novel_circ_003003	LOC108002845	NW_016019774.1	-	3108724	3111683	2960	Intronic
novel_circ_003004	LOC108002797	NW_016019774.1	-	3152606	3153537	775	Exon–intron
novel_circ_003005	LOC108002797	NW_016019774.1	-	3163391	3164893	1005	Exonic
novel_circ_003006	LOC108002797	NW_016019774.1	+	3163441	3168110	4670	Antisense
novel_circ_003007	LOC108002704	NW_016019774.1	-	3173859	3174122	264	Exonic
novel_circ_003008	LOC108002876	NW_016019774.1	-	3340455	3340769	315	Antisense
novel_circ_003009	LOC108002904	NW_016019774.1	+	3367489	3367647	159	Exonic
novel_circ_003010	LOC108002806	NW_016019774.1	-	3550423	3551019	597	Exonic
novel_circ_003011	LOC108002806	NW_016019774.1	-	3555994	3563870	345	Exonic
novel_circ_003012	LOC108002805	NW_016019774.1	+	3601714	3602788	901	Exonic
novel_circ_003013	LOC108002818	NW_016019774.1	+	3657902	3658302	331	Exonic
novel_circ_003014	LOC108002819	NW_016019774.1	-	3684658	3685080	361	Exonic
novel_circ_003015	LOC108002968	NW_016019775.1	-	174068	174839	490	Exonic
novel_circ_003016	LOC108002961	NW_016019775.1	-	382757	384968	2212	Antisense
novel_circ_003017	LOC108002978	NW_016019781.1	+	4622	5057	340	Exonic
novel_circ_003018	LOC108003022	NW_016019786.1	-	107119	107280	162	Exonic
novel_circ_003019	LOC108002990	NW_016019786.1	+	286100	287043	944	Antisense
novel_circ_003020	LOC108003017	NW_016019786.1	-	429639	431788	1746	Exonic
novel_circ_003021	LOC108003017	NW_016019786.1	-	430688	430952	265	Exonic
novel_circ_003022	LOC108003018	NW_016019786.1	-	447906	448473	470	Exonic
novel_circ_003023	LOC108003018	NW_016019786.1	-	447906	456047	2280	Exonic
novel_circ_003024	LOC108003018	NW_016019786.1	-	453885	456047	1810	Exonic
novel_circ_003025	LOC108003018	NW_016019786.1	-	455606	456047	442	Exonic
novel_circ_003026	LOC108003018	NW_016019786.1	+	456473	457476	1004	Antisense
novel_circ_003027	LOC108003004	NW_016019786.1	-	597138	597511	159	Exon–intron
novel_circ_003028	LOC108003004	NW_016019786.1	-	597139	597508	155	Exonic
novel_circ_003029	LOC108003004	NW_016019786.1	-	597139	597511	158	Exonic
novel_circ_003030	LOC108003004	NW_016019786.1	-	597139	597515	162	Exonic
novel_circ_003031	LOC108003004	NW_016019786.1	-	597458	598038	189	Exonic
novel_circ_003032	LOC108003038	NW_016019786.1	+	620937	623034	453	Exonic

novel_circ_003033	LOC108003038	NW_016019786.1	+	620985	623034	405	Exonic
novel_circ_003034	LOC108003038	NW_016019786.1	+	622409	631045	1168	Exonic
novel_circ_003035	LOC108002989	NW_016019786.1	+	754184	757583	402	Exonic
novel_circ_003036	LOC108003080	NW_016019791.1	-	3160	6000	2841	Antisense
novel_circ_003037	LOC108003145	NW_016019808.1	+	35052	36908	815	Exonic
novel_circ_003038	LOC108003128	NW_016019808.1	+	49320	51393	337	Exonic
novel_circ_003039	LOC108003128	NW_016019808.1	+	49408	53448	1101	Exonic
novel_circ_003040	LOC108003130	NW_016019808.1	+	51250	133921	78240	Exon–intron
novel_circ_003041	LOC108003128	NW_016019808.1	+	51274	51786	249	Exonic
novel_circ_003042	LOC108003128	NW_016019808.1	-	51746	52523	778	Antisense
novel_circ_003043	LOC108003128	NW_016019808.1	-	51893	52493	601	Antisense
novel_circ_003044	LOC108003128	NW_016019808.1	-	51951	52488	538	Antisense
novel_circ_003045	LOC108003128	NW_016019808.1	+	52485	53442	370	Exonic
novel_circ_003046	LOC108003146	NW_016019808.1	+	241255	243126	980	Exonic
novel_circ_003047	LOC108003146	NW_016019808.1	+	241255	246086	1247	Exonic
novel_circ_003048	LOC108003146	NW_016019808.1	+	241255	248994	1496	Exonic
novel_circ_003049	LOC108003146	NW_016019808.1	+	245820	248994	516	Exonic
novel_circ_003050	LOC108003141	NW_016019808.1	+	362071	364374	598	Exonic
novel_circ_003051	LOC108003141	NW_016019808.1	+	363341	370416	1151	Exonic
novel_circ_003052	LOC108003125	NW_016019808.1	-	541209	542447	742	Exonic
novel_circ_003053	LOC108003125	NW_016019808.1	-	547876	555101	2112	Exonic
novel_circ_003054	LOC108003127	NW_016019808.1	+	579483	580312	771	Exonic
novel_circ_003055	LOC114578168	NW_016019808.1	+	609987	630732	12802	Exon–intron
novel_circ_003056	LOC114578168	NW_016019808.1	+	609987	630996	13066	Exon–intron
novel_circ_003057	LOC108003171	NW_016019819.1	-	160024	162168	2145	Antisense
novel_circ_003058	LOC108003167	NW_016019819.1	-	442469	457362	12855	Exon–intron
novel_circ_003059	LOC108003167	NW_016019819.1	-	442469	457702	13195	Exon–intron
novel_circ_003060	LOC108003176	NW_016019819.1	-	587775	588400	438	Exonic
novel_circ_003061	LOC108003197	NW_016019841.1	+	319507	321787	367	Exonic
novel_circ_003062	LOC108003197	NW_016019841.1	+	319507	322226	557	Exonic
novel_circ_003063	LOC108003197	NW_016019841.1	+	537450	544381	291	Exonic
novel_circ_003064	LOC108003197	NW_016019841.1	+	537450	572007	27917	Exon–intron
novel_circ_003065	LOC108003197	NW_016019841.1	+	597521	600007	1617	Exonic
novel_circ_003066	LOC108003198	NW_016019841.1	-	680648	681859	523	Exonic
novel_circ_003067	NA	NW_016019841.1	-	693796	694049	254	Intergenic region
novel_circ_003068	LOC108003229	NW_016019852.1	+	24817	27244	702	Exonic
novel_circ_003069	LOC108003229	NW_016019852.1	+	24817	47024	20482	Exon–intron
novel_circ_003070	LOC108003247	NW_016019852.1	-	94589	99230	2790	Exon–intron
novel_circ_003071	LOC108003247	NW_016019852.1	-	94907	97492	1478	Exonic
novel_circ_003072	LOC108003247	NW_016019852.1	-	95223	97492	1290	Exonic
novel_circ_003073	LOC108003247	NW_016019852.1	-	96907	121050	3763	Exonic
novel_circ_003074	LOC108003259	NW_016019852.1	-	219031	223726	508	Exonic
novel_circ_003075	LOC108003259	NW_016019852.1	-	220726	223726	339	Exonic
novel_circ_003076	LOC108003254	NW_016019852.1	-	651230	652304	644	Exonic
novel_circ_003077	LOC108003256	NW_016019852.1	+	688257	689062	806	Antisense
novel_circ_003078	LOC108003256	NW_016019852.1	+	688272	689000	729	Antisense
novel_circ_003079	LOC108003301	NW_016019863.1	+	92001	92188	188	Exon–intron
novel_circ_003080	LOC108003297	NW_016019863.1	+	102105	102586	401	Exon–intron
novel_circ_003081	LOC108003297	NW_016019863.1	-	105391	105809	419	Antisense

novel_circ_003082	LOC108003297	NW_016019863.1	-	105749	106021	273	Antisense
novel_circ_003083	LOC108003297	NW_016019863.1	+	105946	106616	452	Exonic
novel_circ_003084	LOC108003297	NW_016019863.1	-	106576	107002	427	Antisense
novel_circ_003085	LOC108003328	NW_016019863.1	+	137310	145720	7759	Exon–intron
novel_circ_003086	LOC108003328	NW_016019863.1	+	137310	147064	9103	Exon–intron
novel_circ_003087	LOC108003328	NW_016019863.1	+	137310	147878	9917	Exon–intron
novel_circ_003088	LOC108003328	NW_016019863.1	+	137310	155063	1669	Exon–intron
novel_circ_003089	LOC108003317	NW_016019863.1	+	218958	221806	2849	Exon–intron
novel_circ_003090	LOC108003317	NW_016019863.1	+	221236	221806	571	Exonic
novel_circ_003091	LOC108003317	NW_016019863.1	+	221236	223014	773	Exonic
novel_circ_003092	LOC108003317	NW_016019863.1	+	221236	251682	1042	Exonic
novel_circ_003093	LOC108003317	NW_016019863.1	+	251414	251682	269	Exonic
novel_circ_003094	LOC108003334	NW_016019863.1	+	306657	308299	479	Exonic
novel_circ_003095	LOC108003308	NW_016019863.1	+	460020	460644	437	Exonic
novel_circ_003096	LOC108003308	NW_016019863.1	-	460090	460699	610	Antisense
novel_circ_003097	LOC108003308	NW_016019863.1	+	460384	460849	466	Exonic
novel_circ_003098	LOC108003308	NW_016019863.1	-	460509	461268	760	Antisense
novel_circ_003099	LOC108003308	NW_016019863.1	-	460582	460845	264	Antisense
novel_circ_003100	LOC108003308	NW_016019863.1	+	460585	461318	534	Exonic
novel_circ_003101	LOC108003308	NW_016019863.1	+	460619	460867	249	Exonic
novel_circ_003102	LOC108003308	NW_016019863.1	+	460642	460842	201	Exonic
novel_circ_003103	LOC108003308	NW_016019863.1	-	460642	460842	201	Antisense
novel_circ_003104	LOC108003308	NW_016019863.1	-	460679	460909	231	Antisense
novel_circ_003105	LOC108003308	NW_016019863.1	+	460754	461869	647	Exonic
novel_circ_003106	LOC108003308	NW_016019863.1	-	460805	462059	1255	Antisense
novel_circ_003107	LOC108003308	NW_016019863.1	+	460808	461356	349	Exonic
novel_circ_003108	LOC108003308	NW_016019863.1	-	460808	461367	560	Antisense
novel_circ_003109	LOC108003308	NW_016019863.1	+	460808	461426	419	Exonic
novel_circ_003110	LOC108003308	NW_016019863.1	+	460834	461330	297	Exonic
novel_circ_003111	LOC108003308	NW_016019863.1	+	460834	461339	306	Exonic
novel_circ_003112	LOC108003308	NW_016019863.1	-	460843	461316	474	Antisense
novel_circ_003113	LOC108003308	NW_016019863.1	+	460843	461819	508	Exonic
novel_circ_003114	LOC108003308	NW_016019863.1	+	460843	462005	694	Exonic
novel_circ_003115	LOC108003308	NW_016019863.1	-	460847	461316	470	Antisense
novel_circ_003116	LOC108003308	NW_016019863.1	+	460847	462486	1171	Exonic
novel_circ_003117	LOC108003308	NW_016019863.1	+	460847	462604	1289	Exonic
novel_circ_003118	LOC108003308	NW_016019863.1	+	460856	461310	255	Exonic
novel_circ_003119	LOC108003308	NW_016019863.1	+	460856	461322	267	Exonic
novel_circ_003120	LOC108003308	NW_016019863.1	+	460856	461753	429	Exonic
novel_circ_003121	LOC108003308	NW_016019863.1	+	460859	461310	252	Exonic
novel_circ_003122	LOC108003308	NW_016019863.1	-	460859	461315	457	Antisense
novel_circ_003123	LOC108003308	NW_016019863.1	+	460859	461315	257	Exonic
novel_circ_003124	LOC108003308	NW_016019863.1	-	460859	461316	458	Antisense
novel_circ_003125	LOC108003308	NW_016019863.1	+	460859	461318	260	Exonic
novel_circ_003126	LOC108003308	NW_016019863.1	-	460859	461327	469	Antisense
novel_circ_003127	LOC108003308	NW_016019863.1	+	460859	461330	272	Exonic
novel_circ_003128	LOC108003308	NW_016019863.1	+	460859	461339	281	Exonic
novel_circ_003129	LOC108003308	NW_016019863.1	+	460859	461767	440	Exonic
novel_circ_003130	LOC108003308	NW_016019863.1	-	460862	461310	449	Antisense

novel_circ_003131	LOC108003308	NW_016019863.1	+	460862	461343	282	Exonic
novel_circ_003132	LOC108003308	NW_016019863.1	+	460865	461318	254	Exonic
novel_circ_003133	LOC108003308	NW_016019863.1	+	460871	461318	248	Exonic
novel_circ_003134	LOC108003308	NW_016019863.1	-	460910	461397	488	Antisense
novel_circ_003135	LOC108003308	NW_016019863.1	+	461368	462002	366	Exonic
novel_circ_003136	LOC108003308	NW_016019863.1	-	461740	462052	313	Antisense
novel_circ_003137	LOC108003308	NW_016019863.1	-	461753	462484	732	Antisense
novel_circ_003138	LOC108003308	NW_016019863.1	+	461779	462059	281	Exonic
novel_circ_003139	LOC108003308	NW_016019863.1	+	461779	462455	677	Exonic
novel_circ_003140	LOC108003308	NW_016019863.1	+	461779	462535	757	Exonic
novel_circ_003141	LOC108003308	NW_016019863.1	-	461787	462455	669	Antisense
novel_circ_003142	LOC108003308	NW_016019863.1	-	461808	462059	252	Antisense
novel_circ_003143	LOC108003308	NW_016019863.1	-	461816	462456	641	Antisense
novel_circ_003144	LOC108003308	NW_016019863.1	+	461822	462455	634	Exonic
novel_circ_003145	LOC108003308	NW_016019863.1	-	461822	462455	634	Antisense
novel_circ_003146	LOC108003308	NW_016019863.1	+	461825	462455	631	Exonic
novel_circ_003147	LOC108003308	NW_016019863.1	-	461825	462455	631	Antisense
novel_circ_003148	LOC108003308	NW_016019863.1	-	461825	462486	662	Antisense
novel_circ_003149	LOC108003308	NW_016019863.1	-	461832	462449	618	Antisense
novel_circ_003150	LOC108003308	NW_016019863.1	+	461832	462455	624	Exonic
novel_circ_003151	LOC108003308	NW_016019863.1	-	461832	462455	624	Antisense
novel_circ_003152	LOC108003308	NW_016019863.1	-	461837	462449	613	Antisense
novel_circ_003153	LOC108003308	NW_016019863.1	-	461940	462908	969	Antisense
novel_circ_003154	LOC108003308	NW_016019863.1	+	461960	462486	527	Exonic
novel_circ_003155	LOC108003308	NW_016019863.1	-	461973	462455	483	Antisense
novel_circ_003156	LOC108003308	NW_016019863.1	-	461995	462493	499	Antisense
novel_circ_003157	LOC108003308	NW_016019863.1	-	462044	462455	412	Antisense
novel_circ_003158	LOC108003308	NW_016019863.1	+	462044	462455	412	Exonic
novel_circ_003159	LOC108003308	NW_016019863.1	-	462054	462449	396	Antisense
novel_circ_003160	LOC108003308	NW_016019863.1	-	462060	462458	399	Antisense
novel_circ_003161	LOC108003308	NW_016019863.1	-	462066	462449	384	Antisense
novel_circ_003162	LOC108003308	NW_016019863.1	+	462208	462978	771	Exonic
novel_circ_003163	LOC108003318	NW_016019863.1	-	645895	647150	861	Exonic
novel_circ_003164	LOC108003363	NW_016019874.1	-	23961	24771	386	Exonic
novel_circ_003165	LOC108003380	NW_016019874.1	-	139275	139633	264	Exonic
novel_circ_003166	LOC108003378	NW_016019874.1	+	152314	152566	168	Exon–intron
novel_circ_003167	LOC108003406	NW_016019874.1	-	187636	199842	9575	Exon–intron
novel_circ_003168	LOC108003416	NW_016019874.1	-	374204	384213	964	Exonic
novel_circ_003169	LOC108003416	NW_016019874.1	-	380579	381812	550	Exonic
novel_circ_003170	LOC108003416	NW_016019874.1	-	380579	384213	801	Exonic
novel_circ_003171	LOC108003419	NW_016019874.1	+	459246	459794	253	Exonic
novel_circ_003172	LOC108003370	NW_016019874.1	+	487216	487974	643	Exonic
novel_circ_003173	LOC108003395	NW_016019874.1	+	564837	567967	1784	Exonic
novel_circ_003174	LOC108003383	NW_016019874.1	+	598638	603426	2463	Exonic
novel_circ_003175	LOC108003383	NW_016019874.1	+	620180	621960	363	Exonic
novel_circ_003176	LOC108003385	NW_016019874.1	-	659700	663096	1888	Exon–intron
novel_circ_003177	LOC108003385	NW_016019874.1	-	659700	663868	2660	Exon–intron
novel_circ_003178	LOC108003385	NW_016019874.1	-	659700	664271	3063	Exon–intron

Table S4A. DEcircRNAs in the AcCK1 vs AcT1 comparison group

CircRNA ID	AcCK1_count	AcT1_count	AcCK1 RPM	AcT1 RPM	log2(Fold change)	*P* value	FDR	Significant	Source gene ID	Chromosome	Strand	Genomic start	Genomic end	Length	Type
novel_circ_000015	6	0	210.8518414	0.001	-17.6858701	0.03127344	0.392499239	yes	LOC108002084	NW_016017455.1	-	893544	895102	386	annot_exons
novel_circ_000050	9	0	316.277622	0.001	-18.2708326	0.003913285	0.098304236	yes	LOC108003653	NW_016017455.1	-	2583353	2584739	1387	exon_intron
novel_circ_000051	1370	2807	48144.5038	102422.8271	1.089094273	2.78E-107	6.22E-104	yes	LOC108003695	NW_016017455.1	+	2661045	2670682	9161	exon_intron
novel_circ_000091	26	8	913.6913129	291.9068817	-1.646198616	0.002958466	0.07775546	yes	LOC114577180	NW_016017456.1	+	198678	200971	2294	exon_intron
novel_circ_000093	22	4	773.1234186	145.9534409	-2.405190516	0.000537698	0.022244768	yes	NA	NW_016017456.1	-	407476	417110	9635	intergenic
novel_circ_000102	26	11	913.6913129	401.3719623	-1.186766997	0.020187194	0.311783718	yes	LOC107995988	NW_016017456.1	+	1404383	1406972	350	annot_exons
novel_circ_000114	6	0	210.8518414	0.001	-17.6858701	0.03127344	0.392499239	yes	LOC107995475	NW_016017456.1	+	2031624	2034449	2273	annot_exons
novel_circ_000129	0	10	0.001	364.8836021	18.47707679	0.001957522	0.057243404	yes	LOC107994102	NW_016017456.1	-	3156540	3157108	443	annot_exons
novel_circ_000133	33	13	1159.685128	474.3486828	-1.289713299	0.006668724	0.152019679	yes	LOC107994792	NW_016017456.1	+	3482166	3485591	1915	annot_exons
novel_circ_000182	23	7	808.2653922	255.4185215	-1.661965932	0.005255641	0.124905333	yes	LOC107999178	NW_016017479.1	-	358910	359324	415	one_exon
novel_circ_000186	6	0	210.8518414	0.001	-17.6858701	0.03127344	0.392499239	yes	LOC107998870	NW_016017479.1	-	628431	628957	406	annot_exons
novel_circ_000255	8	0	281.1357886	0.001	-18.10090759	0.007823441	0.159758136	yes	LOC108003569	NW_016017567.1	-	1581187	1587589	1635	annot_exons
novel_circ_000278	8	0	281.1357886	0.001	-18.10090759	0.007823441	0.159758136	yes	LOC108003479	NW_016017567.1	-	2560409	2560768	360	one_exon
novel_circ_000284	16	5	562.2715772	182.4418011	-1.623830803	0.026681361	0.379657071	yes	LOC108003597	NW_016017567.1	+	3150889	3151348	366	annot_exons
novel_circ_000331	11	2	386.5617093	72.97672043	-2.405190516	0.022502846	0.326437384	yes	LOC108003857	NW_016017623.1	+	291902	293075	442	annot_exons
novel_circ_000350	7	0	245.993815	0.001	-17.90826252	0.01564141	0.260767977	yes	LOC108003872	NW_016017634.1	-	526296	546338	598	annot_exons
novel_circ_000353	17	41	597.4135507	1496.022769	1.324330266	0.001571551	0.050154927	yes	LOC108003872	NW_016017634.1	-	533754	546338	317	annot_exons
novel_circ_000364	11	0	386.5617093	0.001	-18.56033921	0.00097925	0.034222525	yes	LOC108003950	NW_016017656.1	+	326358	333294	6389	exon_intron
novel_circ_000445	17	3	597.4135507	109.4650806	-2.448259238	0.002588858	0.069680827	yes	LOC108004244	NW_016017745.1	+	13027	14330	573	annot_exons
novel_circ_000515	11	28	386.5617093	1021.674086	1.402164406	0.009543715	0.185397042	yes	LOC107992469	NW_016017823.1	-	336809	340175	2601	annot_exons
novel_circ_000518	2	11	70.28394715	401.3719623	2.513672721	0.022502846	0.326437384	yes	LOC107992482	NW_016017823.1	+	395758	396198	331	annot_exons
novel_circ_000562	534	246	18765.81389	8976.136612	-1.063940324	3.71E-22	2.07E-19	yes	NA	NW_016017900.1	-	605032	605750	719	intergenic
novel_circ_000575	17	6	597.4135507	218.9301613	-1.448259238	0.034791993	0.431807293	yes	LOC107992818	NW_016017900.1	-	1369198	1372070	348	annot_exons
novel_circ_000594	17	5	597.4135507	182.4418011	-1.711293644	0.016959285	0.27654775	yes	LOC107992891	NW_016017901.1	+	163583	165990	2408	one_exon
novel_circ_000599	58	24	2038.234467	875.7206451	-1.218777392	0.000548662	0.022285635	yes	LOC107992899	NW_016017912.1	+	349132	350982	1779	exon_intron
novel_circ_000602	31	6	1089.401181	218.9301613	-2.314992707	4.19E-05	0.002673811	yes	NA	NW_016017945.1	-	21302	21607	306	intergenic
novel_circ_000627	3	16	105.4259207	583.8137634	2.469278602	0.004442811	0.106723015	yes	LOC107992960	NW_016017956.1	+	172365	172685	321	one_exon
novel_circ_000651	0	10	0.001	364.8836021	18.47707679	0.001957522	0.057243404	yes	LOC107992960	NW_016017956.1	+	218340	220412	425	annot_exons
novel_circ_000	77	18	2705.9319	656.79048	-2.04262	3.25E-09	6.05E-07	yes	LOC107993	NW_01601823	-	56217	57401	1185	intronic

962			65	38	0437				804	3.1					
novel_circ_000983	23	8	808.2653922	291.9068817	-1.469320854	0.0107306	0.201492914	yes	LOC107993750	NW_016018233.1	-	753253	754446	444	annot_exons
novel_circ_001069	58	21	2038.234467	766.2555645	-1.41142247	0.000105781	0.006059357	yes	LOC107994172	NW_016018344.1	-	2135368	2146392	7228	exon_intron
novel_circ_001092	8	0	281.1357886	0.001	-18.10090759	0.00782341	0.159758136	yes	LOC107994268	NW_016018418.1	+	1548	2472	546	annot_exons
novel_circ_001106	0	10	0.001	364.8836021	18.47707679	0.00195752 2	0.057243404	yes	LOC107994399	NW_016018455.1	-	575024	575431	305	annot_exons
novel_circ_001142	9	1	316.2777622	36.48836021	-3.115683899	0.021515144	0.31831015	yes	LOC107994579	NW_016018511.1	+	115625	115950	326	antisense
novel_circ_001148	16	37	562.2715772	1350.069328	1.263694468	0.003885152	0.098304236	yes	LOC107994623	NW_016018533.1	-	109692	116206	6429	exon_intron
novel_circ_001175	13	27	456.8456565	985.1857258	1.108688886	0.038669498	0.459788861	yes	LOC107994726	NW_016018566.1	+	1972335	1972743	409	one_exon
novel_circ_001176	4	21	140.5678943	766.2555645	2.446558525	0.000916869	0.033578432	yes	LOC107994726	NW_016018566.1	+	1972335	1976168	578	annot_exons
novel_circ_001193	11	0	386.5617093	0.001	-18.56033921	0.00097925	0.034222525	yes	LOC107994823	NW_016018566.1	+	3586705	3587604	900	one_exon
novel_circ_001276	68	31	2389.654203	1131.139167	-1.079025429	0.000579873	0.023132772	yes	LOC107995057	NW_016018567.1	+	315572	324820	8586	exon_intron
novel_circ_001346	21	0	737.98145	0.001	-19.49322502	9.64E-07	9.79E-05	yes	LOC107995435	NW_016018716.1	+	1541	2040	500	one_exon
novel_circ_001350	0	8	0.001	291.9068817	18.1551487	0.00782341	0.159758136	yes	LOC107995527	NW_016018789.1	+	58278	60664	1653	annot_exons
novel_circ_001358	37	16	1300.253022	583.8137634	-1.155212263	0.007866336	0.159758136	yes	LOC107995486	NW_016018789.1	+	1285460	1293290	7277	exon_intron
novel_circ_001375	279	581	9804.610627	21199.73728	1.112514144	1.00E-26	7.48E-24	yes	LOC114576763	NW_016018823.1	-	27821	41027	13207	exon_intron
novel_circ_001420	19	1	667.6974979	36.48836021	-4.193686411	4.04E-05	0.002652103	yes	LOC107995892	NW_016019011.1	+	224087	224679	294	annot_exons
novel_circ_001447	6	0	210.8518414	0.001	-17.6858701	0.03127344	0.392499239	yes	LOC107996055	NW_016019053.1	+	123769	129096	4019	exon_intron
novel_circ_001453	10	0	351.4197357	0.001	-18.42283569	0.001957522	0.057243404	yes	LOC107996135	NW_016019053.1	+	692641	693478	411	annot_exons
novel_circ_001467	19	2	667.6974979	72.97672043	-3.193686411	0.000222778	0.010368469	yes	LOC107996152	NW_016019057.1	-	2593	6076	3482	annot_exons
novel_circ_001483	6	0	210.8518414	0.001	-17.6858701	0.03127344	0.392499239	yes	LOC107996185	NW_016019064.1	+	1403019	1403446	428	one_exon
novel_circ_001484	20	6	702.8394715	218.9301613	-1.682724492	0.009399957	0.184206173	yes	LOC107996185	NW_016019064.1	+	1403019	1404614	1252	annot_exons
novel_circ_001502	0	22	0.001	802.7439247	19.61458032	4.82E-07	5.39E-05	yes	LOC107996165	NW_016019064.1	-	1633180	1635297	2118	antisense
novel_circ_001518	0	7	0.001	255.4185215	17.96250362	0.01564141	0.260767977	yes	LOC107996346	NW_016019075.1	-	1089457	1103341	13885	exon_intron
novel_circ_001520	17	3	597.4135507	109.4650806	-2.448259238	0.002588858	0.069680827	yes	LOC107996281	NW_016019075.1	-	1270838	1272015	576	annot_exons
novel_circ_001529	69	18	2424.796177	656.7904838	-1.884358353	1.31E-07	1.72E-05	yes	LOC107996327	NW_016019075.1	-	1598647	1599243	597	intronic
novel_circ_001536	12	3	421.7036829	109.4650806	-1.945758898	0.035222917	0.43235163	yes	LOC107996375	NW_016019086.1	+	275039	299670	1122	annot_exons
novel_circ_001561	184	87	6466.123137	3174.487339	-1.026377358	2.79E-08	4.15E-06	yes	LOC107996434	NW_016019086.1	+	1391740	1401625	6985	exon_intron
novel_circ_001562	0	6	0.001	218.9301613	17.7401112	0.03127344	0.392499239	yes	LOC114577344	NW_016019086.1	+	1508250	1525383	17134	exon_intron
novel_circ_001612	20	6	702.8394715	218.9301613	-1.682724492	0.009399957	0.184206173	yes	LOC107996901	NW_016019108.1	+	10863	11476	376	annot_exons
novel_circ_001648	6	0	210.8518414	0.001	-17.6858701	0.03127344	0.392499239	yes	LOC107996648	NW_016019108.1	-	5387163	5425273	15844	exon_intron
novel_circ_001684	0	8	0.001	291.9068817	18.1551487	0.00782341	0.159758136	yes	NA	NW_016019121.1	+	10003	11270	1268	intergenic

novel_circ_001721	9	1	316.277622	36.4836021	-3.115683899	0.021515144	0.31831015	yes	LOC107997401	NW_016019153.1	-	149599	158737	566	annot_exons
novel_circ_001752	17	4	597.4135507	145.9534409	-2.033221739	0.007226519	0.159758136	yes	LOC107997489	NW_016019164.1	-	1169104	1173807	1146	annot_exons
novel_circ_001777	26	2	913.6913129	72.9767243	-3.646198616	3.07E-06	0.00026419	yes	LOC107997643	NW_016019175.1	-	1161926	1162348	423	one_exon
novel_circ_001780	63	7	2213.944335	255.4185215	-3.115683899	4.42E-12	9.87E-10	yes	LOC107997643	NW_016019175.1	-	1256016	1256317	302	one_exon
novel_circ_001799	29	0	1019.117234	0.001	-19.95888859	3.80E-09	6.53E-07	yes	LOC107997783	NW_016019197.1	-	369005	389709	273	annot_exons
novel_circ_001800	6	0	210.851814	0.001	-17.6858701	0.03127344	0.392499239	yes	LOC107997877	NW_016019197.1	-	519760	520434	539	annot_exons
novel_circ_001827	47	15	1651.672758	547.3254032	-1.593457154	9.05E-05	0.005320766	yes	LOC107997823	NW_016019197.1	-	1336036	1356187	4644	exon_intron
novel_circ_001828	14	4	491.98763	145.9534409	-1.75311382	0.030957355	0.392499239	yes	LOC107997823	NW_016019197.1	-	1353341	1356187	2711	exon_intron
novel_circ_001834	16	3	562.2715772	109.4650806	-2.360796397	0.004442811	0.106723015	yes	LOC107997935	NW_016019205.1	+	10823	11868	887	annot_exons
novel_circ_001845	9	1	316.277622	36.4836021	-3.115683899	0.021515144	0.31831015	yes	LOC107998249	NW_016019219.1	-	26046	32448	634	annot_exons
novel_circ_001856	12	3	421.7036829	109.4650806	-1.945758898	0.035222917	0.43235163	yes	LOC107998096	NW_016019219.1	+	822790	825589	690	annot_exons
novel_circ_001863	13	4	456.8456565	145.9534409	-1.646198616	0.049136186	0.560052242	yes	LOC107998144	NW_016019219.1	-	1787103	1788306	920	annot_exons
novel_circ_001874	6	0	210.851814	0.001	-17.6858701	0.03127344	0.392499239	yes	LOC107998101	NW_016019219.1	-	2397387	2398613	1227	exon_intron
novel_circ_001925	0	7	0.001	255.4185215	17.96250362	0.01564141	0.260767977	yes	LOC107998284	NW_016019220.1	+	1263493	1263720	228	one_exon
novel_circ_001937	10	1	351.4197357	36.4836021	-3.267686993	0.011740243	0.21498117	yes	LOC107998284	NW_016019220.1	+	1263954	1264181	228	one_exon
novel_circ_001954	8	1	281.1357886	36.4836021	-2.945758898	0.039104693	0.459788861	yes	LOC107998401	NW_016019231.1	+	1178818	1179425	398	annot_exons
novel_circ_001959	45	15	1581.388811	547.3254032	-1.530721398	0.000208018	0.009887472	yes	LOC107998347	NW_016019231.1	-	1269123	1269311	189	antisense
novel_circ_001960	129	30	4533.314591	1094.650806	-2.050095558	6.76E-15	2.52E-12	yes	LOC107998347	NW_016019231.1	-	1269123	1269401	279	antisense
novel_circ_001961	49	17	1721.956705	620.3021236	-1.473005901	0.000155784	0.007838026	yes	LOC107998347	NW_016019231.1	-	1269141	1269329	189	antisense
novel_circ_001964	29	4	1019.117234	145.9534409	-2.803739893	1.11E-05	0.000854727	yes	LOC107998347	NW_016019231.1	-	1269159	1269347	189	antisense
novel_circ_001965	28	2	983.9752601	72.9767243	-3.75311382	8.82E-07	9.39E-05	yes	LOC107998347	NW_016019231.1	-	1269204	1269482	279	antisense
novel_circ_001967	172	37	6044.419455	1350.069328	-2.162570287	9.90E-21	4.42E-18	yes	LOC107998347	NW_016019231.1	-	1269240	1269518	279	antisense
novel_circ_001970	20	2	702.8394715	72.9767243	-3.267686993	0.000122056	0.006492192	yes	LOC107998347	NW_016019231.1	-	1269285	1269374	90	antisense
novel_circ_001971	13	0	456.8456565	0.001	-18.80134731	0.000245094	0.011174287	yes	LOC107998347	NW_016019231.1	-	1269285	1269482	198	antisense
novel_circ_001972	82	18	2881.641833	656.7904838	-2.133385901	2.97E-10	6.04E-08	yes	LOC107998347	NW_016019231.1	-	1269330	1269518	189	antisense
novel_circ_001973	8	0	281.1357886	0.001	-18.10090759	0.007823441	0.159758136	yes	LOC107998347	NW_016019231.1	+	1274306	1274422	117	one_exon
novel_circ_001975	21	2	737.98145	72.9767243	-3.33807632	6.66E-05	0.004022033	yes	LOC107998347	NW_016019231.1	-	1274315	1274431	117	antisense
novel_circ_001986	9	1	316.277622	36.4836021	-3.115683899	0.021515144	0.31831015	yes	LOC107998414	NW_016019242.1	+	143075	147648	735	annot_exons
novel_circ_002039	11	24	386.5617093	875.7206451	1.179771984	0.041134536	0.478617467	yes	LOC107998746	NW_016019275.1	+	557607	559754	672	annot_exons
novel_circ_002078	6	0	210.851814	0.001	-17.6858701	0.03127344	0.392499239	yes	LOC107998930	NW_016019297.1	+	27949	28331	383	one_exon
novel_circ_002084	31	9	1089.401181	328.3952419	-1.730030207	0.000687676	0.026038435	yes	LOC107998877	NW_016019297.1	-	309763	320580	9388	exon_intron

novel_circ_002087	17	6	597.4135507	218.9301613	-1.448259238	0.034791993	0.431807293	yes	LOC107998974	NW_016019297.1	-	431683	432366	418	annot_exons
novel_circ_002163	7	26	245.993815	948.6973655	1.947325898	0.001330398	0.043707475	yes	LOC107999286	NW_016019330.1	-	3143987	3145820	1028	annot_exons
novel_circ_002165	4	21	140.5678943	766.2555645	2.446558525	0.000916869	0.033578432	yes	LOC107999287	NW_016019330.1	+	3191831	3207371	15541	antisense
novel_circ_002175	13	2	456.8456565	72.97672043	-2.646198616	0.007406097	0.159758136	yes	LOC107999482	NW_016019331.1	-	471375	471722	253	annot_exons
novel_circ_002180	0	7	0.001	255.4185215	17.96250362	0.01564141	0.260767977	yes	LOC107999516	NW_016019331.1	-	593429	593841	319	annot_exons
novel_circ_002185	11	1	386.5617093	36.48836021	-3.405190516	0.006362167	0.148052917	yes	LOC107999505	NW_016019331.1	-	1004815	1010710	831	annot_exons
novel_circ_002199	55	14	1932.808547	510.837043	-1.919763689	1.15E-06	0.00011213	yes	LOC107999597	NW_016019342.1	+	42755	63444	6098	exon_intron
novel_circ_002206	26	7	913.6913129	255.4185215	-1.838843694	0.001330398	0.043707475	yes	LOC107999588	NW_016019342.1	+	625257	637143	8642	exon_intron
novel_circ_002215	6	0	210.8518414	0.001	-17.6858701	0.03127344	0.392499239	yes	LOC107999690	NW_016019353.1	+	202451	205422	1451	annot_exons
novel_circ_002267	0	7	0.001	255.4185215	17.96250362	0.01564141	0.260767977	yes	LOC107999825	NW_016019375.1	+	665494	665676	183	one_exon
novel_circ_002275	340	764	11948.27101	27877.1072	1.222278994	1.77E-39	1.98E-36	yes	LOC107999817	NW_016019375.1	-	687908	691683	3776	exon_intron
novel_circ_002286	18	4	632.5555243	145.9534409	-2.115683899	0.004363941	0.106723015	yes	LOC107999893	NW_016019386.1	-	393967	394850	388	annot_exons
novel_circ_002326	31	14	1089.401181	510.837043	-1.092600286	0.02277604	0.328268853	yes	LOC114577815	NW_016019397.1	-	267408	271584	4177	exon_intron
novel_circ_002354	6	0	210.8518414	0.001	-17.6858701	0.03127344	0.392499239	yes	LOC108000109	NW_016019419.1	+	338750	339330	448	annot_exons
novel_circ_002367	2	12	70.28394715	437.8603226	2.639203603	0.012969461	0.229950598	yes	LOC108000201	NW_016019430.1	+	514314	518155	1193	annot_exons
novel_circ_002377	28	55	983.9752601	2006.859812	1.028245894	0.001973027	0.057243404	yes	LOC108000176	NW_016019430.1	-	694364	695281	312	annot_exons
novel_circ_002378	0	6	0.001	218.9301613	17.7401112	0.03127344	0.392499239	yes	LOC108000173	NW_016019430.1	+	789557	790493	656	annot_exons
novel_circ_002388	14	1	491.98763	36.48836021	-3.75311382	0.000980413	0.034222525	yes	LOC108000170	NW_016019430.1	+	964004	964401	322	annot_exons
novel_circ_002405	0	6	0.001	218.9301613	17.7401112	0.03127344	0.392499239	yes	LOC108000369	NW_016019441.1	-	341401	341926	526	one_exon
novel_circ_002429	11	2	386.5617093	72.97672043	-2.405190516	0.022502846	0.326437384	yes	LOC108000332	NW_016019441.1	+	4133465	4139645	1212	annot_exons
novel_circ_002442	14	4	491.98763	145.9534409	-1.75311382	0.030957355	0.392499239	yes	LOC108000569	NW_016019442.1	+	361391	361827	275	annot_exons
novel_circ_002445	16	0	562.2715772	0.001	-19.10090759	3.07E-05	0.00214332	yes	LOC108000547	NW_016019442.1	+	481712	485016	316	annot_exons
novel_circ_002486	33	14	1159.685128	510.837043	-1.182798095	0.011444489	0.213058229	yes	LOC108000798	NW_016019475.1	+	167626	168515	814	annot_exons
novel_circ_002490	23	9	808.2653922	328.3952419	-1.299395852	0.02015933	0.311783718	yes	LOC108000823	NW_016019475.1	+	315730	316492	458	annot_exons
novel_circ_002496	25	10	878.5493393	364.8836021	-1.267686993	0.016767455	0.275430112	yes	LOC108000800	NW_016019475.1	+	413392	413722	331	one_exon
novel_circ_002502	43	20	1511.104864	729.7672043	-1.050095558	0.007237054	0.159758136	yes	LOC108000839	NW_016019475.1	-	471242	471758	258	annot_exons
novel_circ_002504	16	4	562.2715772	145.9534409	-1.945758898	0.011858624	0.215383472	yes	LOC108000839	NW_016019475.1	-	471618	481845	651	annot_exons
novel_circ_002505	23	9	808.2653922	328.3952419	-1.299395852	0.02015933	0.311783718	yes	LOC108000839	NW_016019475.1	-	471618	496478	14312	exon_intron
novel_circ_002506	16	2	562.2715772	72.97672043	-2.945758898	0.001318335	0.043707475	yes	LOC108000839	NW_016019475.1	-	475968	476299	332	one_exon
novel_circ_002521	45	19	1581.388811	693.278844	-1.189684481	0.0022628	0.064051082	yes	LOC108000805	NW_016019475.1	-	844669	846186	1159	exon_intron
novel_circ_002548	34	16	1194.827101	583.8137634	-1.033221739	0.021451342	0.31831015	yes	LOC108001011	NW_016019508.1	+	95288	95775	488	one_exon

novel_circ_002570	9	23	316.277762 2	839.2322849	1.407878057	0.02015933	0.311783718	yes	LOC108000995	NW_016019508.1	-	743042	743818	462	annot_exons
novel_circ_002596	13	50	456.856565	1824.418011	1.997657574	1.95E-06	0.000174176	yes	LOC108001152	NW_016019530.1	-	119027	119161	135	one_exon
novel_circ_002598	5	18	175.7098679	656.7904838	1.902238009	0.010665387	0.201492914	yes	LOC108001153	NW_016019530.1	+	119027	119368	342	intronic
novel_circ_002601	16	48	562.2715772	1751.44129	1.639203603	5.22E-05	0.003241729	yes	LOC108001152	NW_016019530.1	-	119035	119169	135	one_exon
novel_circ_002604	3	21	105.4259207	766.2555645	2.861596024	0.000279321	0.0123535	yes	LOC108001152	NW_016019530.1	-	119107	119310	204	one_exon
novel_circ_002605	4	22	140.5678943	802.7439247	2.513672721	0.000537698	0.022244768	yes	LOC108001153	NW_016019530.1	+	119107	119310	204	intronic
novel_circ_002606	9	26	316.2777622	948.6973655	1.584755819	0.006030945	0.141822425	yes	LOC108001152	NW_016019530.1	-	119107	119379	273	one_exon
novel_circ_002607	4	23	140.5678943	839.2322849	2.577803058	0.000313464	0.013466886	yes	LOC108001153	NW_016019530.1	+	119108	119311	204	intronic
novel_circ_002608	3	22	105.4259207	802.7439247	2.92871022	0.000157883	0.007838026	yes	LOC108001152	NW_016019530.1	-	119108	119311	204	one_exon
novel_circ_002609	9	28	316.2777622	1021.674086	1.691671023	0.002586079	0.069680827	yes	LOC108001152	NW_016019530.1	-	119108	119380	273	one_exon
novel_circ_002610	13	39	456.856565	1423.046048	1.639203603	0.000273429	0.012216814	yes	LOC108001153	NW_016019530.1	+	119108	119380	273	intronic
novel_circ_002619	8	0	281.1357886	0.001	-18.10090759	0.007823441	0.159758136	yes	LOC108001218	NW_016019530.1	+	511416	511869	454	one_exon
novel_circ_002625	59	122	2073.376441	4451.579946	1.10233539	1.25E-06	0.000116097	yes	LOC108001231	NW_016019541.1	+	160754	167218	6376	exon_intron
novel_circ_002629	27	11	948.8332865	401.3719623	-1.241214781	0.013941737	0.243326877	yes	LOC108001250	NW_016019541.1	+	209718	215649	5932	exon_intron
novel_circ_002673	4	14	140.5678943	510.870433	1.861596024	0.030957355	0.392499239	yes	LOC108001520	NW_016019553.1	-	538386	539273	730	annot_exons
novel_circ_002674	3	17	105.4259207	620.3021236	2.556741443	0.002588858	0.069680827	yes	LOC108001512	NW_016019553.1	+	577721	577991	271	one_exon
novel_circ_002690	6	0	210.8518414	0.001	-17.6858701	0.03127344	0.392499239	yes	LOC108001529	NW_016019564.1	-	894318	895720	551	annot_exons
novel_circ_002730	9	1	316.2777622	36.4883621	-3.115683899	0.021515144	0.31831015	yes	LOC108001667	NW_016019608.1	+	705648	708844	307	annot_exons
novel_circ_002756	27	9	948.8332865	328.3952419	-1.530721398	0.003964685	0.098412298	yes	LOC108001888	NW_016019641.1	+	294034	294523	490	one_exon
novel_circ_002766	1	8	35.14197357	291.9068817	3.054241102	0.039104693	0.459788861	yes	LOC108001957	NW_016019652.1	+	102451	105178	2728	exon_intron
novel_circ_002812	10	0	351.4197357	0.001	-18.42283569	0.001957522	0.057243404	yes	LOC108002038	NW_016019663.1	-	3282444	3283274	831	antisense
novel_circ_002833	19	5	667.6974979	182.418011	-1.871758316	0.006642077	0.152019679	yes	LOC108002298	NW_016019675.1	-	406536	408463	532	annot_exons
novel_circ_002852	21	74	737.981445	2700.138656	1.871377045	1.25E-08	2.00E-06	yes	LOC114578104	NW_016019697.1	-	183819	189121	2434	exon_intron
novel_circ_002869	6	0	210.8518414	0.001	-17.6858701	0.03127344	0.392499239	yes	LOC108002442	NW_016019708.1	-	29270	30875	1606	intronic
novel_circ_002882	42	17	1475.96289	620.3021236	-1.250613479	0.002265011	0.064051082	yes	LOC108002419	NW_016019708.1	+	577672	582121	1818	exon_intron
novel_circ_002884	14	3	491.98763	109.4650806	-2.168151319	0.012762169	0.228085479	yes	LOC108002492	NW_016019719.1	-	367187	367511	325	one_exon
novel_circ_002914	10	2	351.4197357	72.9767243	-2.267686993	0.038630625	0.459788861	yes	LOC108002638	NW_016019763.1	-	579018	579620	352	annot_exons
novel_circ_002951	15	5	527.1296036	182.418011	-1.530721398	0.041489297	0.480243983	yes	LOC108002801	NW_016019774.1	+	1586667	1588508	1530	annot_exons
novel_circ_002973	8	0	281.1357886	0.001	-18.10090759	0.007823441	0.159758136	yes	LOC108002696	NW_016019774.1	+	2372525	2373533	512	annot_exons
novel_circ_002977	51	18	1792.240652	656.7904838	-1.448259238	0.000134215	0.00697293	yes	LOC108002698	NW_016019774.1	+	2682716	2691498	8411	exon_intron
novel_circ_002986	10	1	351.4197357	36.4883621	-3.267686993	0.011740243	0.21498117	yes	LOC108002701	NW_016019774.1	-	2807386	2808036	651	one_exon

novel_circ_003002	8	1	281.1357886	36.48836021	-2.945758898	0.039104693	0.459788861	yes	LOC108002845	NW_016019774.1	-	3084677	3086029	1216	annot_exons
novel_circ_003085	12	75	421.7036829	2736.627016	2.698097292	1.73E-12	4.30E-10	yes	LOC108003328	NW_016019863.1	+	137310	145720	7759	exon_intron
novel_circ_003092	7	0	245.993815	0.001	-17.90826252	0.01564141	0.260767977	yes	LOC108003317	NW_016019863.1	+	221236	251682	1042	annot_exons

Table S4B. DEcircRNAs in the AcCK2 vs AcT2 comparison group

CircRNA ID	AcCK2_count	AcT2_count	AcCK2 RPM	AcT2 RPM	log2(Fold change)	*P* value	FDR	Significant	Source gene ID	Chromosome	Strand	Genomic start	Genomic end	Length	Type
novel_circ_000132	1	8	27.96889858	262.7085249	3.23156767	0.039104693	0.679936635	yes	ncbi_107995253	NW_016017456.1	+	3426392	3427473	674	annot_exons
novel_circ_000133	15	2	419.5334788	65.67713122	-2.675322926	0.00419502	0.254308937	yes	ncbi_107994792	NW_016017456.1	+	3482166	3485591	1915	annot_exons
novel_circ_000139	12	2	335.626783	65.67713122	-2.353394831	0.022502846	0.542503374	yes	ncbi_107996802	NW_016017457.1	+	514662	515276	319	annot_exons
novel_circ_000182	3	13	83.90669575	426.9013529	2.347044887	0.012762169	0.454243248	yes	ncbi_107999178	NW_016017479.1	-	358910	359324	415	one_exon
novel_circ_000188	6	0	167.8133915	0.001	-17.35649832	0.03127344	0.631948879	yes	ncbi_107999718	NW_016017501.1	-	25381	26776	690	annot_exons
novel_circ_000292	34	14	950.9425519	459.7399186	-1.048540249	0.018760753	0.500956768	yes	ncbi_108003484	NW_016017567.1	+	3246629	3247649	526	annot_exons
novel_circ_000306	5	15	139.8444929	492.5784842	1.816530171	0.011858624	0.443314912	yes	ncbi_108003717	NW_016017579.1	-	504274	554874	3384	annot_exons
novel_circ_000331	8	1	223.7511887	32.83856561	-2.76843233	0.039104693	0.679936635	yes	ncbi_108003857	NW_016017623.1	+	291902	293075	442	annot_exons
novel_circ_000450	21	6	587.3468703	197.0313937	-1.575787252	0.01923675	0.502491792	yes	ncbi_108004278	NW_016017745.1	-	212656	219180	6031	exon_intron
novel_circ_000504	43	17	1202.662639	558.2556154	-1.107234243	0.008738727	0.36298082	yes	ncbi_107992423	NW_016017812.1	+	396783	397380	598	one_exon
novel_circ_000518	1	13	27.96889858	426.9013529	3.932007388	0.000980413	0.07093759	yes	ncbi_107992482	NW_016017823.1	+	395758	396198	331	annot_exons
novel_circ_000524	16	3	447.5023774	98.51569683	-2.183469829	0.007563544	0.33109393	yes	ncbi_107992480	NW_016017823.1	+	418611	419785	620	annot_exons
novel_circ_000558	1	11	27.96889858	361.2242217	3.690999289	0.003427498	0.219653633	yes	ncbi_107992808	NW_016017900.1	-	2540	30945	577	annot_exons
novel_circ_000560	7	0	195.7822901	0.001	-17.57889074	0.01564141	0.460037265	yes	ncbi_107992839	NW_016017900.1	-	244921	245642	722	exon_intron
novel_circ_000610	8	1	223.7511887	32.83856561	-2.76843233	0.039104693	0.679936635	yes	ncbi_107992960	NW_016017956.1	+	109254	195008	28906	exon_intron
novel_circ_000627	9	21	251.7200873	689.6098778	1.453960091	0.016202828	0.460037265	yes	ncbi_107992960	NW_016017956.1	+	172365	172685	321	one_exon
novel_circ_000633	6	15	167.8133915	492.5784842	1.553495765	0.026681361	0.592537548	yes	ncbi_107992960	NW_016017956.1	+	172365	218602	1617	annot_exons
novel_circ_000651	0	13	0.001	426.9013529	18.70354321	0.000122627	0.013752576	yes	ncbi_107992960	NW_016017956.1	+	218340	220412	425	annot_exons
novel_circ_000850	8	1	223.7511887	32.83856561	-2.76843233	0.039104693	0.679936635	yes	ncbi_107993192	NW_016018011.1	-	664474	666478	2005	intronic
novel_circ_000882	23	2	643.2846674	65.67713122	-3.291994286	3.62E-05	0.005078582	yes	ncbi_107993312	NW_016018045.1	-	277040	279392	523	annot_exons
novel_circ_000895	15	3	419.5334788	98.51569683	-2.090360425	0.012762169	0.454243248	yes	ncbi_107993361	NW_016018069.1	-	3613	4278	504	annot_exons
novel_circ_000901	6	0	167.8133915	0.001	-17.35649832	0.03127344	0.631948879	yes	ncbi_107993429	NW_016018100.1	+	134748	199369	3566	exon_intron
novel_circ_001073	19	6	531.4090731	197.0313937	-1.431397343	0.043414226	0.732166237	yes	ncbi_107994200	NW_016018355.1	+	419	4027	3396	exon_intron
novel_circ_001080	9	0	251.7200873	0.001	-17.94146082	0.007823441	0.33109393	yes	ncbi_107994239	NW_016018389.1	-	182287	208876	616	annot_exons
novel_circ_001123	13	2	363.5956816	65.67713122	-2.468872048	0.012969461	0.454243248	yes	ncbi_107994397	NW_016018455.1	+	2426701	2427627	742	annot_exons
novel_circ_001	0	6	0.001	197.03139	17.58806599	0.0312734	0.6319488	yes	ncbi_107994	NW_01601848	-	578	2047	1177	annot_ex

134				37		4	79		550	7.1					ons
novel_circ_001166	0	7	0.001	229.8699593	17.81045841	0.01564141	0.460037265	yes	ncbi_107994917	NW_016018566.1	+	1834623	1835456	581	annot_exons
novel_circ_001175	34	11	950.9425519	361.2242217	-1.396463553	0.003694317	0.230176483	yes	ncbi_107994726	NW_016018566.1	+	1972335	1972743	409	one_exon
novel_circ_001266	7	0	195.7822901	0.001	-17.57889074	0.01564141	0.460037265	yes	ncbi_107995056	NW_016018567.1	+	285747	286040	294	one_exon
novel_circ_001391	59	17	1650.165017	558.2556154	-1.563612538	3.49E-05	0.005078582	yes	ncbi_107995620	NW_016018900.1	+	586736	588247	1281	annot_exons
novel_circ_001394	33	12	922.9736533	394.0627873	-1.227863949	0.009637793	0.379255595	yes	ncbi_107995623	NW_016018900.1	-	1175517	1176013	497	one_exon
novel_circ_001497	9	0	251.7200873	0.001	-17.94146082	0.007823441	0.33109393	yes	ncbi_107996164	NW_016019064.1	+	1616547	1619701	1365	annot_exons
novel_circ_001502	17	0	475.4712759	0.001	-18.85899866	3.07E-05	0.004918753	yes	ncbi_107996165	NW_016019064.1	-	1633180	1635297	2118	antisense
novel_circ_001531	11	23	307.6578844	755.2870091	1.295698007	0.016767455	0.463898479	yes	NA	NW_016019086.1	-	2851	3365	515	intergenic
novel_circ_001534	11	2	307.6578844	65.67713122	-2.227863949	0.038630625	0.679936635	yes	ncbi_107996375	NW_016019086.1	+	237649	242636	4988	one_exon
novel_circ_001553	6	0	167.8133915	0.001	-17.35649832	0.03127344	0.631948879	yes	ncbi_107996410	NW_016019086.1	-	949229	950018	790	one_exon
novel_circ_001570	7	23	195.7822901	755.2870091	1.947774704	0.000885647	0.068500213	yes	ncbi_107996539	NW_016019097.1	+	1117866	1119886	476	annot_exons
novel_circ_001573	2	10	55.93779717	328.3856561	2.553495765	0.022502846	0.542503374	yes	ncbi_107996539	NW_016019097.1	+	1121598	1123356	620	annot_exons
novel_circ_001745	17	4	475.4712759	131.3542624	-1.855895171	0.026681361	0.592537548	yes	ncbi_107997446	NW_016019153.1	+	1103648	1107426	2949	exon_intron
novel_circ_001755	9	1	251.7200873	32.83856561	-2.938357331	0.039104693	0.679936635	yes	ncbi_107997537	NW_016019164.1	+	1335252	1335984	472	annot_exons
novel_circ_001828	10	1	279.6889858	32.83856561	-3.090360425	0.021515144	0.542229988	yes	ncbi_107997823	NW_016019197.1	-	1353341	1356187	2711	exon_intron
novel_circ_001838	7	17	195.7822901	558.2556154	1.511675589	0.022735317	0.542503374	yes	ncbi_107997992	NW_016019208.1	-	778082	780422	1475	annot_exons
novel_circ_001904	8	0	223.7511887	0.001	-17.77153582	0.007823441	0.33109393	yes	NA	NW_016019219.1	+	4982690	4983321	632	intergenic
novel_circ_001920	18	5	503.4401745	164.1928281	-1.616429237	0.034791993	0.661342718	yes	ncbi_107998284	NW_016019220.1	+	1263145	1264284	1140	one_exon
novel_circ_001925	10	1	279.6889858	32.83856561	-3.090360425	0.021515144	0.542229988	yes	ncbi_107998284	NW_016019220.1	+	1263493	1263720	228	one_exon
novel_circ_001935	18	3	503.4401745	98.51569683	-2.353394831	0.002588858	0.170788482	yes	ncbi_107998284	NW_016019220.1	-	1263858	1264085	228	antisense
novel_circ_001983	8	0	223.7511887	0.001	-17.77153582	0.007823441	0.33109393	yes	ncbi_107998457	NW_016019242.1	-	68663	69794	388	annot_exons
novel_circ_001997	0	6	0.001	197.0313937	17.58806599	0.03127344	0.631948879	yes	ncbi_107998447	NW_016019242.1	-	897967	900606	1764	annot_exons
novel_circ_002038	34	13	950.9425519	426.9013529	-1.155455453	0.011444489	0.43508454	yes	ncbi_107998746	NW_016019275.1	+	557607	558185	398	annot_exons
novel_circ_002045	88	33	2461.263075	1083.672665	-1.183469829	4.76E-05	0.006281734	yes	ncbi_107998728	NW_016019275.1	-	947067	948758	1267	annot_exons
novel_circ_002058	7	0	195.7822901	0.001	-17.57889074	0.01564141	0.460037265	yes	ncbi_114577681	NW_016019286.1	-	410050	410234	185	one_exon
novel_circ_002062	0	7	0.001	229.8699593	17.81045841	0.01564141	0.460037265	yes	ncbi_107998795	NW_016019286.1	+	559016	559474	324	annot_exons
novel_circ_002067	0	6	0.001	197.0313937	17.58806599	0.03127344	0.631948879	yes	ncbi_107998779	NW_016019286.1	-	890534	892098	1402	annot_exons
novel_circ_002071	12	22	335.626783	722.4484435	1.106036788	0.041134536	0.709728957	yes	ncbi_107998781	NW_016019286.1	+	968566	969515	506	annot_exons
novel_circ_002163	15	50	419.5334788	1641.928281	1.968533264	7.53E-07	0.000168861	yes	ncbi_107999286	NW_016019330.1	-	3143987	3145820	1028	annot_exons
novel_circ_002166	21	5	587.3468703	164.1928281	-1.838821658	0.009399957	0.376501846	yes	ncbi_107999287	NW_016019330.1	+	3191831	3207895	16065	antisense

novel_circ_002184	6	14	167.8133915	459.7399186	1.453960091	0.041489297	0.710385438	yes	ncbi_107999505	NW_016019331.1	-	1004815	1007566	698	annot_exons
novel_circ_002188	8	1	223.7511887	32.83856561	-2.76843233	0.039104693	0.679936635	yes	ncbi_107999525	NW_016019331.1	-	1071851	1074161	1774	annot_exons
novel_circ_002191	20	5	559.3779717	164.1928281	-1.76843233	0.01469352	0.460037265	yes	ncbi_107999520	NW_016019331.1	-	1180813	1195250	14438	antisense
novel_circ_002300	70	18	1957.822901	591.094181	-1.727790346	1.08E-06	0.00022081	yes	ncbi_107999877	NW_016019386.1	+	1001097	1021229	8622	exon_intron
novel_circ_002305	7	0	195.7822901	0.001	-17.57889074	0.01564141	0.460037265	yes	ncbi_107999877	NW_016019386.1	+	1011537	1021229	879	annot_exons
novel_circ_002321	66	25	1845.947307	820.9641403	-1.16897026	0.00045085	0.038894466	yes	ncbi_107999979	NW_016019397.1	-	133008	149416	16060	exon_intron
novel_circ_002355	33	14	922.9736533	459.7399186	-1.005471527	0.026065347	0.590551249	yes	ncbi_108000109	NW_016019419.1	+	338750	345793	6911	exon_intron
novel_circ_002424	24	7	671.253566	229.8699593	-1.546039909	0.016202828	0.460037265	yes	ncbi_108000242	NW_016019441.1	+	3163843	3165472	1630	one_exon
novel_circ_002490	11	2	307.6578844	65.67713122	-2.227863949	0.038630625	0.679936635	yes	ncbi_108000823	NW_016019475.1	+	315730	316492	458	annot_exons
novel_circ_002548	18	5	503.4401745	164.1928281	-1.616429237	0.034791993	0.661342718	yes	ncbi_108001011	NW_016019508.1	+	95288	95775	488	one_exon
novel_circ_002558	10	23	279.6889858	755.2870091	1.433201531	0.009098519	0.371054153	yes	ncbi_108001033	NW_016019508.1	-	561396	563255	942	annot_exons
novel_circ_002561	0	6	0.001	197.0313937	17.58806599	0.03127344	0.631948879	yes	ncbi_108001033	NW_016019508.1	-	563139	565513	564	annot_exons
novel_circ_002567	8	0	223.7511887	0.001	-17.77153582	0.007823441	0.33109393	yes	ncbi_108001012	NW_016019508.1	+	704306	727262	589	annot_exons
novel_circ_002568	12	2	335.626783	65.67713122	-2.353394831	0.022502846	0.542503374	yes	ncbi_108000995	NW_016019508.1	-	738155	740766	2019	annot_exons
novel_circ_002593	7	0	195.7822901	0.001	-17.57889074	0.01564141	0.460037265	yes	ncbi_108001079	NW_016019519.1	-	865247	865594	262	annot_exons
novel_circ_002598	17	167	475.4712759	5484.040457	3.527809121	1.18E-37	1.33E-34	yes	ncbi_108001153	NW_016019530.1	+	119027	119368	342	intronic
novel_circ_002604	7	39	195.7822901	1280.704059	2.709614967	1.83E-07	4.55E-05	yes	ncbi_108001152	NW_016019530.1	-	119107	119310	204	one_exon
novel_circ_002606	10	46	279.6889858	1510.574018	2.433201531	9.32E-08	2.99E-05	yes	ncbi_108001152	NW_016019530.1	-	119107	119379	273	one_exon
novel_circ_002608	7	41	195.7822901	1346.38119	2.781764753	3.36E-08	1.51E-05	yes	ncbi_108001152	NW_016019530.1	-	119108	119311	204	one_exon
novel_circ_002609	10	46	279.6889858	1510.574018	2.433201531	9.32E-08	2.99E-05	yes	ncbi_108001152	NW_016019530.1	-	119108	119380	273	one_exon
novel_circ_002610	10	33	279.6889858	1083.672665	1.954033694	0.000107819	0.012728272	yes	ncbi_108001153	NW_016019530.1	+	119108	119380	273	intronic
novel_circ_002611	7	0	195.7822901	0.001	-17.57889074	0.01564141	0.460037265	yes	ncbi_108001153	NW_016019530.1	+	119120	119182	63	intronic
novel_circ_002616	12	1	335.626783	32.83856561	-3.353394831	0.006362167	0.33109393	yes	ncbi_108001200	NW_016019530.1	+	368295	390707	9602	exon_intron
novel_circ_002658	12	1	335.626783	32.83856561	-3.353394831	0.006362167	0.33109393	yes	ncbi_108001352	NW_016019552.1	+	3608386	3610492	807	annot_exons
novel_circ_002702	8	0	223.7511887	0.001	-17.77153582	0.007823441	0.33109393	yes	ncbi_108001569	NW_016019586.1	-	390980	394274	1215	annot_exons
novel_circ_002752	9	0	251.7200873	0.001	-17.94146082	0.007823441	0.33109393	yes	ncbi_108001884	NW_016019641.1	-	116628	121806	335	annot_exons
novel_circ_002769	5	14	139.8444929	459.7399186	1.716994497	0.019266293	0.502491792	yes	ncbi_108001956	NW_016019652.1	-	203673	205565	396	annot_exons
novel_circ_002771	7	0	195.7822901	0.001	-17.57889074	0.01564141	0.460037265	yes	ncbi_108001958	NW_016019652.1	+	543809	545043	867	annot_exons
novel_circ_002842	47	10	1314.538233	328.3856561	-2.001093087	8.94E-06	0.001670231	yes	ncbi_108002344	NW_016019686.1	+	508494	508847	354	antisense
novel_circ_002879	18	4	503.4401745	131.3542624	-1.938357331	0.016959285	0.463898479	yes	ncbi_108002419	NW_016019708.1	+	573699	577893	4195	intronic
novel_circ_002894	6	0	167.8133915	0.001	-17.35649832	0.03127344	0.631948879	yes	ncbi_108002511	NW_016019727.1	-	3797	4608	384	annot_exons

novel_circ_002938	11	2	307.6578844	65.67713122	-2.227863949	0.038630625	0.679936635	yes	ncbi_108002836	NW_016019774.1	+	1211048	1211465	347	annot_exons
novel_circ_002946	17	33	475.4712759	1083.672665	1.188498948	0.006667095	0.33109393	yes	ncbi_108002801	NW_016019774.1	+	1581069	1581760	443	annot_exons
novel_circ_003046	19	2	531.4090731	65.67713122	-3.016359843	0.000404902	0.037841449	yes	ncbi_108003146	NW_016019808.1	+	241255	243126	980	annot_exons
novel_circ_003047	8	0	223.7511887	0.001	-17.77153582	0.007823441	0.33109393	yes	ncbi_108003146	NW_016019808.1	+	241255	246086	1247	annot_exons
novel_circ_003063	4	15	111.8755943	492.5784842	2.138458266	0.004442811	0.262242781	yes	ncbi_108003197	NW_016019841.1	+	537450	544381	291	annot_exons
novel_circ_003072	0	7	0.001	229.8699593	17.81045841	0.01564141	0.460037265	yes	ncbi_108003247	NW_016019852.1	-	95223	97492	1290	annot_exons
novel_circ_003085	46	18	1286.569335	591.094181	-1.122069285	0.005221826	0.300321941	yes	ncbi_108003328	NW_016019863.1	+	137310	145720	7759	exon_intron
novel_circ_003094	2	10	55.93779717	328.3856561	2.553495765	0.022502846	0.542503374	yes	ncbi_108003334	NW_016019863.1	+	306657	308299	479	annot_exons

Table S4C. DEcircRNAs in the AcCK3 vs AcT3 comparison group

CircRNA ID	AcCK3_count	AcT3_count	AcCK3 RPM	AcT3 RPM	log2(Fold change)	*P* value	FDR	Significant	Source gene ID	Chromosome	Strand	Genomic start	Genomic end	Length	Type
novel_circ_000012	1	7	36.37024914	363.6552548	3.32174071	0.039104693	0.747568992	yes	ncbi_108002084	NW_016017455.1	-	884856	886844	673	annot_exons
novel_circ_000043	10	1	363.7024914	51.95075069	-2.807542307	0.021515144	0.56364366	yes	ncbi_108003778	NW_016017455.1	-	2372297	2373068	292	annot_exons
novel_circ_000134	10	25	363.7024914	1298.768767	1.836313883	0.000761073	0.064599878	yes	ncbi_107994792	NW_016017456.1	+	3484999	3485591	304	annot_exons
novel_circ_000177	9	1	327.3322422	51.95075069	-2.655539214	0.039104693	0.747568992	yes	ncbi_114577653	NW_016017479.1	+	195048	195293	246	exon_intron
novel_circ_000187	8	0	290.9619931	0.001	-18.15047119	0.01564141	0.526842397	yes	ncbi_114577727	NW_016017490.1	+	387469	394777	6680	exon_intron
novel_circ_000285	13	1	472.8132388	51.95075069	-3.18605393	0.022502846	0.582329737	yes	ncbi_108003484	NW_016017567.1	+	3240424	3242024	1183	annot_exons
novel_circ_000292	55	19	2000.363702	987.0642631	-1.019046412	0.008670984	0.438091157	yes	ncbi_108003484	NW_016017567.1	+	3246629	3247649	526	annot_exons
novel_circ_000332	7	16	254.591744	831.212011	1.707030866	0.010665387	0.471499003	yes	ncbi_108003869	NW_016017634.1	+	46409	47274	393	annot_exons
novel_circ_000446	17	3	618.2942353	155.8522521	-1.988114553	0.019266293	0.56364366	yes	ncbi_108004244	NW_016017745.1	+	13027	41397	915	annot_exons
novel_circ_000495	8	0	290.9619931	0.001	-18.15047119	0.01564141	0.526842397	yes	ncbi_108004516	NW_016017790.1	-	273932	284130	533	annot_exons
novel_circ_000501	0	6	0.001	311.7045041	18.24981948	0.01564141	0.526842397	yes	ncbi_107992422	NW_016017812.1	-	185446	199748	14303	exon_intron
novel_circ_000524	10	0	363.7024914	0.001	-18.47239928	0.003913285	0.251636058	yes	ncbi_107992480	NW_016017823.1	+	418611	419785	620	annot_exons
novel_circ_000548	12	1	436.4429896	51.95075069	-3.070576713	0.038630625	0.747568992	yes	ncbi_107992595	NW_016017867.1	-	178833	186982	2387	annot_exons
novel_circ_000558	2	7	72.74049827	363.6552548	2.32174071	0.039104693	0.747568992	yes	ncbi_107992808	NW_016017900.1	-	2540	30945	577	annot_exons
novel_circ_000575	8	17	290.9619931	883.1627617	1.601848629	0.009399957	0.448209376	yes	ncbi_107992818	NW_016017900.1	-	1369198	1372070	348	annot_exons
novel_circ_000609	8	0	290.9619931	0.001	-18.15047119	0.01564141	0.526842397	yes	ncbi_107992960	NW_016017956.1	+	109254	174346	28756	exon_intron
novel_circ_000876	0	8	0.001	415.6060055	18.66485698	0.003913285	0.251636058	yes	ncbi_107993301	NW_016018045.1	-	91185	95869	433	annot_exons
novel_circ_000884	0	5	0.001	259.7537534	17.98678507	0.03127344	0.67032565	yes	ncbi_107993305	NW_016018045.1	-	326970	332279	803	annot_exons
novel_circ_000930	10	0	363.7024914	0.001	-18.47239928	0.003913285	0.251636058	yes	ncbi_107993580	NW_016018150.1	+	800	3864	2491	exon_intron
novel_circ_000957	16	24	581.9239862	1246.818017	1.099348289	0.027690349	0.67032565	yes	ncbi_107993704	NW_016018222.1	-	125030	125691	419	annot_exons
novel_circ_001	9	1	327.33224	51.950750	-2.65553	0.0391046	0.7475689	yes	ncbi_107994	NW_01601831	+	90933	91923	546	annot_ex

032			22	69	9214	93	92		002	1.1					ons
novel_circ_001036	15	2	545.553737	103.9015014	-2.392504808	0.021320761	0.56364366	yes	ncbi_107994046	NW_016018322.1	+	15072	20384	4868	exon_intron
novel_circ_001053	0	5	0.001	259.7537534	17.98678507	0.03127344	0.67032565	yes	ncbi_107994179	NW_016018344.1	+	951879	952211	333	one_exon
novel_circ_001074	2	9	72.74049827	467.5567562	2.684310789	0.011740243	0.5084244	yes	ncbi_107994204	NW_016018356.1	+	169024	169476	453	one_exon
novel_circ_001076	16	2	581.9239862	103.9015014	-2.485614212	0.012762169	0.520794653	yes	ncbi_107994228	NW_016018367.1	-	52592	58950	6359	antisense
novel_circ_001095	10	0	363.7024914	0.001	-18.47239928	0.003913285	0.251636058	yes	ncbi_107994275	NW_016018422.1	-	117942	135806	390	annot_exons
novel_circ_001164	0	6	0.001	311.7045041	18.24981948	0.01564141	0.526842397	yes	ncbi_107994844	NW_016018566.1	+	1750042	1750573	532	exon_intron
novel_circ_001248	7	0	254.591744	0.001	-17.95782611	0.03127344	0.67032565	yes	NA	NW_016018567.1	-	45392	45730	339	intergenic
novel_circ_001257	0	6	0.001	311.7045041	18.24981948	0.01564141	0.526842397	yes	ncbi_107995056	NW_016018567.1	-	283938	284613	676	antisense
novel_circ_001260	14	0	509.1834879	0.001	-18.95782611	0.000489894	0.043314822	yes	ncbi_107995056	NW_016018567.1	+	285266	285560	295	one_exon
novel_circ_001299	11	0	400.0727405	0.001	-18.60990281	0.001957522	0.148352232	yes	ncbi_107995149	NW_016018567.1	+	1887515	1888433	677	annot_exons
novel_circ_001366	2	8	72.74049827	415.6060055	2.514385788	0.021515144	0.56364366	yes	ncbi_107995510	NW_016018789.1	-	1688801	1690941	489	annot_exons
novel_circ_001388	58	16	2109.47445	831.212011	-1.343595207	0.000450305	0.041545537	yes	ncbi_107995644	NW_016018900.1	-	360625	361743	704	annot_exons
novel_circ_001391	29	44	1054.737225	2285.83303	1.115836411	0.001546482	0.121542045	yes	ncbi_107995620	NW_016018900.1	+	586736	588247	1281	annot_exons
novel_circ_001446	0	6	0.001	311.7045041	18.24981948	0.01564141	0.526842397	yes	ncbi_107996050	NW_016019052.1	-	33483	34880	865	annot_exons
novel_circ_001469	1	8	36.37024914	415.6060055	3.514385788	0.021515144	0.56364366	yes	ncbi_107996159	NW_016019063.1	-	28463	29183	529	annot_exons
novel_circ_001499	4	10	145.4809965	519.5075069	1.836313883	0.035222917	0.732774804	yes	ncbi_107996164	NW_016019064.1	+	1619355	1620204	437	annot_exons
novel_circ_001502	12	21	436.4429896	1090.965764	1.32174071	0.024425681	0.61703922	yes	ncbi_107996165	NW_016019064.1	-	1633180	1635297	2118	antisense
novel_circ_001519	290	441	10547.37225	22910.28105	1.119111543	3.11E-24	6.60E-21	yes	ncbi_107996346	NW_016019075.1	-	1095400	1103341	7942	exon_intron
novel_circ_001536	9	0	327.3322422	0.001	-18.32039619	0.007823441	0.436097646	yes	ncbi_107996375	NW_016019086.1	+	275039	299670	1122	annot_exons
novel_circ_001546	0	5	0.001	259.7537534	17.98678507	0.03127344	0.67032565	yes	ncbi_107996440	NW_016019086.1	+	526730	527679	435	annot_exons
novel_circ_001641	0	5	0.001	259.7537534	17.98678507	0.03127344	0.67032565	yes	ncbi_107996907	NW_016019108.1	-	4969148	4971791	1224	annot_exons
novel_circ_001651	24	35	872.8859793	1818.276274	1.058706304	0.006294205	0.385729069	yes	ncbi_107996648	NW_016019108.1	-	5409413	5425273	15424	exon_intron
novel_circ_001681	1	13	36.37024914	675.3597589	4.214825506	6.14E-05	0.008679857	yes	ncbi_107997067	NW_016019120.1	-	1483545	1484595	408	annot_exons
novel_circ_001714	2	7	72.74049827	363.6552548	2.32174071	0.039104693	0.747568992	yes	ncbi_107997257	NW_016019142.1	+	1069036	1070052	485	annot_exons
novel_circ_001780	8	0	290.9619931	0.001	-18.15047119	0.01564141	0.526842397	yes	ncbi_107997643	NW_016019175.1	-	1256016	1256317	302	one_exon
novel_circ_001807	9	0	327.3322422	0.001	-18.32039619	0.007823441	0.436097646	yes	ncbi_107997812	NW_016019197.1	+	889136	889908	392	annot_exons
novel_circ_001840	1	7	36.37024914	363.6552548	3.32174071	0.039104693	0.747568992	yes	ncbi_107997953	NW_016019208.1	-	1133155	1135195	1145	exon_intron
novel_circ_001844	4	11	145.4809965	571.4582576	1.973817407	0.021320761	0.56364366	yes	ncbi_107998249	NW_016019219.1	-	627	1946	1313	annot_exons
novel_circ_001930	10	17	363.7024914	883.1627617	1.279920534	0.035825563	0.738076164	yes	ncbi_107998284	NW_016019220.1	+	1263638	1264093	456	one_exon
novel_circ_001936	56	11	2036.733952	571.4582576	-1.833537515	3.86E-05	0.005855773	yes	ncbi_107998284	NW_016019220.1	+	1263949	1264176	228	one_exon

novel_circ_001986	4	10	145.4809965	519.5075069	1.836313883	0.035222917	0.732774804	yes	ncbi_107998414	NW_016019242.1	+	143075	147648	735	annot_exons
novel_circ_002006	9	0	327.322422	0.001	-18.32039619	0.007823441	0.436097646	yes	ncbi_107998560	NW_016019253.1	+	470253	474998	338	annot_exons
novel_circ_002009	101	19	3673.395163	987.0642631	-1.895898181	3.55E-09	1.25E-06	yes	ncbi_107998561	NW_016019253.1	-	486082	487534	956	annot_exons
novel_circ_002103	2	10	72.74049827	519.5075069	2.836313883	0.006362167	0.385729069	yes	ncbi_107999044	NW_016019308.1	+	901909	902475	567	antisense
novel_circ_002124	7	0	254.591744	0.001	-17.95782611	0.03127344	0.67032565	yes	ncbi_107999220	NW_016019330.1	+	1533671	1537860	691	annot_exons
novel_circ_002186	7	0	254.591744	0.001	-17.95782611	0.03127344	0.67032565	yes	ncbi_107999505	NW_016019331.1	-	1004815	1023496	2514	exon_intron
novel_circ_002267	26	7	945.6264775	363.6552548	-1.378699008	0.029573372	0.67032565	yes	ncbi_107999825	NW_016019375.1	+	665494	665676	183	one_exon
novel_circ_002313	17	3	618.2942353	155.8522521	-1.988114553	0.019266293	0.56364366	yes	ncbi_107999918	NW_016019388.1	+	4831	5831	716	annot_exons
novel_circ_002326	10	1	363.7024914	51.95075069	-2.807542307	0.021515144	0.56364366	yes	ncbi_14577815	NW_016019397.1	-	267408	271584	4177	exon_intron
novel_circ_002349	8	0	290.9619931	0.001	-18.15047119	0.01564141	0.526842397	yes	ncbi_108000089	NW_016019408.1	+	742917	744357	984	annot_exons
novel_circ_002378	17	3	618.2942353	155.8522521	-1.988114553	0.019266293	0.56364366	yes	ncbi_108000173	NW_016019430.1	+	789557	790493	656	annot_exons
novel_circ_002386	1	16	36.37024914	831.212011	4.514385788	7.69E-06	0.001359483	yes	ncbi_108000170	NW_016019430.1	+	960522	962084	1091	annot_exons
novel_circ_002399	1	17	36.37024914	883.1627617	4.601848629	3.85E-06	0.000816423	yes	ncbi_108000225	NW_016019441.1	+	179214	193259	8768	exon_intron
novel_circ_002429	0	5	0.001	259.7537534	17.98678507	0.03127344	0.67032565	yes	ncbi_108000332	NW_016019441.1	+	4133465	4139645	1212	annot_exons
novel_circ_002502	18	28	654.6644845	1454.621019	1.151815709	0.00801499	0.436097646	yes	ncbi_108000839	NW_016019475.1	-	471242	471758	258	annot_exons
novel_circ_002548	4	11	145.4809965	571.4582576	1.973817407	0.021320761	0.56364366	yes	ncbi_108001011	NW_016019508.1	+	95288	95775	488	one_exon
novel_circ_002606	2	15	72.74049827	779.2612603	3.421276384	0.000145836	0.016529392	yes	ncbi_108001152	NW_016019530.1	-	119107	119379	273	one_exon
novel_circ_002608	1	8	36.37024914	415.6060055	3.514385788	0.021515144	0.56364366	yes	ncbi_108001152	NW_016019530.1	-	119108	119311	204	one_exon
novel_circ_002609	2	16	72.74049827	831.212011	3.514385788	7.68E-05	0.009588582	yes	ncbi_108001152	NW_016019530.1	-	119108	119380	273	one_exon
novel_circ_002643	6	16	218.2214948	831.212011	1.929423287	0.010665387	0.471499003	yes	NA	NW_016019552.1	-	378094	378260	167	intergenic
novel_circ_002651	8	15	290.9619931	779.2612603	1.421276384	0.034791993	0.732774804	yes	ncbi_108001331	NW_016019552.1	+	2337070	2347657	9063	exon_intron
novel_circ_002695	17	3	618.2942353	155.8522521	-1.988114553	0.019266293	0.56364366	yes	ncbi_108001561	NW_016019586.1	+	178530	179629	560	annot_exons
novel_circ_002709	410	116	14911.80215	6026.28708	-1.307113316	1.26E-19	8.94E-17	yes	ncbi_108001641	NW_016019597.1	-	100949	103155	1368	annot_exons
novel_circ_002734	7	0	254.591744	0.001	-17.95782611	0.03127344	0.67032565	yes	ncbi_108001834	NW_016019630.1	-	109062	110074	787	annot_exons
novel_circ_002737	11	24	400.0727405	1246.818017	1.63991667	0.002586079	0.189229639	yes	ncbi_108001839	NW_016019630.1	-	485901	487472	1572	intronic
novel_circ_002752	1	8	36.37024914	415.6060055	3.514385788	0.021515144	0.56364366	yes	ncbi_108001884	NW_016019641.1	-	116628	121806	335	annot_exons
novel_circ_002756	4	11	145.4809965	571.4582576	1.973817407	0.021320761	0.56364366	yes	ncbi_108001888	NW_016019641.1	+	294034	294523	490	one_exon
novel_circ_002840	9	1	327.322422	51.95075069	-2.655539214	0.039104693	0.747568992	yes	ncbi_108002344	NW_016019686.1	-	206281	207323	612	annot_exons
novel_circ_002848	21	32	763.752319	1662.424022	1.122068365	0.009165577	0.448209376	yes	ncbi_108002348	NW_016019686.1	-	720170	720928	759	one_exon
novel_circ_002983	8	0	290.9619931	0.001	-18.15047119	0.01564141	0.526842397	yes	ncbi_108002839	NW_016019774.1	+	2779251	2779653	270	annot_exons
novel_circ_002996	16	3	581.9239862	155.8522521	-1.900651711	0.030957355	0.67032565	yes	ncbi_108002703	NW_016019774.1	-	2992572	2993799	766	annot_exons

novel_circ_003058	21	30	763.7752319	1558.522521	1.028958961	0.017678447	0.56364366	yes	ncbi_108003167	NW_016019819.1	-	442469	457362	12855	exon_intron
novel_circ_003087	4	25	145.4809965	1298.768767	3.158241978	4.72E-06	0.000910515	yes	ncbi_108003328	NW_016019863.1	+	137310	147878	9917	exon_intron
novel_circ_003088	6	31	218.2214948	1610.473271	2.883619598	3.43E-07	0.000104053	yes	ncbi_108003328	NW_016019863.1	+	137310	155063	1669	exon_intron
novel_circ_003151	1	8	36.37024914	415.6060055	3.514385788	0.021515144	0.56364366	yes	ncbi_108003308	NW_016019863.1	-	461832	462455	624	antisense

Table S5. Antioxidant enzyme-associated DEcircRNAs shared by the 4-, 5-, and 6-day-old comparison groups

Antioxidant enzyme	mRNA	DEmiRNA	DEcircRNA
Superoxide dismutase	XM_017061630.1	miR-1277-x	novel_circ_001562
Superoxide dismutase	XM_017061630.1	miR-1277-x	novel_circ_002165
Superoxide dismutase	XM_017061630.1	miR-1277-x	novel_circ_003087
Catalase	XM_017050424.1	miR-1344-x	novel_circ_000609
Catalase	XM_017050424.1	novel-m0032-5p	novel_circ_000138
Catalase	XM_017050424.1	novel-m0032-5p	novel_circ_000589
Catalase	XM_017050424.1	novel-m0032-5p	novel_circ_000942
Catalase	XM_017050424.1	novel-m0032-5p	novel_circ_001307
Catalase	XM_017050424.1	novel-m0032-5p	novel_circ_001397
Catalase	XM_017050424.1	novel-m0032-5p	novel_circ_002117
Catalase	XM_017050424.1	novel-m0032-5p	novel_circ_002206
Catalase	XM_017050424.1	novel-m0032-5p	novel_circ_002624
Catalase	XM_017050424.1	novel-m0032-5p	novel_circ_002778
Glutathione S-transferase	XM_017060408.1	novel-m0044-5p	novel_circ_000609
Glutathione S-transferase	XM_017060408.1	novel-m0044-5p	novel_circ_002321
Glutathione S-transferase	XM_017060408.1	novel-m0044-5p	novel_circ_002616
Glutathione S-transferase	XM_017060408.1	novel-m0003-3p	novel_circ_000129
Glutathione S-transferase	XM_017060408.1	novel-m0003-3p	novel_circ_002165
Glutathione S-transferase	XM_017060408.1	novel-m0003-3p	novel_circ_003087

Table S6. Targeting relationships between DEcircRNAs and DEmiRNAs and between DEmiRNAs and Immune defense-associated DEmRNAs

DEmRNA-Pathway		miRNA-mRNA		miRNA-circRNA	
DEmRNA	Pathway	DEmiRNA	DEmRNA	DEmiRNA	DEcircRNA
XM_028665820.1	Melanogenesis	miR-6001-x	XM_028665820.1	miR-6001-x	novel_circ_001148
XM_017066215.2	Melanogenesis	miR-6001-y	XM_017066215.2	miR-6001-x	novel_circ_001797
XM_028668804.1	Melanogenesis	miR-6001-y	XM_028668804.1	miR-6001-x	novel_circ_002617
XM_017063183.2	Melanogenesis	miR-6001-y	XM_017063183.2	miR-6001-x	novel_circ_002632
XM_017060255.2	Melanogenesis	miR-6001-y	XM_017060255.2	miR-6001-x	novel_circ_002635
XM_017052856.2	Melanogenesis	miR-6001-y	XM_028665820.1	miR-6001-x	novel_circ_003058
XM_017052685.2	Melanogenesis	miR-6001-y	XM_017052856.2	miR-6001-x	novel_circ_003059
XM_017049275.2	Melanogenesis	novel-m0032-5p	XM_028665820.1	miR-6001-y	novel_circ_000090
XM_017060185.2	Endocytosis	novel-m0032-5p	XM_017052685.2	miR-6001-y	novel_circ_000091
XM_017054665.2	Endocytosis	novel-m0032-5p	XM_017049275.2	miR-6001-y	novel_circ_000137

XM_017051613.2	Endocytosis	miR-2779-x	XM_017060185.2	miR-6001-y	novel_circ_000382
XM_017066472.2	Endocytosis	miR-5106-y	XM_017054665.2	miR-6001-y	novel_circ_000501
XM_017054127.2	Endocytosis	miR-6001-x	XM_017054665.2	miR-6001-y	novel_circ_000589
XM_017066770.2	Endocytosis	miR-6001-x	XM_017051613.2	miR-6001-y	novel_circ_001037
XM_017066209.2	Endocytosis	miR-6001-y	XM_017066472.2	miR-6001-y	novel_circ_001069
XM_017058627.2	Endocytosis	miR-6001-y	XM_017054127.2	miR-6001-y	novel_circ_001148
XM_028666742.1	Autophagy - animal	novel-m0032-5p	XM_017066770.2	miR-6001-y	novel_circ_001169
XM_017059234.2	Autophagy - animal	novel-m0032-5p	XM_017066472.2	miR-6001-y	novel_circ_001170
XM_017053048.2	Autophagy - animal	novel-m0032-5p	XM_017066209.2	miR-6001-y	novel_circ_001276
XM_028665190.1	Autophagy - animal	novel-m0032-5p	XM_017058627.2	miR-6001-y	novel_circ_001281
XM_028666329.1	Apoptosis - fly	novel-m0032-5p	XM_017051613.2	miR-6001-y	novel_circ_001498
XM_017055568.2	Apoptosis - fly	miR-2779-x	XM_028666742.1	miR-6001-y	novel_circ_001561
XM_017058590.2	Apoptosis - fly	miR-6001-x	XM_017059234.2	miR-6001-y	novel_circ_001627
XM_017063771.2	Apoptosis - fly	novel-m0006-5p	XM_017053048.2	miR-6001-y	novel_circ_001653
XM_017059464.2	Apoptosis - fly	novel-m0032-5p	XM_017059234.2	miR-6001-y	novel_circ_001828
XM_017055524.2	Apoptosis - fly	novel-m0043-5p	XM_028665190.1	miR-6001-y	novel_circ_002117
XM_017054502.2	Apoptosis - fly	miR-2770-y	XM_028666329.1	miR-6001-y	novel_circ_002199
XM_017049987.1	Apoptosis - fly	miR-2779-x	XM_017055568.2	miR-6001-y	novel_circ_002206
XM_017056893.2	Apoptosis - fly	miR-6001-x	XM_017058590.2	miR-6001-y	novel_circ_002233
XM_017058590.2	Apoptosis	miR-6001-x	XM_028666329.1	miR-6001-y	novel_circ_002257
XM_017063771.2	Apoptosis	miR-6001-y	XM_017063771.2	miR-6001-y	novel_circ_002321
XM_017061972.2	Apoptosis	miR-6001-y	XM_017059464.2	miR-6001-y	novel_circ_002424
XM_017056893.2	Apoptosis	miR-6001-y	XM_017058590.2	miR-6001-y	novel_circ_002624
XM_028665190.1	Apoptosis	miR-6001-y	XM_017055568.2	miR-6001-y	novel_circ_002635
XM_028666849.1	Insect hormone biosynthesis	miR-6001-y	XM_017055524.2	miR-6001-y	novel_circ_002651
XM_017062490.2	MAPK signaling pathway - fly	miR-6001-y	XM_028666329.1	miR-6001-y	novel_circ_002694
XM_017055568.2	MAPK signaling pathway - fly	miR-6001-y	XM_017054502.2	miR-6001-y	novel_circ_002747
XM_028669298.1	MAPK signaling pathway - fly	miR-965-y	XM_017055524.2	miR-6001-y	novel_circ_002766
XM_017065018.2	MAPK signaling pathway - fly	novel-m0006-5p	XM_017049987.1	miR-6001-y	novel_circ_002881
XM_017055524.2	MAPK signaling pathway - fly	novel-m0032-5p	XM_017063771.2	miR-6001-y	novel_circ_003059
XM_017051652.2	MAPK signaling pathway - fly	novel-m0032-5p	XM_017056893.2	novel-m0032-5p	novel_circ_000138
XM_017048593.2	MAPK signaling pathway - fly	novel-m0032-5p	XM_017055568.2	novel-m0032-5p	novel_circ_000589
XM_028669067.1	MAPK signaling pathway - fly	novel-m0032-5p	XM_017061972.2	novel-m0032-5p	novel_circ_000942
XM_028668004.1	MAPK signaling pathway - fly	novel-m0032-5p	XM_028666849.1	novel-m0032-5p	novel_circ_001307
XM_017055524.2	Toll and Imd signaling pathway	miR-2765-x	XM_017062490.2	novel-m0032-5p	novel_circ_001397
XM_017048593.2	Toll and Imd signaling pathway	miR-6001-y	XM_028669298.1	novel-m0032-5p	novel_circ_002117
XM_017055524.2	Toll-like receptor signaling pathway	miR-6001-y	XM_017065018.2	novel-m0032-5p	novel_circ_002206
XM_017051652.2	Toll-like receptor signaling pathway	miR-980-y	XM_017051652.2	novel-m0032-5p	novel_circ_002624
XM_017048593.2	Toll-like receptor signaling pathway	novel-m0006-5p	XM_017048593.2	novel-m0032-5p	novel_circ_002778
XM_017051964.2	NF-kappa B signaling pathway	novel-m0032-5p	XM_028669067.1	miR-2779-x	novel_circ_000501

XM_017063980.2	Jak-STAT signaling pathway	novel-m0032-5p	XM_017062490.2	miR-2779-x	novel_circ_001653
XM_017052795.2	Jak-STAT signaling pathway	novel-m0032-5p	XM_028668004.1	miR-2779-x	novel_circ_001815
XM_017055568.2	MAPK signaling pathway	novel-m0032-5p	XM_017048593.2	miR-2779-x	novel_circ_001816
XM_017062903.2	MAPK signaling pathway	miR-6001-y	XM_017051964.2	miR-2779-x	novel_circ_002321
XM_017063980.2	MAPK signaling pathway	miR-6001-y	XM_017063980.2	miR-2779-x	novel_circ_002881
XM_028667636.1	MAPK signaling pathway	miR-6001-y	XM_017052795.2	miR-5106-y	novel_circ_001747
XM_017059464.2	MAPK signaling pathway	miR-4451-y	XM_017062903.2	miR-5106-y	novel_circ_002747
XM_017056787.2	MAPK signaling pathway	miR-5106-y	XM_017062903.2	novel-m0006-5p	novel_circ_000095
XM_017055524.2	MAPK signaling pathway	miR-6001-y	XM_028667636.1	novel-m0006-5p	novel_circ_000401
XM_017052856.2	MAPK signaling pathway	miR-6001-y	XM_017056787.2	novel-m0006-5p	novel_circ_001653
XM_017050145.2	MAPK signaling pathway	miR-6001-y	XM_017050145.2	novel-m0006-5p	novel_circ_001747
XM_017048388.2	MAPK signaling pathway	miR-6001-y	XM_017048388.2	novel-m0006-5p	novel_circ_002206
XM_017051652.2	MAPK signaling pathway	miR-980-y	XM_028667636.1	novel-m0006-5p	novel_circ_002321
XM_028665909.1	MAPK signaling pathway	novel-m0006-5p	XM_028665909.1	novel-m0006-5p	novel_circ_002624
XM_017048593.2	MAPK signaling pathway	novel-m0032-5p	XM_017062828.2	novel-m0006-5p	novel_circ_002651
XM_017062828.2	MAPK signaling pathway	novel-m0032-5p	XM_017062635.2	novel-m0006-5p	novel_circ_003059
XM_017062635.2	MAPK signaling pathway	novel-m0032-5p	XM_028665909.1	novel-m0043-5p	novel_circ_000256
		novel-m0032-5p	XM_017050145.2	novel-m0043-5p	novel_circ_002616
		novel-m0044-5p	XM_017062635.2	miR-2770-y	novel_circ_000138
				miR-2770-y	novel_circ_000501
				miR-2770-y	novel_circ_000942
				miR-965-y	novel_circ_000138
				miR-965-y	novel_circ_000571
				miR-965-y	novel_circ_001649
				miR-965-y	novel_circ_002233
				miR-965-y	novel_circ_002321
				miR-980-y	novel_circ_000093
				miR-980-y	novel_circ_000501
				miR-980-y	novel_circ_001148
				miR-980-y	novel_circ_002737
				miR-980-y	novel_circ_002747
				miR-2765-x	novel_circ_000401
				miR-2765-x	novel_circ_000589
				miR-2765-x	novel_circ_002206
				miR-2765-x	novel_circ_002321
				miR-2765-x	novel_circ_002635
				novel-m0044-5p	novel_circ_000609
				novel-m0044-5p	novel_circ_002321
				novel-m0044-5p	novel_circ_002616
				miR-4451-y	novel_circ_000609

miR-4451-y	novel_circ_001010
miR-4451-y	novel_circ_001484

Table S7A. GO terms annotated by ORFs within DEcircRNAs in the 4-day-old comparison group

Ontology	Class	Gene number	Gene ID
Biological Process	locomotion	1	LOC107998974
Biological Process	developmental process	1	LOC107998974
Biological Process	cellular process	8	LOC107992482;LOC107995527;LOC107998974;LOC107999286;LOC107999482;LOC108000201;LOC108000569;LOC108002801
Biological Process	localization	3	LOC107999286;LOC108001520;LOC108002801
Biological Process	single-organism process	6	LOC107995527;LOC107998974;LOC107999286;LOC107999482;LOC108001667;LOC108002801
Biological Process	cellular component organization or biogenesis	1	LOC107998974
Biological Process	multicellular organismal process	1	LOC107998974
Biological Process	metabolic process	6	LOC107992482;LOC107995527;LOC107998974;LOC108000201;LOC108000569;LOC108001667
Biological Process	signaling	1	LOC107998974
Biological Process	response to stimulus	1	LOC107998974
Biological Process	biological regulation	2	LOC107998974;LOC108000201
Molecular Function	nucleic acid binding transcription factor activity	2	LOC107999482;LOC108000201
Molecular Function	transporter activity	2	LOC107999286;LOC108002801
Molecular Function	binding	8	LOC107994399;LOC107998974;LOC107999482;LOC108000201;LOC108000569;LOC108001667;LOC108002801;LOC108002845
Molecular Function	catalytic activity	6	LOC107992482;LOC107995527;LOC108000173;LOC108000569;LOC108001667;LOC108002801
Cellular Component	membrane-enclosed lumen	1	LOC107998974
Cellular Component	membrane	4	LOC107998401;LOC107998974;LOC107999286;LOC108002801
Cellular Component	macromolecular complex	2	LOC107998974;LOC107999286
Cellular Component	membrane part	3	LOC107998401;LOC107999286;LOC108002801
Cellular Component	organelle	3	LOC107998974;LOC107999482;LOC108000201
Cellular Component	organelle part	1	LOC107998974
Cellular Component	cell	3	LOC107998974;LOC107999482;LOC108000201
Cellular Component	cell part	3	LOC107998974;LOC107999482;LOC108000201

Table S7B. GO terms annotated by ORFs within DEcircRNAs in the 5-day-old comparison group

Ontology	Class	Gene number	Gene ID
Biological Process	cellular process	10	LOC107995253;LOC107992482;LOC107996539;LOC107998795;LOC107999286;LOC108001033;LOC108002511;LOC108002801;LOC108003146;LOC108003247
Biological Process	response to stimulus	3	LOC107995253;LOC108002511;LOC108003146
Biological Process	localization	3	LOC107995620;LOC107999286;LOC108002801
Biological Process	signaling	2	LOC108002511;LOC108003146
Biological Process	single-organism process	7	LOC107996539;LOC107997992;LOC107999286;LOC108001033;LOC108002511;LOC108002801;LOC108003146
Biological Process	multicellular organismal process	1	LOC107997992
Biological Process	metabolic process	7	LOC107995253;LOC107992482;LOC107995620;LOC107998795;LOC108001033;LOC108002511;LOC108003247
Biological Process	biological regulation	3	LOC108001352;LOC108002511;LOC108003146
Molecular Function	transporter activity	2	LOC107999286;LOC108002801
Molecular Function	molecular transducer activity	2	LOC107995620;LOC108003146
Molecular Function	nucleic acid binding transcription factor activity	1	LOC108002511
Molecular Function	binding	9	LOC107995253;LOC107994550;LOC107995620;LOC107998795;LOC108001033;LOC108001352;LOC108001569;LOC108002801;LOC108003247
Molecular Function	signal transducer activity	1	LOC108003146
Molecular Function	catalytic activity	7	LOC107995253;LOC107992482;LOC107995620;LOC107998795;LOC108001033;LOC108002801;LOC108003247
Cellular Component	membrane part	4	LOC107997992;LOC107999286;LOC108002801;LOC108003146

Cellular Component	membrane	4	LOC107997992;LOC107999286;LOC108002801;LOC108003146
Cellular Component	macromolecular complex	2	LOC107999286;LOC108002511
Cellular Component	organelle	3	LOC107994550;LOC108001352;LOC108002511
Cellular Component	cell	3	LOC107994550;LOC108001352;LOC108002511
Cellular Component	cell part	3	LOC107994550;LOC108001352;LOC108002511

Table S7C. GO terms annotated by ORFs within DEcircRNAs in the 6-day-old comparison group

Ontology	Class	Gene number	Gene ID
Biological Process	biological regulation	4	LOC108003778;LOC107995149;LOC107997067;LOC108002839
Biological Process	signaling	1	LOC108003778
Biological Process	response to stimulus	1	LOC108003778
Biological Process	localization	1	LOC107995620
Biological Process	metabolic process	3	LOC108003778;LOC108003484;LOC107995620
Biological Process	cellular process	3	LOC108003778;LOC108003484;LOC107993301
Biological Process	single-organism process	2	LOC108003778;LOC107993301
Molecular Function	nucleic acid binding transcription factor activity	2	LOC108003778;LOC107997067
Molecular Function	molecular transducer activity	2	LOC108003778;LOC107995620
Molecular Function	binding	8	LOC108003778;LOC108003484;LOC107993301;LOC107993704;LOC107994204;LOC107995149;LOC107995620;LOC107997067
Molecular Function	signal transducer activity	1	LOC108003778
Molecular Function	catalytic activity	6	LOC108003484;LOC107993301;LOC107994275;LOC107995620;LOC108000173;LOC108001561
Cellular Component	cell	5	LOC108003778;LOC107993301;LOC107995149;LOC107997067;LOC108002839
Cellular Component	cell part	5	LOC108003778;LOC107993301;LOC107995149;LOC107997067;LOC108002839
Cellular Component	organelle	4	LOC108003778;LOC107993301;LOC107995149;LOC107997067
Cellular Component	organelle part	1	LOC107993301
Cellular Component	macromolecular complex	1	LOC107993301

Table S8A. KEGG pathways annotated by ORFs within DEcircRNAs in the 4-day-old comparison group

KEGG A class	KEGG B class	Pathway	AcCK1 vs AcT1	All	*P* value	Q value	Pathway ID	Gene	K number
Organismal Systems	Endocrine system	Insulin signaling pathway	4	70	0.000564657	0.06041825	ko04910	LOC107994726;LOC107998974;LOC107999690;LOC108000569	K00871+K02183+K07200+K03083
Organismal Systems	Endocrine system	Glucagon signaling pathway	3	59	0.004284846	0.22923926	ko04922	LOC107994726;LOC107998974;LOC107999690	K00871+K02183+K07200
Organismal Systems	Development	Dorso-ventral axis formation	2	31	0.01320856	0.34810524	ko04320	LOC107998870;LOC108000995	K02098+K02360
Human Diseases	Infectious diseases	Tuberculosis	2	45	0.02680901	0.34810524	ko05152	LOC107998974;LOC107999286	K02183+K02154
Human Diseases	Cancers	Gastric cancer	2	51	0.03382833	0.34810524	ko05226	LOC108000569;LOC108002801	K03083+K05658
Organismal Systems	Sensory system	Phototransduction	1	6	0.03405711	0.34810524	ko04744	LOC107998974	K02183
Organismal Systems	Endocrine system	Melanogenesis	2	53	0.03631316	0.34810524	ko04916	LOC107998974;LOC108000569	K02183+K03083
Human Diseases	Infectious diseases	Kaposi sarcoma-associated herpesvirus infection	2	54	0.03758177	0.34810524	ko05167	LOC107998974;LOC108000569	K02183+K03083

Organismal Systems	Nervous system	Neurotrophin signaling pathway	2	57	0.04148975	0.34810524	ko04722	LOC107998974;LOC108000569	K02183+K03083
Human Diseases	Endocrine and metabolic diseases	Insulin resistance	2	57	0.04148975	0.34810524	ko04931	LOC107999690;LOC108000569	K07200+K03083
Environmental Information Processing	Signal transduction	Hippo signaling pathway -fly	2	58	0.04282575	0.34810524	ko04391	LOC108000798;LOC108003317	K16507+K02306
Metabolism	Glycan biosynthesis and metabolism	Glycosaminoglycan biosynthesis - chondroitin sulfate / dermatan sulfate	1	8	0.04516316	0.34810524	ko00532	LOC108000170	K13499
Organismal Systems	Environmental adaptation	Circadian rhythm - fly	1	8	0.04516316	0.34810524	ko04711	LOC108000569	K03083
Environmental Information Processing	Signal transduction	Apelin signaling pathway	2	60	0.04554648	0.34810524	ko04371	LOC107998974;LOC107999690	K02183+K07200
Organismal Systems	Endocrine system	Oxytocin signaling pathway	2	65	0.0526229	0.35460661	ko04921	LOC107998974;LOC107999690	K02183+K07200
Organismal Systems	Nervous system	Dopaminergic synapse	2	66	0.05408363	0.35460661	ko04728	LOC107998974;LOC108000569	K02183+K03083
Environmental Information Processing	Signal transduction	Calcium signaling pathway	2	70	0.06007139	0.35460661	ko04020	LOC107994726;LOC107998974	K00871+K02183
Organismal Systems	Excretory system	Collecting duct acid secretion	1	11	0.06159568	0.35460661	ko04966	LOC107999286	K02154
Environmental Information Processing	Signal transduction	Wnt signaling pathway	2	73	0.06470887	0.35460661	ko04310	LOC108000569;LOC108003317	K03083+K02306
Human Diseases	Cancers	MicroRNAs in cancer	2	74	0.06628161	0.35460661	ko05206	LOC108000201;LOC108002801	K09409+K05658
Organismal Systems	Environmental adaptation	Circadian rhythm	1	13	0.07240146	0.36155738	ko04710	LOC107999690	K07200
Human Diseases	Infectious diseases	Human cytomegalovirus infection	2	79	0.0743389	0.36155738	ko05163	LOC107998974;LOC108000569	K02183+K03083
Metabolism	Metabolism of cofactors and vitamins	Thiamine metabolism	1	15	0.08308929	0.37808471	ko00730	LOC107992482	K01077
Metabolism	Glycan biosynthesis and metabolism	Other glycan degradation	1	18	0.09890252	0.37808471	ko00511	LOC108001667	K01191
Environmental Information Processing	Membrane transport	ABC transporters	1	18	0.09890252	0.37808471	ko02010	LOC108002801	K05658
Human Diseases	Immune diseases	Rheumatoid arthritis	1	19	0.1041159	0.37808471	ko05323	LOC107999286	K02154
Metabolism	Carbohydrate metabolism	Starch and sucrose metabolism	1	20	0.1093008	0.37808471	ko00500	LOC107995527	K01196
Human Diseases	Endocrine and metabolic diseases	Non-alcoholic fatty liver disease (NAFLD)	2	99	0.1094148	0.37808471	ko04932	LOC107999690;LOC108000569	K07200+K03083
Environmental Information Processing	Signal transduction	Hedgehog signaling pathway	1	22	0.1195853	0.37808471	ko04340	LOC108000569	K03083
Environmental Information Processing	Signal transduction	Hippo signaling pathway - multiple species	1	22	0.1195853	0.37808471	ko04392	LOC108000798	K16507
Organismal Systems	Sensory system	Olfactory transduction	1	22	0.1195853	0.37808471	ko04740	LOC107998974	K02183

Human Diseases	Infectious diseases	Pertussis	1	22	0.1195853	0.37808471	ko05133	LOC107998974	K02183
Metabolism	Metabolism of cofactors and vitamins	Folate biosynthesis	1	24	0.1297572	0.37808471	ko00790	LOC107992482	K01077
Organismal Systems	Immune system	B cell receptor signaling pathway	1	24	0.1297572	0.37808471	ko04662	LOC108000569	K03083
Human Diseases	Cancers	Basal cell carcinoma	1	24	0.1297572	0.37808471	ko05217	LOC108000569	K03083
Organismal Systems	Immune system	IL-17 signaling pathway	1	25	0.1348013	0.37808471	ko04657	LOC108000569	K03083
Environmental Information Processing	Signal transduction	Hedgehog signaling pathway - fly	1	26	0.1398177	0.37808471	ko04341	LOC108000569	K03083
Organismal Systems	Endocrine system	Prolactin signaling pathway	1	26	0.1398177	0.37808471	ko04917	LOC108000569	K03083
Organismal Systems	Endocrine system	Adipocytokine signaling pathway	1	26	0.1398177	0.37808471	ko04920	LOC107999690	K07200
Human Diseases	Cancers	Glioma	1	27	0.1448066	0.37808471	ko05214	LOC107998974	K02183
Human Diseases	Neurodegenerative diseases	Alzheimer disease	2	119	0.1480897	0.37808471	ko05010	LOC107998974;LOC108000569	K02183+K03083
Human Diseases	Cancers	Endometrial cancer	1	28	0.149768	0.37808471	ko05213	LOC108000569	K03083
Human Diseases	Drug resistance	EGFR tyrosine kinase inhibitor resistance	1	31	0.1644892	0.37808471	ko01521	LOC108000569	K03083
Human Diseases	Infectious diseases	Human papillomavirus infection	2	128	0.1663646	0.37808471	ko05165	LOC107999286;LOC108000569	K02154+K03083
Organismal Systems	Sensory system	Phototransduction - fly	1	32	0.1693424	0.37808471	ko04745	LOC107998974	K02183
Human Diseases	Cardiovascular diseases	Hypertrophic cardiomyopathy (HCM)	1	32	0.1693424	0.37808471	ko05410	LOC107999690	K07200
Organismal Systems	Nervous system	Long-term potentiation	1	34	0.1789687	0.37808471	ko04720	LOC107998974	K02183
Organismal Systems	Endocrine system	Renin secretion	1	34	0.1789687	0.37808471	ko04924	LOC107998974	K02183
Human Diseases	Infectious diseases	Hepatitis C	1	34	0.1789687	0.37808471	ko05160	LOC108000569	K03083
Human Diseases	Infectious diseases	Measles	1	34	0.1789687	0.37808471	ko05162	LOC108000569	K03083
Organismal Systems	Immune system	C-type lectin receptor signaling pathway	1	35	0.1837421	0.37808471	ko04625	LOC107998974	K02183
Organismal Systems	Immune system	T cell receptor signaling pathway	1	35	0.1837421	0.37808471	ko04660	LOC108000569	K03083
Organismal Systems	Nervous system	Synaptic vesicle cycle	1	37	0.1932102	0.39006588	ko04721	LOC107999286	K02154
Environmental Information Processing	Signal transduction	ErbB signaling pathway	1	39	0.2025742	0.39273846	ko04012	LOC108000569	K03083

Human Diseases	Infectious diseases	Vibrio cholerae infection	1	39	0.2025742	0.39273846	ko05110	LOC107999286	K02154
Organismal Systems	Digestive system	Bile secretion	1	40	0.2072175	0.39273846	ko04976	LOC108002801	K05658
Human Diseases	Cancers	Colorectal cancer	1	41	0.2118352	0.39273846	ko05210	LOC108000569	K03083
Metabolism	Carbohydrate metabolism	Amino sugar and nucleotide sugar metabolism	1	43	0.2209942	0.39273846	ko00520	LOC107994792	K01183
Human Diseases	Substance dependence	Amphetamine addiction	1	44	0.2255358	0.39273846	ko05031	LOC107998974	K02183
Human Diseases	Infectious diseases	Epithelial cell signaling in Helicobacter pylori infection	1	44	0.2255358	0.39273846	ko05120	LOC107999286	K02154
Organismal Systems	Environmental adaptation	Thermogenesis	2	160	0.2341301	0.39273846	ko04714	LOC107998414;LOC107999690	K15601+K07200
Organismal Systems	Sensory system	Inflammatory mediator regulation of TRP channels	1	46	0.2345439	0.39273846	ko04750	LOC107998974	K02183
Organismal Systems	Digestive system	Gastric acid secretion	1	46	0.2345439	0.39273846	ko04971	LOC107998974	K02183
Organismal Systems	Digestive system	Salivary secretion	1	48	0.2434527	0.39273846	ko04970	LOC107998974	K02183
Organismal Systems	Aging	Longevity regulating pathway - mammal	1	51	0.2566318	0.39273846	ko04211	LOC107999690	K07200
Organismal Systems	Endocrine system	GnRH signaling pathway	1	52	0.2609763	0.39273846	ko04912	LOC107998974	K02183
Organismal Systems	Endocrine system	Estrogen signaling pathway	1	52	0.2609763	0.39273846	ko04915	LOC107998974	K02183
Human Diseases	Cancers	Prostate cancer	1	52	0.2609763	0.39273846	ko05215	LOC108000569	K03083
Human Diseases	Cancers	Breast cancer	1	52	0.2609763	0.39273846	ko05224	LOC108000569	K03083
Organismal Systems	Aging	Longevity regulating pathway - multiple species	1	53	0.2652968	0.39273846	ko04213	LOC107999690	K07200
Cellular Processes	Cellular community - eukaryotes	Signaling pathways regulating pluripotency of stem cells	1	53	0.2652968	0.39273846	ko04550	LOC108000569	K03083
Human Diseases	Cardiovascular diseases	Fluid shear stress and atherosclerosis	1	55	0.273866	0.39273846	ko05418	LOC107998974	K02183
Cellular Processes	Transport and catabolism	Phagosome	1	57	0.2823405	0.39273846	ko04145	LOC107999286	K02154
Organismal Systems	Endocrine system	Aldosterone synthesis and secretion	1	58	0.2865425	0.39273846	ko04925	LOC107998974	K02183
Organismal Systems	Immune system	Chemokine signaling pathway	1	59	0.2907212	0.39273846	ko04062	LOC108000569	K03083
Organismal Systems	Circulatory system	Vascular smooth muscle contraction	1	59	0.2907212	0.39273846	ko04270	LOC107998974	K02183
Human Diseases	Cancers	Pathways in cancer	2	187	0.2929279	0.39273846	ko05200	LOC107998974;LOC108000569	K02183+K03083

Environmental Information Processing	Signal transduction	FoxO signaling pathway	1	60	0.2948767	0.39273846	ko04068	LOC107999690	K07200
Environmental Information Processing	Signal transduction	Phosphatidylinositol signaling system	1	60	0.2948767	0.39273846	ko04070	LOC107998974	K02183
Organismal Systems	Environmental adaptation	Circadian entrainment	1	60	0.2948767	0.39273846	ko04713	LOC107998974	K02183
Organismal Systems	Development	Axon guidance	1	65	0.3153105	0.39273846	ko04360	LOC108000569	K03083
Environmental Information Processing	Signal transduction	AMPK signaling pathway	1	66	0.3193294	0.39273846	ko04152	LOC107999690	K07200
Organismal Systems	Circulatory system	Adrenergic signaling in cardiomyocytes	1	66	0.3193294	0.39273846	ko04261	LOC107998974	K02183
Cellular Processes	Cellular community - eukaryotes	Tight junction	1	66	0.3193294	0.39273846	ko04530	LOC107999690	K07200
Organismal Systems	Endocrine system	Thyroid hormone signaling pathway	1	66	0.3193294	0.39273846	ko04919	LOC108000569	K03083
Human Diseases	Substance dependence	Alcoholism	1	66	0.3193294	0.39273846	ko05034	LOC107998974	K02183
Human Diseases	Infectious diseases	Influenza A	1	66	0.3193294	0.39273846	ko05164	LOC108000569	K03083
Cellular Processes	Cell growth and death	Cellular senescence	1	69	0.3312524	0.4027728	ko04218	LOC107998974	K02183
Environmental Information Processing	Signal transduction	Hippo signaling pathway	1	70	0.3351825	0.40297222	ko04390	LOC108000569	K03083
Cellular Processes	Cell growth and death	Oocyte meiosis	1	73	0.3468421	0.40758167	ko04114	LOC107998974	K02183
Human Diseases	Cancers	Hepatocellular carcinoma	1	73	0.3468421	0.40758167	ko05225	LOC108000569	K03083
Environmental Information Processing	Signal transduction	cGMP - PKG signaling pathway	1	74	0.3506854	0.40758167	ko04022	LOC107998974	K02183
Cellular Processes	Cell growth and death	Cell cycle	1	76	0.3583078	0.40758167	ko04110	LOC108000569	K03083
Cellular Processes	Transport and catabolism	Lysosome	1	76	0.3583078	0.40758167	ko04142	LOC107999286	K02154
Environmental Information Processing	Signal transduction	Rap1 signaling pathway	1	78	0.3658454	0.40758167	ko04015	LOC107998974	K02183
Cellular Processes	Cellular community - eukaryotes	Focal adhesion	1	78	0.3658454	0.40758167	ko04510	LOC108000569	K03083
Human Diseases	Endocrine and metabolic diseases	Cushing syndrome	1	79	0.3695827	0.40758167	ko04934	LOC108000569	K03083
Environmental Information Processing	Signal transduction	MAPK signaling pathway - fly	1	80	0.3732991	0.40758167	ko04013	LOC108002492	K10176
Environmental Information Processing	Signal transduction	Ras signaling pathway	1	82	0.3806697	0.41143089	ko04014	LOC107998974	K02183
Environmental Information Processing	Signal transduction	mTOR signaling pathway	1	87	0.3987386	0.4266503	ko04150	LOC108000569	K03083
Metabolism	Energy metabolism	Oxidative phosphorylation	1	94	0.42319	0.4475	ko0019	LOC107999286	K02154

					77	2635	0		
Environmental Information Processing	Signal transduction	cAMP signaling pathway	1	95	0.4266139	0.44752635	ko04024	LOC107998974	K02183
Environmental Information Processing	Signal transduction	PI3K-Akt signaling pathway	1	100	0.4434094	0.46062918	ko04151	LOC108000569	K03083
Human Diseases	Infectious diseases	Epstein-Barr virus infection	1	102	0.4499962	0.46297686	ko05169	LOC108000569	K03083
Human Diseases	Infectious diseases	HTLV-I infection	1	119	0.5030785	0.51266095	ko05166	LOC108000569	K03083
Metabolism	Global and overview maps	Metabolic pathways	5	828	0.5379515	0.54302651	ko01100	LOC107992482;LOC107994792;LOC107995527;LOC107999286;LOC108000170	K01077+K01183+K01196+K02154+K13499
Metabolism	Nucleotide metabolism	Purine metabolism	1	133	0.543111	0.543111	ko00230	LOC107998249	K01769

Table S8B. KEGG pathways annotated by ORFs within DEcircRNAs in the 5-day-old comparison group

KEGG_A_class	KEGG_B_class	Pathway	AcCK2-vs-AcT2 (13)	All (3302)	*P* value	Q value	Pathway ID	Genes	K_IDs
Organismal Systems	Endocrine system	Parathyroid hormone synthesis, secretion and action	2	46	0.01343327	0.2635337	ko04928	LOC107996802;LOC107998728	K09260+K09048
Cellular Processes	Cellular community - eukaryotes	Adherens junction	2	49	0.01516205	0.2635337	ko04520	LOC108001569;LOC108002511	K06084+K04501
Human Diseases	Infectious diseases	Hepatitis B	2	49	0.01516205	0.2635337	ko05161	LOC107998728;LOC108002511	K09048+K04501
Human Diseases	Cancers	Gastric cancer	2	51	0.01636567	0.2635337	ko05226	LOC108002511;LOC108002801	K04501+K05658
Organismal Systems	Endocrine system	Glucagon signaling pathway	2	59	0.02157588	0.2635337	ko04922	LOC107994726;LOC107998728	K00871+K09048
Environmental Information Processing	Signal transduction	Apelin signaling pathway	2	60	0.02227045	0.2635337	ko04371	LOC107996802;LOC108002511	K09260+K04501
Environmental Information Processing	Signal transduction	cGMP - PKG signaling pathway	2	74	0.03294638	0.3148051	ko04022	LOC107996802;LOC107998728	K09260+K09048
Environmental Information Processing	Signal transduction	MAPK signaling pathway - fly	2	80	0.0380365	0.3148051	ko04013	LOC107994550;LOC107996802	K09237+K09260
Organismal Systems	Excretory system	Collecting duct acid secretion	1	11	0.04252775	0.3148051	ko04966	LOC107999286	K02154
Metabolism	Metabolism of cofactors and vitamins	Thiamine metabolism	1	15	0.05757386	0.3148051	ko00730	LOC107992482	K01077
Environmental Information Processing	Membrane transport	ABC transporters	1	18	0.06871489	0.3148051	ko02010	LOC108002801	K05658
Human Diseases	Immune diseases	Rheumatoid arthritis	1	19	0.07240146	0.3148051	ko05323	LOC107999286	K02154
Organismal Systems	Immune system	Th17 cell differentiation	1	23	0.08701345	0.3148051	ko04659	LOC108002511	K04501
Human Diseases	Infectious diseases	Human papillomavirus infection	2	128	0.08799176	0.3148051	ko05165	LOC107998728;LOC107999286	K09048+K02154
Metabolism	Metabolism of cofactors and vitamins	Folate biosynthesis	1	24	0.0906331	0.3148051	ko00790	LOC107992482	K01077
Genetic Information Processing	Replication and repair	Base excision repair	1	25	0.0942395	0.3148051	ko03410	LOC107995253	K10776
Organismal Systems	Excretory system	Vasopressin-regulated water reabsorption	1	25	0.0942395	0.3148051	ko04962	LOC107998728	K09048
Human Diseases	Cancers	Chronic myeloid leukemia	1	30	0.1120743	0.3148051	ko05220	LOC108002511	K04501
Organismal Systems	Development	Dorso-ventral axis formation	1	31	0.1156021	0.3148051	ko04320	LOC108000995	K02360
Human Diseases	Cancers	Pancreatic cancer	1	31	0.115602	0.31480	ko05212	LOC108002511	K04501

					1	51			
Organismal Systems	Endocrine system	Cortisol synthesis and secretion	1	32	0.119117	0.3148051	ko04927	LOC107998728	K09048
Human Diseases	Substance dependence	Cocaine addiction	1	32	0.119117	0.3148051	ko05030	LOC107998728	K09048
Human Diseases	Endocrine and metabolic diseases	AGE-RAGE signaling pathway in diabetic complications	1	35	0.1295844	0.3148051	ko04933	LOC108002511	K04501
Environmental Information Processing	Signal transduction	TGF-beta signaling pathway	1	36	0.1304479	0.3148051	ko04350	LOC108002511	K04501
Environmental Information Processing	Signal transduction	TNF signaling pathway	1	36	0.1304479	0.3148051	ko04668	LOC107998728	K09048
Organismal Systems	Nervous system	Synaptic vesicle cycle	1	37	0.1364988	0.3148051	ko04721	LOC107999286	K02154
Organismal Systems	Endocrine system	Thyroid hormone synthesis	1	38	0.1399369	0.3148051	ko04918	LOC107998728	K09048
Human Diseases	Infectious diseases	Vibrio cholerae infection	1	39	0.1433624	0.3148051	ko05110	LOC107999286	K02154
Organismal Systems	Digestive system	Bile secretion	1	40	0.1467753	0.3148051	ko04976	LOC108002801	K05658
Human Diseases	Cancers	Colorectal cancer	1	41	0.1501756	0.3148051	ko05210	LOC108002511	K04501
Metabolism	Carbohydrate metabolism	Amino sugar and nucleotide sugar metabolism	1	43	0.1569388	0.3148051	ko00520	LOC107994792	K01183
Human Diseases	Substance dependence	Amphetamine addiction	1	44	0.1603018	0.3148051	ko05031	LOC107998728	K09048
Human Diseases	Infectious diseases	Epithelial cell signaling in Helicobacter pylori infection	1	44	0.1603018	0.3148051	ko05120	LOC107999286	K02154
Human Diseases	Infectious diseases	Tuberculosis	1	45	0.1636523	0.3148051	ko05152	LOC107999286	K02154
Organismal Systems	Aging	Longevity regulating pathway - mammal	1	51	0.1834978	0.3148051	ko04211	LOC107998728	K09048
Organismal Systems	Endocrine system	Estrogen signaling pathway	1	52	0.1867628	0.3148051	ko04915	LOC107998728	K09048
Human Diseases	Cancers	Prostate cancer	1	52	0.1867628	0.3148051	ko05215	LOC107998728	K09048
Cellular Processes	Cellular community - eukaryotes	Signaling pathways regulating pluripotency of stem cells	1	53	0.1900158	0.3148051	ko04550	LOC108002511	K04501
Organismal Systems	Endocrine system	Melanogenesis	1	53	0.1900158	0.3148051	ko04916	LOC107998728	K09048
Organismal Systems	Nervous system	Cholinergic synapse	1	54	0.1932567	0.3148051	ko04725	LOC107998728	K09048
Human Diseases	Cardiovascular diseases	Fluid shear stress and atherosclerosis	1	55	0.1964856	0.3148051	ko05418	LOC107996802	K09260
Cellular Processes	Transport and catabolism	Phagosome	1	57	0.2029078	0.3148051	ko04145	LOC107999286	K02154
Organismal Systems	Endocrine system	Insulin secretion	1	57	0.2029078	0.3148051	ko04911	LOC107998728	K09048
Organismal Systems	Endocrine system	Relaxin signaling pathway	1	57	0.2029078	0.3148051	ko04926	LOC107998728	K09048
Human Diseases	Endocrine and metabolic diseases	Insulin resistance	1	57	0.2029078	0.3148051	ko04931	LOC107998728	K09048
Organismal Systems	Endocrine system	Aldosterone synthesis and secretion	1	58	0.2061011	0.3148051	ko04925	LOC107998728	K09048
Environmental Information Processing	Signal transduction	FoxO signaling pathway	1	60	0.2124523	0.3148051	ko04068	LOC108002511	K04501
Environmental Information Processing	Signal transduction	AMPK signaling pathway	1	66	0.2312254	0.3148051	ko04152	LOC107998728	K09048
Organismal Systems	Circulatory system	Adrenergic signaling in cardiomyocytes	1	66	0.2312254	0.3148051	ko04261	LOC107998728	K09048
Organismal Systems	Nervous system	Dopaminergic synapse	1	66	0.2312254	0.3148051	ko04728	LOC107998728	K09048

Human Diseases	Substance dependence	Alcoholism	1	66	0.2312254	0.3148051	ko05034	LOC107998728	K09048
Environmental Information Processing	Signal transduction	Calcium signaling pathway	1	70	0.2435105	0.3148051	ko04020	LOC107994726	K00871
Environmental Information Processing	Signal transduction	Hippo signaling pathway	1	70	0.2435105	0.3148051	ko04390	LOC108002511	K04501
Organismal Systems	Endocrine system	Insulin signaling pathway	1	70	0.2435105	0.3148051	ko04910	LOC107994726	K00871
Environmental Information Processing	Signal transduction	Wnt signaling pathway	1	73	0.252605	0.3148051	ko04310	LOC108002511	K04501
Human Diseases	Cancers	Hepatocellular carcinoma	1	73	0.252605	0.3148051	ko05225	LOC108002511	K04501
Human Diseases	Cancers	MicroRNAs in cancer	1	74	0.2556141	0.3148051	ko05206	LOC108002801	K05658
Cellular Processes	Cell growth and death	Cell cycle	1	76	0.2615986	0.3148051	ko04110	LOC108002511	K04501
Cellular Processes	Transport and catabolism	Lysosome	1	76	0.2615986	0.3148051	ko04142	LOC107999286	K02154
Human Diseases	Endocrine and metabolic diseases	Cushing syndrome	1	79	0.2704921	0.3148351	ko04934	LOC107998728	K09048
Human Diseases	Infectious diseases	Human cytomegalovirus infection	1	79	0.2704921	0.3148351	ko05163	LOC107998728	K09048
Human Diseases	Cancers	Viral carcinogenesis	1	84	0.2850953	0.3264801	ko05203	LOC107998728	K09048
Metabolism	Energy metabolism	Oxidative phosphorylation	1	94	0.3134955	0.3508702	ko00190	LOC107999286	K02154
Environmental Information Processing	Signal transduction	cAMP signaling pathway	1	95	0.3162774	0.3508702	ko04024	LOC107998728	K09048
Environmental Information Processing	Signal transduction	PI3K-Akt signaling pathway	1	100	0.3300319	0.3604964	ko04151	LOC107998728	K09048
Human Diseases	Infectious diseases	HTLV-I infection	1	119	0.3800026	0.4087907	ko05166	LOC108002511	K04501
Genetic Information Processing	Folding, sorting and degradation	Protein processing in endoplasmic reticulum	1	123	0.3900742	0.4133622	ko04141	LOC108001958	K10636
Human Diseases	Neurodegenerative diseases	Huntington disease	1	156	0.4675836	0.4882123	ko05016	LOC107998728	K09048
Organismal Systems	Environmental adaptation	Thermogenesis	1	160	0.4763336	0.4901404	ko04714	LOC107998728	K09048
Human Diseases	Cancers	Pathways in cancer	1	187	0.5320114	0.5396116	ko05200	LOC108002511	K04501
Metabolism	Global and overview maps	Metabolic pathways	3	828	0.6701406	0.6701406	ko01100	LOC107992482;LOC107994792;LOC107999286	K01077+K01183+K02154

Table S8C. KEGG pathways annotated by ORFs within DEcircRNAs in the 6-day-old comparison group

KEGG_A_class	KEGG_B_class	Pathway	AcCK3-vs-AcT3 (9)	All (3302)	*P* value	Q value	Pathway ID	Genes	K_IDs
Metabolism	Glycan biosynthesis and metabolism	Glycosaminoglycan biosynthesis - chondroitin sulfate / dermatan sulfate	1	8	0.02162079	0.2553965	ko00532	LOC108000170	K13499
Human Diseases	Infectious diseases	HTLV-I infection	2	119	0.03928392	0.2553965	ko05166	LOC107995510;LOC108002839	K12076+K15309
Organismal Systems	Immune system	T cell receptor signaling pathway	1	35	0.0915568	0.2553965	ko04660	LOC107995510	K12076
Organismal Systems	Nervous system	Synaptic vesicle cycle	1	37	0.09655587	0.2553965	ko04721	LOC107997812	K18211
Metabolism	Carbohydrate metabolism	Amino sugar and nucleotide sugar metabolism	1	43	0.1114067	0.2553965	ko00520	LOC107994792	K01183
Organismal Systems	Digestive system	Protein digestion and absorption	1	45	0.1163085	0.2553965	ko04974	LOC107994275	K01278
Cellular Processes	Cell growth and death	Apoptosis - fly	1	51	0.1308701	0.2553965	ko04214	LOC107997067	K09428

Organismal Systems	Endocrine system	Insulin secretion	1	57	0.145218	0.2553965	ko04911	LOC107997812	K18211
Environmental Information Processing	Signal transduction	Hippo signaling pathway -fly	1	58	0.1475887	0.2553965	ko04391	LOC107995510	K12076
Cellular Processes	Cellular community - eukaryotes	Tight junction	1	66	0.1663452	0.2553965	ko04530	LOC107995510	K12076
Environmental Information Processing	Signal transduction	Hippo signaling pathway	1	70	0.1755851	0.2553965	ko04390	LOC107995510	K12076
Human Diseases	Cancers	Viral carcinogenesis	1	84	0.2072123	0.2762831	ko05203	LOC107995510	K12076
Cellular Processes	Transport and catabolism	Endocytosis	1	121	0.2856699	0.3425266	ko04144	LOC107999220	K12485
Human Diseases	Infectious diseases	Human papillomavirus infection	1	128	0.2997108	0.3425266	ko05165	LOC107995510	K12076
Organismal Systems	Environmental adaptation	Thermogenesis	1	160	0.360824	0.3848789	ko04714	LOC107998414	K15601
Metabolism	Global and overview maps	Metabolic pathways	2	828	0.7018418	0.7018418	ko01100	LOC107994792;LOC108000170	K01183+K13499
